# Organelle‐Resolved Tetrazine‐*trans*‐Cyclooctene Click Chemistry for Cargo Delivery and Release

**DOI:** 10.1002/chem.71198

**Published:** 2026-06-01

**Authors:** Oleh Durydivka, Marek Chovanec, Rastislav Dzijak, Michal Rahm, Jachym Hrusak, Paul E. Reyes‐Gutierrez, Milan Vrabel

**Affiliations:** ^1^ Department of Bioorganic and Medicinal Chemistry Institute of Organic Chemistry and Biochemistry of the Czech Academy of Sciences Prague Czech Republic

**Keywords:** click chemistry, doxorubicin, drug delivery, inverse‐electron‐demand Diels‐Alder reaction, live‐cell imaging, organelle targeting

## Abstract

Accurate delivery of small molecules to specific cell compartments or organelles offers great potential for bioimaging and targeted therapy but remains challenging to predict and control. In this study, we evaluate a modular strategy for organelle‐directed delivery based on organelle‐targeting groups combined with tetrazine‐*trans*‐cyclooctene bioorthogonal chemistry. We demonstrate that organelle‐targeting probes bearing tetrazine or *trans*‐cyclooctene can efficiently undergo inverse‐electron‐demand Diels‐Alder reactions within various cellular compartments, including the plasma membrane, mitochondria, nucleus, endoplasmic reticulum, and lysosomes. Using this method, we localize fluorophores and the cytotoxic drug doxorubicin to specific organelles in living cells. We further show that tetrazine‐ or *trans*‐cyclooctene‐modified doxorubicin prodrugs restore cytotoxic activity upon click‐to‐release activation inside cells. However, direct comparisons of different probe architectures revealed that organelle targeting and reaction efficiency are highly sensitive to structural context, cargo type, and subcellular environment, and cannot be predicted solely from targeting motifs. Overall, these findings establish a framework for comparing organelle‐targeted bioorthogonal chemistry and offer practical guidance for designing probes and prodrugs that enable precise spatiotemporal control of small‐molecule activity in living cells.

## Graphical abstract

Bioorthogonal click chemistry tools provide a means for specific intracellular conjugation of molecules. In this study, we used reactive tetrazine (Tz) and TCO moieties for labeling of organelles and organelle‐specific delivery and activation of doxorubicin prodrugs.

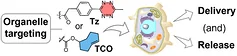

## 1 Introduction

Eukaryotic cells rely on membrane‐bound organelles to coordinate biochemical reactions in space and time, enabling tight control over metabolism, signaling, and cellular homeostasis [1]. Disruption of this compartmentalization is implicated in diverse diseases, with examples including mitochondria‐linked neuromuscular and neuroophthalmic syndromes [2, 3, 4], the endoplasmic reticulum (ER) stress‐associated metabolic and neurodegenerative diseases and cancer [5, 6, 7], lysosomal storage disorders [8], and Golgi apparatus dysfunction in neurodegenerative diseases [9]. Given the tight connection between organelle dysfunction and disease, the precise subcellular delivery of therapeutics has emerged as a promising strategy to enhance their therapeutic efficacy, improve selectivity, and minimize off‐target toxicity [10, 11, 12].

A common strategy to achieve subcellular specificity is to attach molecules of interest to organelle‐targeting groups, which exploit physicochemical biases directing them to a specific organelle or compartment [13]. For example, lipophilic cations such as triphenylphosphonium are often incorporated to achieve mitochondrial targeting [14, 15], weakly basic amines (such as morpholine) are used for lysosomal targeting [16], and peptide signals/retention sequences or specific protein binders are used for targeting the nucleus and the ER [17, 18].

Despite the broad use of organelle‐targeting groups, predictive rules for designing such groups/probes remain elusive because their performance depends on cell context, probe charge/hydrophobicity, local microenvironment (pH, ΔΨ, redox state), and competing sinks (e.g., lysosomal sequestration). Structural modifications, such as the incorporation of linkers, fluorophores, or therapeutic cargos, can alter probe parameters and thereby intracellular distribution. Consequently, the behavior of an organelle‐targeting group cannot be assumed to remain constant once functional moieties are added, motivating systematic, side‐by‐side comparisons under controlled, matched cellular conditions [19, 20, 21].

To pair localization with on‐site chemistry, tetrazine‐*trans*‐cyclooctene (Tz‐TCO) inverse‐electron‐demand Diels‐Alder (IEDDA) reactions offer exceptional speed and selectivity in live cells. Foundational studies have demonstrated very fast second‐order rates and broad biocompatibility of this reaction, enabling labeling at low micromolar to nanomolar concentrations [22, 23, 24, 25]. Click‐to‐release variants of the Tz‐TCO reaction transform ligation into activation: the IEDDA step triggers cleavage of TCO‐linked carbamates/ethers to unmask drugs or reporters precisely where tetrazine is encountered. Pioneering and evolving work (including antibody‐drug conjugates, prodrugs, and radioimmunoconjugates) demonstrates in vivo feasibility and continual kinetic improvements via sTCO linkers and advanced tetrazines [26, 27, 28, 29]. Integrating organelle‐targeting motifs with Tz‐TCO click‐to‐release chemistry enables spatially‐confined drug uncaging, thereby coupling subcellular localization and chemical activation.

The combination of targeting groups and localized chemistry constitutes a modular delivery platform, in which (I) an organelle‐targeting group directs the construct to a defined compartment, (II) a Tz or TCO handle enables ultrafast orthogonal reactivity, and (III) a payload (imaging agent, cytotoxin, or therapeutic) is either retained after ligation or released on demand. Although powerful in principle, such a system faces several challenges. As discussed above, the behavior of an organelle‐targeting motif cannot be assumed to remain intact once a Tz or TCO is appended, as these handles alter hydrophobicity, charge distribution, and steric profile, potentially disrupting membrane permeability, ΔΨ‐driven uptake, or leading to nonspecific sequestration. A second issue is whether Tz‐TCO reactions operate efficiently within the heterogeneous physicochemical environments of the nucleus, ER, Golgi, mitochondria, and lysosomes. Although IEDDA reactions exhibit exceptional intrinsic kinetics in solution, Tz stability may vary with pH and redox state [30]. TCO isomerization might be accelerated in thiol‐rich environments [31], and macromolecular crowding may restrict encounter rates [13, 24, 32]. Thus, whether click chemistry proceeds uniformly across organelles remains unclear. Despite growing interest in organelle‐targeted probes and Tz‐TCO chemistry, systematic, head‐to‐head comparisons within a unified framework are missing, and most available studies differ in probe architectures, cell models, and assay conditions.

In this work, we address these gaps by synthesizing and evaluating a panel of organelle‐targeting group‐Tz/TCO probes spanning the plasma membrane (PM), mitochondria, nucleus, ER, Golgi, and lysosomes. We quantify their subcellular localization and click performance in live cells under standardized conditions, demonstrating compartment‐specific delivery and on‐site activation of a model cytotoxic agent, doxorubicin.

## 2 Results and Discussion

### 2.1 Design of Organelle‐Targeting Probes and Cell Labeling Experiments

**FIGURE 1:**
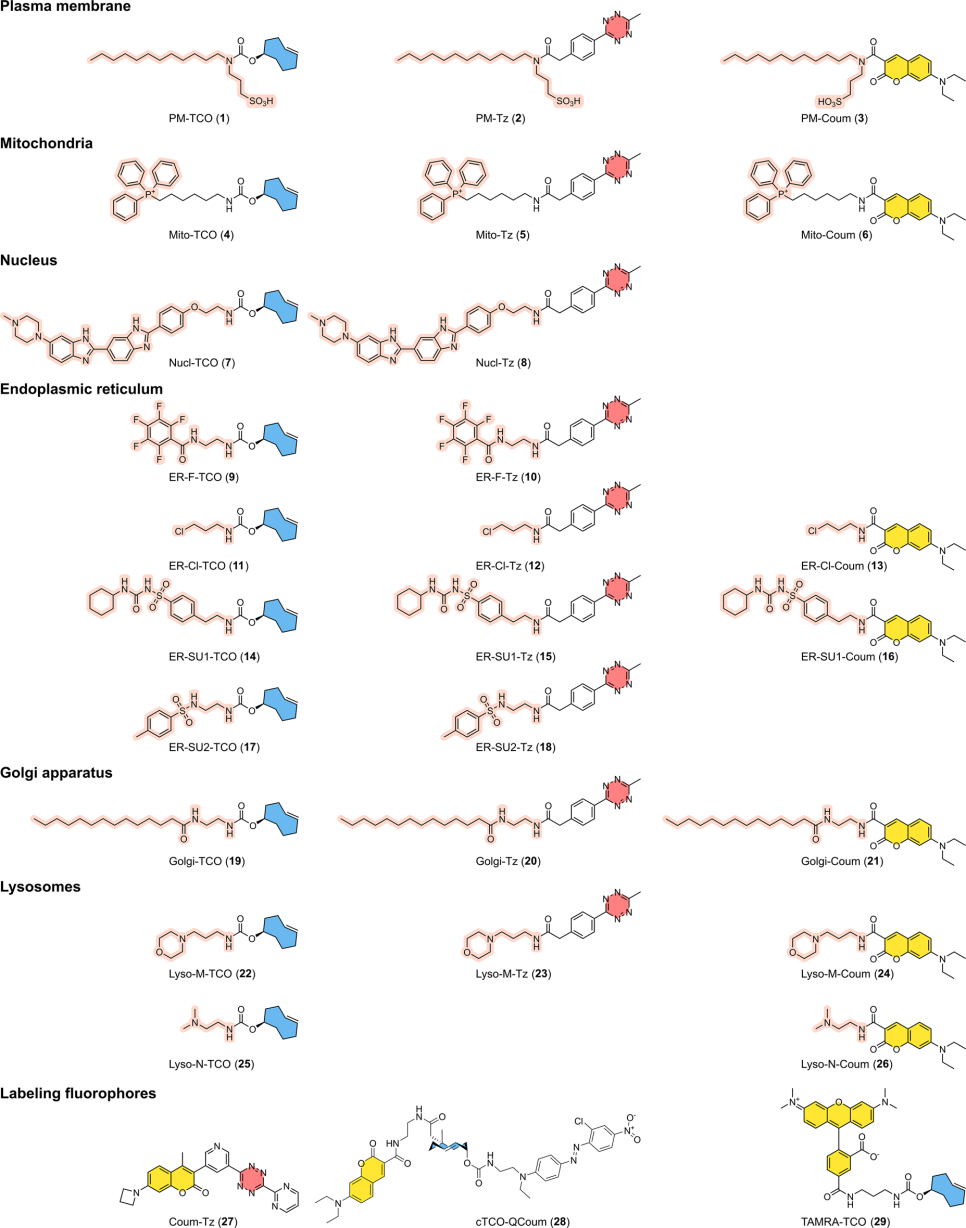
Structures of the organelle‐targeting probes and labeling fluorophores used in this study. The organelle‐targeting groups are highlighted in pink, *trans*‐cyclooctene (TCO) in blue, tetrazine (Tz) in red, and the fluorophores in yellow.

To create probes targeting specific organelles/compartments, we selected targeting motifs based on published structures and structures of commercially used organelle trackers (Figure 1 and Table S1). We included 1,2,4,5‐tetrazine (Tz) or TCO as bioorthogonal groups in our organelle‐targeting probes. In addition, we prepared probes bearing a coumarin fluorophore instead of a bioorthogonal group (Figure 1), allowing direct visualization without the need for click‐mediated labeling. To visualize the organelle‐targeting probe localization in cells, we used previously developed fluorogenic coumarin‐tetrazine Coum‐Tz (**27**) and cTCO‐QCoum (**28**) probes (Figure 1), which generate fluorescence only upon click reaction, enabling convenient wash‐free labeling and visualization of the Tz/TCO‐containing probes in live cells with a high signal‐to‐noise ratio [33, 34, 35, 36, 37]. We previously demonstrated that such coumarin‐based probes are reactive in various compartments, including the extracellular space, cytoplasm, mitochondria, and the nucleus [33, 38, 39]. The main advantages of the coumarin fluorophore are cell permeability and the lack of a strong preference for any compartment, as opposed to rhodamine or cyanine dyes known to target mitochondria (the principle used in MitoTrackers) and some rhodol derivatives targeting the ER [40]. To visualize the Tz‐containing probes, we also used the nonfluorogenic TAMRA‐TCO (**29**) probe. This derivative of rhodamine has been successfully used by Murrey and colleagues for fluorescent labeling of HaloTag‐Tz ligands in cellular experiments [41], demonstrating low preference for mitochondria. In addition, TAMRA has good cellular diffusivity [42] and a low background staining even during in vivo labeling [43].

Labeling and imaging were performed in live cells. To assess probe performance, we compared the staining generated by the click labeling of the organelle‐targeting probe with that of the reference control for the same organelle. For each organelle, we employed two types of references: genetically encoded protein markers and commercially available chemical trackers (Table S1). We demonstrate the colocalization of the best‐performing probes with the organelle controls by creating merged red‐and‐green‐channel composites, 2D histograms, and intensity profiles. We also calculated the colocalization coefficients: Pearson's correlation coefficient (PCC) for colocalization with genetically‐encoded markers (designated PCC_gen_) and chemical trackers (PCC_chem_), and, similarly, M1 Manders' colocalization coefficients (click ligation vs. control) for colocalization (MCC_gen_ and MCC_chem_).

### 2.2 PM Targeting

**FIGURE 2:**
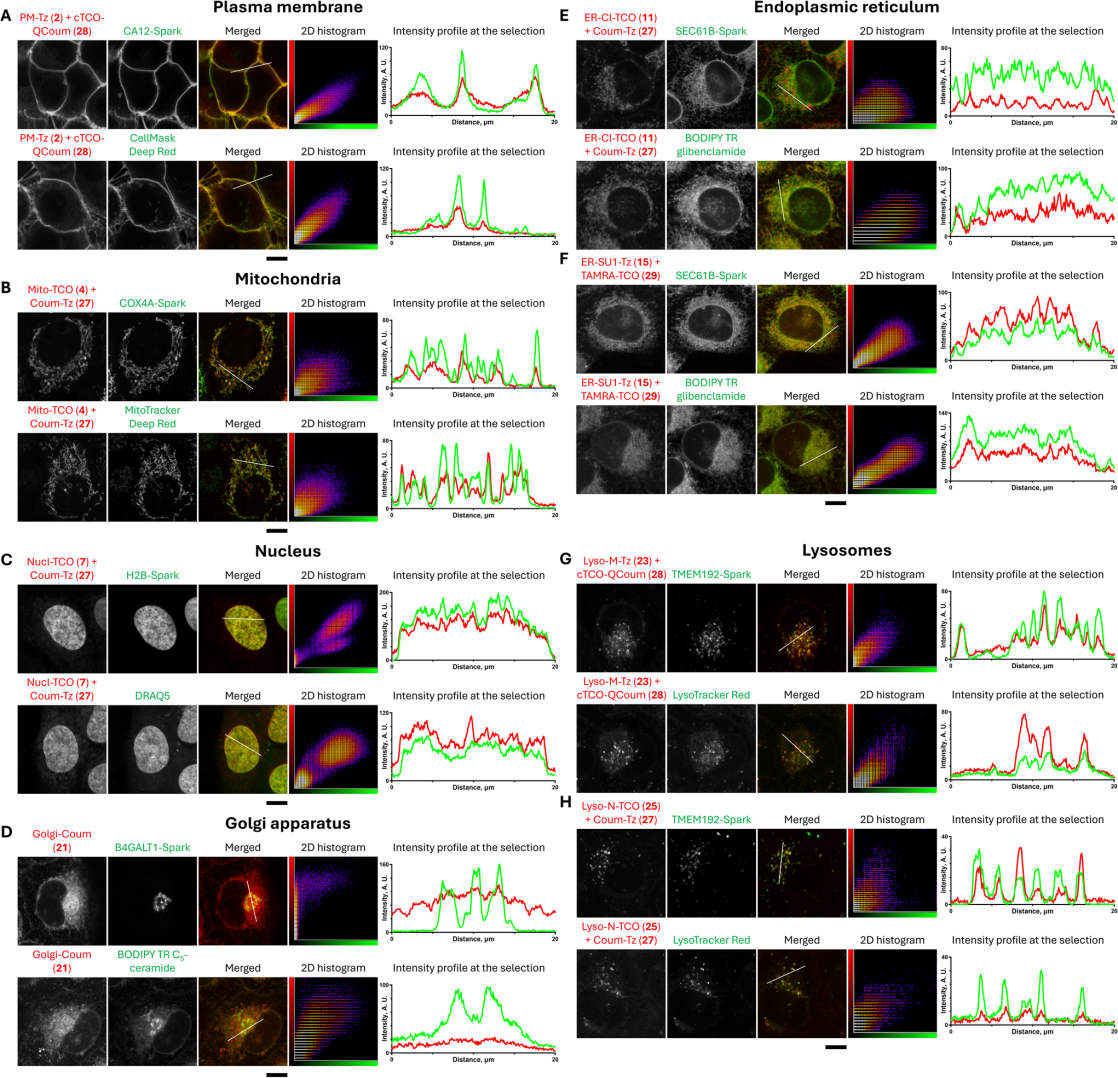
Subcellular localization of the selected organelle‐targeting probes. Organelle‐targeting probes for the PM (A), mitochondria (B), nucleus (C), Golgi apparatus (D), ER (E, F), and lysosomes (G, H) were incubated with U2OS cells (at 10 µM, except 1 µM for Mito‐TCO (**4**); for 30 min at 37°C, except 5 min for PM‐Tz (**2**) at room temperature and 30 min at 4°C for Golgi‐Coum (**21**)). After this, the probes were labeled with fluorophores containing the compatible bioorthogonal group at 10 µM for 30 min at 37°C, except 5 min at room temperature for the PM‐Tz (**2**) labeling. The intensity from such labeling is shown in merged images in red. The U2OS cells either stably expressed the Spark‐labeled organelle markers or the nontransfected U2OS cells were incubated with chemical trackers (as in Table S1); the intensities from these markers/trackers are shown in merged images in green. Detailed experimental procedures are provided in the Supporting Information. Additional microscopy images, including controls, are provided in Figures S2–7. The 2D histograms reflect the correlation of intensities in the red and green channels. Intensity profiles illustrate changes in intensity in both channels along the indicated line in the merged images. Scale bar 10 µm.

For the PM‐targeting probes, we used an 11‐carbon lipophilic sulfonyl derivative [44], and, as controls, we used carbonic anhydrase 12 (CA12)‐Spark and CellMask Deep Red. Because the PM undergoes rapid lipid turnover, we limited the incubation time for the probe and fluorophore to 5 min each at room temperature. Even under these conditions, most of PM‐TCO (**1**) accumulated intracellularly (Figure S2). In contrast, following the click reaction between PM‐Tz (**2**) and cTCO‐QCoum (**28**), the conjugate showed clear and specific localization to the PM (Figure 2A), distinct from the behavior of the click product with TAMRA‐TCO (**29**). We attribute this difference to reduced mobility and enhanced anchoring of the PM‐Tz (**2**)‐cTCO‐QCoum (**28**) click conjugate in the membrane, whereas the PM‐Tz (**2**)‐TAMRA‐TCO (**29**) product might remain highly mobile, diffusing into the cytosol during the incubation. The bulkier cTCO‐QCoum (**28**) likely enhances membrane anchoring of the lipophilic sulfonyl‐based PM‐targeting motif, which alone was insufficient to prevent internalization [45]. We did not directly measure the long‐term stability of this PM‐anchored conjugate; however, its membrane localization persisted for at least one hour. The coumarin‐containing PM‐Coum (**3**) probe demonstrated a distribution similar to that of the PM‐TCO (**1**) probe (Figure S2). PM‐Tz (**2**)‐cTCO‐QCoum (**28**) click product demonstrated good correlation with the controls (mean coefficients PCC_gen_ 0.70, PCC_chem_ 0.77, MCC_gen_ 0.74, MCC_chem_ 0.82, Figure S12A). Collectively, these data demonstrate that the PM‐Tz (**2**)‐cTCO‐QCoum (**28**) click product localizes reliably to the PM.

### 2.3 Mitochondrial Targeting

For mitochondrial targeting, we used the canonical triphenylphosphonium (TPP^+^) moiety, a lipophilic, delocalized cation that accumulates across the negative potential of the inner mitochondrial membrane, driving high mitochondrial uptake of conjugated cargo in a membrane potential‐dependent manner [46, 47, 48]. However, only the TCO‐TPP^+^‐bearing probe, Mito‐TCO (**4**), localized robustly to mitochondria (Figure 2B), showing excellent overlap with both the genetic marker (N‐terminal mitochondrial localization sequence from cytochrome c oxidase 4A fused with Spark protein, COX4A‐Spark) and the membrane‐potential‐dependent dye MitoTracker Deep Red (mean coefficients PCC_gen_ 0.53, PCC_chem_ 0.81, MCC_gen_ 0.74, MCC_chem_ 0.83, Figure S12B). On the other hand, Mito‐Tz (**5**) did not yield substantial fluorescence above background when labeled with either TCO‐containing fluorophore (Figure S3). This loss of signal suggests that the Tz‐containing scaffold in Mito‐Tz (**5**) may attenuate ΔΨ‐driven accumulation or alter cell membrane permeability relative to Mito‐TCO (**4**) [49]. Another possibility is inhibition of the click reaction by steric effects in Mito‐Tz (**5**). We used Mito‐TCO (**4**) at 1 µM to minimize interference with organelle integrity, because we observed mitochondrial destabilization at 10 µM, possibly due to dissipation of mitochondrial membrane potential [50]. For the more cation‐dense and likely better cell‐permeable Mito‐Coum (**6**), an additional decrease to 0.1 µM was required to avoid mitochondrial fragmentation. Overall, Mito‐TCO (**4**) reliably localized to mitochondria.

### 2.4 Nuclear Targeting

Nuclear targeting employed the minor‐groove DNA binder Hoechst 33258, previously utilized to direct cargos into nuclei [51, 52]. Consistent with this mechanism, Nucl‐TCO (**7**) labeled with Coum‐Tz (**27**) produced a distinct nuclear signal that overlapped closely with the genetic marker histone H2B‐Spark and with the far‐red DNA stain DRAQ5 (Figure 2C), indicating both efficient nuclear accumulation and successful IEDDA ligation in the nucleus. Because Hoechst 33258 itself emits in the blue range, we evaluated whether its intrinsic fluorescence contributed to the coumarin emission channel due to spectral overlap. Nucl‐TCO (**7**) alone produced only a weak but detectable signal, whereas the click product showed a markedly stronger emission (Figure S4). The high colocalization coefficients (mean coefficients PCC_gen_ 0.77, PCC_chem_ 0.93, MCC_gen_ 0.93, MCC_chem_ 0.92, Figure S12C) are consistent with a dominant contribution from the nuclear click product rather than from free probe fluorescence.

For the Tz‐bearing Nucl‐Tz (**8**) probe, no detectable intracellular or nuclear accumulation above the background staining was detected (Figure S4). Given that Hoechst‐based probes enter cells through passive diffusion and are sensitive to structural perturbations, this result indicates that the Tz handle either reduced cellular permeability or retention or suppressed Hoechst fluorescence to near‐background levels. Hoechst 33258 is less cell‐permeant than related Hoechst 33342 and is known to exhibit environment‐dependent fluorescence, so additional polarity or steric bulk can readily push cellular uptake below detection [53]. In parallel, tetrazines are widely documented to quench appended fluorophores via through‐bond or dark‐state energy‐transfer mechanisms, and fluorescence is typically restored after IEDDA reaction [54, 55]. Thus, direct tetrazine‐mediated quenching of the Hoechst chromophore remains a plausible contributor to the weak signal of Nucl‐Tz (**8**). While the present data do not distinguish between reduced uptake and quenching, the absence of click‐derived nuclear fluorescence despite incubation with the complementary partner argues that the limiting step is upstream of nuclear ligation, most likely at the level of cell entry or intracellular stability. Overall, we show that only Nucl‐TCO (**7**) reliably traffics to the nucleus and supports efficient IEDDA conjugation there.

### 2.5 ER Targeting

Because the ER lacks a dominant physical driving force (e.g., a membrane potential) or a defined small‐molecule sink, ER targeting by small molecules typically relies on either selective interaction with ER‐resident proteins or preferential partitioning into the ER membrane network [56, 57]. We used several strategies to target the ER. The first employs sulfonamide and sulfonylurea groups, which interact with sulfonylurea receptors within ATP‐sensitive K^+^ channel complexes enriched on the ER membranes, a principle exemplified by glibenclamide‐based ER‐Tracker dyes [58, 59, 60]. Second, highly lipophilic aromatic substituents favor partitioning into the ER lipid bilayer; increasing aromaticity and fluorination (including pentafluorophenyl groups) can bias localization toward the ER [44, 56, 61]. Third, electrophilic chloroalkyl warheads can trap probes in the ER through covalent thioether formation with cysteine residues on the ER proteins, such as a propyl chloride moiety designed to bind the ER‐resident chloride channels [62], a strategy used in some ER‐selective fluorophores and consistent with the high nucleophilicity of protein cysteines toward alkylating groups [56, 63, 64].

Guided by these design principles, we evaluated ER‐targeting probes incorporating a pentafluorophenyl, a chloromethyl, and two sulfonylureas, each bearing either Tz or TCO for IEDDA ligation. Following the click labeling with the complementary fluorophores, three constructs produced an ER‐like reticular staining pattern: ER‐F‐TCO (**9**), ER‐Cl‐TCO (**11**), and ER‐SU1‐Tz (**15**) (Figures 2E–F and S5). Among these, ER‐Cl‐TCO (**11**) and ER‐SU1‐Tz (**15**) gave higher signal‐to‐background ratios, whereas ER‐F‐TCO (**9**) produced a substantial diffuse intracellular background after labeling (Figure S5).

Although sulfonylurea derivatives are established ER‐targeting motifs, their performance in our modular Tz/TCO format was mixed: only one sulfonylurea probe, ER‐SU1‐Tz (**15**), remained functional after introduction of the bioorthogonal handle, and it produced ER‐selective staining only when labeled with TAMRA‐TCO (**29**) (Figures 2F and Figure S5). Appending Tz or TCO might perturb sulfonylurea receptor binding by altering steric bulk or polarity near the pharmacophore, an effect that has been noted generally for ER Tracker‐like ligands, where receptor engagement is sensitive to structural modification [56, 58]. Another possibility is that receptor binding geometrically shields the Tz/TCO handle from productive encounter with the partner reagent, lowering effective click yield in situ; this is consistent with the observation that ER‐SU1‐Coum (**16**) retained an ER staining pattern, while ER‐SU1‐TCO (**14**)‐Coum‐Tz (**27**) and ER‐SU1‐Tz (**15**)‐cTCO‐QCoum (**28**) did not generate clear ER‐restricted click signal (Figure S5).

ER‐SU1‐Tz (**15**) yielded a clear ER signal when labeled with TAMRA‐TCO (**29**), but not with cTCO‐QCoum (**28**). This discrepancy might reflect differences in reaction kinetics and fluorogenic efficiency between these TCO variants. TAMRA‐TCO (**29**) contains a constitutively fluorescent fluorophore, whereas cTCO‐QCoum (**28**) fluorescence depends on efficient tetrazine‐triggered cleavage and dequenching of a coumarin precursor rather than on ligation alone. As a result, any reduction in effective reaction efficiency or suboptimal alignment of the reacting partners will disproportionately suppress signal in the cTCO‐QCoum case. If the quencher is not eliminated and the intermediate becomes trapped in a nonreleasing tautomeric state, the click reaction does not produce fluorogenic activation [65, 66].

Colocalization analysis confirms strong overlap with both the genetic ER marker SEC61B‐Spark and the glibenclamide‐based ER Tracker for ER‐Cl‐TCO (**11**): PCC_gen_ 0.63, PCC_chem_ 0.89, MCC_gen_ 0.97, MCC_chem_ 0.99, and ER‐SU1‐Tz (**15**): PCC_gen_ 0.82, PCC_chem_ 0.89, MCC_gen_ 0.93, MCC_chem_ 0.94, and a weaker overlap for ER‐F‐TCO (**9**): PCC_gen_ 0.43, PCC_chem_ 0.55, MCC_gen_ 0.55, MCC_chem_ 0.85 (Figure S12D).

Overall, these data demonstrate that ER targeting can be achieved in a modular organelle‐targeting group‐Tz/TCO format; however, success is motif‐dependent, and many of our designs were sensitive to the installation of bioorthogonal handles. This organelle‐specific variability aligns with broader probe‐design literature, which emphasizes that ER targeting frequently depends on subtle structure‐property balances and on the compatibility of targeting, reactivity, and cargo modules [56, 57].

### 2.6 Golgi Apparatus Targeting

Golgi localization typically relies on comparatively subtle lipid partitioning or protein recognition effects, and many reported probes show partial ER staining or high cytosolic background [67, 68, 69]. Lipid‐modified peptides and small‐molecule conjugates can associate with Golgi membranes through hydrophobic insertion and trafficking‐dependent retention, but their specificity is often context‐ and scaffold‐dependent [67, 70]. We tested this notion by utilizing myristic acid as a Golgi‐targeting sequence, based on previous studies [44, 71, 72]. As Golgi markers, we used the first 60 amino acids of β‐1,4‐galactosyltransferase (B4GALT1‐Spark) as a genetically encoded marker and BODIPY TR C_5_‐ceramide as the chemical tracker.

In our hands, neither Golgi‐TCO (**19**) nor Golgi‐Tz (**20**) produced Golgi‐restricted labeling: both yielded diffuse intracellular staining with intensities close to background and conspicuous labeling of non‐Golgi structures (Figure S6). Attempts to improve performance by using a pre‐labeled analog Golgi‐Coum (**21**) did not enhance specificity; the probe still showed only partial overlap with Golgi reference markers and exhibited appreciable non‐Golgi signal (Figures 2D and Figure S6). These observations are supported directly by the imaging data and by the quantitative colocalization values for Golgi‐Coum (**21**): PCC_gen_ 0.53, PCC_chem_ 0.88, MCC_gen_ 0.20, MCC_chem_ 0.19 (Figure S12E), where the inflated PCC_chem_ is explained by the substantial background signal of the chemical Golgi tracker itself, correlating with our probes’ diffuse intracellular staining.

Because many lipid‐based Golgi probes require conditions that minimize endocytosis and nonspecific membrane exchange, we tested our probes under established Golgi‐probe incubation protocols, including BSA pre‐complexation and incubation in phosphate‐buffered saline (PBS) at 4°C, which modestly improved localization relative to standard conditions but still led to apparent ER accumulation. These results indicate that myristate‐driven Golgi targeting is not robust across scaffolds and can be overridden by competing lipid‐partitioning or trafficking routes, consistent with reports that Golgi targeting strategies remain less mature than those for mitochondria or lysosomes [67, 73]. In contrast, one successful Golgi targeting approach relies on Cer‐TCO probes that exploit ceramide transport protein (CERT)‐mediated ER‐to‐Golgi trafficking and Golgi‐specific ceramide processing to achieve high specificity [74, 75, 76]. Cysteine‐rich and polycysteine motifs have been used as alternative Golgi anchoring elements, as several Golgi‐resident proteins depend on cysteine residues or cysteine‐rich domains to maintain stable Golgi localization [69, 77].

Taken together, these results indicate that, within our modular Tz/TCO framework, myristate‐based probes are insufficient for reliable Golgi targeting under the tested conditions, highlighting the need for trafficking‐coupled or protein‐binding Golgi motifs to achieve robust compartment specificity.

### 2.7 Lysosomal Targeting

Lysosomal targeting was based on N‐alkylmorpholine (M) and dimethylamine (N) moieties, which become protonated and trapped within the acidic lysosomal environment [78]. These moieties are widely used lysosome‐targeting groups in fluorescent probes and prodrug designs [16, 78]. In our experiments, all lysosomal constructs produced a detectable signal after click labeling (Figure S7), indicating that lysosomal targeting is broadly compatible with both Tz and TCO handles in this series. Among them, Lyso‐M‐Tz (**23**) clicked with the fluorogenic cTCO‐QCoum (**28**) and Lyso‐N‐TCO (**25**) clicked with Coum‐Tz (**27**) demonstrated optimal performance (Figure 2G,H). Colocalization analysis demonstrated good overlap with genetically encoded marker TMEM192‐Spark and chemical LysoTrackers: for Lyso‐M‐Tz (**23**)‐cTCO‐QCoum (**28**) PCC_gen_ 0.68, PCC_chem_ 0.90, MCC_gen_ 0.57, MCC_chem_ 0.84 and for Lyso‐N‐TCO (**25**)‐Coum‐Tz (**27**) PCC_gen_ 0.67, PCC_chem_ 0.78, MCC_gen_ 0.78, MCC_chem_ 0.90 (Figure S12F).

In preliminary multiplexing experiments, we observed that DRAQ5 co‐incubation abolished LysoTracker staining (not shown). DRAQ5 is a membrane‐permeable anthraquinone DNA dye that contains tertiary amine groups (weakly basic substituents) [79, 80]. Because LysoTracker retention depends on lysosomal acidity and protonation, any treatment that partially alkalinizes lysosomes or competes as a weak base, such as incubation with DRAQ5, can diminish its signal.

Our lysosomal Tz probe performed in line with recent organelle‐specific click‐to‐release work that used morpholine‐type lysosome‐targeted tetrazines to enable lysosome‐restricted IEDDA chemistry [81]. Overall, in contrast to the nuclear and ER series, installation of Tz/TCO handles did not measurably compromise lysosomal targeting or labeling efficiency, suggesting that weak base organelle‐targeting groups tolerate modular bioorthogonal modification and remain suitable for compartment‐specific delivery and click‐to‐release applications under acidic organelle conditions.

### 2.8 Overall Probe Performance

Although Tz‐TCO ligations rank among the fastest bioorthogonal reactions in biological media, their performance in cells strongly depends on handle design and probe architecture [22]. Across the full probe panel, constructs bearing TCO were labeled successfully in more cases than their Tz analogs, with the clearest Tz/TCO divergence observed in the nuclear and mitochondrial series (Table 1). This pattern is consistent with the idea that IEDDA performance in cells reflects not only intrinsic reaction kinetics but also how the bioorthogonal handle modulates physicochemical properties and stability of probes.

**TABLE 1:** The performance of organelle‐targeting probes in labeling with the click reaction‐compatible fluorophores and doxorubicin derivatives.

Successful labeling?	Coum‐Tz (**27**)	cTCO‐QCoum (**28**)	TAMRA‐TCO (**29**)	Doxo‐Tz (**31**)	Doxo‐TCO (**32**)	Doxo‐rTz (**33**)	Doxo‐rTCO (**34**)
PM‐TCO (**1**)	No			Yes		No	
PM‐Tz (**2**)		Yes	No		No		
PM‐Coum (**3**)	No^a^						
Mito‐TCO (**4**)	Yes			Yes		Yes	
Mito‐Tz (**5**)		No	No				
Mito‐Coum (**6**)	Yes^a^						
Nucl‐TCO (**7**)	Yes			Yes		Yes	
Nucl‐Tz (**8**)		No	No				
ER‐F‐TCO (**9**)	Yes			Yes		Yes	
ER‐F‐Tz (**10**)		No	No				
ER‐Cl‐TCO (**11**)	Yes			No			
ER‐Cl‐Tz (**12**)		No	No				
ER‐Cl‐Coum (**13**)	No^a^						
ER‐SU1‐TCO (**14**)	No						
ER‐SU1‐Tz (**15**)		No	Yes		No		
ER‐SU1‐Coum (**16**)	Yes^a^						
ER‐SU2‐TCO (**17**)	No						
ER‐SU2‐Tz (**18**)		No	No				
Golgi‐TCO (**19**)	No						
Golgi‐Tz (**20**)		No	No				
Golgi‐Coum (**21**)	No^a^						
Lyso‐M‐TCO (**22**)	Yes			No			
Lyso‐M‐Tz (**23**)		Yes	Yes		Yes		Yes
Lyso‐M‐Coum (**24**)	Yes						
Lyso‐N‐TCO (**25**)	Yes			No			
Lyso‐N‐Coum (**26**)	Yes^a^						

^a^ No click reaction labeling was performed because the compound already contains a fluorophore.

Blank cells indicate combinations that were not tested or that are not applicable.

**FIGURE 3:**
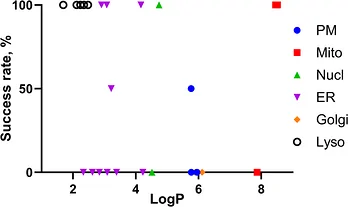
Lipophilicity (LogP) of the organelle‐targeting probes and the observed targeting success rate. LogP values were determined using StarDrop. For Tz‐containing probes that reacted with only one of the two TCO‐containing fluorophores, the success rate was scored 50%.

The calculated logP, logS, and logD values (Table S2) show that TCO‐containing molecules are, in general, more lipophilic than Tz‐modified probes. In principle, greater lipophilicity can increase passive membrane permeation and intracellular accumulation [82]. However, lipophilicity alone cannot explain the observed localization differences because some Tz probes are more lipophilic than other TCO analogs yet still fail to accumulate or label efficiently, indicating that additional determinants dominate in specific scaffolds and organelles. In addition, some probes with high lipophilicity, such as Mito‐TCO (**4**), produced a highly specific staining, in contrast to its Tz‐containing derivative (Figure 3). A second, nonexclusive explanation is the differential stability of Tz versus TCO in living cells. Tetrazine reactivity must be balanced with stability, as electron‐poor, highly reactive Tz can undergo side reactions or degradation in aqueous and cellular environments [83, 84]. Importantly, methyl‐substituted tetrazines are reported to be among the more stable, live‐cell‐compatible variants and have previously generated efficient intracellular labeling [85, 86]. Thus, reduced stability is not expected to be a universal limitation for all tetrazines, but it can remain scaffold‐ and compartment‐dependent.

Our results suggest that even modest architectural changes, such as swapping TCO for Tz within a fixed organelle‐targeting group scaffold, can markedly alter cellular accumulation, distribution, and organelle localization. Nevertheless, we obtained at least one functional probe for each organelle, except the Golgi. We next asked whether our organelle‐targeting group‐Tz/TCO platform can redirect cargos other than fluorophores to specific organelles. Unlike many fluorophores, which often distribute relatively homogeneously without strong bias in cells, drugs and other therapeutics typically exhibit characteristic, mechanism‐driven localization profiles. We therefore used our platform to modify the localization of a clinically‐established therapeutic with intrinsic localization biases and high potency.

### 2.9 Targeted Delivery of Therapeutics to Organelles via Click Chemistry

Organelle‐directed delivery provides the means for concentrating drugs at their subcellular sites of action, which may raise effective potency and lower systemic or off‐target toxicity relative to nontargeted dosing [87, 88, 89]. To test whether our organelle‐targeting group‐Tz/TCO platform can alter drug localization, we selected doxorubicin (Doxo, **30**), a clinically important anthracycline whose intrinsic red fluorescence enables direct tracking in live cells [90, 91]. In its native form, doxorubicin rapidly accumulates in the nucleus due to its interaction with DNA and topoisomerase II, a distribution that is routinely observed by fluorescence microscopy [91, 92] and that we confirmed here (Figure S8, bottom panel).

**FIGURE 4:**
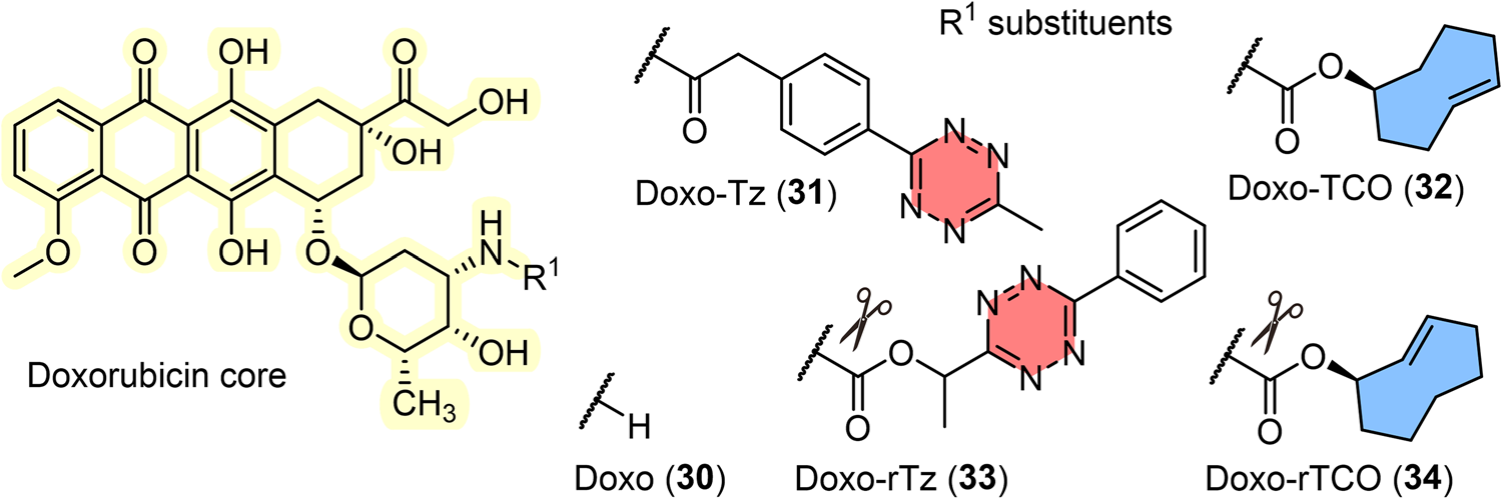
Chemical structures of doxorubicin (Doxo) and its Tz and TCO derivatives. TCO is highlighted in blue, and Tz is highlighted in red. The scissor icons point at the bond cleaved during the click‐to‐release elimination step for the derivatives containing releasable TCO/Tz (rTCO/rTz). Subcellular distribution of the compounds is shown in Figure S8.

We converted doxorubicin into Tz‐ or TCO‐bearing conjugates by derivatizing its primary amine, generating either nonreleasable or click‐to‐release versions (Figure 4). As a result, all Doxo‐Tz/TCO derivatives lost dominant nuclear localization and instead showed a strong ER‐biased distribution with mitochondrial overlap (Figure S8), indicating that prodrug conversion redefines organelle partitioning even before any click reaction occurs. Similar redistribution has been reported for doxorubicin prodrugs and conjugates, including TCO‐linked constructs, where nuclear accumulation is reduced until click‐to‐release or intracellular cleavage restores free doxorubicin [93, 94, 95]. Reports specifically on Doxo‐Tz are comparatively rare relative to Doxo‐TCO [95, 96].

We therefore asked whether organelle‐targeting probes could redirect these ER‐biased Doxo‐Tz/TCO prodrugs toward selected compartments via click chemistry. For imaging‐only experiments, we used the nonreleasable conjugates Doxo‐Tz (**31**) and Doxo‐TCO (**32**) to isolate localization effects from drug release (Figure 4). Because Doxo‐Tz/TCO showed a strong overlap with the ER and mitochondria when incubated with cells at 10 µM, we lowered their concentrations to 1 and 0.5 µM, respectively, which aided the washout of unreacted doxorubicin derivatives. We used a 10 µM concentration for most organelle‐targeting probes, except the mitochondria‐targeting probe, which was used at 1 µM to avoid TPP^+^‐associated mitochondrial perturbation.

**FIGURE 5:**
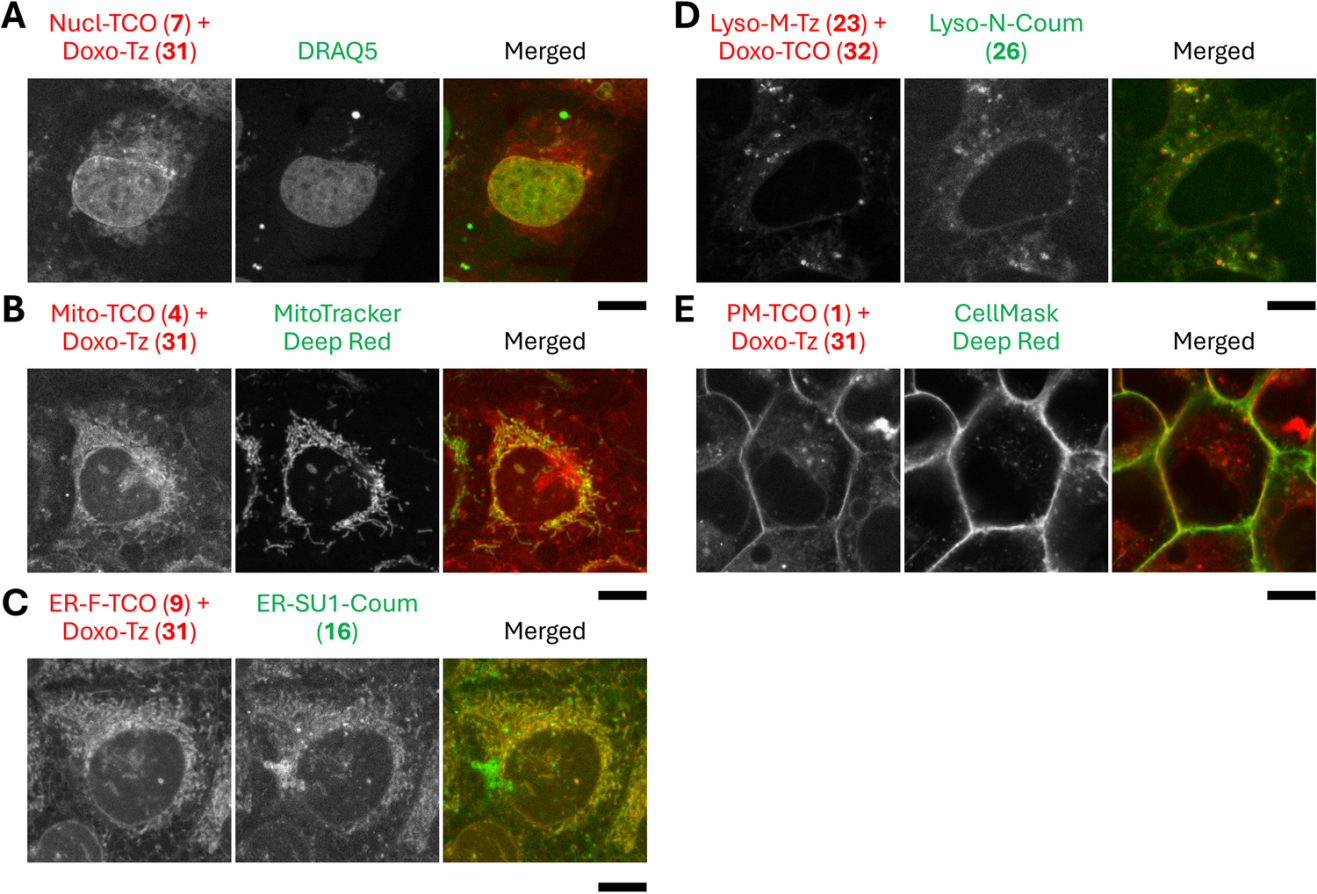
Subcellular localization of the click reaction products between organelle‐targeting probes for the nucleus (A), mitochondria (B), ER (C), lysosomes (D), and PM (E) and nonreleasable doxorubicin derivatives Doxo‐Tz (**31**) used at 1 µM or Doxo‐TCO (**32**) used at 0.5 µM in U2OS cells incubated with corresponding organelle controls. The organelle targeting probes were used at 10 µM, except for the mitochondria‐targeting probe, which was used at 1 µM. Additional microscopy images, including controls, are provided in Figure S9. Scale bar 10 µm.

As a stringent proof of principle, we first tested whether click ligation could restore nuclear localization of doxorubicin prodrugs. Reaction between Nucl‐TCO (**7**) and Doxo‐Tz (**31**) produced a strong signal that largely overlapped with nuclear staining (Figure 5A), whereas Doxo‐Tz (**31**) alone gave minimal nuclear fluorescence under the same conditions (Figure S9), demonstrating that organelle‐anchored click can override intrinsic prodrug routing. We then extended this approach to other organelle‐targeting groups. The most effective targeting was achieved with Mito‐TCO (**4**), PM‐TCO (**1**), ER‐F‐TCO (**9**), and Lyso‐M‐Tz (**23**) (Figure 5B–E), indicating that multiple organelles can serve as capture sites for Doxo conjugates under matched conditions. In general, probe‐TCO + Doxo‐Tz combinations outperformed probe‐Tz + Doxo‐TCO (Figure S9 and Table 1), consistent with two factors supported by our earlier probe screening: TCO‐bearing organelle‐targeting groups showed higher overall targeting fidelity, and tetrazine‐bearing cargos wash out more readily when unreacted, lowering diffuse background.

Notably, several organelle‐targeting probes that performed well in the click reaction with fluorophores were less effective with Doxo‐Tz/TCO cargos. PM‐Tz (**2**) + Doxo‐TCO (**32**) generated a strong intracellular signal, whereas PM‐TCO (**1**) + Doxo‐Tz (**31**) yielded cleaner plasma‐membrane staining, and among ER probes, only ER‐F‐TCO (**9**) reliably retained Doxo‐Tz (**31**) in the ER despite its higher background in the earlier experiments (Figure S9). Even though Doxo‐Tz (**31**) itself shows a tendency to accumulate in the ER, the presence of ER‐F‐TCO (**9**) probe increased the accumulation of doxorubicin in the ER as a result of conjugation with the probe. Likewise, TCO‐based lysosomal organelle‐targeting probes underperformed with Doxo‐Tz (**31**) cargo, while Lyso‐M‐Tz (**23**) demonstrated weaker but still compartment‐distinguishable lysosomal capture (Figures 5D and S9). These shifts directly illustrate that cargo size, steric profile, and polarity can modulate both organelle‐targeting probe retention and click accessibility, so the performance of organelle‐targeting probes with fluorophores cannot always be extrapolated to drug cargos.

### 2.10 Release of Active Therapeutics at Target Organelles

Chemical modifications of doxorubicin can substantially alter its potency, because doxorubicin's primary amine is required for DNA intercalation, topoisomerase II binding, and for the drug's cationic character that promotes nuclear uptake; accordingly, masking of this amine often reduces cytotoxicity and changes its intracellular routing [91, 93, 96]. As shown above, installing Tz/TCO on doxorubicin significantly alters its localization, likely reflecting decreased activity. Adding a second module (an organelle‐targeting group) could plausibly further compromise efficacy by increasing steric bulk and altering charge/hydrophobicity. To overcome this, we used doxorubicin bearing releasable Tz or TCO handles (rTz/rTCO), where IEDDA ligation triggers linker fragmentation and regenerates native doxorubicin (Figure 4).

Click‐to‐release reactions from TCO carbamate/ether linkers have been extensively characterized and shown to restore doxorubicin toxicity relative to the corresponding prodrug [26, 97, 98]. The ligation and release steps often have different kinetic parameters. The initial tetrazine‐TCO IEDDA step is typically extremely fast in biological settings (often 10^3–^10^6^ M^−1^ s^−1^ depending on the pair), while the subsequent elimination that liberates cargo can proceed on slower timescales that depend on the TCO architecture (e.g., allylic substitution, sTCO design) and tetrazine electronics [26, 99]. Prior click‐to‐release platforms exploit this two‐step behavior, using very fast ligation to localize the prodrug and tuned elimination to control the rate of active drug appearance at the target site [97]. In our system, any organelle‐to‐organelle differences in apparent release should therefore be interpreted as the composite of local ligation efficiency and local elimination kinetics, both of which can vary with microenvironment and accessibility.

**FIGURE 6:**
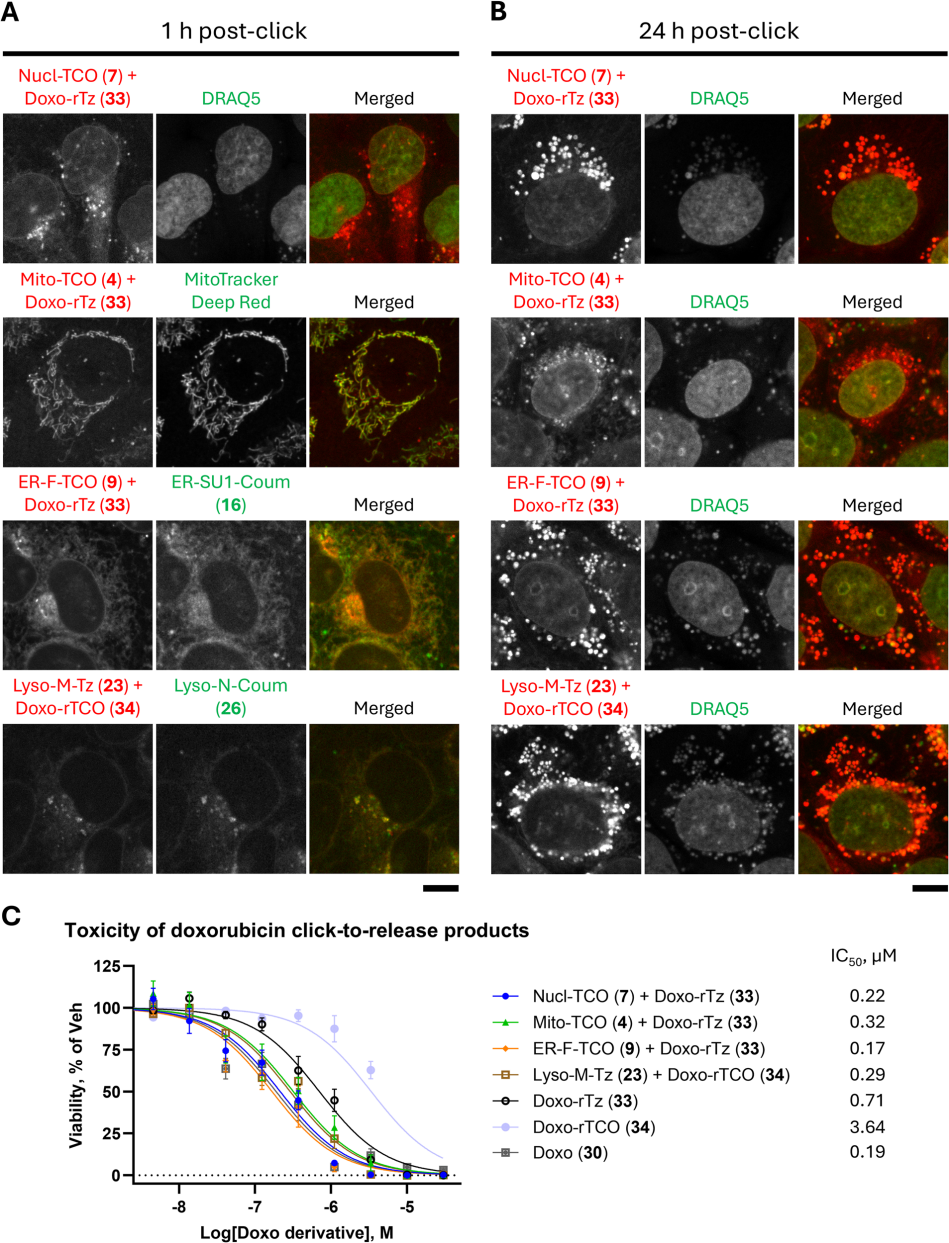
The subcellular localization of doxorubicin conjugates or click‐to‐release products 1 h (A) and 24 h (B) after the click reaction, and the effect of doxorubicin click‐to‐release products on cell viability (C). In the viability assay, the U2OS cells were incubated with the organelle‐targeting probes or vehicle for 1 h, followed by incubation with the corresponding doxorubicin derivative or native doxorubicin. Cell imaging was performed immediately (within 1 h) and 24 h after the click reaction. For toxicity assessment, the incubation period was 72 h under standard cultivation conditions. Cell viability was measured at the end of the incubation using PrestoBlue reagent. Microscopy images and the viability assay of additional click reactions, including controls, are provided in Figures S10, S11, and S13 and Table S3. Data are means ± SEM from four independent experiments. Scale bar 10 µm.

To evaluate whether the release restores the cytotoxic activity of doxorubicin in our system, we incubated cells with organelle‐targeting probes for 1 h (or 15 min in the case of the PM probes), removed excess probe, added corresponding Doxo‐rTz/‐rTCO, incubated the cells for 72 h under standard cultivation conditions, and measured cell viability at the end of the incubation. Several click pairs produced significantly greater cytotoxicity than Doxo‐rTz/‐rTCO alone, matching the cytotoxicity of unmodified doxorubicin within experimental error (p > 0.05 vs. Doxo (**30**), Table S3). The best‐performing probes in this experiment were Nucl‐TCO (**7**), ER‐F‐TCO (**9**), and Lyso‐M‐Tz (**23**) (Figure 6C), indicating that click‐to‐release can restore native‐drug‐like potency when ligation occurs in these compartments. Even though Mito‐TCO (**4**) enabled clear organelle labeling at 1 µM, achieving measurable cytotoxicity upon click‐to‐release required 10 µM of the probe (Figures 6C and S13), consistent with the need for sufficient ligation and release yield to generate an active drug. At 10 µM, the Mito‐TCO (**4**) probe induced mitochondrial fragmentation, a known concentration‐dependent liability of TPP^+^, yet this short exposure did not reduce cell viability under our assay time window (Figure S14; consistent with the literature) [49]. Moreover, incubation of the organelle‐targeting probes alone with the cells under the same conditions did not affect cell viability (Figure S14).

To confirm and visualize our findings from the viability assay, we performed microscopy imaging of the click‐to‐release products at 1, 24, and 48 h after the click reaction. The microscopy results confirmed productive click reactions in the expected organelles at the one hour post‐click (Figure 6A and Table 1). The distribution patterns of these click reaction products resembled those of nonreleasable Doxo‐Tz/‐TCO, but differed in detail, which may result from differences in the rates of click ligation or elimination. For example, the PM‐directed click product showed an increase in intracellular signal, and the nucleus‐directed click showed weaker initial labeling despite preserved toxicity, whereas the mitochondria‐directed probe maintained the same distribution (Figures 6A and S10). Such discrepancies are expected, given that click and release kinetics can be independently sensitive to microenvironment, and click‐to‐release systems in other contexts likewise report faster ligation than payload liberation [26, 97].

At later time points after the click‐to‐release reaction (24 and 48 h), doxorubicin fluorescence emerged in the nucleus, similar to Doxo (**30**) incubation (Figures 6B and S11). In contrast, the controls without the click reaction, Doxo‐rTz (**33**) and Doxo‐rTCO (**34**), did not show any nuclear accumulation during the same incubation periods. This pattern is consistent with click‐triggered regeneration of unmodified doxorubicin, conceptually analogous to tumor‐localized click activation but operating at subcellular resolution [26, 97].

A significant portion of doxorubicin fluorescence originated from particles/aggregates around the nucleus, which overlapped with DRAQ5 nuclear staining. These particles may result from DNA fragmentation due to doxorubicin activity below acute toxicity levels, allowing cells to preserve their nuclei through active repair processes. These particles were not detected in cells treated with Doxo‐rTz/‐rTCO or vehicle alone (Figure S11).

Even though we successfully achieved the release of native doxorubicin at the organelles, doxorubicin escaped the targeted organelle and redistributed according to its intrinsic preference for the nucleus. Additional design refinements may be needed to retain activity locally. In future iterations, this could include slower‐releasing linkers to bias activity toward the release compartment, or minimally perturbing retention motifs added to the liberated drug to reduce efflux without compromising potency [26, 99]. Tuning charge, polarity, and hydrogen‐bonding capacity may help reduce membrane permeability and limit redistribution of the released drug, whereas macromolecular or polymeric scaffolds could further constrain diffusion and promote local retention [100, 101]. Another direction is to apply the same organelle‐localized click‐to‐release logic to fast‐acting or locally reactive cargos whose effects occur before diffusion takes place, thereby converting spatially‐restricted release into spatially‐restricted function [97].

Overall, these experiments demonstrate that organelle‐resident probes can retarget bioorthogonally‐modified doxorubicin away from its ER‐biased prodrug distribution and toward selected compartments, and they highlight steric and physicochemical constraints that will need to be optimized for future click chemistry‐based release and activation of therapeutics [26, 94, 95].

### 2.11 Limitations of the Study

First, although Tz‐TCO chemistry enables rapid and selective ligation in living cells, the performance of individual probes was strongly context‐dependent. Small structural changes, including Tz‐to‐TCO exchange or variation of the attached cargo, frequently altered probe performance. Therefore, the conclusions cannot be directly extrapolated to other probes, even when the same targeting motif is used. Second, although click‐to‐release chemistry successfully restored doxorubicin cytotoxicity, the released native drug redistributed according to its intrinsic physicochemical properties and ultimately accumulated in the nucleus. This behavior illustrates that subcellular click‐to‐release enables spatially localized activation but, by itself, does not ensure long‐term retention of freely diffusible drugs within the release compartment. Third, all experiments were performed in cultured cell lines under defined conditions. Differences in membrane composition, organelle physiology, metabolic state, or expression of transport and binding proteins across cell types may substantially influence probe behavior. Therefore, the generality of the observed targeting and release patterns across primary cells, tissues, or in vivo systems remains to be established. Last, while this study establishes a comparative framework for organelle‐directed Tz‐TCO chemistry, it does not exhaust the chemical space of targeting motifs, linkers, or reactive handles. Some organelles, particularly the Golgi apparatus, remain difficult to target reliably with small molecules, and improved delivery strategies may require trafficking‐based or protein‐binding approaches not explored here.

## 3 Conclusions

In this work, we present a systematic evaluation of Tz‐TCO click chemistry for directing small molecules and therapeutic cargos to defined cellular organelles. By combining a diverse set of organelle‐targeting groups with Tz or TCO handles, we demonstrate localization of the resulting probes to the PM, mitochondria, nucleus, ER, and lysosomes, but with strongly organelle‐ and scaffold‐dependent outcomes. We found that the bioorthogonal handle itself is not a neutral appendage. Exchanging TCO and Tz within otherwise identical probes frequently altered organelle accumulation and effective labeling. TCO‐containing probes were generally more robust across organelles, whereas Tz‐containing probes were more sensitive to structural context and the intracellular environment. These observations underscore that organelle‐targeting performance cannot be predicted solely from the targeting motif and must be evaluated in the final, fully assembled construct.

By extending this platform to a clinically relevant drug, doxorubicin, we show that organelle‐localized click reactions can override the intrinsic intracellular distribution of a modified therapeutic. Moreover, using releasable TCO or Tz linkers, we demonstrate that bioorthogonal ligation can trigger the regeneration of active doxorubicin within cells, restoring cytotoxicity to levels comparable to those of the unmodified drug. However, our results indicate that released doxorubicin ultimately redistributes according to its native physicochemical properties, emphasizing that localized activation does not guarantee localized action for freely diffusible drugs. Therefore, additional chemical design strategies may be needed to retain small‐molecule drugs at the release site. These designs might include tuning prodrug properties to reduce membrane permeability of activated drugs, generating minimally‐perturbing retention motifs upon release, using slower‐releasing bioorthogonal handles to prevent accumulation of released drugs at their preferential locations, or designing fast‐acting or locally‐reactive drugs with their effects occurring before diffusion, for example, molecules generating short‐lived reactive species.

## Author Contributions

**Oleh Durydivka**: methodology, investigation, validation, formal analysis, visualization, writing – original draft, writing – review & editing. **Marek Chovanec**: methodology, investigation, validation, formal analysis, visualization, writing – review & editing. **Rastislav Dzijak**: conceptualization, methodology, resources. **Michal Rahm**: investigation, validation. **Jachym Hrusak**: investigation, validation. **Paul E. Reyes‐Gutierrez**: investigation, validation. **Milan Vrabel**: conceptualization, formal analysis, supervision, funding acquisition, project administration, writing – review & editing.

## Conflicts of Interest

The authors declare no conflicts of interest.

## Supporting information




**Supporting File 1**: chem71198‐sup‐0001‐SI‐Figures.pdf. Figures S1–14. Microscopy images of click labeling of organelles, including all controls, colocalization values, and additional conditions and controls for cell viability experiments.

**Supporting File 2**: chem71198‐sup‐0002‐SI‐Tables.pdf. Tables S1‐3. The overview of targeting groups, commercial trackers, and genetic markers used, calculated logP, logS, and logD values of the probes, and the potencies of doxorubicin click‐to‐release products in a viability assay.

**Supporting File 3**: chem71198‐sup‐0003‐SI‐Experimental.pdf. Experimental procedures and NMR spectra. Experimental procedures for cell handling and cell line generation, cell labeling, imaging, and viability assay, as well as synthetic procedures and characterization data of all new compounds.
The authors have cited additional references within the Supporting Information [102, 103, 104, 105, 106, 107, 108, 109, 110, 111, 112, 113, 114, 115, 116, 117, 118, 119].

## Data Availability

The data that support the findings of this study are available in the main text and the supporting information of this article.
