# Supplementary material for: Organelle‐Resolved Tetrazine‐trans‐Cyclooctene Click Chemistry for Cargo Delivery and Release
Source: Chemistry. 2026 Jun 1;32(35):e71198. doi: 10.1002/chem.71198 (PMC13587637; doi:10.1002/chem.71198)
Supplement: Supplementary file 1 [file CHEM-32-e71198-s001.pdf]

Organelle-Resolved Tetrazine-*trans*-Cyclooctene Click Chemistry for Cargo Delivery and Release

Oleh Durydivka, Marek Chovanec, Rastislav Dzijak, Michal Rahm, Jachym Hrusak, Paul E. Reyes-Gutierrez, and Milan Vrabel*

Institute of Organic Chemistry and Biochemistry of the Czech Academy of Sciences, Flemingovo nám. 2, 160 00, Prague, Czech Republic

*Correspondence: vrabel@uochb.cas.cz

Experimental procedures and NMR spectra

CONTENT

1. Experimental procedures (biology) ... 3
   1.1 Plasmids and lentiviral particle production ... 3
   1.2 Cell culture and stable cell line generation ... 3
   1.3 Cell labeling ... 4
   1.4 Microscopy and image analysis ... 6
   1.5 Cell viability assay ... 6
2. Experimental procedures (chemistry) ... 8
   2.1 General information ... 8
   2.2 Synthesis of intermediates ... 8
      3-(dodecylamino)propane-1-sulfonic acid (35) ... 8
      Hoechst 33258 O-ethyl-Boc-carbamate (36) ... 9
      Hoechst 33258 *O*-ethylamine (37) ... 9
      Tert-butyl (2-(perfluorobenzamido)ethyl)carbamate (38) ... 10
      2-(perfluorobenzamido)ethan-1-aminium 2,2,2-trifluoroacetate (39) ... 10
      tert-butyl (4-(N-(cyclohexylcarbamoyl)sulfamoyl)phenethyl)carbamate (40) ... 11
      4-(2-aminoethyl)-N-(cyclohexylcarbamoyl)benzenesulfonamide, 2,2,2-trifluoroacetate salt (41) ... 11
      N-(2-aminoethyl)-4-methylbenzenesulfonamide (42) ... 12
      2-tetradecanamidoethan-1-aminium 2,2,2-trifluoroacetate (43) ... 12
      2-((tetrahydro-2H-pyran-2-yl)oxy)propanenitrile (44) ... 13
      1-(6-phenyl-1,2,4,5-tetrazin-3-yl)ethan-1-ol (45) ... 13
      2,5-dioxopyrrolidin-1-yl (1-(6-phenyl-1,2,4,5-tetrazin-3-yl)ethyl) carbonate (46) ... 14
   2.3 Synthesis of the organelle-targeting TCO probes ... 15
      PM-TCO (1) ... 15
      Mito-TCO (4) ... 16
      Nucl-TCO (7) ... 16
      ER-F-TCO (9) ... 17

ER-Cl-TCO (11) 18
ER-SU1-TCO (14) 18
ER-SU2-TCO (17) 19
Golgi-TCO (19) 19
Lyso-M-TCO (22) 20
Lyso-N-TCO (25) 20
2.4 Synthesis of the organelle-targeting Tz probes 21
PM-Tz (2) 21
Mito-Tz (5) 22
Nucl-Tz (8) 23
ER-F-Tz (10) 23
ER-Cl-Tz (12) 24
ER-SU1-Tz (15) 24
ER-SU2-Tz (18) 25
Golgi-Tz (20) 25
Lyso-M-Tz (23) 26
2.5 Synthesis of the organelle-targeting coumarin probes 27
PM-Coum (3) 27
Mito-Coum (6) 28
ER-Cl-Coum (13) 29
ER-SU1-Coum (16) 29
Golgi-Coum (21) 30
Lyso-M-Coum (24) 30
Lyso-N-Coum (26) 31
2.6 Synthesis of the labeling fluorophores 32
Coum-Tz (27) 32
cTCO-QCoum (28) 32
TAMRA-TCO (29) 32
2.7 Synthesis of the doxorubicin caged compounds 33
Doxo-Tz (31) 33
Doxo-TCO (32) 34
Doxo-rTz (33) 35
Doxo-rTCO (34) 36
3. REFERENCES 37
4. NMR Spectra 39

1. EXPERIMENTAL PROCEDURES (BIOLOGY)

1.1 Plasmids and lentiviral particle production

Coding sequences of organelle marker proteins or targeting sequences were amplified from original plasmids to create C-terminal Spark fusions (except for the ER-targeting SEC61B, which had an N-terminal Spark fusion) in the lentiviral expression plasmids. For the plasma membrane-Spark targeting plasmid, we used the coding sequence from the human carbonic anhydrase 12 (CAXII/CA12) ORF mammalian expression plasmid, C-GFPSpark tag (Sino Biological, China).[1] For targeting Spark to mitochondria, we introduced the N-terminal mitochondrial localization sequence from cytochrome c oxidase 4A (COX4A/COX4-1) into the Spark coding sequence by amplification from mito-V5-APEX2 plasmid, a gift from Alice Ting (Addgene plasmid # 72480; http://n2t.net/addgene:72480; RRID: Addgene_72480).[2] For the nuclear-targeting plasmid construction, the template was the human histone H2B-coding plasmid (a gift from Dr. Plevka, CEITEC, Czech Republic).[3] For the ER targeting, we amplified SEC61B from the human GFP-SEC61B plasmid, a gift from Christine Mayr (Addgene plasmid # 121159; http://n2t.net/addgene:121159; RRID: Addgene_121159).[4] For the Golgi targeting, we used the human B4GALT1 ORF mammalian expression plasmid (Promega, US).[5] For lysosomal-targeting plasmid generation, we used the transmembrane protein 192 (TMEM192) coding plasmid, a gift from Dr. Sabatini, IOCB Prague, Czech Republic.[6-7]

The coding sequences of organelle markers, together with Spark fluorescent protein, were inserted into the pCDH-CMV-MCS-EF1α-Puro vector (SBI Systems Biosciences, US). The obtained plasmids were then used for the preparation of lentiviral particles. For this, HEK293T cells (ATCC CRL-3216, RRID: CVCL_0063) growing in a 10 cm dish were transfected with the pMD2G, psPAX2, and the corresponding lentiviral transfer vector (4, 7, and 9 μg of DNA, respectively) using Lipofectamine 3000 transfection reagent (ThermoFisher, US). Twenty-four hours post-transfection, the media were replaced with DMEM containing 20% FBS, and the cells were cultured for an additional 72 hours. Media containing the lentiviral particles were collected and filtered using a 0.45 μm syringe filter (Millipore, Germany).

1.2 Cell culture and stable cell line generation

Human osteosarcoma U2OS cells (ATCC HTB-96, RRID: CVCL_0042, authenticated by Generi Biotech, Czech Republic) were cultured in high-glucose Dulbecco's Modified Eagle Medium (DMEM, Biowest, France) containing 10% fetal bovine serum (FBS, Gibco, US) at 37 °C and 5% CO_2 in air in a humidified incubator. To generate stable cell lines expressing organelle markers, the cells were transduced with lentiviral particles.

For viral transduction, U2OS cells were seeded into a 6-well plate (TPP, Switzerland) at 500 000 per well. After 24 hours of cultivation, the media were aspirated and replaced with 1 mL of fresh DMEM containing 10% FBS. Then, the cells were transduced with 500 μL of the filtered supernatant containing the viral particles. Two days later, the media were replaced with 2 mL of fresh DMEM containing 10% FBS and 2 μg/mL of puromycin (Sigma-Aldrich, Germany). The cells were then selected for 2 days. The stable integrated cell population was enriched by sorting against Spark protein fluorescence, and the cells were routinely cultured in the presence of 2 μg/mL puromycin.

1.3 Cell labeling

Cell labeling and imaging were performed in live cells. Generally, after an initial wash, cells were incubated with either a TCO- or Tz-containing probe at 10 µM for 30 min at 37 °C. After a second wash, the corresponding labeling reagent (Coum-Tz (**27**) for TCO-probes, cTCO-QCoum (**28**) or TAMRA-TCO (**29**) for Tz-probes) was applied at 10 µM and incubated for 30 min at 37 °C. In the case of TAMRA-TCO (**29**) labeling, an additional washing step was performed prior to imaging to remove excess unreacted reagent. When labeled with Coum-Tz (**27**) or cTCO-QCoum (**28**), no wash was required before imaging due to their fluorogenicity (Suppl. Figure 1). We also collected images of the localization of the fluorophore-TCO/-Tz without incubation with the organelle-targeting probes to assess probe specificity and background labeling of the fluorophores.

One day before imaging, the cells were plated into wells of a 96-well black plate with a clear bottom (Cellvis, US) at 25 000 cells per well in the complete cultivation medium without antibiotics. After 24 h incubation, the plates were washed once with 1x phosphate-buffered saline (PBS, Biowest, France). The live cells during labeling were incubated generally in serum-free DMEM, and after the labeling, were kept in the cell imaging medium: serum-free phenol red-free high-glucose DMEM containing 25 mM HEPES (Gibco, US). When TAMRA-TCO (**29**) was used for click labeling or a coumarin-containing compound was used, the wells containing cells were washed before switching to cell imaging medium. When Coum-Tz (**27**) and cTCO-QCoum (**28**) were used, the incubation was performed in cell imaging medium, and no further washing steps were done. The specific labeling conditions for each organelle are provided in the table below:

**Organelle**	**First incubation (with probe)**	**Wash, once/or with**	**Second incubation (with labeling fluorophore) in**	**Wash, once/ with (for TAMRA- or -Coum)**
**Plasma membrane**	Cell imaging medium, 5 min, room temperature (RT)	-	Cell imaging medium, 5 min, RT	Cell imaging medium
**Mitochondria**	DMEM, 30 min, 37 °C	DMEM	DMEM or Cell imaging medium, 30 min, 37 °C	DMEM
**Nucleus**	DMEM, 30 min, 37 °C	DMEM	DMEM or Cell imaging medium, 30 min, 37 °C	DMEM
**Endoplasmic reticulum**	HBSS, 30 min, 37 °C	DMEM	DMEM or Cell imaging medium, 30 min, 37 °C	DMEM
**Golgi apparatus**	PBS, 30 min, 4 °C	Twice, DMEM + 10% FBS, at 4 °C	Preincubation without labeling fluorophores in DMEM + 10% FBS, 30 min at 37 °C, and two washes with DMEM + 10% FBS. Then, incubation with labeling fluorophores in DMEM + 10% FBS or Cell imaging medium + 10% FBS, 30 min, 37 °C	DMEM + 10% FBS

**Lysosome**	DMEM + 10% FBS, 30 min, 37 °C	DMEM + 10% FBS	DMEM + 10% FBS or Cell imaging medium + 10% FBS, 30 min, 37 °C	Thrice, DMEM + 10% FBS

The organelle-targeting probes were used at a final concentration of 10 µM, except for probes targeting mitochondria: Mito-TCO (**4**), Mito-Tz (**5**) – at 1 µM, and Mito-Coum (**6**) – at 0.1 µM. The labeling fluorophores were used at 10 µM. Probes targeting the Golgi apparatus were preabsorbed with 10 µM bovine serum albumin (BSA) for 30 min at 37 °C. The organelle trackers were incubated simultaneously with organelle-targeting probes in non-transfected U2OS cells. All organelle trackers were from Invitrogen, US, except for DRAQ5 Fluorescent Probe, which was from Thermo Scientific, US. The concentrations of the trackers are provided in the table below:

**Cell compartment**	**Chemical tracker**	**Working concentration**
Plasma membrane	CellMask Deep Red Plasma Membrane Stain	0.5 µg/mL
Mitochondria	MitoTracker Deep Red FM	25 nM
Nucleus	DRAQ5 Fluorescent Probe	1 µM
Endoplasmic reticulum	BODIPY FL glibenclamide<br>BODIPY TR glibenclamide	1 µM<br>1 µM
Golgi apparatus	BODIPY FL C_5-ceramide<br>BODIPY TR C_5-ceramide	5 µM<br>5 µM
Lysosome	LysoTracker Green DND-26<br>LysoTracker Red DND-99	75 nM<br>50 nM

Doxorubicin and its derivatives were used at 10 µM in simple incubations (as in Suppl. Figure 8) or usually at 1 µM in experiments involving the click reaction. The incubations were performed in DMEM (without FBS), and the compounds were coincubated with organelle chemical trackers used at the same concentrations as above, or 5 µM ER-SU1-Coum (**16**) or Lyso-N-Coum (**26**), to avoid spectral overlap of the red-emitting trackers with doxorubicin emission. After incubation, the wells were washed once with DMEM before switching to cell imaging medium. The imaging was performed immediately in live cells at room temperature.

For the click reaction between organelle-targeting probes and doxorubicin derivatives, we used the same conditions as described above for the probe labeling and 1 µM for Doxo-Tz/TCO incubations for 1 h, with some exceptions: the corresponding PM probes were used with either 1 µM Doxo-Tz (**31**) for 10 min or 0.5 µM Doxo-TCO (**32**) for 5 min, Lyso-M-Tz (**23**) probe was used with 0.5 µM Doxo-TCO (**32**) for 1 h.

For the click-to-release reaction visualization by microscopy, we used 0.2 µM Doxo-rTz for the PM and Mito probes, and 0.5 µM Doxo-rTz/-rTCO for the Nucl, ER, and Lyso probes. The imaging was performed either immediately (for this, appropriate organelle trackers were coincubated with the probes as above), or the cells were incubated for 24 or 48 hours under normal growth conditions (in this case, the cells

were stained only with DRAQ5 before the imaging). The imaging was performed in live cells incubated in cell imaging medium at room temperature.

1.4 Microscopy and image analysis

Living cells after the labeling were imaged at room temperature without CO_2 supplementation under the Leica SP8 Falcon confocal microscope (Leica Microsystems, Germany) using HC PL APO CS2 63x/1.20 water immersion objective, 2x digital zoom, 1.5 AU (167.1 µm) pinhole, 1120 x 1120 resolution, resulting in a pixel size of 80 nm. For excitation, the lasers were: 405 nm diode laser, 488 nm Ar laser, 561 nm DPSS laser, and 633 nm HeNe laser. Fluorescence emission was detected using a Leica HyD SMD detector in 8-bit mode and with a smart gain set to 10. We used the following detection windows based on the fluorophore: blue channel: coumarin (410 - 483 nm); green channel: Spark/BODIPY FL/DND-26 (493 - 556 nm); red channel: TAMRA/BODIPY TR/DND-99 (566 - 628 nm), doxorubicin (525 - 628 nm); far red channel: Deep Red FM/ DRAQ5 Fluorescent Probe/ CellMask Deep Red (638 - 700 nm). Each channel was recorded in sequential mode. The microscopy images were recorded within 1.5 h after the click labeling. The images were processed in ImageJ 1.54p (NIH, US). Image modifications, such as background and contrast changes, were applied to the entire image. All processing parameters were the same for the experimental click ligations and their controls. 2D histograms were created using the BIOP-JACoP plugin, and intensity profiles were extracted from ImageJ and plotted in GraphPad Prism. Pearson's and Manders' colocalization coefficients were determined using the JACoP plugin,[8] using manual thresholding, and using five different fields of view per condition to calculate the colocalization coefficients: PCC for colocalization with genetically-encoded markers (PCC_{gen}) and chemical trackers (PCC_{chem}), and, similarly, M1 Manders' correlation coefficients (click ligation vs. control) for colocalization with genetically-encoded and chemical trackers (MCC_{gen}, MCC_{chem}, respectively). Colocalization coefficients were summarized as means ± SEM.

For each organelle, we employed two types of references: genetically encoded protein markers and commercially available chemical trackers (Suppl. Table 1). Genetic markers offer a higher signal-to-noise ratio, stable expression, and defined targeting; however, they require transfection or generation of a stable cell line and overexpression of the organelle-targeting sequence or the entire protein, which may alter organelle morphology or function. Chemical trackers are fast to apply and do not require genetic manipulation, but their localization may depend on cell health, organelle state, or loading efficiency, and they may delocalize under stress or altered cellular conditions. Moreover, chemical trackers may decrease the performance of our probes due to competition for the same environment.[9-11] These two types of organelle references therefore provide complementary advantages and mitigate each other's limitations.

1.5 Cell viability assay

U2OS cells were plated in wells of a 96-well transparent plate (TPP, Switzerland) at 12 000 cells per well, precoated with poly-D-lysine (Gibco, US) (0.01% solution in 1x PBS incubated for 1 h at 37 °C and removed afterward), and the assay was performed the next day. The conditions were adjusted, and washing steps were minimized, and careful aspiration was used to reduce cell disturbance and detachment. First, the cells were incubated in DMEM + 20 mM HEPES containing 10 or 1 µM of organelle-targeting probes (except for the ER probe, which was incubated in HBSS at 10 µM) or vehicle (0.1% DMSO). The probes were incubated with the cells for 1 h (except for the PM probe - 15 min). Then, the

medium was changed to vehicle (0.5% DMSO) or to increasing concentrations of Doxo-rTz/-rTCO or Doxo, using 1:3 serial dilutions resulting in 10 concentrations, ranging from 1.52 nM to 30 μM, in complete DMEM + 20 mM HEPES. The cells were incubated with doxorubicin or its derivatives for 72 h under normal growth conditions without changing the medium. After that, the medium was replaced with the cell imaging medium containing 1:10 (v/v) PrestoBlue reagent (Invitrogen, US) and incubated for 30 min at 37 °C. After the incubation, the fluorescence was read using Tecan Spark reader (Tecan, Switzerland) with the following parameters: excitation: 560 ± 5 nm, emission: 590 ± 5 nm, bottom reading mode. The blank (cell imaging medium containing PrestoBlue reagent in a well without cells) was subtracted from all readings. The assay was performed in four independent experiments, each with two technical replicates. The viability data were normalized to the vehicle control (100%). The concentration-response data were visualized and analyzed in GraphPad Prism 10.6.1 for Windows 64-bit (GraphPad Software, US) using a four-parameter nonlinear regression (log(inhibitor) vs. response) to determine the potency (logIC_{50}, IC_{50}) and standard error of the mean (SEM) of the treatments. The bottom and top were constrained to 0 and 100, respectively. To express IC_{50} values as a percentage of Doxo (**30**) and to determine their 95% confidence intervals, we used the EC50 shift nonlinear regression function in GraphPad Prism.

2. EXPERIMENTAL PROCEDURES (CHEMISTRY)

2.1 General information

All chemicals were obtained from commercial suppliers and were used without further purification. Reactions with air-and moisture-sensitive reactants were performed in anhydrous solvents under argon atmosphere. Solutions were concentrated using a rotary evaporator *Heidolph* equipped with a PC3001 VARIOpro pump from *Vacuubrand*. Column chromatography was carried out on silica gel 60A (particle size: 40-60 µm) from *Acros Organics*. Mixtures of solvents are each stated as volume fractions. For flash column chromatography, a Combi*Flash*® Rf+ from *Teledyne ISCO* or puri*Flash*® 5.250 from *Interchim* was used. Preparative HPLC purification was performed by 1200 Infinity HPLC System from *Agilent Technologies*. Thin-layer chromatography was performed on aluminum sheets from *Merck* (silica gel 60 F254, 20 × 20 cm). Chromatograms were visualized by UV light (λ = 254 or 366 nm). ^{1}H- and ^{13}C-NMR spectra were measured at rt (20 °C) on a Bruker Avance III™ HD 400 MHz NMR system equipped with Prodigy cryo-probe or on a Bruker Avance III™ 500 MHz NMR spectrometer (^{1}H at 500.0 MHz, ^{13}C at 125.7 MHz) and Bruker Avance III™ 600 MHz NMR spectrometer (^{1}H at 600.1 MHz, ^{13}C at 150.9 MHz). Chemical shifts δ are quoted in ppm in relation to the chemical shift of the residual non-deuterated solvent peak ($CDCl_3$: δ (^{1}H) = 7.26, δ(^{13}C) = 77.16, C_6D_6: δ (^{1}H) = 7.16, δ (^{13}C) = 128.06, CD_3OD: δ (^{1}H) = 3.31, δ (^{13}C) = 49.00, DMSO-d_6: δ (^{1}H) = 2.50, δ (^{13}C) = 39.52, CD_3CN: δ (^{1}H) = 1.94, δ (^{13}C) = 1.32 or 118.26, acetone-d_6: δ (^{1}H) = 2.05, δ (^{13}C) = 29.84 or 206.26. The structures were generally assigned based on 2D NMR experiments (COSY, HSQC, HMBC). High-resolution mass spectra were recorded on an *Agilent* 5975C MSD Quadrupol or LTQ Orbitrap XL from *Thermo Fisher Scientific*. The reaction progress was followed by HPLC-MS measurements on an LCMS-2020 system from *Shimadzu* equipped with a CORTECS C18 column (2.7 µm, 50 × 4.6 mm) or Luna C18 column (3 µm, 10 × 4.6 mm). Gradient of MeCN + 0.05% HCOOH in water + 0.05% HCOOH was used for the analysis. LogP, logS, and logD values were calculated in StarDrop 8.0.0.9512.

2.2 Synthesis of intermediates

3-(dodecylamino)propane-1-sulfonic acid (35)

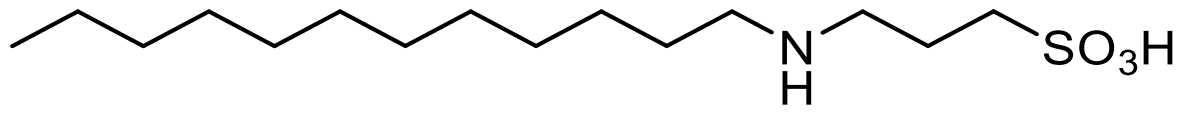

This compound was synthesised according to the published protocol.[12-13]

Dodecylamine (1.0 g, 5.40 mmol, 1 equiv.) and **propane sultone** (568 µL, 6.48 mmol, 1.2 equiv.) were dissolved in **acetone** (36 mL) and at reflux stirred for 10 h. After cooling to RT, the reaction mixture was filtered and washed 3× with acetone to obtain **3-(dodecylamino)propane-1-sulfonic acid (35)** as a white solid (692 mg, 42%).

The measured ^{1}H NMR spectrum corresponds to the published spectra.[13]

^{1}H NMR (400 MHz, DMSO-d_6): δ = 8.47 (bs, 2H), 3.03 (t, J = 6.8 Hz, 2H), 2.85 (t, J = 7.6 Hz, 2H), 2.61 (t, J = 6.6 Hz, 2H), 1.92 (p, J = 6.8 Hz, 2H), 1.59–1.48 (m, 2H), 1.34–1.17 (m, 19H), 0.86 (t, 3H).

Hoechst 33258 O-ethyl-Boc-carbamate (36)

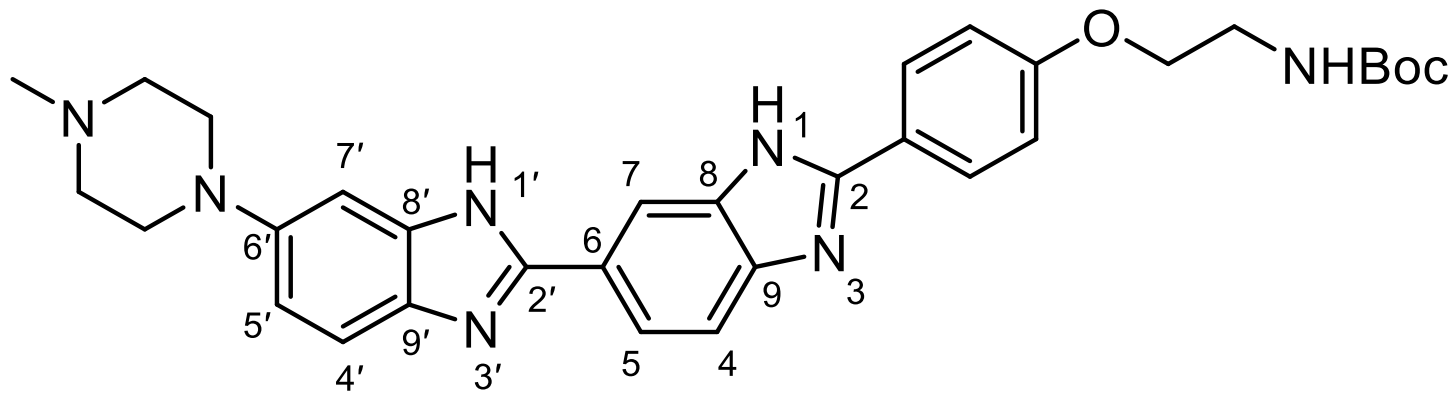

Into a stirred solution of **Hoechst 33258 trihydrochloride** (150 mg, 0.28 mmol, 1 equiv.) in dry **DMF** (2.5 mL), ***tert*-butyl *N*-(2-bromoethyl)carbamate** (125 mg, 0.64 mmol, 2.3 equiv.) in dry **DMF** (1 mL) and **K_2CO_3** (234 mg, 1.69 mmol, 6 equiv.) were added. The reaction mixture was stirred at 50 °C for 72 h. HPLC-MS analysis showed satisfactory conversion. The solvents were evaporated under reduced pressure, and the residue was purified by reversed-phase chromatography (C18 column, eluent: MeCN/H_2O), followed by lyophilisation to obtain **Hoechst 33258 *O*-ethyl-Boc-carbamate (36)** (47 mg, 30%) as a yellow solid.

^{1}H NMR (400 MHz, CD_3OD): δ = 8.20 (d, J = 1.7 Hz, 1H, **H**-7), 8.03–7.97 (m, 2H, C**H**CHCOCH$_2$), 7.90 (dd, J = 8.5, 1.7 Hz, 1H, **H**-5), 7.66–7.62 (m, 1H, H-4), 7.49 (d, J = 8.8 Hz, 1H, **H**-4'), 7.12 (d, J = 2.2 Hz, 1H, **H**-7'), 7.08–6.99 (m, 3H, **H**-5', CHC**H**COCH$_2$), 4.04 (t, J = 5.6 Hz, 2H, OC**H$_2$**CH$_2$NH), 3.44 (t, J = 5.6 Hz, 2H, OCH$_2$C**H$_2$**NH), 3.30–3.28 (m, 4H, CH$_3$N(CH$_2$C**H$_2$**)$_2$N), 3.03 (t, J = 5.0 Hz, 4H, CH$_3$N(C**H$_2$**CH$_2$)$_2$N), 2.64 (s, 3H, C**H$_3$**N), 1.45 (s, 9H, (C**H$_3$**)$_3$C). **^{13}C NMR** (101 MHz, CD_3OD): δ = 162.3 (C$_{ipso}$(CHCH)$_2$**C**$_{ipso}$O), 158.5 (NH**C**OO), 155.3 (**C**-2), 153.9 (**C**-2'), 148.9 (**C**-6'), 141.9 (**C**-9), 140.4 (**C**-8, **C**-8'), 135.9 (**C**-9'), 129.6 (C$_{ipso}$(**C**HCH)$_2$C$_{ipso}$O), 125.4 (**C**-6), 123.1 (**C**$_{ipso}$(CHCH)$_2$C$_{ipso}$O), 122.5 (**C**-5), 116.5 (**C**-4', **C**-5'), 116.1 (C-4, C$_{ipso}$(CH**C**H)$_2$C$_{ipso}$O), 113.9 (**C**-7), 102.8 (**C**-7'), 80.3 ((CH$_3$)$_3$**C**), 68.1 (O**C**H$_2$CH$_2$NH), 55.6 (CH$_3$N(**C**H$_2$CH$_2$)$_2$N), 50.8 (CH$_3$N(CH$_2$**C**H$_2$)$_2$N), 44.9 (**C**H$_3$N), 40.9 (OCH$_2$**C**H$_2$NH), 28.8 ((**C**H$_3$)$_3$C). **HRMS** (ESI+): calculated for $C_{32}H_{38}O_3N_7^+$ ([M+H]$^+$) 568.3031, found 568.3030.

Hoechst 33258 *O*-ethylamine (37)

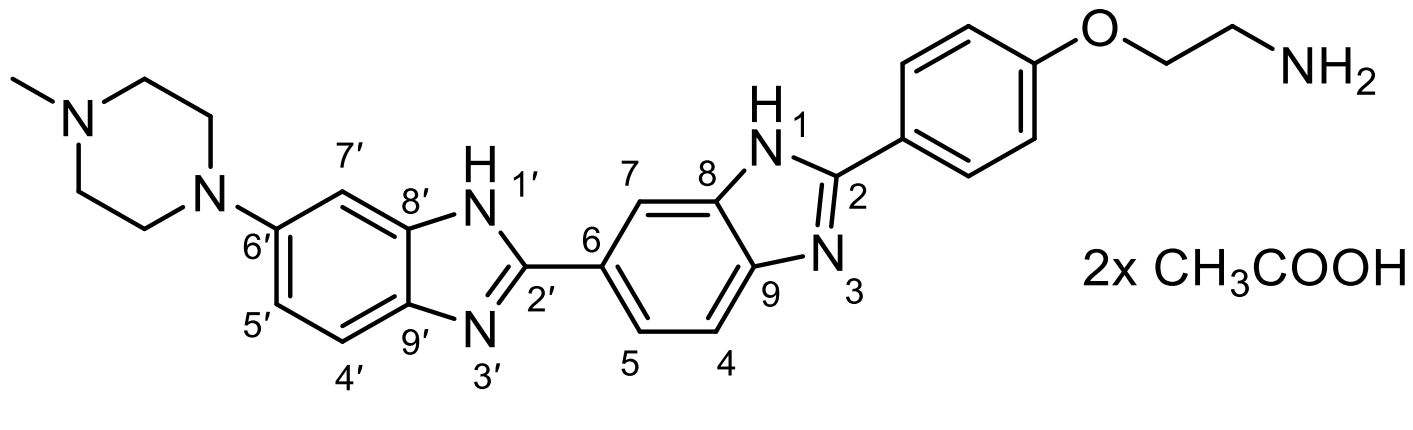

Hoechst 33258 *O*-ethyl-Boc-carbamate 36 (75 mg, 0.13 mmol, 1 equiv.) was dissolved in **DCM**, cooled to 0 °C, followed by dropwise addition of **TFA** (0.5 mL, 6.6 mmol, 50 equiv.). The reaction mixture was allowed to warm to RT and stirred at RT for 1h. Afterwards, volatiles were evaporated under reduced pressure, followed by ion exchange on Dowex (50W-X8) to exchange TFA$^-$ for AcO$^-$ anion to be able to

observe the ratio of counterions in the ^{1}H NMR spectrum. The deprotected **Hoechst 33258 O-ethylamine (37)** was obtained as a diacetate salt in sufficient purity without any purification (65 mg, 84%).

^{1}H NMR (400 MHz, CD_3OD): δ = 8.26 (dd, J = 1.7, 0.7 Hz, 1H, **H**-7), 8.11–8.06 (m, 2H, C_{ipso}(C**H**CH)$_2C_{ipso}$O), 7.96 (dd, J = 8.5, 1.7 Hz, 1H, **H**-5), 7.69 (dd, J = 8.4, 0.7 Hz, 1H, **H**-4), 7.52 (d, J = 8.8 Hz, 1H, **H**-4'), 7.19–7.13 (m, 3H, **H**-7', C_{ipso}(CHC**H**)$_2C_{ipso}$O), 7.05 (dd, J = 8.8, 2.3 Hz, 1H, **H**-5'), 4.32–4.26 (m, 2H, OC**H$_2$**CH_2NH), 3.41–3.35 (m, 2H, OCH_2C**H$_2$**NH_2), 3.31–3.28 (m, 4H, $CH_3N(CH_2$C**H$_2$**$)_2$N), 2.94 (t, J = 5.0 Hz, 4H, CH_3N(C**H$_2$**$CH_2)_2$N), 2.57 (s, 3H, C**H$_3$**N), 1.95 (s, 6H, C**H$_3$**COOH). **^{13}C NMR** (101 MHz, CD_3OD): δ = 178.5 (CH_3**C**OOH), 161.4 (C_{ipso}(CHCH)$_2$**C**$_{ipso}$O), 155.0 (**C**-2), 153.9 (**C**-2'), 149.1 (**C**-6'), 142.2 (**C**-9), 140.4 (**C**-8, **C**-8'), 136.1 (**C**-9'), 129.7 (C_{ipso}(**C**HCH)$_2C_{ipso}$O), 125.7 (**C**-6), 124.0 (**C**$_{ipso}$(CHCH)$_2C_{ipso}$O), 122.6 (**C**-5), 116.6 (**C**-4', **C**-5'), 116.5 (**C**-4), 116.2 (C_{ipso}(CH**C**H)$_2C_{ipso}$O), 113.9 (**C**-7), 102.7 (C-7'), 65.7 (O**C**H_2CH_2NH), 55.7 (CH_3N(**C**$H_2CH_2)_2$N), 51.1 ($CH_3N(CH_2$**C**$H_2)_2$N), 45.2 (**C**H$_3$N), 40.3 (OCH_2**C**H_2NH), 22.7 (**C**H_3COOH). **HRMS** (ESI+): calculated for $C_{27}H_{30}O_7N_7^+$ ([M+H]$^+$) 468.2506, found 468.2505.

***Tert*-butyl (2-(perfluorobenzamido)ethyl)carbamate (38)**

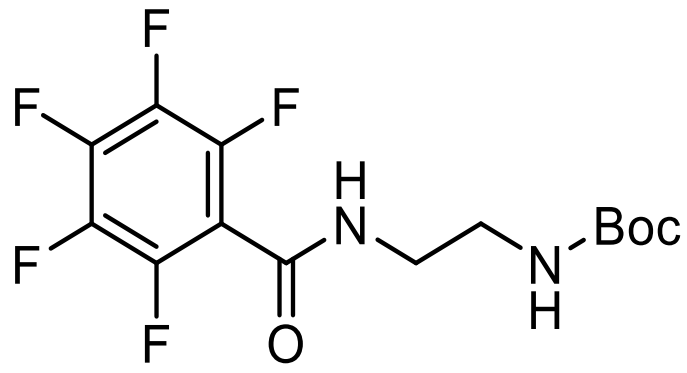

A solution of **tert-butyl (2-aminoethyl)carbamate** (891 mg, 5.6 mmol, 1 equiv.) in **DCM** (10 mL) was added to a solution of **2,3,4,5,6-pentafluorobenzoyl chloride** (800 L, 5.6 mmol, 1 equiv.) in **DCM** (5.0 mL) at 0 °C. After addition, the reaction mixture was warmed up to room temperature and stirred for 48 h. After complete consumption of the starting materials, the organic solvent was removed *in vacuo*. The crude product was purified by flash column chromatography (silica gel, eluent: gradient of PE/EtOAc) followed by evaporation to obtain ***tert*-butyl (2-(perfluorobenzamido)ethyl)carbamate (38)** (1.04 g, 52%) as an off-white solid.

^{1}H NMR (400 MHz, $CDCl_3$): δ = 7.20 (s, 1H), 4.97 (t, J = 6.3 Hz, 1H), 3.56 (q, J = 5.2 Hz, 2H), 3.38 (q, J = 5.9 Hz, 2H), 1.41 (s, 9H). **^{13}C NMR** (101 MHz, $CDCl_3$): δ = 158.0, 157.7, 145.5, 143.0, 138.7, 136.5, 80.5, 42.6, 39.7, 28.7, 28.4, 28.2, 28.0.

2-(perfluorobenzamido)ethan-1-aminium 2,2,2-trifluoroacetate (39)

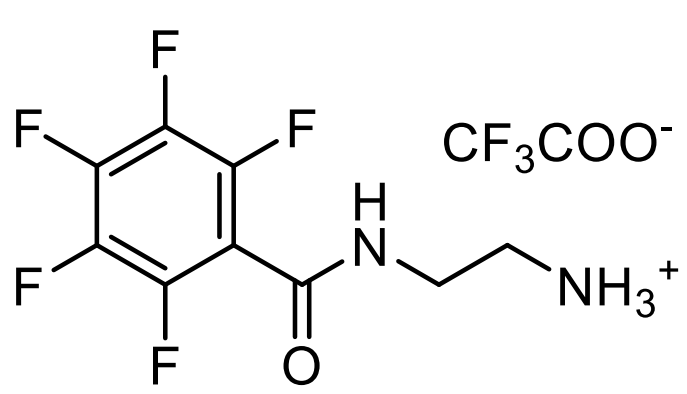

***tert*-Butyl (2-(perfluorobenzamido)ethyl)carbamate (38)** (1.0 g, 2.8 mmol, 1 equiv.) was dissolved in **DCM** (100 mL), and **TFA** (10 mL) was added. The reaction mixture was stirred at RT for 1 h. Then, liquids were removed *in vacuo*. The resulting crude reaction was suspended in a H_2O/MeCN mixture and lyophilised. After lyophilisation, **2-(perfluorobenzamido)ethan-1-aminium 2,2,2-trifluoroacetate (39)** (1.0 g, quant.) was obtained as a solid.

^{1}H NMR (400 MHz, $CDCl_3$): δ = 9.35–9.30 (m, 2H), 8.36–8.31 (m, 3H), 3.70–3.61 (m, 2H), 3.12–3.05 (m, 2H). **^{13}C NMR** (101 MHz, $CDCl_3$): δ = 159.2, 145.1, 142.6, 138.6, 136.1, 38.9, 38.7, 38.5, 37.6, 37.4, 37.1.

tert-butyl (4-(N-(cyclohexylcarbamoyl)sulfamoyl)phenethyl)carbamate (40)

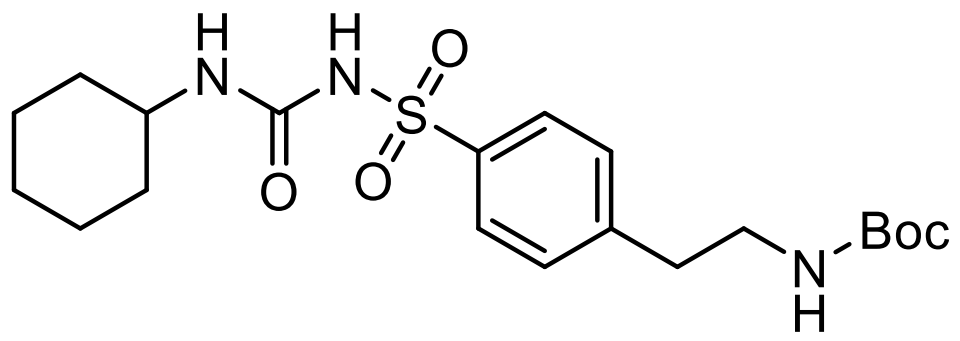

This compound was synthesized according to the published protocol.[14]

Tert-butyl (4-sulfamoylphenethyl)carbamate (500 mg, 1.7 mmol, 1 equiv.) and **K_2CO_3** (2.3 g, 16.6 mmol, 9.8 equiv.) were suspended in **acetone** (30 mL) and stirred at RT for 6 hours. Then, **cyclohexylisocyanate** (1.1 mL, 8.6 mmol, 5 equiv.) was added. After the addition, the reaction mixture was warmed up to 60 °C and stirred for 1 h. Afterwards, the reaction mixture was filtered, and the filtrate was concentrated *in vacuo*. The resulting reaction residue was dissolved in EtOAc (50 mL) and washed with brine solution (3 × 25 mL). Organic extract was dried with $MgSO_4$, filtered, and concentrated *in vacuo*. The crude product was purified by flash column chromatography (silica gel, eluent: gradient of PE/EtOAc) and then evaporated to obtain ***tert*-butyl (4-(N-(cyclohexylcarbamoyl)sulfamoyl)phenethyl)carbamate (40)** as a solid (500 mg, 66%).

^{1}H NMR (400 MHz, DMSO-d_6): δ = 7.63 (d, J = 8.2 Hz, 2H), 7.14 (d, J = 8.3 Hz, 2H), 3.18–3.06 (m, 2H), 2.70 (dd, J = 8.5, 6.5 Hz, 2H), 1.59–1.67 (m, J = 16.0, 11.7, 6.4 Hz, 8H), 1.38 (s, 9H), 1.30–0.90 (m, 8H). **^{13}C NMR** (101 MHz, $CDCl_3$): δ = 157.2, 156.0, 129.9, 129.6, 127.4, 79.7, 49.6, 49.3, 49.0, 34.2, 34.0, 33.0, 28.6, 28.4, 25.6, 24.8. **HRMS** (ESI+): calculated for $C_{20}H_{31}N_3O_5SNa^+$ ($[M+Na]^+$) 514.2869, found 514.2863.

4-(2-aminoethyl)-N-(cyclohexylcarbamoyl)benzenesulfonamide, 2,2,2-trifluoroacetate salt (41)

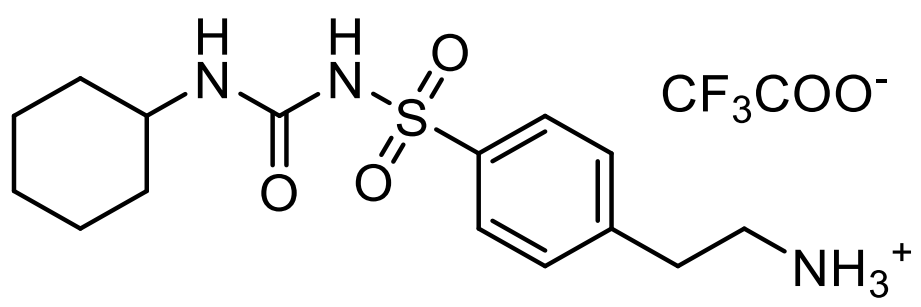

(4-(*N*-(Cyclohexylcarbamoyl)sulfamoyl)phenethyl)carbamate (40) (500 mg, 1.1 mmol, 1 equiv.) was dissolved in **DCM** (50 mL) and **TFA** (5 mL) was added. The reaction mixture was stirred at room temperature for 1 h. Then, liquids were removed *in vacuo*. The resulting residue was dissolved in an H_2O-

MeCN mixture and lyophilised. After lyophilisation, **4-(2-aminoethyl)-N-(cyclohexylcarbamoyl)benzenesulfonamide, 2,2,2-trifluoroacetate salt (41)** (500 mg, quant.) was obtained as a solid.

^{1}H NMR (401 MHz, $CDCl_3$/MeOD): δ = 7.80 (d, J = 7.8 Hz, 2H), 7.29 (d, J = 8.3 Hz, 2H), 3.44–3.19 (m, 3H), 3.11–3.00 (m, 2H), 2.93 (t, J = 7.7 Hz, 2H), 1.86–1.33 (m, 5H), 1.31–0.81 (m, 5H). **^{13}C NMR** (101 MHz, $CDCl_3$): δ = 151.2, 142.3, 138.9, 129.4, 128.0, 127.8, 40.1, 33.5, 33.3, 33.1, 24.5.

N-(2-aminoethyl)-4-methylbenzenesulfonamide (42)

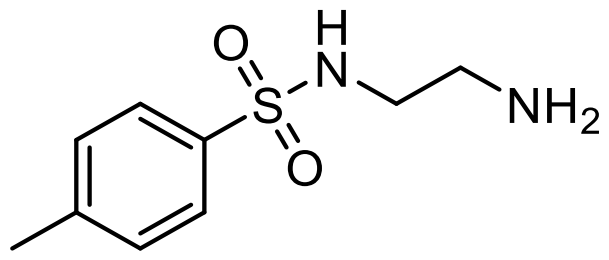

Ethylenediamine (4.6 mL, 85 mmol, 33 equiv.) was added to a solution of **4-methylbenzenesulfonyl chloride** (500 mg, 2.6 mmol, 1 equiv.) in **DCM** (50 mL) at 0 °C. After the addition, the reaction mixture was stirred for 1 h at 0 °C. Then it was allowed to warm to RT and stirred for another 18 h. Then, the reaction mixture was diluted with DCM (10 mL), and the pH was adjusted to neutral with 1N HCl. The resulting solution was washed with water. The combined aqueous phases were adjusted to pH 10 with 10N NaOH solution and washed with DCM (5 mL). All organic phases were combined, dried with Na_2SO_4, filtered and concentrated *in vacuo*. The product was used in the next reaction without additional purification.

^{1}H NMR (401 MHz, $CDCl_3$): δ = 7.74 (d, J = 8.3 Hz, 2H), 7.30 (d, J = 8.0 Hz, 2H), 2.98–2.91 (m, 2H), 2.81–2.74 (m, 2H), 2.42 (s, 3H). **^{13}C NMR** (101 MHz, $CDCl_3$): δ = 143.5, 137.1, 130.0, 130.0, 129.7, 127.3, 127.1, 127.1, 45.5, 41.0, 21.6.

2-tetradecanamidoethan-1-aminium 2,2,2-trifluoroacetate (43)

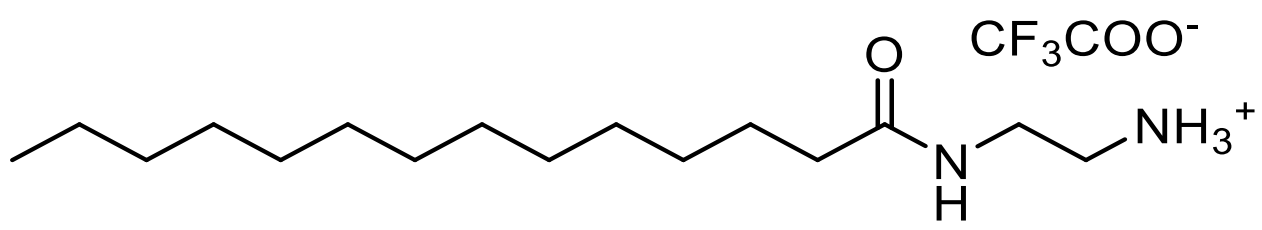

Under argon atmosphere, **myristic acid** (270 mg, 1.18 mmol, 1 equiv.) was mixed with **$SOCl_2$** (0.86 mL, 11.8 mmol, 10 equiv.) and stirred at reflux overnight. After cooling to RT, the resulting mixture was 3× co-evaporated with toluene under reduced pressure. The crude chloride was dissolved in **DCM** (12 mL). To that solution, *N*-boc-ethylenediamine (187 µL, 1.18 mmol, 1 equiv.) was added dropwise via syringe pump over 30 min as a solution in **DCM** (12 mL). Lastly, **Et_3N** (329 µL, 2.37 mmol, 2 equiv.) was added, and the reaction mixture was stirred overnight. Then, volatiles were evaporated under reduced pressure. The crude carbamate was then dissolved in **TFA** (2.2 mL, 29.6 mmol, 25 equiv.) with a drop of H_2O. After stirring for 2 h, LC-MS showed full deprotection of the Boc protecting group. The reaction mixture was further diluted with H_2O and then lyophilised. The crude ammonium salt was purified by reversed-phase

chromatography (C18 column, eluent: gradient of H_2O/MeCN) followed by lyophilisation, yielding **2-tetradecanamidoethan-1-aminium 2,2,2-trifluoroacetate (43)** (327 mg, 0.85 mmol, 72%) as a fine white powder.
^{1}H NMR (400 MHz, DMSO-*d*$_6$): δ = 7.99 (t, $^3J_{H\text{-}H}$ = 5.7 Hz, 1H, N**H**), 7.84 (bs, 3H, N**H$_3$$^+$**), 3.26 (dt, $^3J_{H\text{-}H}$ = 6.4 Hz, $^3J_{H\text{-}H}$ = 5.9 Hz, 2H, C**H$_2$**NH), 2.84 (t, $^3J_{H\text{-}H}$ = 6.5 Hz, 2H, C**H$_2$**NH_3^+), 2.07 (t, $^3J_{H\text{-}H}$ = 7.5 Hz, 2H, C**H$_2$**CONH), 1.48 (p, $^3J_{H\text{-}H}$ = 6.9 Hz, 2H, CH_3C**H$_2$**), 1.31–1.16 (m, 20H, C**H$_2$**), 0.89–0.80 (m, 3H, C**H$_3$**) ppm, **^{19}F NMR** (377 MHz, DMSO-*d*$_6$): δ = −73.6 (s, C**F$_3$**COO^-) ppm, **^{13}C NMR** (101 MHz, DMSO-*d*$_6$): δ = 173.0 (**C**ONH), 158.2 (q, $^2J_{C\text{-}F}$ = 31 Hz, CF_3**C**OO^-), 117.3 (q, $^1J_{C\text{-}F}$ = 300 Hz, **C**F_3COO^-), 38.7 (**C**$H_2NH_3^+$), 36.4 (**C**H_2NH_2), 35.3 (**C**H_2CONH), 31.3 (**C**H_2), 29.12–28.98 (m, **C**H_2), 28.9 (**C**H_2), 28.8 (**C**H_2), 28.7 (**C**H_2), 25.0 (**C**H_2), 22.1 (**C**H_2CH_3), 14.0 (**C**H_3) ppm, **LRMS** (ESI+), *m/z* (%): 271.3 $[M]^+$ (100). **HRMS** (ESI+): calculated for $C_{16}H_{35}ON_2^+$ ($[M]^+$) 271.2744, found 271.2746.

2-((tetrahydro-2H-pyran-2-yl)oxy)propanenitrile (44)

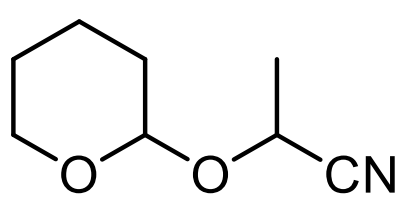

This compound was synthesized according to the published protocol.[15]
2-Hydroxypropanenitrile (6.11 g, 86 mmol, 1 equiv.) and **3,4-Dihydro-2H-pyran** (9.4 g, 112 mmol, 1.3 equiv.) were dissolved in **DCM** (170 mL). To the formed solution, ***p*-toluenesulfonic acid hydrate** (1.6 g, 8.6 mmol, 0.1 equiv.) was added, and the reaction mixture was stirred at RT for 18h. Afterwards, the crude reaction mixture was diluted with water, extracted with DCM, washed with brine, dried over $MgSO_4$, filtered, and evaporated to obtain **2-((tetrahydro-2H-pyran-2-yl)oxy)propanenitrile (44)** (13 g, 84 mmol, quant.) as a dark, dense liquid with satisfactory purity, as determined by NMR analysis.
The measured ^{1}H NMR spectrum corresponds to the published spectra.[16]
^{1}H NMR (400 MHz, $CDCl_3$): δ = 4.91–4.87 (m, 1H, C**H**CH_2), 4.79 (t, *J* = 2.9 Hz, 1H, C**H**CH_2), 4.64 (q, *J* = 6.9 Hz, 1H, C**H**CH_3), 4.38 (q, *J* = 6.9 Hz, 1H, C**H**CH_3), 4.01–3.50 (m, 2H, OC**H$_2$**), 1.90–1.45 (m, 9H, CHC**H$_3$**, CHC**H$_2$**C**H$_2$**C**H$_2$**) ppm.

1-(6-phenyl-1,2,4,5-tetrazin-3-yl)ethan-1-ol (45)

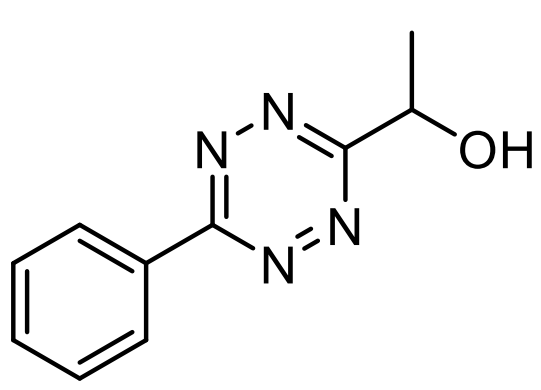

This compound was synthesized according to the procedure inspired by a published protocol.[17]
2-((tetrahydro-2H-pyran-2-yl)oxy)propanenitrile (44) (5.08 g, 32.75 mmol, 1 equiv.) and **benzonitrile** (13.4 mL, 131.0 mmol, 4 equiv.) were dissolved in **MeOH** (164 mL). Into the formed solution, **zinc triflate** (2.98 g, 8.19 mmol, 0.25 equiv.) was added, followed by **hydrazine hydrate** (40

mL, 818.6 mmol, 25 equiv.). The reaction mixture was stirred at 60 °C for 18h. Afterwards, the reaction mixture was cooled to RT, and the volatiles were evaporated under reduced pressure. The residue containing dihydrotetrazine precursor was redissolved in a mixture of **THF/AcOH** (164 mL) and cooled to 0 °C. To the cooled reaction mixture, a solution of **$NaNO_2$** (56.5 g, 818.6 mmol, 25 equiv.) in **water** (280 mL) was added dropwise with the dropping funnel. Then the reaction mixture was allowed to warm up to RT, and at RT, an additional 20 min. Afterwards, the volatiles were evaporated under reduced pressure. The residue containing the protected tetrazine precursor was redissolved in **MeOH** (164 mL) again, followed by the addition of ***p*-toluenesulfonic acid hydrate** (780 mg, 3.85 mmol, 0.1 equiv.), and the mixture was stirred at RT for 18 h. Finally, the volatiles were evaporated under reduced pressure, and the crude reaction mixture was diluted with water, extracted with DCM, washed with brine, dried over $MgSO_4$, filtered, and evaporated. The crude product was prepurified by flash chromatography over silica gel (eluent: gradient of DCM/MeOH) to remove lipophilic diphenyltetrazine as a main unwanted product, followed by reversed-phase chromatography (C18 column, eluent: gradient of H_2O/MeCN) to obtain **1-(6-phenyl-1,2,4,5-tetrazin-3-yl)ethan-1-ol (45)** (778 mg, 3.85 mmol, 12%) as a pink solid after lyophilisation.

The measured ^1H NMR spectrum corresponds to the published spectra.[17]

^{1}H NMR (400 MHz, $CDCl_3$): δ = 8.66–8.57 (m, 2H, C**H**CCNN), 7.70–7.56 (m, 3H, C**H**C**H**CHCCNN), 5.56–5.44 (m, 1H, C**H**CH_3), 3.39 (d, J = 5.8 Hz, 1H, O**H**), 1.82 (d, J = 6.7 Hz, 3H, C**H$_3$**) ppm.

2,5-dioxopyrrolidin-1-yl (1-(6-phenyl-1,2,4,5-tetrazin-3-yl)ethyl) carbonate (46)

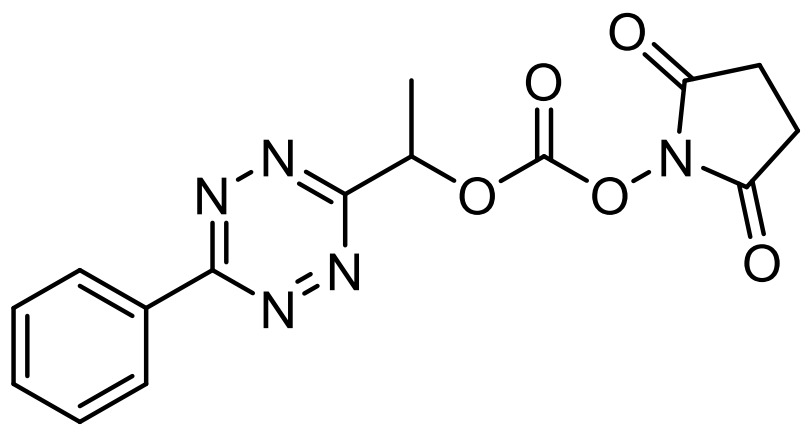

Under an argon atmosphere, **1-(6-phenyl-1,2,4,5-tetrazin-3-yl)ethan-1-ol (45)** (778 mg, 3.85 mmol, 1 equiv.) and ***N,N′*-disuccinimidyl carbonate** (3.94 g, 15.4 mmol, 4 equiv.) were dissolved in dry **MeCN** (39 mL). To the formed solution, **DIPEA** (2 mL, 11.5 mmol, 3 equiv.) was added, and the reaction mixture was stirred at RT for 2 h to confirm full conversion by LC-MS analysis. Afterwards, the crude reaction mixture was diluted with water, extracted with DCM, washed with brine, dried over $MgSO_4$, filtered, and evaporated. The crude product was purified by reversed-phase chromatography (C18 column, eluent: gradient of H_2O/MeCN) to obtain **2,5-dioxopyrrolidin-1-yl (1-(6-phenyl-1,2,4,5-tetrazin-3-yl)ethyl) carbonate (46)** (1.29 g, 3.76 mmol, quant.) as a pink powder after lyophilisation.

^{1}H NMR (400 MHz, $CDCl_3$): δ = 8.68–8.60 (m, 2H, C**H**CCNN), 7.70–7.57 (m, 3H, C**H**C**H**CHCCNN), 6.37 (q, J = 6.8 Hz, 1H, C**H**CH_3), 2.83 (s, 4H, C**H$_2$**), 2.02 (d, J = 6.8 Hz, 3H, C**H$_3$**CH) ppm, **^{13}C NMR** (101 MHz, $CDCl_3$): δ = 168.4 (CH_2**C**ON), 166.6 (CH**C**NN), 165.5 (CHC**C**NN), 151.4 (O**C**OO), 133.4 (**C**HCHCHCCNN), 131.4

(CHCCNN), 129.5 (**C**HCHCCNN), 128.6 (**C**HCCNN), 77.1 (**C**HCH$_3$), 25.6 (**C**H$_2$), 19.4 (**C**H$_3$CH) ppm, **LRMS** (ESI+), *m/z* (%): 366.1 [M+Na]$^+$ (100), 344.1 [M+H]$^+$ (10), **HRMS** (ESI+): calculated for $C_{15}H_{14}O_5N_5^+$ ([M+H]$^+$) 344.0990, found 344.0987, calculated for $C_{15}H_{13}O_5N_5Na^+$ ([M+Na]$^+$) 366.0809, found 366.0807.

2.3 Synthesis of the organelle-targeting TCO probes

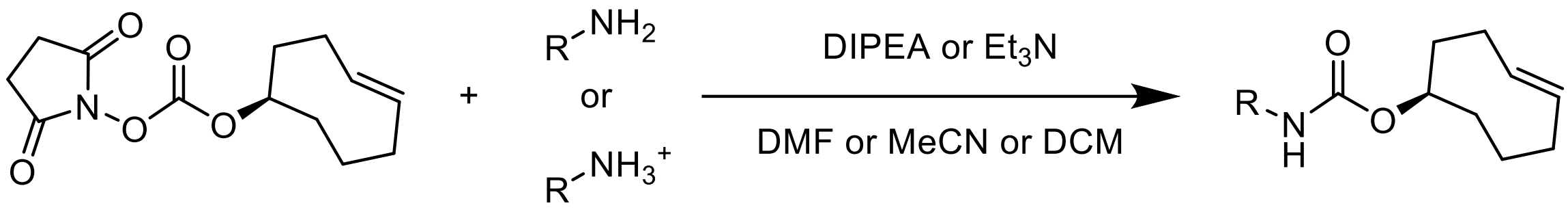

General procedure for the synthesis of the organelle-targeting TCO probes:

Under argon atmosphere, **TCO-4-en-NHS ester** (from SiChem, SC-8072) and the corresponding **amine** (or **ammonium salt**) were dissolved in dry solvent (**DMF**, **MeCN** or **DCM**), followed by the addition of base (**DIPEA** or **Et$_3$N**). The reaction mixture was stirred overnight at RT or until TLC or LC-MS analysis showed full conversion. The crude reaction mixture was extracted with DCM, washed with brine, dried over $MgSO_4$, filtered, and evaporated. The crude product was purified by reversed-phase chromatography, flash column chromatography or preparative HPLC followed by lyophilisation or evaporation to obtain the desired organelle-targeting TCO probes.

PM-TCO (1)

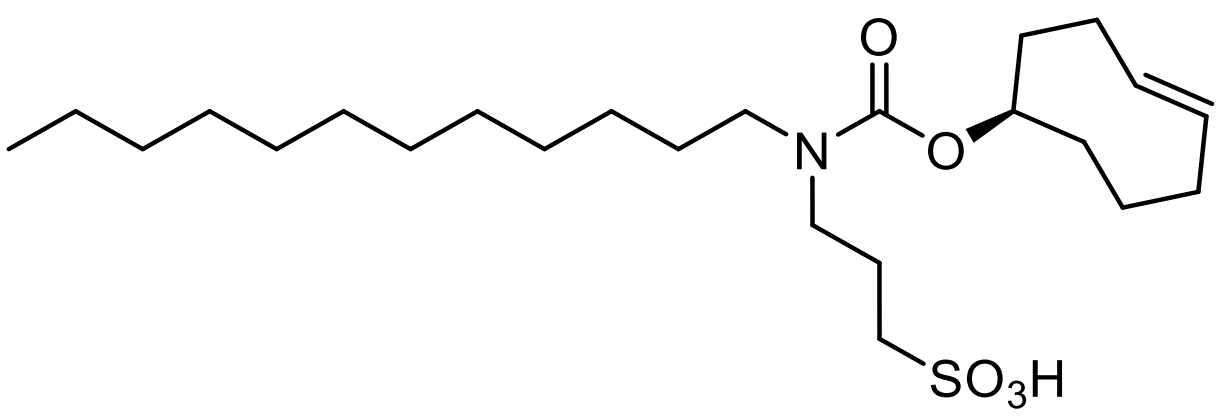

TCO-4-en-NHS ester (30 mg, 0.11 mmol, 1 equiv.), **3-(dodecylamino)propane-1-sulfonic acid (35)** (41 mg, 0.14 mmol, 1.2 equiv.), **Et$_3$N** (39 µL, 0.28 mmol, 2,5 equiv.), dry **MeCN** (1.5 mL). After extraction, the crude product was re-purified by reversed-phase chromatography (C18 column, eluent: gradient of H_2O/MeCN + 0.05% HCOOH) followed by lyophilisation to obtain partially purified **PM-TCO (1)**. Residual **3-(dodecylamino)propane-1-sulfonate** was separated from the desired product by flash column chromatography in HILIC mode (silica gel, eluent: gradient of MeCN/H_2O) followed by lyophilisation to obtain slightly impure **PM-TCO (1)**. The final purification was accomplished by flash column chromatography (silica gel, eluent: gradient of DCM/MeOH) to obtain pure **PM-TCO (1)** (30 mg, 57%) as a colourless oil.

In the NMR spectra (both ^1H and ^{13}C), signals of two rotamers of the amide bond were observed. The presence of these rotamers was confirmed by NMR at 55 °C. **^{1}H NMR** (400 MHz, CD_3OD): δ = 5.75–5.58 (m, 2H,

CHCH), 4.73 (td, J = 9.1, 4.2 Hz, 1H, COOCH), 3.36 (t, J = 7.4 Hz, 2H, $CH_2NCH_2CH_2CH_2SO_3H$), 3.25 (dd, J = 8.4, 6.5 Hz, 2H, $CH_2NCH_2CH_2CH_2SO_3H$), 2.78 (t, J = 7.7 Hz, 2H, CH_2SO_3H), 2.44–1.5 (m, 13H, CH_2), 1.33–1.24 (m, 19H, CH_2), 0.93–0.87 (m, 3H, CH_3). **^{13}C NMR** (101 MHz, CD_3OD): δ = 157.7 (NCOO), 130.9 (CHCH), 130.7 (CHCH), [77.9, 77.7 (COOCH)] 50.1 (CH_2SO_3H), [48.0, 47.9 ($CH_2NCH_2CH_2CH_2SO_3H$)], [47.3, 47.0 ($CH_2NCH_2CH_2CH_2SO_3H$)], 35.2 (CH_2), 34.7 (CH_2), 34.5 (CH_2), 33.6 (CH_2), 33.2 (CH_2), 33.1 (CH_2), 31.4 (CH_2), 30. 8 (CH_2), 30.8 (CH_2), 30.7 (CH_2), 30.7 (CH_2), 30.5 (CH_2), 29.6 (CH_2), 29.5 (CH_2), 29.1 (CH_2), 27.8 (CH_2), 26.5 (CH_2), 25.9 (CH_2), 25.5 (CH_2), 24.9 (CH_2), 23.7 (CH_2), 23.3 (CH_2), 14.5 (CH_3). **HRMS** (ESI−): calculated for $C_{24}H_{44}O_5NS^-$ ($[M-H]^-$) 458.2946, found 458.2942.

Mito-TCO (4)

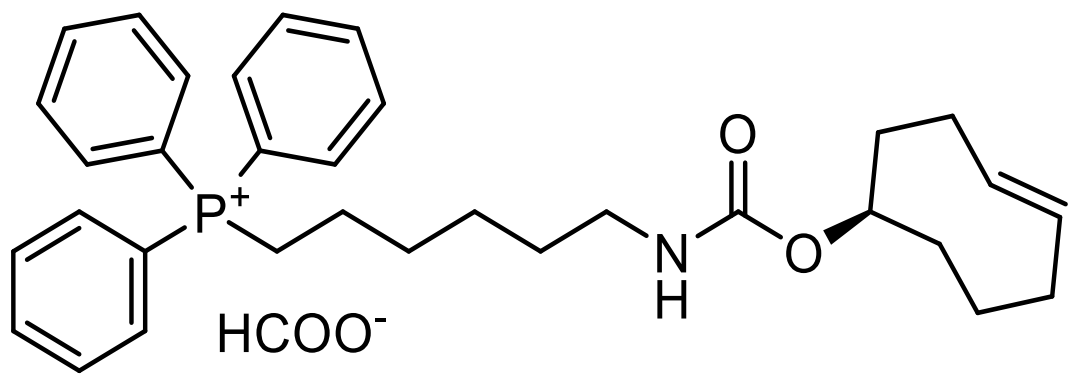

TCO-4-en-NHS ester (10 mg, 37 µmol, 1 equiv.), **(6-aminohexyl)triphenylphosphonium bromide hydrobromide** (25 mg, 56 µmol, 1.5 equiv.), **DIPEA** (33 µL, 0.19 mmol, 5 equiv.), **DMF** (3 mL). After the extraction, the crude product was purified by reversed-phase chromatography (C18 column, eluent: gradient of H_2O/MeCN) followed by lyophilisation to obtain **Mito-TCO (4)** as a white powder (15 mg, 72%).

^{1}H NMR (400 MHz, DMSO-d_6): δ = 8.56 (s, 1H, $HCOO^-$), 8.01–7.66 (m, 15H, CH^{Ar}), 7.15 (t, $^3J_{H-H}$ = 5.8 Hz, 1H, NH), 5.72–5.41 (m, 2H, CH_2CHCH), 4.69 (dd, $^3J_{H-H}$ = 10.5 Hz, 5.3 Hz, 1H, COOCH), 3.64–3.52 (m, 2H, PCH_2), 2.98–2.87 (m, 2H, $NHCH_2$), 2.35–1.92 (m, 5H, CH_2), 1.87–1.08 (m, 13H, CH_2) ppm, **^{31}P NMR** (162 MHz, C_6D_6): δ = 27.8 ppm, **^{13}C NMR** (101 MHz, DMSO-d_6): δ = 165.0 ($HCOO^-$), 155.8 (COO), 135.0 (CH_2CH), 134.9 (d, $^4J_{C-P}$ = 3.0 Hz, CH^{Ar}), 133.6 (d, $^3J_{C-P}$ = 10.1 Hz, CH^{Ar}), 131.5 (CH_2CH), 130.2 (d, $^2J_{C-P}$ = 12.4 Hz, CH^{Ar}), 118.6 (d, $^1J_{C-P}$ = 85.7 Hz, PCCH), 68.2 (COOCH), 40.5 (CH_2), 33.9 (CH_2), 32.2 (CH_2), 29.6 (CH_2), 29.4 (CH_2), 29.1 (CH_2), 27.4 (CH_2), 25.4 (CH_2), 21.8 (d, $^3J_{C-P}$ = 4.2 Hz, PCH_2CH_2), 20.1 (d, $^2J_{C-P}$ = 50.0 Hz, PCH_2) ppm, **LRMS** (ESI+), m/z (%): 514.3 $[M]^+$ (100). **HRMS** (ESI+): calculated for $C_{33}H_{41}O_2NP^+$ ($[M]^+$) 514.2869, found 514.2863.

Nucl-TCO (7)

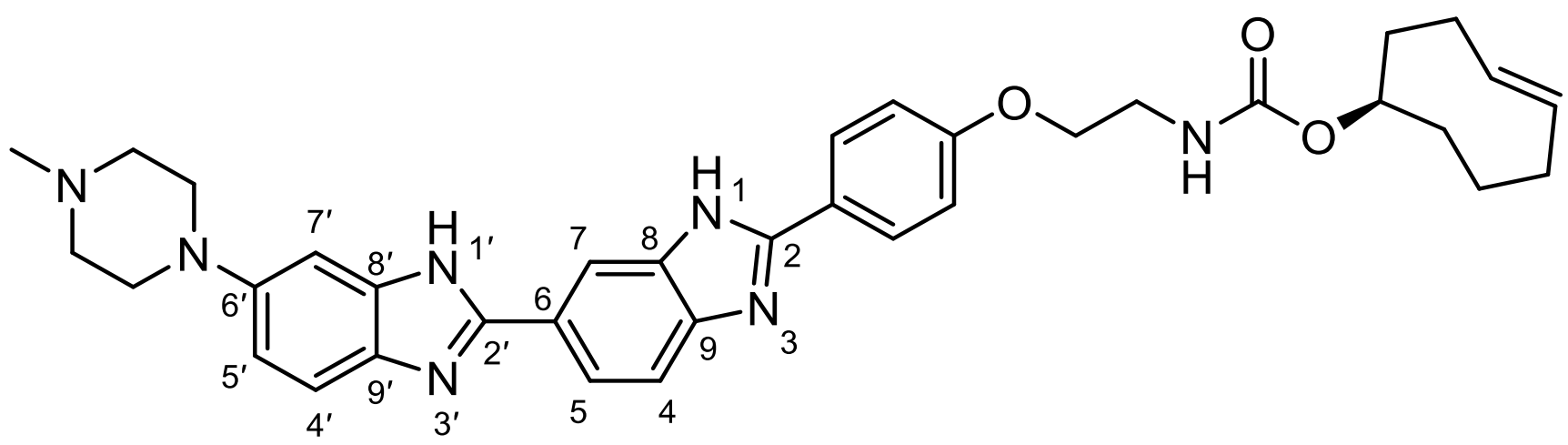

TCO-4-en-NHS ester (10 mg, 0.34 mmol, 1.1 equiv.), **Hoechst 33258 *O*-ethylamine 37** (20 mg, 0.34 mmol, 1 equiv.), **Et_3N** (24 µL, 0.17 mmol, 5 equiv.), dry **DMF** (4 mL). TLC analysis (DCM/MeOH + 1% Et_3N) showed full conversion in 2h. After extraction, the crude product was purified by flash column chromatography (silica gel, eluent: gradient of DCM/MeOH + 1% Et_3N) followed by evaporation to obtain **Nucl-TCO (7)** (16 mg, 80%) as a yellow-brown solid.

^{1}H NMR (400 MHz, CD_3OD): δ = 8.20 (d, *J* = 1.7 Hz, 1H, **H**-7), 8.04–7.97 (m, 2H, C_{ipso}(C**H**CH)$_2$$C_{ipso}$O), 7.91 (dd, *J* = 8.5, 1.6 Hz, 1H, **H**-5), 7.64 (d, *J* = 8.5 Hz, 1H, **H**-4), 7.48 (d, *J* = 8.8 Hz, 1H, **H**-4'), 7.11 (d, *J* = 2.2 Hz, 1H, **H**-7'), 7.07–7.03 (m, 2H, C_{ipso}(CHC**H**)$_2$$C_{ipso}$O), 7.01 (dd, *J* = 8.8, 2.2 Hz, 1H, **H**-5'), 5.72–5.46 (m, 2H, TCO-C**H**C**H**), 4.86–4.80 (m, 1H, COOC**H**), 4.07 (t, *J* = 5.6 Hz, 1H, OC**H₂**CH$_2$NH), 3.51 (dt, *J* = 5.4, 2.9 Hz, 2H, OCH$_2$C**H₂**NH), 3.25 (t, *J* = 5.0 Hz, 4H, CH_3N(CH$_2$C**H₂**)$_2$N), 2.84 (t, *J* = 4.9 Hz, 4H, CH_3N(C**H₂**CH$_2$)$_2$N), 2.50 (s, 3H, C**H₃**N), 2.43–2.16 (m, 4H, C**H₂**-TCO), 2.10–1.97 (m, 1H, C**H₂**-TCO), 1.87–1.49 (m, 4H, C**H₂**-TCO), 1.29 (s, 1H, C**H₂**-TCO). **^{13}C NMR** (101 MHz, CD_3OD): δ = 160.9 (C_{ipso}(CHCH)$_2$**C**$_{ipso}$O), 157.4 (NH**C**OO), 153.8 (**C**-2), 152.4 (**C**-2'), 147.8 (**C**-6'), 138.9 (**C**-8, **C**-8'), 135.0 (TCO-**C**HCH), 134.7 (C-9'), 131.1 (TCO-**C**HCH), 128.2 (C_{ipso}(**C**HCH)$_2$$C_{ipso}$O), 124.1 (**C**-6), 121.7 (**C**$_{ipso}$(CHCH)$_2$$C_{ipso}$O), 121.1 (**C**-5), 115.2 (C-4'), 115.0 (**C**-5'), 114.7 (**C**-4, C_{ipso}(CH**C**H)$_2$$C_{ipso}$O), 112.4 (**C**-7) 101.1 (**C**-7'), 70.2 (COO**C**H), 66.6 (O**C**H$_2$CH$_2$NH), 54.5 (CH_3N(**C**H$_2$CH$_2$)$_2$N), 49.9 (CH_3N(CH$_2$**C**H$_2$)$_2$N), 44.1 (**C**H₃N), 40.6 (**C**H$_2$-TCO), 39.9 (O**C**H$_2$CH$_2$NH), 33.9 (**C**H$_2$-TCO), 32.3 (**C**H$_2$-TCO), 29.5 (**C**H$_2$-TCO), 27.6 (**C**H$_2$-TCO). **HRMS** (ESI+): calculated for $C_{36}H_{42}O_3N_7^+$ ($[M+H]^+$) 620.3344, found 620.3341.

ER-F-TCO (9)

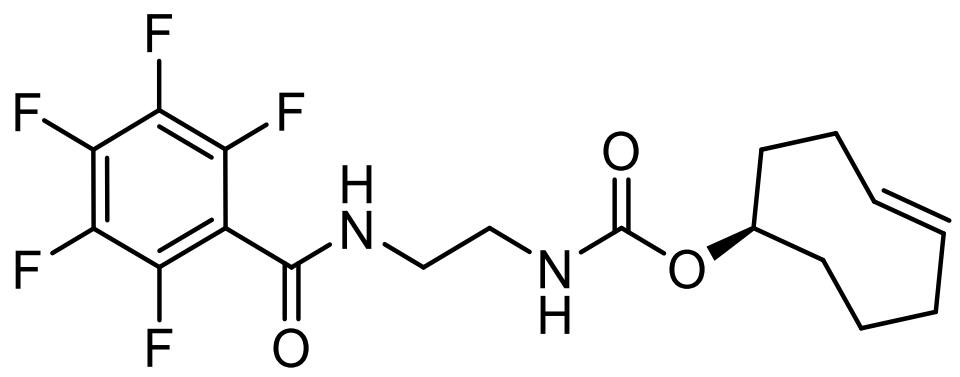

TCO-4-en-NHS ester (20 mg, 75 µmol, 1 equiv.), **2-(perfluorobenzamido)ethan-1-aminium 2,2,2-trifluoroacetate (39)** (28 mg, 76 µmol, 1 equiv.), **DIPEA** (130 µL, 0.75 mmol, 10 equiv.), **DMF** (3 mL). After the extraction, the crude product was purified by reversed-phase chromatography (C18 column, eluent: gradient of H_2O/MeCN) followed by lyophilization to obtain **ER-F-TCO (9)** as a white powder (24 mg, 79%).

^{1}H NMR (400 MHz, C_6D_6): δ = 6.53 (bs, 1H, N**H**), 5.74–5.62 (m, 1H, C**H**CH), 5.45–5.33 (m, 1H, C**H**CH), 4.97–4.89 (m, 1H, COOC**H**), 4.22–4.14 (m, 1H, N**H**), 3.25–3.07 (m, 2H, C**H₂**NH), 2.86–2.72 (m, 2H, C**H₂**NH), 2.45–2.31 (m, 1H, C**H₂**^{TCO}), 2.30–2.11 (m, 3H, C**H₂**^{TCO}), 2.03–1.90 (m, 1H, C**H₂**^{TCO}), 1.85–1.45 (m, 3H, C**H₂**^{TCO}), 1.37–1.24 (m, 1H, C**H₂**^{TCO}), 0.97–0.85 (m, 1H, C**H₂**^{TCO}) ppm, **^{19}F NMR** (377 MHz, C_6D_6): δ = −141.9–−142.1 (m, 2F), −152.6–−152.8 (m, 1F), −161.1–−161.3 (m, 2F) ppm, **^{13}C NMR** (101 MHz, C_6D_6, *aromatic carbons were not observed due to the C-F coupling*): δ = 157.6 (**C**O), 157.4 (**C**O), 135.4 (CH$_2$C**H**CH), 132.0 (CH$_2$C**H**CH), 70.9 (COO**C**H), 42.1 (NH**C**H$_2$), 41.1 (TCO-**C**H$_2^{TCO}$), 40.1 (NH**C**H$_2^{TCO}$), 34.6 (TCO-**C**H$_2^{TCO}$), 32.8

(TCO-$\mathbf{C}H_2^{TCO}$), 30.3 (TCO-$\mathbf{C}H_2^{TCO}$), 28.3 (TCO-$\mathbf{C}H_2^{TCO}$) ppm. **LRMS** (ESI−), *m/z* (%): 441.1 $[M+Cl]^-$ (100), 405.1 $[M-H]^-$ (55). **HRMS** (ESI−): calculated for $C_{18}H_{19}O_3N_2ClF_5^-$ ($[M+Cl]^-$) 441.1010, found 441.1008. Calculated for $C_{18}H_{18}O_3N_2F_5^-$ ($[M-H]^-$) 405.1243, found 405.1242.

ER-Cl-TCO (11)

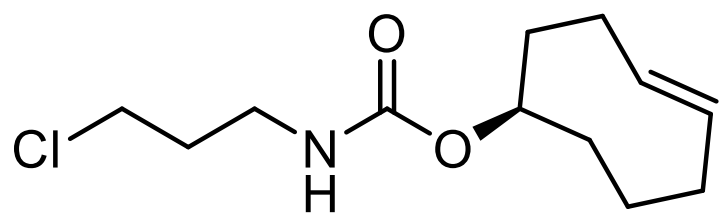

TCO-4-en-NHS ester (20 mg, 75 µmol, 1 equiv.), **3-chloropropylamine hydrochloride** (20 mg, 0.15 mmol, 2 equiv.), **DIPEA** (130 µL, 0.75 mmol, 10 equiv.), **MeCN** (3 mL). After the extraction, the crude product was purified by reversed-phase chromatography (C18 column, eluent: gradient of H_2O/MeCN) followed by lyophilisation to obtain **ER-Cl-TCO (11)** as an oilish colourless liquid (14 mg, 76%).

^{1}H NMR (400 MHz, C_6D_6): δ = 5.75–5.63 (m, 1H, C**H**CH), 5.47–5.35 (m, 1H, C**H**CH), 5.00 (dd, $^3J_{H\text{-}H}$ = 10.5 Hz, $^3J_{H\text{-}H}$ = 5.4 Hz, 1H, COOC**H**), 4.07 (bs, 1H, N**H**), 3.02 (t, $^3J_{H\text{-}H}$ = 6.4 Hz, 2H, C**H₂**Cl), 2.95 (q, $^3J_{H\text{-}H}$ = 6.5 Hz, 2H, C**H₂**NH), 2.48–2.31 (m, 1H, C**H₂**), 2.31–2.12 (m, 3H, C**H₂**), 2.03–1.93 (m, 1H, C**H₂**), 1.85–1.38 (m, 5H, C**H₂**), 1.38–1.26 (m, 1H, C**H₂**), 0.99–0.87 (m, 1H, C**H₂**) ppm, **^{13}C NMR** (101 MHz, C_6D_6): δ = 156.0 (**C**O), 135.5 (**C**H), 132.0 (**C**H), 70.1 (COO**C**H), 42.3 (**C**H_2Cl), 41.2 (**C**H_2), 38.5 (**C**H_2), 34.7 (**C**H_2), 32.9 (**C**H_2), 30.4 (**C**H_2), 28.4 (**C**H_2) ppm. **LRMS** (ESI+), *m/z* (%): 268.1 $[M+Na]^+$ (100). **HRMS** (ESI+): calculated for $C_{12}H_{20}O_2NClNa^+$ ($[M+Na]^+$) 268.1075, found 268.1075.

ER-SU1-TCO (14)

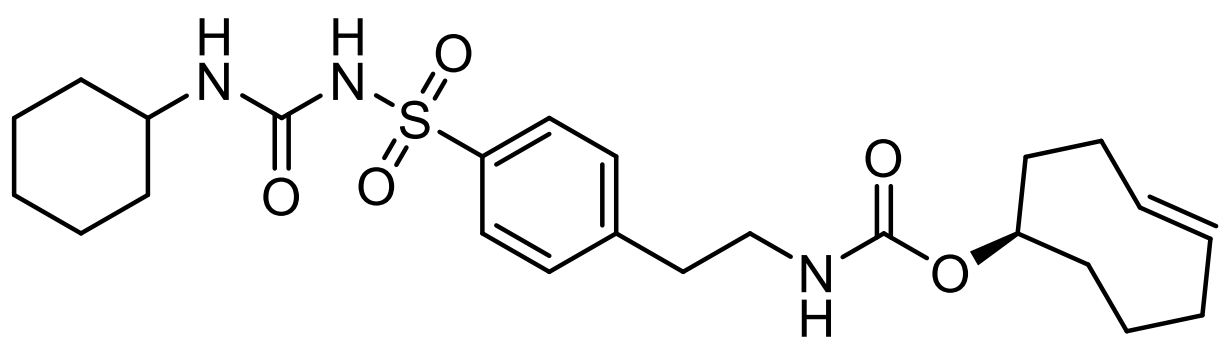

TCO-4-en-NHS ester (20 mg, 75 µmol, 1 equiv.), **4-(2-aminoethyl)-N-(cyclohexylcarbamoyl)benzenesulfonamide, 2,2,2-trifluoroacetate salt (41)** (24 mg, 55 µmol, 1 equiv.), **DIPEA** (130 µL, 0.75 mmol, 13 equiv.), **DMF** (3 mL). After the extraction, the crude product was purified by reversed-phase chromatography (C18 column, eluent: gradient of H_2O/MeCN) followed by lyophilisation to obtain **ER-SU1-TCO (14)** as a white powder (14 mg, 53%).

^{1}H NMR (400 MHz, DMSO-d_6): δ = 10.29 (s, 1H, SO_2N**H**), 7.80 (d, $^3J_{H\text{-}H}$ = 8.2 Hz, 2H, SO_2CC**H**), 7.43 (d, $^3J_{H\text{-}H}$ = 8.2 Hz, 2H, CH_2CC**H**), 7.20 (t, $^3J_{H\text{-}H}$ = 5.7 Hz, 1H, CHN**H**), 6.33 (d, $^3J_{H\text{-}H}$ = 7.8 Hz, 1H, CH_2N**H**), 5.69–5.41 (m, 2H, CH_2C**H**C**H**), 4.76–4.63 (m, 1H, COOC**H**), 3.45–3.14 (m, 4H, NHC**H**, NHC**H₂**), 2.92–2.76 (m, 2H, NHCH₂C**H₂**), 2.30–1.95 (m, 4H, C**H₂**CHCHC**H₂**), 1.85–1.01 (m, 15H, C**H₂**) ppm, **^{13}C NMR** (101 MHz, DMSO-d_6): δ = 155.7 (**C**OO), 150.5 (NH**C**ONH), 145.2 (CH_2CH_2**C**^{Ar}), 138.2 (SO_2**C**^{Ar}), 135.0 (CH_2**C**HCHCH_2), 131.4 (CH_2**C**HCHCH_2), 129.2 (CH_2C**C**H), 127.2 (SO_2C**C**H), 68.3 (COO**C**H), 48.0 (CONH**C**H), 41.1 (**C**H_2), 40.4 (**C**H_2), 35.2 (**C**H_2), 33.9 (**C**H_2), 32.3 (**C**H_2), 32.1 (**C**H_2), 29.6 (**C**H_2), 27.3 (**C**H_2), 25.0 (**C**H_2), 24.16 (**C**H_2) ppm. **LRMS**

(ESI−), *m/z* (%): 476.2 $[M-H]^-$ (100), 935.5 $[2M-H]^-$ (5). **HRMS** (ESI−): calculated for $C_{24}H_{34}O_5N_3S^-$ ($[M-H]^-$) 476.2225, found 476.2223.

ER-SU2-TCO (17)

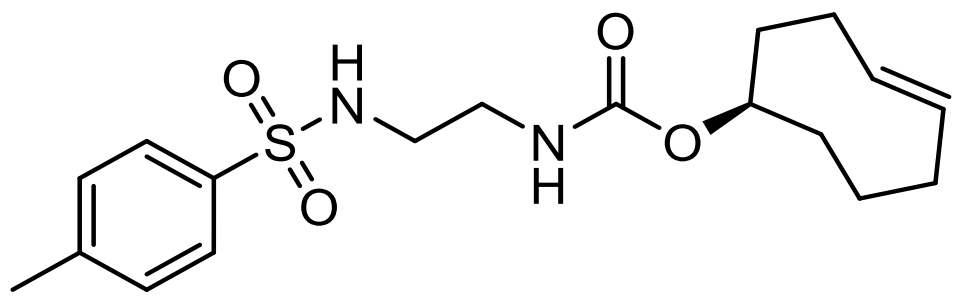

TCO-4-en-NHS ester (20 mg, 0.08 mmol, 1 equiv.), **N-(2-aminoethyl)-4-methylbenzenesulfonamide (42)** (17 mg, 0.08 mmol, 1.1 equiv.), **DIPEA** (26 µL, 0.15 mmol, 2 equiv.), dry **DCM** (2 mL). TLC analysis (PE/EtOAc) showed full conversion in 3h. After extraction, the crude product was purified by flash column chromatography (silica gel, eluent: gradient of DCM/MeOH) followed by evaporation to obtain **ER-SU2-TCO (17)** as a white solid (28 mg, quant.).

^{1}H NMR (400 MHz, $CDCl_3$): δ = 7.76–7.73 (m, 2H, C**H**CSO$_2$NH), 7.33–7.28 (m, 2H, C**H**CHCSO$_2$NH), 5.56–5.48 (m, 2H, C**H**C**H**), 5.22 (t, *J* = 6.1 Hz, 1H, SO$_2$N**H**), 5.12 (d, *J* = 6.3 Hz, 1H, CON**H**), 4.99–4.83 (m, 1H, COOC**H**), 3.30 (q, *J* = 5.9 Hz, 2H, CONHC**H$_2$**), 3.08 (q, *J* = 5.7 Hz, 2H, SO$_2$NHC**H$_2$**), 2.42 (s, 3H, C**H$_3$**), 2.35–2.19 (m, 4H, TCO-C**H$_2$**), 2.13–2.03 (m, 1H, TCO-C**H$_2$**), 1.91–1.71 (m, 2H, TCO-C**H$_2$**), 1.70–1.58 (m, 1H, TCO-C**H$_2$**), 1.55–1.40 (m, 1H, TCO-C**H$_2$**), 1.26–1.15 (m, 1H, TCO-C**H$_2$**). **^{13}C NMR** (101 MHz, $CDCl_3$): δ = 156.9 (**C**OO), 143.7 (CH$_3$**C**), 136.9 (**C**SO$_2$NH), 135.5 (**C**HCH), 131.8 (**C**HCH), 129.9 (**C**HCHCSO$_2$NH), 127.2 (CH**C**HCSO$_2$NH), 70.8 (COO**C**H), 43.7 (SO$_2$NH**C**H$_2$), 41.2 (TCO-**C**H$_2$), 40.8 (**C**H$_2$NHCOO), 34.4 (TCO-**C**H$_2$), 32.8 (TCO-**C**H$_2$), 30.0 (TCO-**C**H$_2$), 28.1 (TCO-**C**H$_2$), 21.7 (**C**H$_3$). **HRMS** (ESI+): calculated for $C_{18}H_{26}O_4N_2NaS^+$ ($[M+H]^+$) 389.1506, found 389.1503.

Golgi-TCO (19)

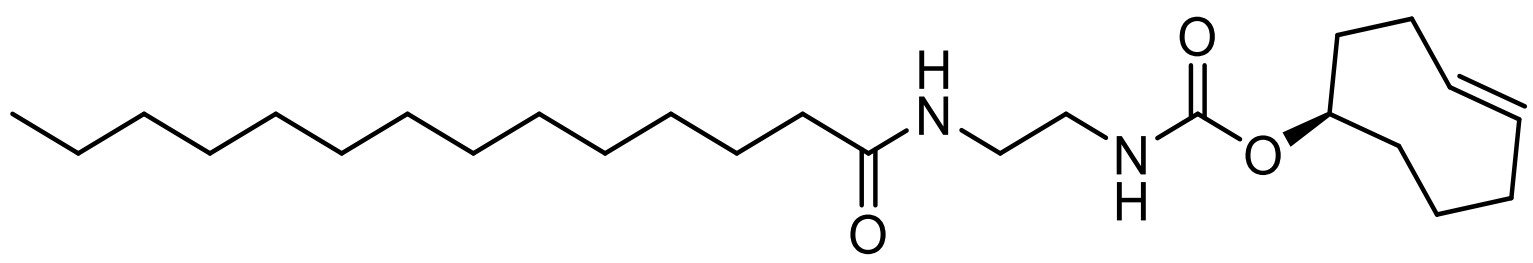

TCO-4-en-NHS ester (25 mg, 94 µmol, 1 equiv.), **2-tetradecanamidoethan-1-aminium 2,2,2-trifluoroacetate (43)** (36 mg, 94 µmol, 1 equiv.), **DIPEA** (163 µL, 0.94 mmol, 10 equiv.), **DMF** (3 mL). After the extraction, the crude product was purified by reversed-phase chromatography (C18 column, eluent: gradient of H_2O/MeCN) followed by lyophilisation to obtain **Golgi-TCO (19)** as a white powder (22 mg, 56%).

^{1}H NMR (400 MHz, C_6D_6): δ = 5.86–5.70 (m, 3H, N**H**, C**H**CH), 5.48–5.36 (m, 3H, N**H**, C**H**CH), 5.05 (dd, $^3J_{H\text{-}H}$ = 10.3 Hz, $^3J_{H\text{-}H}$ = 5.1 Hz, 1H, COOC**H**), 3.28–3.01 (m, 4H, NHC**H$_2$**), 2.53–2.36 (m, 1H, C**H$_2$**), 2.34–2.17 (m, 3H, C**H$_2$**), 2.07–1.89 (m, 4H, C**H$_2$**), 1.87–1.58 (m, 5H, C**H$_2$**), 1.44–1.17 (m, 19H, C**H$_2$**), 1.06–0.85 (m, 3H, C**H$_3$**) ppm, **^{13}C NMR** (101 MHz, C_6D_6): δ = 173.5 (**C**ONH), 157.2 (**C**ONH), 135.6 (**C**H), 131.9 (**C**H), 70.2 (**C**OOCH), 41.3 (**C**H$_2$NH), 40.4 (**C**H$_2$NH), 36.7 (**C**H$_2$), 34.8 (**C**H$_2$), 33.0 (**C**H$_2$), 32.4 (**C**H$_2$), 30.4 (**C**H$_2$), 30.3–

30.1 (m, **C**H_2), 30.1 (**C**H_2), 29.9 (**C**H_2), 29.9 (**C**H_2), 29.8 (**C**H_2), 28.4 (**C**H_2), 26.1 (**C**H_2), 23.1 (**C**H_2), 14.4 (**C**H_3) ppm, **LRMS** (ESI+), *m/z* (%): 445.3 $[M+Na]^+$ (100), 867.7 $[2M+Na]^+$ (10), 423.4 $[M+H]^+$ (5). **HRMS** (ESI+): calculated for $C_{25}H_{46}O_3N_2Na^+$ ($[M+Na]^+$) 445.3401, found 445.3403. Calculated for $C_{25}H_{47}O_3N_2^+$ ($[M+H]^+$) 423.3581, found 423.3583.

Lyso-M-TCO (22)

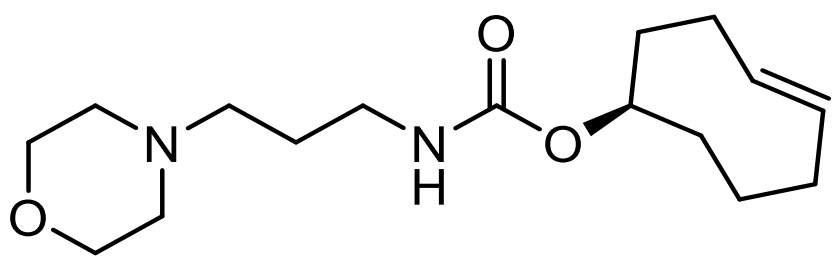

TCO-4-en-NHS ester (20 mg, 75 µmol, 1 equiv.), **4-(3-aminopropyl)morpholine** (22 mg, 0.15 mmol, 2 equiv.), **DIPEA** (65 µL, 0.37 mmol, 5 equiv.), **MeCN** (3 mL). After the extraction, the crude product was purified by reversed-phase chromatography (C18 column, eluent: gradient of H_2O/MeCN) followed by lyophilisation to obtain Lyso-M-TCO (22) as an oily colourless liquid (15 mg, 68%).

^{1}H NMR (400 MHz, C_6D_6): δ = 5.86–5.74 (m, 1H, C**H**CH), 5.57 (bs, 1H, N**H**), 5.50–5.38 (m, 1H, C**H**CH), 5.11 (dd, $^3J_{H-H}$ = 10.5 Hz, $^3J_{H-H}$ = 5.4 Hz, 1H, COOC**H**), 3.58 (t, $^3J_{H-H}$ = 4.7 Hz, 4H, OC**H₂**), 3.17 (q, $^3J_{H-H}$ = 6.1 Hz, 2H, CONHC**H₂**), 2.53–2.39 (m, 1H, C**H₂**), 2.37–2.19 (m, 3H, C**H₂**), 2.11–1.91 (m, 7H, C**H₂**), 1.86–1.65 (m, 2H, C**H₂**), 1.43–1.21 (m, 3H, C**H₂**), 1.02–0.91 (m, 1H, C**H₂**) ppm, **^{13}C NMR** (101 MHz, C_6D_6): δ = 156.0 (**C**O), 135.4 (**C**HCH), 132.2 (**C**HCH), 69.6 (COO**C**H), 67.2 (O**C**H_2), 57.7 (N**C**$H_2CH_2CH_2$), 54.0 (OCH_2**C**H_2), 41.4 (**C**H_2), 41.0 (**C**H_2NH), 34.7 (**C**H_2), 33.0 (**C**H_2), 30.6 (**C**H_2), 28.5 (**C**H_2), 25.8 (**C**H_2) ppm. **LRMS** (ESI+), *m/z* (%): 319.2 $[M+Na]^+$ (100), 297.2 $[M+H]^+$ (10). **HRMS** (ESI+): calculated for $C_{16}H_{28}O_3N_2Na^+$ ($[M+Na]^+$) 319.1992, found 319.1995. Calculated for $C_{16}H_{29}O_3N_2^+$ ($[M+H]^+$) 297.2173, found 297.2175.

Lyso-N-TCO (25)

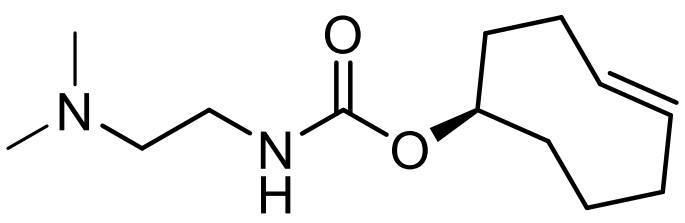

TCO-4-en-NHS ester (203 mg, 0.76 mmol, 1 equiv.), ***N*,*N*-dimethylethylenediamine** (268 mg, 3.04 mmol, 4 equiv.), **DIPEA** (333 µL, 4.57 mmol, 6 equiv.), **MeCN** (3.8 mL). TLC in PE/AcOEt stained with $KMnO_4$ stain and heated showed full conversion in 3 hours. Afterwards, the crude reaction mixture was diluted with water and directly purified by reversed-phase chromatography (C18 column, eluent: gradient of H_2O/MeCN) to obtain **Lyso-N-TCO (25)** (153 mg, 0.64 mmol, 84%) as a colourless oil after lyophilisation.

^{1}H NMR (400 MHz, $CDCl_3$): δ = 5.65–5.47 (m, 2H, C**H**=C**H**^{TCO}), 5.18–5.14 (m, 1H, N**H**), 4.93–4.86 (m, 1H, OC**H**^{TCO}), 3.33–3.23 (m, 2H, C**H₂**NH), 2.42 (t, $^3J_{H-H}$ = 6.0 Hz, 2H, C**H₂**NCH_3), 2.37–2.19 (m, 4H, C**H₂**^{TCO}), 2.25 (s, 6H, C**H₃**), 2.11–2.04 (m, 1H, C**H₂**^{TCO}), 1.90–1.72 (m, 2H, C**H₂**^{TCO}), 1.72–1.47 (m, 2H, C**H₂**^{TCO}), 1.27–1.14 (m, 1H, C**H₂**^{TCO}) ppm, **^{13}C NMR** (101 MHz, CD_3OD): δ = 156.5 (**C**OONH), 135.5 (**C**H=CH^{TCO}), 131.9

(**C**H=CH^{TCO}), 70.2 (O**C**H^{TCO}), 58.5 (**C**H$_2$NCH$_3$), 45.4 (N**C**H$_3$), 41.3 (**C**H$_2$^{TCO}), 38.5 (**C**H$_2$NH), 34.5 (**C**H$_2$^{TCO}), 32.8 (**C**H$_2$^{TCO}), 28.1 (**C**H$_2$^{TCO}) ppm, **LRMS** (ESI+), *m/z* (%): 241.2 [M+H]$^+$ (100), 263,2 [M+Na]$^+$ (20), **HRMS** (ESI+): calculated for $C_{13}H_{25}O_2N_2^+$ ([M+H]$^+$) 241.1911, found 241.1909, calculated for $C_{13}H_{24}O_2N_2Na^+$ ([M+Na]$^+$) 263.1730, found 263.1728.

2.4 Synthesis of the organelle-targeting Tz probes

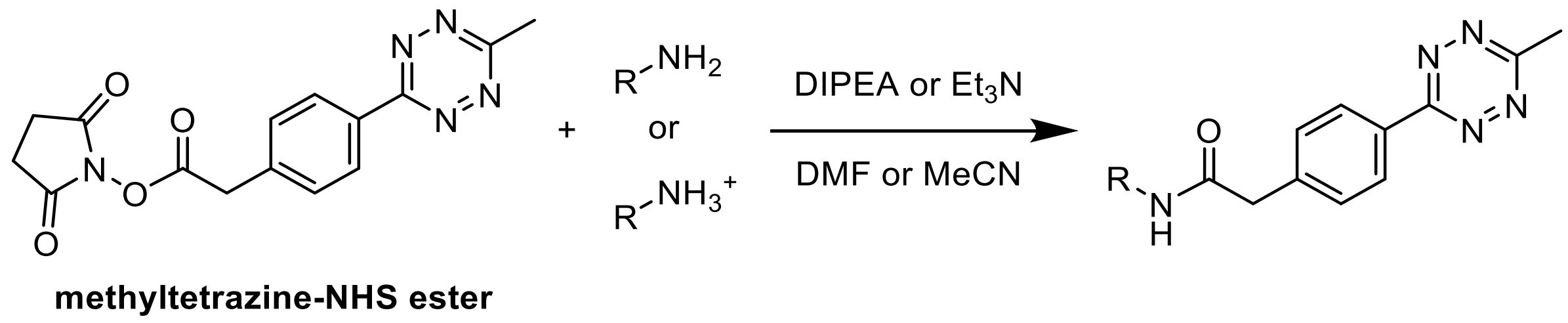

General procedure for the synthesis of the organelle-targeting Tz probes:

Under argon atmosphere, **methyltetrazine-NHS ester** (from Conju-probe, CP-6117) and the corresponding **amine** (or **ammonium salt**) were dissolved in solvent (DMF or MeCN), followed by the addition of base (DIPEA or Et_3N). The reaction mixture was stirred at RT overnight or until TLC or LC-MS analysis showed full conversion. The crude reaction mixture was extracted with DCM, washed with brine, dried over $MgSO_4$, filtered, and evaporated. The crude product was purified by reversed-phase chromatography, flash column chromatography, or preparative HPLC, followed by lyophilisation or evaporation to obtain the desired tetrazine derivative.

PM-Tz (2)

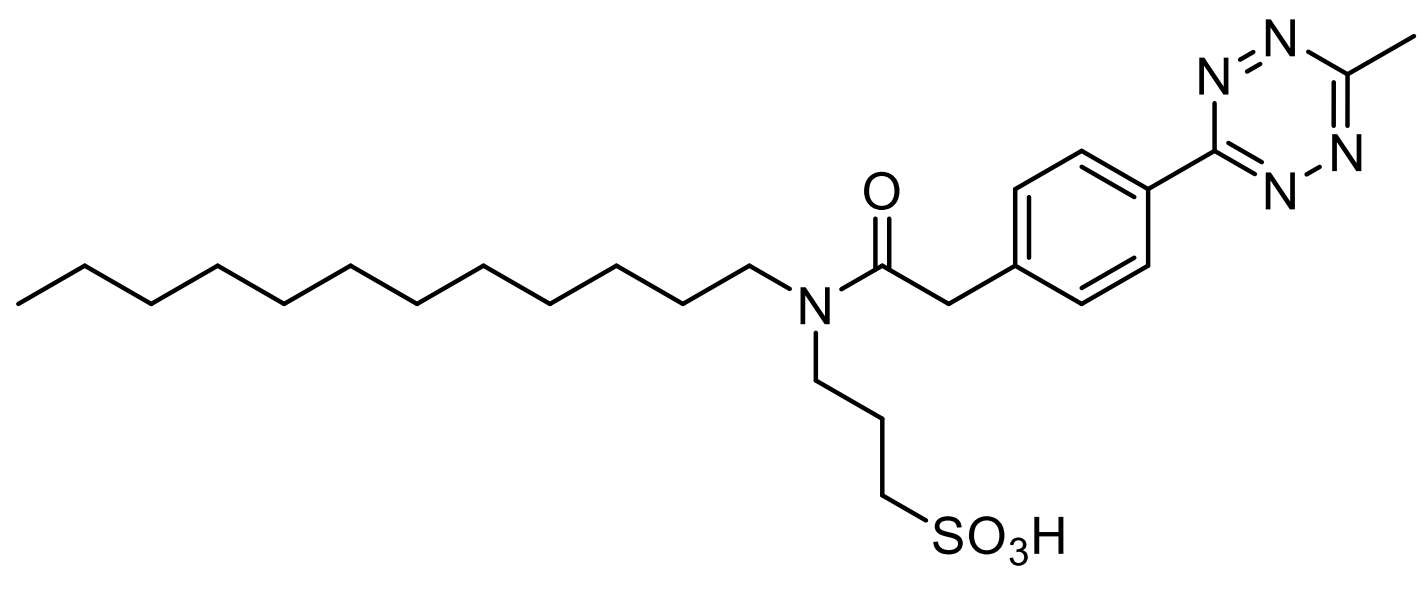

Methyltetrazine-NHS ester (46 mg, 0.14 mmol, 1.2 equiv.), **3-(dodecylamino)propane-1-sulfonic acid (35)** (36 mg, 0.12 mmol, 1 equiv.), **Et_3N** (41 µL, 0.29 mmol, 2.5 equiv.), **MeCN** (2.3 mL). TLC analysis (DCM/MeOH = 7:1 v/v) showed full conversion in 21 h. Before extraction, **AcOH** (179 µL, 2.34 mmol, 20 equiv.) was added to the reaction mixture. After the extraction, the crude product was re-purified by reversed-phase chromatography (C18 column, eluent: gradient of H_2O/MeCN + 0.1% TFA) followed by lyophilisation to obtain partially purified **PM-Tz (2)**. Residual **3-(dodecylamino)propane-1-sulfonate**

was separated from the desired product by flash column chromatography in Hilic mode (silica gel, eluent: gradient of MeCN/H_2O) followed by lyophilisation to obtain pure **PM-Tz (2)** as a pink solid (31 mg, 51%). *In the NMR spectra (both ^1H and ^{13}C), signals of two rotamers of the amide bond were observed. The presence of these rotamers was confirmed by NMR at 55 °C.*

^{1}H NMR (400 MHz, CD_3OD): δ = 8.54–8.47 (m, 2H, TzCC**H**), 7.60–7.49 (m, 2H, TzCCHC**H**), 3.95 (d, J = 13.6 Hz, 2H, PhC**H₂**), 3.65–3.58 (m, 1H, C**H₂**), 3.54 (m, 1H, C**H₂**), 3.42 (m, 2H, C**H₂**), 3.03 (s, 3H, C**H₃**Tz), 2.86 (t, J = 6.9 Hz, 2H, C**H₂**SO₃H), 2.13–2.01 (m, 2H, C**H₂**CH₂SO₃H), 1.63–1.47 (m, 2H, CH₂), 1.35–1.17 (m, 18H, 9×C**H₂**), 0.95–0.84 (m, 3H, CH₂C**H₃**). **^{13}C NMR** (101 MHz, CD_3OD): δ [173.1, 173.0 (**C**ON)], [168.7, 168.7 (CH₃**C**NN)], [165.2, 165.2 (NN**C**Ph)] , [141.6, 141.6 (**C**CH₂CON)], [132.9, 132.1 (Tz**C**)], [131.1, 130.9 TzCCH**C**H], [129.1, 129.0 TzC**C**H], 50.0 (**C**H₂), 48.4 (**C**H₂), 47.2 (**C**H₂), 46.3 (**C**H₂), [41.2, 40.9 (Ph**C**H₂)], 33.0 (**C**H₂), 30.8 (**C**H₂), 30.7 (**C**H₂), 30.7 (**C**H₂), 30.6 (**C**H₂), 30.5 (**C**H₂), 30.5 (**C**H₂), 30.4 (**C**H₂), 29.9 (**C**H₂), 28.6 (**C**H₂), 28.0 (**C**H₂), 27.8 (**C**H₂), 25.5 (**C**H₂), 24.3 (**C**H₂), 23.7 (**C**H₂), [21.1, 21.0 (**C**H₃Tz)], 14.4 (CH₂**C**H₃). **HRMS** (ESI–): calculated for $C_{26}H_{40}O_4N_5S^-$ ([M–H]$^-$) 518.2807, found 518.2804.

Mito-Tz (5)

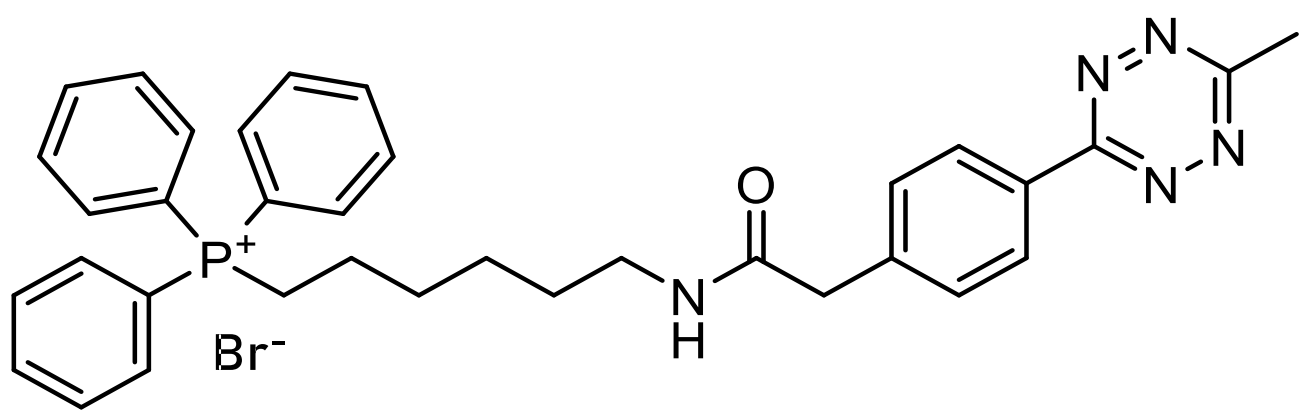

Methyltetrazine-NHS ester (25 mg, 76 µmol, 1.0 equiv.), **6-bromohexyl(triphenyl)phosphanium bromide** (41 mg, 92 µmol, 1.2 equiv.), **DIPEA** (47 µL, 0.27 mmol, 3.5 equiv.), **MeCN** (3 mL). After the extraction, the crude product was purified by preparative TLC (eluent: DCM/MeOH). **Mito-Tz (5)** was obtained as a pink powder (16 mg, 32%).

^{1}H NMR (400 MHz, $CDCl_3$): δ = 8.39–8.29 (m, 3H, C**H**CCNN, N**H**), 7.87–7.75 (m, 9H, PCC**H**CHC**H**), 7.75–7.64 (m, 8H, PCCHC**H**, CH₂CC**H**), 3.87 (s, 2H, C**H₂**CONH), 3.71–3.57 (m, 2H, C**H₂**P), 3.32–3.23 (m, 2H, C**H₂**NH), 3.05 (s, 3H, C**H₃**), 1.74–1.14 (m, 8H, C**H₂**) ppm, **^{13}C NMR** (101 MHz, $CDCl_3$): δ = 171.5 (**C**O), 167.0 (**C**CH₃), 164.3 (CH₂C**C**NN), 142.3 (CH₂**C**), 135.2 (d, $J_{C\text{-}P}$ = 3 Hz, PCCHCH**C**H), 133.8 (d, $J_{C\text{-}P}$ = 10 Hz, PC**C**H), 130.7 (CH₂C**C**H), 130.6 (d, $J_{C\text{-}P}$ = 13 Hz, PCCH**C**H), 129.7 (CH**C**CNN), 127.9 (**C**HCCNN), 118.4 (d, $J_{C\text{-}P}$ = 86 Hz, P**C**CH), 43.3 (**C**H₂CONH), 38.8 (CONH**C**H₂), 29.8 (**C**H₂), 29.3 (d, $J_{C\text{-}P}$ = 16 Hz, **C**H₂), 28.3 (**C**H₂), 22.5 (d, $J_{C\text{-}P}$ = 51 Hz, **C**H₂), 22.3 (d, $J_{C\text{-}P}$ = 4 Hz, **C**H₂), 21.2 (**C**H₃) ppm. **LRMS** (ESI+), *m/z* (%): 574.3 [M]$^+$ (100). **HRMS** (ESI+): calculated for $C_{35}H_{37}ON_5P^+$ ([M]$^+$) 574.2730, found 574.2727.

Nucl-Tz (8)

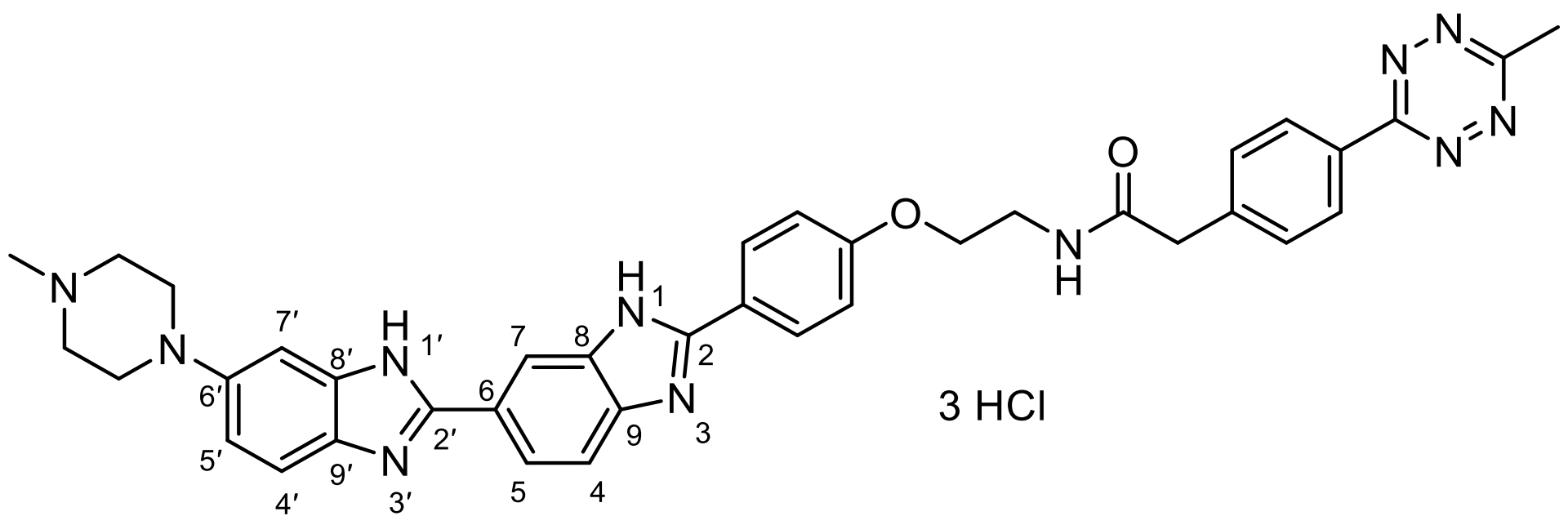

Methyltetrazine-NHS ester (11.5 mg, 0.34 mmol, 1 equiv.), **Hoechst 33258 *O*-ethylamine 37** (20 mg, 0.34 mmol, 1 equiv.), **Et_3N** (10 µL, 0.68 mmol, 2 equiv.), dry **DMF** (6 mL). TLC analysis (DCM/MeOH + 1% Et_3N) showed full conversion in 80 min. After the extraction, the crude product was purified by reversed-phase chromatography (C18 column, eluent: gradient of H_2O/MeCN) followed by lyophilisation and ion exchange on DOWEX (50W-X8) to obtain **Nucl-Tz (8)** in the form of trihydrochloride and as an orange powder (13 mg, 56%).

^{1}H NMR (400 MHz, DMSO-d_6): δ = 8.49 (t, J = 5.6 Hz, 1H, N**H**$COCH_2$), 8.41–8.35 (m, 2H, $C_{ipso}(CHC\mathbf{H})_2C_{ipso}Tz$), 8.28 (s, 1H, **H**-7), 8.18–8.09 (m, 2H, $C_{ipso}(C\mathbf{H}CH)_2C_{ipso}O$), 8.00 (dd, J = 8.4, 1.6 Hz, 1H, **H**-5), 7.66 (d, J = 8.2 Hz, 1H, H-4), 7.58–7.52 (m, 2H, $C_{ipso}(C\mathbf{H}CH)_2C_{ipso}Tz$), 7.44 (d, J = 8.7 Hz, 1H, H-4’), 7.17–7.10 (m, 2H, $C_{ipso}(CHC\mathbf{H})_2C_{ipso}O$), 7.01 (s, 1H, H-7’), 6.93 (dd, J = 8.8, 2.3 Hz, 1H, H-5’), 4.13 (t, J = 5.5 Hz, 2H, $OC\mathbf{H_2}CH_2NH$), 3.61 (s, 2H, $NHCOC\mathbf{H_2}$), 3.54–3.48 (m, 2H, $OCH_2C\mathbf{H_2}NH$), 3.13 (t, J = 5.0 Hz, 4H, $CH_3N(CH_2C\mathbf{H_2})_2N$), 2.95 (s, 3H, $C\mathbf{H_3}Tz$), 2.53–2.50 (m, 4H, $CH_3N(C\mathbf{H_2}CH_2)_2N$), 2.25 (s, 3H, $C\mathbf{H_3}N$). **^{13}C NMR** (101 MHz, DMSO-d_6): δ = 170.4 (NH**C**OCH_2), 167.5 (CH_3**C**-Tz), 163.7 (**C**-Tz), 160.5 ($C_{ipso}(CHCH)_2\mathbf{C}_{ipso}O$), 153.2 (**C**-2), 148.1 (**C**-6’), 141.6 ($\mathbf{C}_{ipso}(CHCH)_2C_{ipso}Tz$), 130.5 ($C_{ipso}(\mathbf{C}HCH)_2C_{ipso}Tz$), 130.5 ($C_{ipso}(CHCH)_2\mathbf{C}_{ipso}Tz$), 128.7 ($C_{ipso}(\mathbf{C}HCH)_2C_{ipso}O$), 127.8 ($C_{ipso}(CH\mathbf{C}H)_2C_{ipso}Tz$), 126.7 (**C**H-imidazol), 124.9 (**C**-imidazol), 123.0 ($\mathbf{C}_{ipso}(CHCH)_2C_{ipso}O$), 121.2 (**C**-5), 115.4 ($C_{ipso}(CH\mathbf{C}H)_2C_{ipso}O$), 114.1 (**C**-5’), 67.0 ($O\mathbf{C}H_2CH_2NH$), 55.3 ($CH_3N(\mathbf{C}H_2CH_2)_2N$), 50.4 ($CH_3N(CH_2\mathbf{C}H_2)_2N$), 46.2 ($\mathbf{C}H_3N$), 42.7 ($NHCO\mathbf{C}H_2$), 21.3 ($\mathbf{C}H_3Tz$). **HRMS** (ESI−): calculated for $C_{38}H_{38}O_2N_{11}^-$ ($[M-H]^-$) 680.3204, found 608.3210.

ER-F-Tz (10)

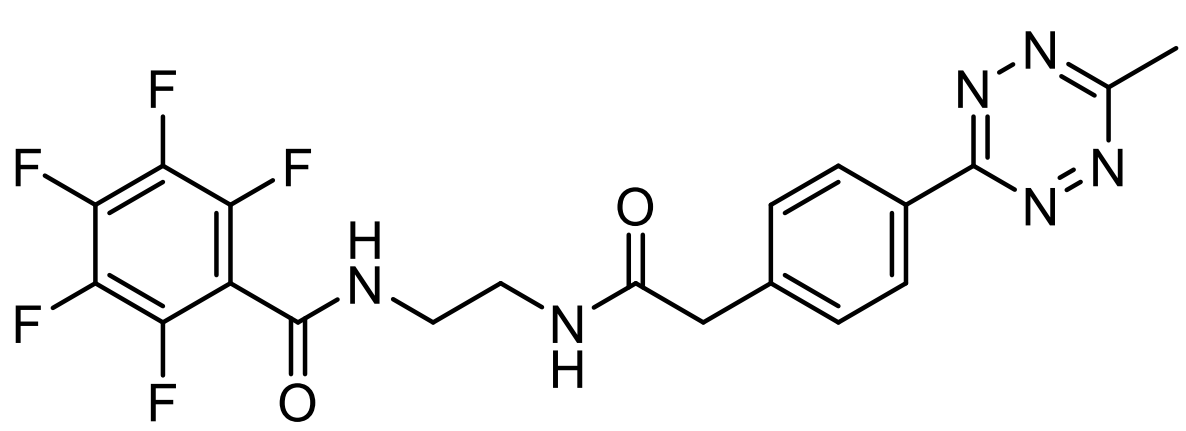

Methyltetrazine-NHS ester (50 mg, 0.15 mmol, 1 equiv.), **2-(perfluorobenzamido)ethan-1-aminium 2,2,2-trifluoroacetate (39)** (58 mg, 0.23 mmol, 1.5 equiv.), **DIPEA** (132 µL, 0.76 mmol, 5 equiv.), **DMF**

(2 mL). After extraction, the crude product was purified by column chromatography (silica gel, eluent: DCM/MeOH 19:1) followed by evaporation to obtain **ER-F-Tz (10)** (37 mg, 53%) as a pink solid.

^{1}H NMR (401 MHz, $CDCl_3$): δ = 8.42 (d, J = 8.0 Hz, 2H), 7.41 (d, J = 8.0 Hz, 2H), 3.55 (s, 2H), 3.42 (s, 2H), 3.33 (s, 2H), 3.00 (s, 3H). **^{19}F NMR** (376 MHz, $CDCl_3$): δ −135.4–−139.5 (m), −146.1–−150.9 (m), −154.8–−159.2 (m). **^{13}C NMR** (101 MHz, $CDCl_3$): δ = 172.3, 167.2, 163.9, 158.8, 139.8, 130.6, 130.2, 128.2, 43.1, 39.8, 39.0, 20.9. **LRMS** (ESI+), *m/z* (%): 489.1 $[M+Na]^+$ (100), 955.2 $[2M+Na]^+$ (15), 467.1 $[M+H]^+$ (5). **HRMS** (ESI+): calculated for $C_{20}H_{16}O_2N_6F_5^+$ ($[M+H]^+$) 467.1249, found 467.1248, calculated for $C_{20}H_{15}O_2N_6F_5Na^+$ ($[M+Na]^+$) 489.1069, found 489.1064.

ER-Cl-Tz (12)

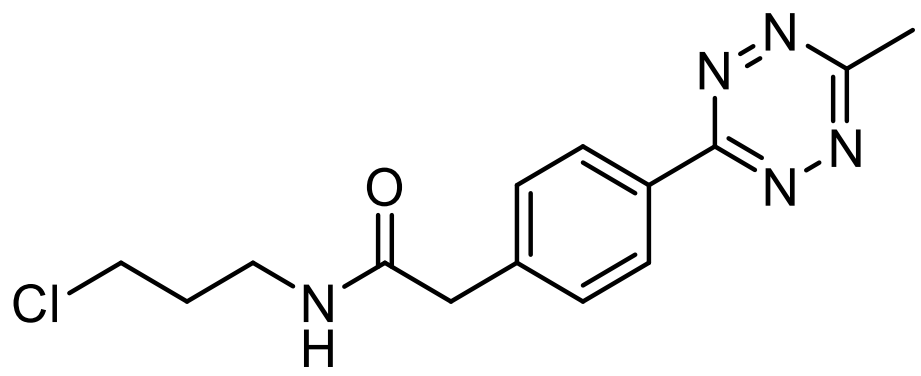

Methyltetrazine-NHS ester (33 mg, 0.10 mmol, 1 equiv.), **3-chloropropylamine hydrochloride** (13 mg, 0.10 mmol, 1 equiv.), **DIPEA** (87 µL, 0.50 mmol, 5 equiv.), **DMF** (3 mL). After the extraction, the crude product was purified by reversed-phase chromatography (C18 column, eluent: gradient of H_2O/MeCN) followed by lyophilization to obtain **ER-Cl-Tz (12)** as a pink fine powder (14 mg, 46%).

^{1}H NMR (400 MHz, DMSO-d_6): δ = 8.40 (d, $^3J_{H-H}$ = 8.3 Hz, 2H, C**H**^{Ar}), 8.24 (t, $^3J_{H-H}$ = 5.7 Hz, 1H, N**H**), 7.54 (d, $^3J_{H-H}$ = 8.3 Hz, 2H, C**H**^{Ar}), 3.64 (t, $^3J_{H-H}$ = 6.5 Hz, 2H, C**H₂**Cl), 3.55 (s, 2H, COC**H₂**), 3.20 (td, $^3J_{H-H}$ = 6.5 Hz, $^3J_{H-H}$ = 5.9 Hz, NHC**H₂**), 2.99 (s, 3H, C**H₃**), 1.87 (p, 2H, $^3J_{H-H}$ = 6.6 Hz, CH_2C**H₂**CH_2) ppm, **^{13}C NMR** (101 MHz, DMSO-d_6): δ = 169.7 (**C**O), 167.1 (**C**CH_3), 163.3 (CHC**C**NN), 141.3 (**C**H_2CO), 130.1 (CH_2C**C**H), 130.1 (CH**C**CNN), 127.4 (**C**HCCNN), 43.0 (**C**H_2Cl), 42.3 (**C**H_2CO), 36.2 (**C**H_2NH), 32.1 (CH_2**C**H_2CH_2), 20.9 (CH_3) ppm, **LRMS** (ESI+), *m/z* (%): 328.1 $[M+Na]^+$ (100), 344.1 $[M+K]^+$ (65), 306.1 $[M+H]^+$ (5). **HRMS** (ESI+): calculated for $C_{14}H_{16}ON_5ClNa^+$ ($[M+Na]^+$) 328.0936, found 328.0932, calculated for $C_{14}H_{16}ON_5ClK^+$ ($[M+K]^+$) 344.0675, found 344.0671, calculated for $C_{14}H_{17}ON_5Cl^+$ ($[M+H]^+$) 306.1116, found 306.1113.

ER-SU1-Tz (15)

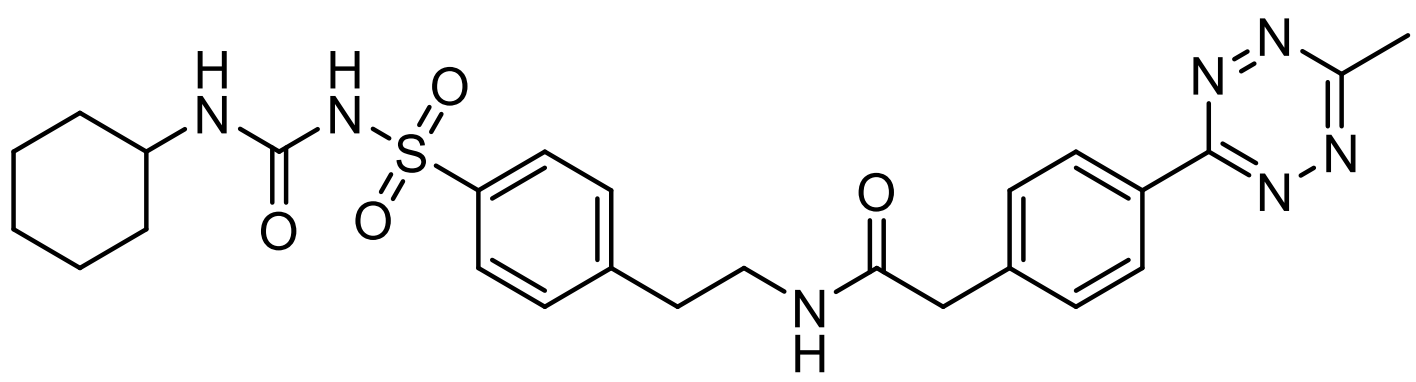

Methyltetrazine-NHS ester (50 mg, 0.15 mmol, 1 equiv.), **4-(2-aminoethyl)-N-(cyclohexylcarbamoyl)benzenesulfonamide, 2,2,2-trifluoroacetate salt (41)** (59 mg, 0.18 mmol, 1.2 equiv.), **DIPEA** (132 µL, 0.76 mmol, 5 equiv.), **DMF** (2 mL). After the extraction, the crude product was

purified by column chromatography (silica gel, eluent: DCM/MeOH 19:1) followed by evaporation to obtain **ER-SU1-Tz (15)** (30 mg, 37%) as a pink solid.

^{1}H NMR (400 MHz, $CDCl_3$/MeOD): δ = 8.38 (d, J = 8.0 Hz, 2H), 7.65 (d, J = 8.0 Hz, 2H), 7.33 (d, J = 8.0 Hz, 2H), 7.13 (d, J = 8.0 Hz, 2H), 3.45 (s, 2H), 3.27–3.18 (m, 2H), 2.97 (s, 3H), 2.74 (t, J = 6.9 Hz, 2H), 1.83–1.36 (m, 9H), 1.27–0.89 (m, 13H). **^{13}C NMR** (101 MHz, $CDCl_3$/MeOD): δ = 171.3, 167.2, 163.9, 151.2, 145.1, 140.1, 137.9, 130.5, 130.0, 129.3, 128.1, 127.3, 43.1, 40.2, 35.1, 33.6, 32.7, 25.5, 25.2, 24.8, 24.4, 20.8. **LRMS** (ESI+), *m/z* (%): 560.2 $[M+Na]^+$ (100), 1097.4 $[2M+Na]^+$ (30), 538.2 $[M+H]^+$ (5). **HRMS** (ESI+): calculated for $C_{26}H_{32}O_4N_7S^+$ ($[M+H]^+$) 538.2231, found 538.2229, calculated for $C_{26}H_{31}O_4N_7NaS^+$ ($[M+Na]^+$) 560.2050, found 560.2048.

ER-SU2-Tz (18)

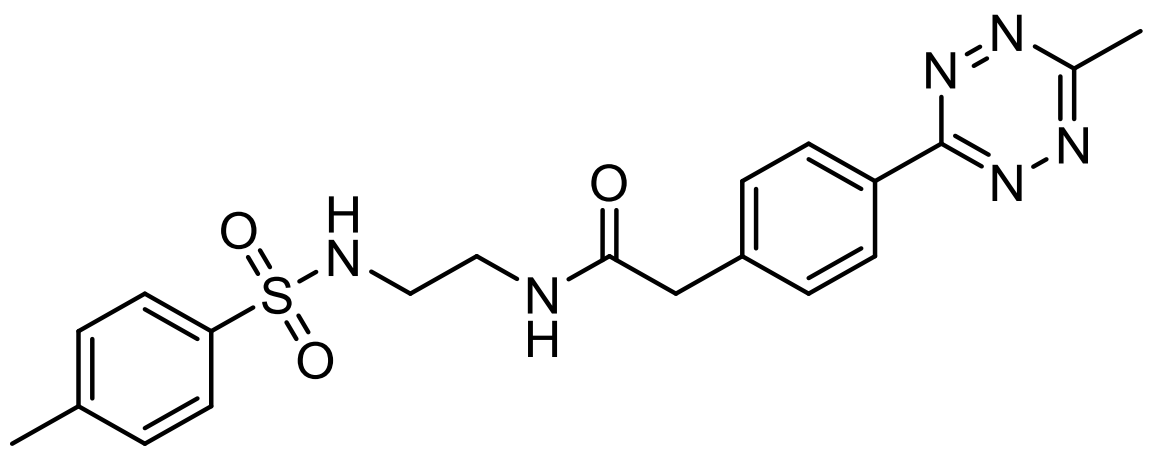

Methyltetrazine-NHS ester (50 mg, 0.15 mmol, 1 equiv.), **N-(2-aminoethyl)-4-methylbenzenesulfonamide (42)** (39 mg, 0.18 mmol, 1.2 equiv.), **DIPEA** (132 µL, 0.76 mmol, 5 equiv.), **DMF** (2 mL). After the extraction, the crude product was purified by column chromatography (silica gel, eluent: DCM/MeOH 19:1) followed by evaporation to obtain **ER-SU2-Tz (18)** (67 mg, 99%) as a pink solid.

^{1}H NMR (400 MHz, $CDCl_3$/MeOD): δ = 8.50 (d, J = 8.3 Hz, 2H), 7.66 (d, J = 8.3 Hz, 2H), 7.46 (d, J = 8.3 Hz, 2H), 7.26 (d, J = 8.3 Hz, 2H), 3.60 (s, 2H), 3.29 (tt, J = 5.9, 3.0 Hz, 2H), 3.06 (s, 3H), 2.97 (dd, J = 6.5, 4.8 Hz, 2H), 2.38 (s, 3H). **^{13}C NMR** (101 MHz, $CDCl_3$/MeOD): δ = 171.6, 167.3, 164.0, 143.7, 139.8, 136.8, 130.7, 130.3, 129.9, 128.4, 127.0, 43.3, 42.5, 39.5, 21.6, 21.2. **LRMS** (ESI+), *m/z* (%): 449.1 $[M+Na]^+$ (100), 875.3 $[2M+Na]^+$ (20), 427.2 $[M+H]^+$ (5). **HRMS** (ESI+): calculated for $C_{20}H_{23}O_3N_6S^+$ ($[M+H]^+$) 427.1547, found 427.1545. Calculated for $C_{20}H_{22}O_3N_6NaS^+$ ($[M+Na]^+$) 449.1366, found 449.1363.

Golgi-Tz (20)

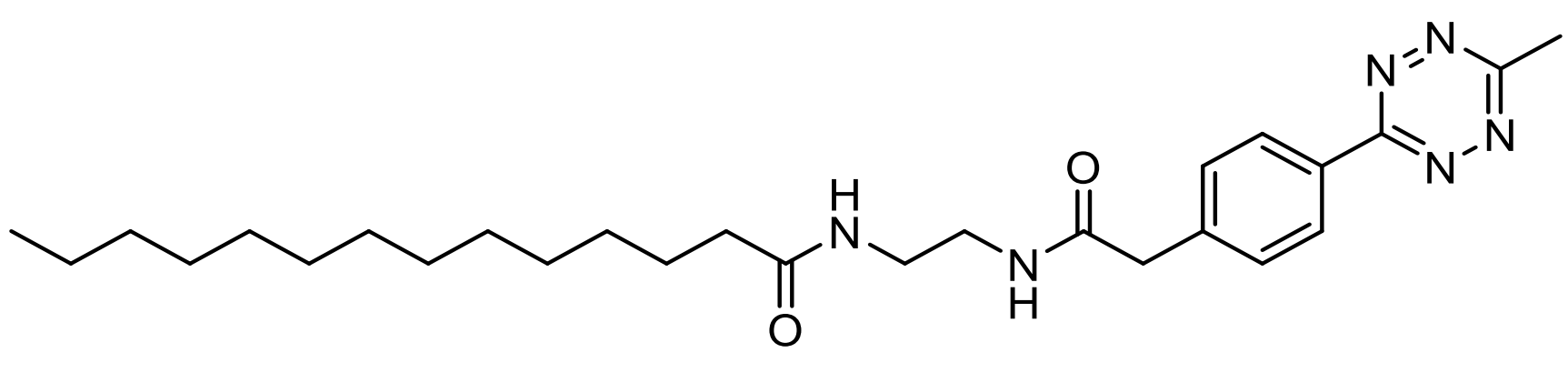

Methyltetrazine-NHS ester (32 mg, 97 µmol, 1.2 equiv.), **2-tetradecanamidoethan-1-aminium 2,2,2-trifluoroacetate (43)** (31 mg, 81 µmol, 1 equiv.), **DIPEA** (140 µL, 0.81 mmol, 10 equiv.), **DMF** (3 mL). After the extraction, the crude product was purified by reversed-phase chromatography (C18 column,

eluent: gradient of H_2O/MeCN) followed by lyophilization to obtain **Golgi-Tz (20)** as a pink fine powder (19 mg, 49%).

^{1}H NMR (400 MHz, $CDCl_3$): δ = 8.58 (d, $^3J_{H-H}$ = 8.3 Hz, 2H, CCC**H**), 7.51 (d, $^3J_{H-H}$ = 8.3 Hz, 2H, CH_2CC**H**), 6.32 (s, 1H, N**H**), 5.97 (s, 1H, N**H**), 3.65 (s, 2H, C**H₂**CCH), 3.41–3.35 (m, 4H, NHC**H₂**), 3.10 (s, 3H, CC**H₃**), 2.13 (t, $^3J_{H-H}$ = 7.7 Hz, 2H, COC**H₂**CH_2), 1.72–1.50 (m, 4H, C**H₂**), 1.41–1.16 (m, 20H, C**H₂**), 0.92–0.80 (m, 3H, CH_2C**H₃**) ppm, **^{13}C NMR** (101 MHz, DMSO-d_6, *quaternary carbons were not observed due to the low concentration of the sample caused by poor solubility*): δ = 130.4 (CC**C**H), 128.6 (CH_2C**C**H), 43.8 (**C**H_2CCH), 41.1 (**C**H_2), 39.9 (**C**H_2), 36.8 (**C**H_2), 32.1 (CO**C**H_2CH_2), 29.9–29.4 (m, **C**H_2), 25.8 (**C**H_2), 22.8 (**C**H_2), 21.3 (C**C**H_3), 14.3 (CH_2**C**H_3) ppm, **LRMS** (ESI+), *m/z* (%): 505.3 $[M+Na]^+$ (100), 348.3 $[M+H]^+$ (30), 987.7 $[2M+Na]^+$ (20). LRMS (ESI−), *m/z* (%): 481.3 $[M+H]^-$ (100), 517.3 $[M+Cl]^-$ (15), 527.3 $[M+FA-H]^-$ (10), 595.3 $[M+TFA-H]^-$ (10). **HRMS** (ESI+): calculated for $C_{25}H_{40}ON_9Na^+$ ($[M+Na]^+$) 505.3248, found 505.3251, calculated for $C_{25}H_{41}ON_9^+$ ($[M+H]^+$) 483.3429, found 483.3432. **HRMS** (ESI−): calculated for $C_{27}H_{41}O_2N_6^-$ ($[M-H]^-$) 481.3297, found 481.3297.

Lyso-M-Tz (23)

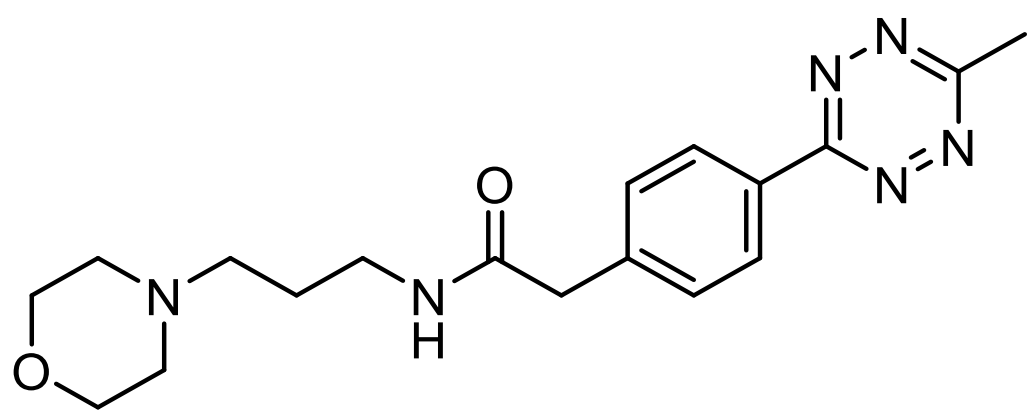

Methyltetrazine-NHS ester (33 mg, 0.10 mmol, 1 equiv.), **4-(3-aminopropyl)morpholine** (14 mg, 0.10 mmol, 1 equiv.), **DIPEA** (87 µL, 0.50 mmol, 5 equiv.), **DMF** (3 mL). After the extraction, the crude product was purified by reversed-phase chromatography (C18 column, eluent: gradient of H_2O/MeCN) followed by lyophilisation to obtain **Lyso-M-Tz (23)** as a pink fine powder (25 mg, 25%).

^{1}H NMR (400 MHz, DMSO-d_6): δ = 8.40 (d, $^3J_{H-H}$ = 8.3 Hz, 2H, C**H**^{Ar}), 8.15 (t, $^3J_{H-H}$ = 5.3 Hz, 1H, N**H**), 7.53 (d, $^3J_{H-H}$ = 8.3 Hz, 2H, C**H**^{Ar}), 3.58–3.52 (m, 6H, OC**H₂**, C**H₂**CONH), 3.09 (td, $^3J_{H-H}$ = 6.7 Hz, $^3J_{H-H}$ = 5.9 Hz), 2.99 (s, 3H, C**H₃**), 2.40–2.20 (m, 6H, NC**H₂**, NHC**H₂**), 1.57 (p, $^3J_{H-H}$ = 7.1 Hz, CH_2C**H₂**CH_2) ppm, **^{13}C NMR** (101 MHz, DMSO-d_6): δ = 169.4 (**C**ONH), 167.1 (**C**CH_3), 163.2 (CHC**C**NN), 141.3 (**C**CH_2), 130.1 (CH_2C**C**H), 130.0 (CH**C**CNN), 127.3 (**C**HCCNN), 66.1 (O**C**H_2), 55.7 (N**C**$H_2CH_2CH_2$), 53.3 (N**C**H_2CH_2O), 42.4 (**C**CH_2), 37.0 (NH**C**H_2), 25.9 (CH_2**C**H_2CH_2), 20.8 (**C**H_3) ppm. **LRMS** (ESI+), *m/z* (%): 357.2 $[M+H]^+$ (100), 379.2 $[M+Na]^+$ (20), 395.2 $[M+K]^+$ (15), 735.4 $[2M+Na]^+$ (5). **HRMS** (ESI+): calculated for $C_{18}H_{25}O_2N_6^+$ ($[M+H]^+$) 357.2034, found 357.2030, calculated for $C_{18}H_{24}O_2N_6Na^+$ ($[M+Na]^+$) 379.1853, found 379.1851.

2.5 Synthesis of the organelle-targeting coumarin probes

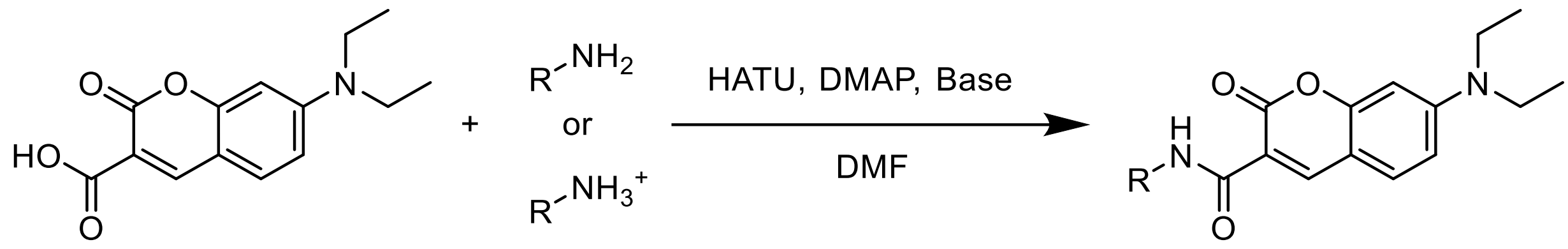

General procedure for the synthesis of the organelle-targeting coumarin probes:

Under argon atmosphere, **7-(diethylamino)coumarin-3-carboxylic acid** was dissolved in dry **DMF**, followed by the addition of **DMAP**, **DIPEA** or **Et_3N** and **HATU**. The reaction mixture was stirred in the dark for 30 min. Afterwards, the corresponding **amine** (or **ammonium salt**) was added. The reaction mixture was stirred at RT overnight or until TLC or LC-MS analysis showed full conversion. The crude reaction mixture was extracted with DCM, washed with brine, dried over $MgSO_4$ or Na_2SO_4, filtered and evaporated. The crude product was purified by reversed-phase chromatography, flash column chromatography, or preparative HPLC, followed by lyophilization or evaporation to obtain desired coumarin derivative.

PM-Coum (3)

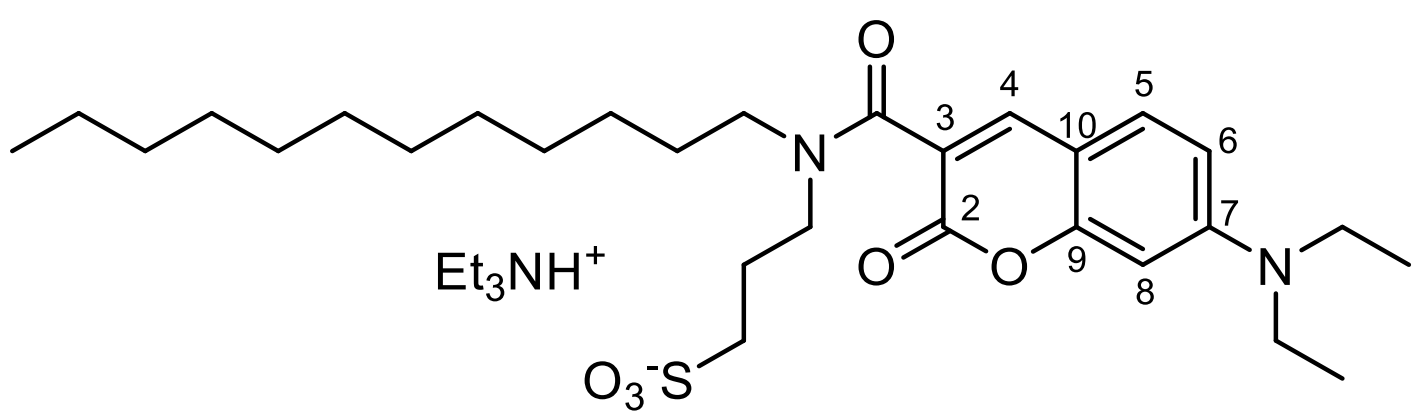

7-(diethylamino)coumarin-3-carboxylic acid (20 mg, 0.08 mmol, 1 equiv.), **HATU** (32 mg, 0.08 mmol, 1.1 equiv.), **Et_3N** (43 µL, 0.31 mmol, 4 equiv.), **3-(dodecylamino)propane-1-sulfonic acid (35)** (26 mg, 0.08 mmol, 1.1 equiv.), dry **DMF** (2 mL). TLC analysis (DCM/MeOH) showed full conversion in 2 h. The crude product was purified directly, without extraction, by flash column chromatography (silica gel, eluent: gradient of MeCN/H_2O) to obtain **PM-Coum (3)** as a yellow oil (50 mg, quantitative).

In the NMR spectra (both ^1H and ^{13}C), signals of two rotamers of the amide bond were observed. The presence of these rotamers was confirmed by NMR at 55 °C.

^{1}H NMR (400 MHz, CD_3OD): δ = 7.86 (d, J = 3.9 Hz, 1H, **H**-4), 7.46 (d, J = 8.9 Hz, 1H, **H**-5), 6.76 (ddd, J = 8.9, 3.9, 2.4 Hz, 1H, **H**-6), 6.57–6.54 (m, 1H, **H**-8), 3.64 (t, J = 7.3 Hz, 1H, C**H_2**), 3.50 (q, J = 7.0 Hz, 4H, NC**H_2**CH_3), 3.48–3.42 (m, 2H, C**H_2**), 3.36–3.31 (m, 1H, C**H_2**), 3.19 (q, J = 7.3 Hz, 6H, NC**H_2**CH_3), 2.95–2.88 (m, 1H, C**H_2**), 2.73–2.67 (m, 1H, C**H_2**), 2.19–2.11 (m, 1H, C**H_2**), 2.09–2.00 (m, 1H, C**H_2**), 1.73–1.56 (m, 2H, C**H_2**), 1.42–1.11 (m, 33H, NCH_2C**H_3** 9×C**H_2**), 0.92–0.86 (m, 3H, C**H_3**). **^{13}C NMR** (101 MHz, CD_3OD): δ [168.91, 168.84 (**C**-2)], [161.27, 161.18 **C**-2], [158.39 158.35 (**C**-9)], [153.32, 153.22 (**C**-7)] [144.73, 144.61 (**C**-4)], [131.34, 131.20 (**C**-5)], [117.66, 117.63 (**C**-3)], [111.01, 110.93 (**C**-6)], [108.94, 108.78 (**C**-10)], 97.77 (**C**-

8), 50.22 (CH_2), 50.07 (CH_2), 49.58 (CH_2), 49.14 (CH_2), 47.88 (CH_2), 47.83 (CH_2), 46.27 (CH_2), 45.80 ($N(CH_2CH_3)_2$), 45.33 (CH_2), 33.09 (CH_2), 30.77 (CH_2), 30.67 (CH_2), 30.64 (CH_2), 30.56 (CH_2), 30.44 (CH_2), 29.99 (CH_2), 29.35 (CH_2), 28.36 (CH_2), 27.97 (CH_2), 27.52 (CH_2), 27.32 (CH_2), 25.41 (CH_2), 24.29 (CH_2), 23.74 (CH_2), 23.01 (CH_2), [14.48, 14.46 (CH_3)], [12.73, 12.71 ($N(CH_2CH_3)_2$)], 9.28 ($N(CH_2CH_3)_3$). **HRMS** (ESI−): calculated for $C_{29}H_{45}O_6N_2S$ ($[M-H]^-$) 549.3004, found 549.3006.

Mito-Coum (6)

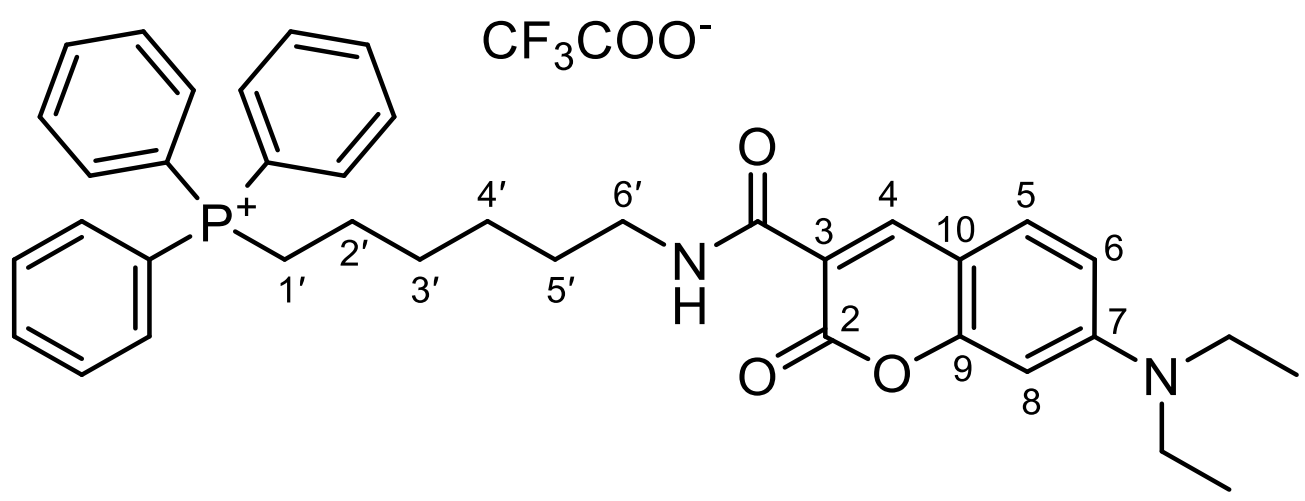

7-(diethylamino)coumarin-3-carboxylic acid (20 mg, 0.08 mmol, 1 equiv.), **HATU** (32 mg, 0.08 mmol, 1.1 equiv.), **Et_3N** (43 µL, 0.31 mmol, 4 equiv.), **(6-Aminohexyl)triphenylphosphonium bromide hydrobromide** (37 mg, 0.08 mmol, 1.1 equiv.), dry **DMF** (1.5 mL). TLC analysis (DCM/MeOH) showed full conversion in 1 h. After extraction, the crude product was purified by reversed-phase chromatography (C18 column; eluent: gradient of H_2O/MeCN) followed by lyophilisation to obtain **Golgi-Coum (21)** as a yellow powder (5 mg, 21%). The crude product was purified by reversed-phase chromatography (C18 column, eluent: gradient of H_2O/MeCN) followed by lyophilisation to obtain a partially purified product. It was then redissolved in DCM with 0.02% TFA, evaporated and repurified by preparative HPLC (C18 column, eluent: gradient of H_2O/MeCN with 0.1% TFA), followed by lyophilisation to obtain **Mito-Coum (6)** as a yellow powder (27 mg, 49%).

^{1}H NMR (400 MHz, $CDCl_3$): δ = 8.75 (t, J = 5.8 Hz, 1H, N**H**), 8.63 (s, 1H, **H**-4), 7.84–7.76 (m, 3H, Ph-H_p), 7.74–7.62 (m, 12H, Ph-$H_{o,m}$), 7.39 (d, J = 9.0 Hz, 1H, **H**-5), 6.64 (dd, J = 9.0, 2.5 Hz, 1H, **H**-6), 6.47 (d, J = 2.4 Hz, 1H, **H**-8), 3.44 (q, J = 7.1 Hz, 4H, NCH_2CH_3), 3.36 (q, J = 6.6 Hz, 2H, **H**-6’), 3.32–3.21 (m, 2H, **H**-1’), 1.70–1.56 (m, 4H, H-2’, **H**-3’), 1.52 (p, J = 7.1 Hz, 2H, **H**-5’), 1.43–1.31 (m, 2H, **H**-3’), 1.22 (t, J = 7.1 Hz, 6H, NCH_2CH_3). **^{19}F NMR** (376 MHz, $CDCl_3$): δ −75.48 (CF_3COO). **^{13}C NMR** (101 MHz, $CDCl_3$): δ = 163.3 (**C**ONH), 162.9 (**C**OO), 160. 5 (q, J = 36.6 Hz, CF_3COO) $^-$, 157.7 (**C**-9), 152.7 (**C**-7), 148.1 (**C**-4), 135.4 (d, J = 3.1 Hz, Ph-C_p), 133.5 (d, J = 9.9 Hz, Ph-C_o), 131.2 (**C**-5), 130.7 (d, J = 12.5 Hz, Ph-C_m), 118.5 (d, J = 86.2 Hz, Ph-C_{ipso}), 110.2 (**C**-3), 110.2 (**C**-6), 108.4 (**C**-10), 96.6 (**C**-8), 45.2 (NCH_2CH_3), 39.3 (**C**-6’), 30.1 (d, J = 16.0 Hz, **C**-3’), 29.2 (**C**-5’), 26.3 (**C**-4’), 22.4 (d, J = 4.5 Hz, **C**-2’), 22.4 (d, J = 51.3 Hz, **C**-1’), 12.5 (NCH_2CH_3). **HRMS** (ESI+): calculated for $C_{38}H_{42}O_3N_2P^+$ ($[M+H]^+$) 605.2928, found 605.2920.

ER-Cl-Coum (13)

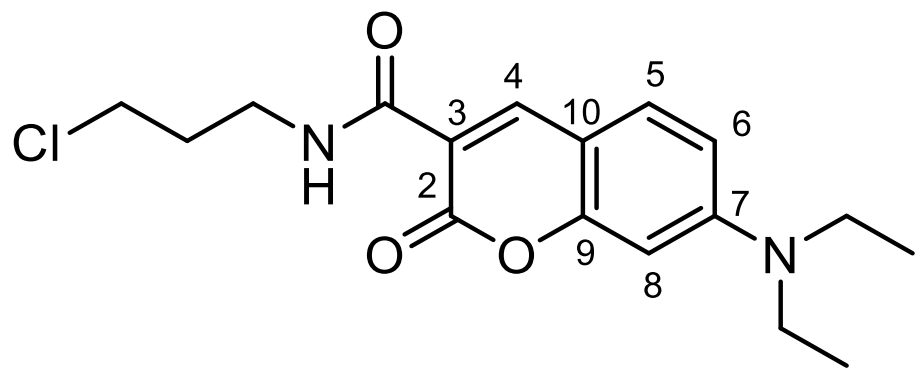

7-(diethylamino)coumarin-3-carboxylic acid (20 mg, 0.08 mmol, 1 equiv.), **HATU** (32 mg, 0.08 mmol, 1.1 equiv.), **Et_3N** (43 µL, 0.31 mmol, 4 equiv.), **3-chloropropylamine hydrochloride** (10.9 mg, 0.08 mmol, 1.1 equiv.), dry **DMF** (2 mL). TLC analysis (DCM/MeOH) showed full conversion in 1.5 h. After the extraction, the crude product was purified by flash column chromatography (silica gel, eluent: gradient of DCM/EtOAc) to obtain **ER-Cl-Coum (13)** as a yellow powder (24 mg, 93%).

^{1}H NMR (400 MHz, $CDCl_3$): δ = 8.88 (t, J = 6.0 Hz, 1H, N**H**), 8.69 (d, J = 0.7 Hz, 1H, **H**-4), 7.42 (d, J = 8.9 Hz, 1H, **H**-5), 6.64 (dd, J = 8.9, 2.5 Hz, 1H, **H**-6), 6.49 (d, J = 2.4 Hz, 1H, **H**-8), 3.62 (t, J = 6.5 Hz, 2H, C**H_2**Cl), 3.59 (q, J = 6.5 Hz, 2H, C**H_2**NH), 3.45 (q, J = 7.1 Hz, 4H, NC**H_2**CH_3), 2.10 (p, J = 6.6 Hz, 2H, ClCH_2C**H_2**CH_2NH), 1.23 (t, J = 7.1 Hz, 6H, N(CH_2C**H_3**)$_2$). **^{13}C NMR** (101 MHz, $CDCl_3$): δ = 163.6 (**C**ONH), 162.9 (**C**OO), 157.8 (C-9), 152.7 (C-7), 148.3 (C-4), 131.3 (C-5), 110.2 (C-3), 110.1 (C-6), 108.5 (C-10), 96.7 (C-8), 45.2 (N**C**H_2CH_3), 42.6 (Cl**C**H_2), 37.1 (**C**H_2NH), 32.6 (ClCH_2**C**H_2CH_2NH), 12.6 (N(CH_2**C**H_3)$_2$). **HRMS** (ESI+): calculated for $C_{17}H_{22}O_3N_2Cl$ ($[M+H]^+$) 337.1314, found 337.1313.

ER-SU1-Coum (16)

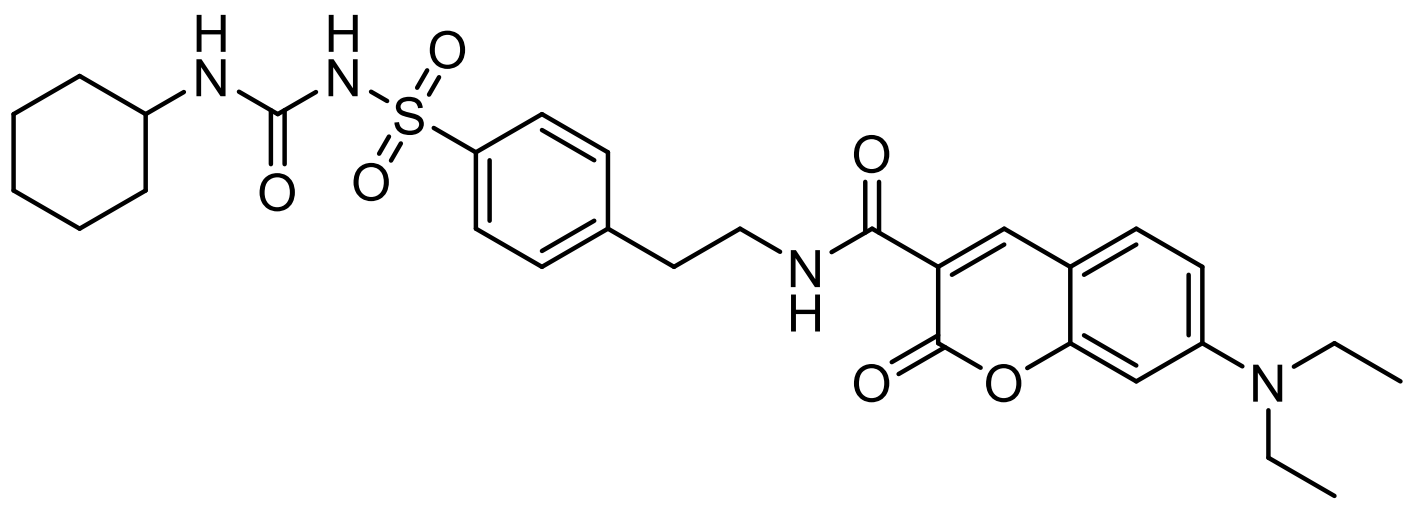

7-(diethylamino)coumarin-3-carboxylic acid (12 mg, 46 µmol, 1.0 equiv.), **HATU** (38 mg, 0.10 mmol, 2.2 equiv.), **DMAP** (<1 mg, 5 µmol, 0.1 equiv.), **DIPEA** (48 µL, 0.28 mmol, 6 equiv.), **4-(2-aminoethyl)-N-(cyclohexylcarbamoyl)benzenesulfonamide, 2,2,2-trifluoroacetate salt (41)** (30 mg, 92 µmol, 2 equiv.), **DMF** (2.2 mL). After the extraction, the crude product was purified by reversed-phase chromatography (C18 column, eluent: gradient of H_2O/MeCN) followed by lyophilisation to obtain **ER-SU1-Coum (16)** as a yellow powder (3 mg, 12%).

^{1}H NMR (400 MHz, $CDCl_3$): δ = 8.95 (t, $^3J_{H\text{-}H}$ = 5.8 Hz, 1H, N**H**), 8.69 (s, 1H, C**H**^{Ar}), 7.86–7.79 (m, 2H, C**H**^{Ar}), 7.48–7.39 (m, 3H, C**H**^{Ar}), 7.34 (bs, 1H, N**H**), 6.65 (dd, $^3J_{H\text{-}H}$ = 9.0 Hz, $^4J_{H\text{-}H}$ = 2.5 Hz, 1H, C**H**^{Ar}), 6.52–6.44 (m, 3H, C**H**^{Ar}, N**H**), 3.70 (dt, $^3J_{H\text{-}H}$ = 7.7 Hz, $^4J_{H\text{-}H}$ = 6.4 Hz, 2H, NHC**H_2**), 3.65–3.53 (m, 1H, C**H**), 3.46 (q, $^3J_{H\text{-}H}$ = 7.1 Hz, 4H, C**H_2**CH_3), 3.02 (t, $^3J_{H\text{-}H}$ = 7.3 Hz, 2H, C**H_2**^{Ar}), 1.89–1.81 (m, 2H, C**H_2**^{cy}), 1.75–1.51 (m, 4H, C**H_2**^{cy}), 1.43–

1.06 (m, 2H, C**H₂**^{cy}, C**H₃**) ppm, **^{13}C NMR** (101 MHz, $CDCl_3$): δ = 163.6 (C**C**ONH), 162.9 (O**C**O), 157.8 (**C**$_q^{Ar}$), 152.8 (**C**NCH₂), 150.1 (NH**C**ONH), 148.4 (**C**H^{Ar}), 146.2 (CH₂**C**^{Ar}), 137.7 (SO₂**C**^{Ar}), 131.4 (**C**H^{Ar}), 130.0 (**C**H^{Ar}), 127.4 (**C**H^{Ar}), 110.2 (**C**H^{Ar}), 110.0 (CO**C**^{Ar}), 108.5 (**C**$_q^{Ar}$), 96.7 (**C**H^{Ar}), 49.4 (**C**H), 45.3 (**C**H₂CH₃), 40.6 (NH**C**H₂), 36.0 (**C**H$_2^{Ar}$), 33.1 (**C**H$_2^{cy}$), 25.5 (**C**H$_2^{cy}$), 24.7 (**C**H$_2^{cy}$), 12.6 (**C**H₃) ppm, **LRMS** (ESI+), *m/z* (%): 591.2 [M+Na]$^+$ (100), 569.2 [M+H]$^+$ (20) , 1159.5 [2M+Na]$^+$ (5). **HRMS** (ESI+): calculated for $C_{29}H_{36}O_6N_4NaS^+$ ([M+Na]$^+$) 591.2248, found 591.2246, calculated for $C_{29}H_{37}O_6N_4S^+$ ([M+H]$^+$) 569.2428, found 569.2427.

Golgi-Coum (21)

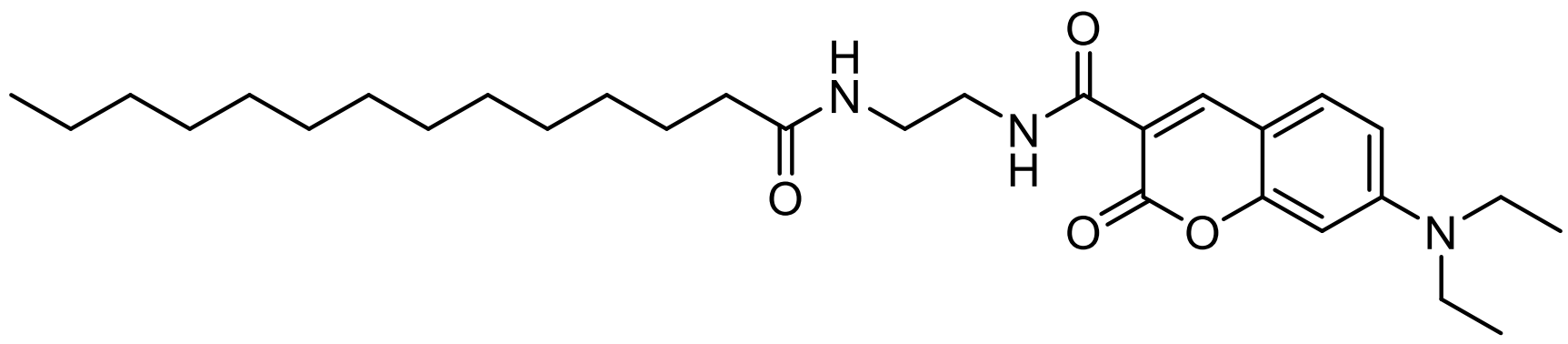

7-(diethylamino)coumarin-3-carboxylic acid (12 mg, 92 µmol, 1.0 equiv.), **HATU** (38 mg, 0.10 mmol, 2.2 equiv.), **DMAP** (<1 mg, 5 µmol, 0.1 equiv.), **DIPEA** (48 µL, 0.28 mmol, 6 equiv.), **2-tetradecanamidoethan-1-aminium 2,2,2-trifluoroacetate (43)** (35 mg, 92 µmol, 2 equiv.), **DMF** (2.2 mL). After the extraction, the crude product was purified by reversed-phase chromatography (C18 column, eluent: gradient of H_2O/MeCN) followed by lyophilisation to obtain **Golgi-Coum (21)** as a yellow powder (5 mg, 21%).

^{1}H NMR (400 MHz, $CDCl_3$): δ = 9.06 (t, $^3J_{H\text{-}H}$ = 6.2 Hz, 1H, N**H**), 8.68 (s, 1H, C**H**), 7.43 (d, $^3J_{H\text{-}H}$ = 9.0 Hz 1H, C**H**), 6.65 (dd, $^3J_{H\text{-}H}$ = 9.0 Hz, $^4J_{H\text{-}H}$ = 2.5 Hz, 1H, C**H**), 6.53–6.46 (m, 3H, C**H**, N**H**), 3.64–3.55 (m, 2H, NHC**H₂**), 3.51–3.41 (m, 6H, NHC**H₂**, NC**H₂**CH₃), 2.17 (t, $^3J_{H\text{-}H}$ = 7.7 Hz, 2H, COC**H₂**), 1.67–1.55 (m, 4H, C**H₂**), 1.37–1.13 (m, 4H, C**H₂**, NCH₂C**H₃**), 0.87 (t, $^3J_{H\text{-}H}$ = 6.8 Hz, 3H, C**H₃**) ppm, **^{13}C NMR** (101 MHz, $CDCl_3$): δ = 173.8 (NH**C**OCH₂), 165.0 (C**C**ONH), 162.8 (**C**OO), 157.9 (**C**$_q^{Ar}$), 152.9 (**C**NCH₂), 148.4 (**C**H), 131.4 (**C**H), 110.2 (**C**H), 109.9 (**C**CO), 108.5 (**C**$_q^{Ar}$), 96.7 (**C**H), 45.3 (N**C**H₂CH₃), 41.3 (**C**H₂NH), 39.3 (**C**H₂NH), 37.0 (CO**C**H₂), 32.1 (**C**H₂), 29.8 (**C**H₂), 29.8 (**C**H₂), 29.7 (**C**H₂), 29.6 (**C**H₂), 29.5 (**C**H₂), 29.5 (**C**H₂), 25.9 (**C**H₂), 22.8 (**C**H₂), 14.3 (**C**H₃), 12.6 (NCH₂**C**H₃) ppm. **LRMS** (ESI+), *m/z* (%): 536.3 [M+Na]$^+$ (100), 514.4 [M+H]$^+$ (5), 1049.7 [2M+Na]$^+$ (5). **HRMS** (ESI+): calculated for $C_{30}H_{48}O_4N_3$ ([M+H]$^+$) 514.3639, found 514.3635.

Lyso-M-Coum (24)

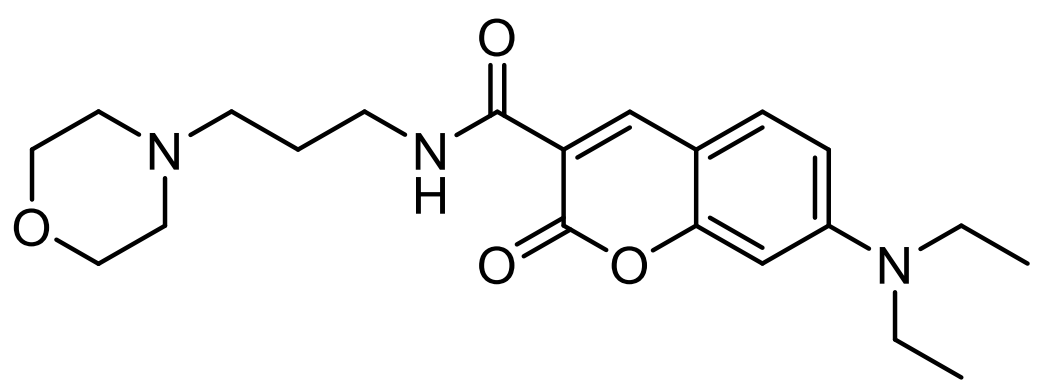

7-(diethylamino)coumarin-3-carboxylic acid (14 mg, 54 µmol, 1.0 equiv.), **HATU** (45 mg, 0.12 mmol, 2.2 equiv.), **DMAP** (<1 mg, 5 µmol, 0.1 equiv.), **DIPEA** (56 µL, 0.32 mmol, 6 equiv.), **4-(3-**

aminopropyl)morpholine (16 mg, 0.11 mmol, 2 equiv.), **DMF** (2.6 mL). After the extraction, the crude product was purified by reversed-phase chromatography (C18 column, eluent: gradient of H_2O/MeCN) followed by lyophilisation to obtain **Lyso-M-Coum (24)** as a yellow powder (14 mg, 67%).

^{1}H NMR (400 MHz, $CDCl_3$): δ = 8.91 (t, $^3J_{H-H}$ = 5.9 Hz, 1H, N**H**), 8.69 (s, 1H, C**H**), 7.42 (d, $^3J_{H-H}$ = 8.9 Hz, 1H, C**H**), 6.63 (dd, $^3J_{H-H}$ = 9.0 Hz, $^4J_{H-H}$ = 2.5 Hz, 1H, C**H**), 6.49 (d, $^4J_{H-H}$ = 2.5 Hz, 1H, C**H**), 3.72 (t, $^3J_{H-H}$ = 4.7 Hz, 4H, OC**H₂**), 3.54–3.40 (m, 6H, NHC$\mathbf{H_2}$, NC$\mathbf{H_2}$$CH_3$), 2.48–2.40 (m, 6H, NC$\mathbf{H_2}$$CH_2$), 1.80 (p, $^3J_{H-H}$ = 7.0 Hz, 2H, CH_2C$\mathbf{H_2}$$CH_2$), 1.23 (t, $^3J_{H-H}$ = 7.1 Hz, 6H, NCH_2C$\mathbf{H_3}$) ppm, **^{13}C NMR** (101 MHz, $CDCl_3$): δ = 163.3 (**C**ONH), 162.8 (**C**OO), 157.7 (**C**$_q^{Ar}$), 152.6 (**C**NCH_2), 148.1 (**C**H), 131.2 (**C**H), 110.5 (**C**COO), 110.0 (**C**H), 108.5 (**C**$_q^{Ar}$), 96.7 (**C**H), 67.1 (O**C**H_2), 56.7 (N**C**H_2), 53.9 (N**C**H_2), 45.2 (N**C**H_2CH_3), 38.1 (NH**C**H_2), 26.4 (CH_2**C**H_2CH_2), 12.6 (NCH_2**C**H_3) ppm. **LRMS** (ESI+), *m/z* (%): 388.2 $[M+H]^+$ (100), 410.2 $[M+Na]^+$ (10). **HRMS** (ESI+): calculated for $C_{21}H_{30}O_4N_3^+$ ($[M+H]^+$) 388.2231, found 388.2229.

Lyso-N-Coum (26)

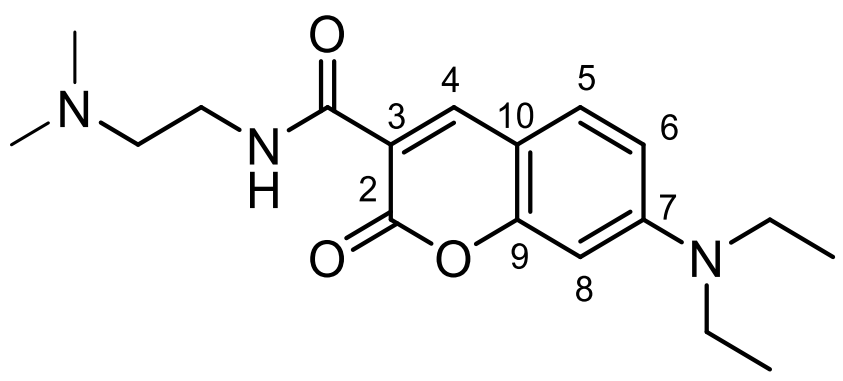

7-(diethylamino)coumarin-3-carboxylic acid (20 mg, 0.08 mmol, 1 equiv.), **HATU** (32 mg, 0.08 mmol, 1.1 equiv.), **Et_3N** (43 µL, 0.31 mmol, 4 equiv.), ***N,N*-dimethylethylenediamine** (9.2 µL, 0.08 mmol, 1.1 equiv.), dry **DMF** (1 mL). TLC analysis (DCM/MeOH) showed full conversion in 1.5 h. After the extraction, the crude product was purified by flash column chromatography (silica gel, eluent: gradient of DCM/MeOH + 0.5% Et_3N) and lyophilisation from MeCN/H_2O to obtain **Lyso-N-Coum (26)** as a yellow powder (25 mg, 98%).

^{1}H NMR (400 MHz, $CDCl_3$): δ = 8.92 (t, *J* = 5.0 Hz, 1H), 8.68 (d, *J* = 0.7 Hz, 1H, **H**-4), 7.41 (d, *J* = 8.9 Hz, 1H, **H**-5), 6.63 (dd, *J* = 9.0, 2.5 Hz, 1H, **H**-6), 6.48 (d, *J* = 2.4 Hz, 1H, **H**-8), 3.55 (td, *J* = 6.6, 5.4 Hz, 2H, C$\mathbf{H_2}$NH), 3.44 (q, *J* = 7.1 Hz, 4H, N(C$\mathbf{H_2}$$CH_3$)), 2.53 (t, *J* = 6.6 Hz, 2H, C$\mathbf{H_2}$N$(CH_3)_2$), 2.30 (s, 6H, N(C$\mathbf{H_3}$)$_2$), 1.23 (t, *J* = 7.1 Hz, 6H, N(CH_2C$\mathbf{H_3}$)$_2$). **^{13}C** NMR (101 MHz, $CDCl_3$): δ = 163.34 (**C**ONH), 162.73 (**C**OO), 157.76 (C-9), 152.58 (C-7), 148.07 (C-4), 131.20 (C-5), 110.63 (C-3), 109.97 (C-6), 108.50 (C-10), 96.72 (C-8), 58.42 (**C**$H_2N(CH_3)_2$), 45.57 N(**C**H_3)$_2$, 45.18 N(CH_2CH_3)$_2$, 37.71 (**C**H_2NH), 12.56 (CH_2N(**C**H_3)$_2$). **HRMS** (ESI+): calculated for $C_{18}H_{26}O_2N_3^+$ ($[M+H]^+$) 332.1969, found 332.1969, calculated for $C_{18}H_{25}O_3N_3Na^+$ ($[M+Na]^+$) 354.1788, found 354.1789.

2.6 Synthesis of the labeling fluorophores

Coum-Tz (27)

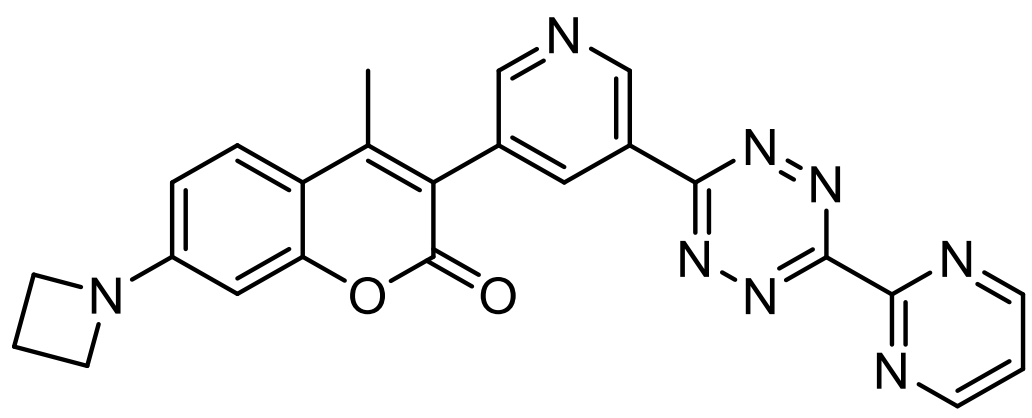

This compound was synthesized according to the published protocol.[18]

cTCO-QCoum (28)

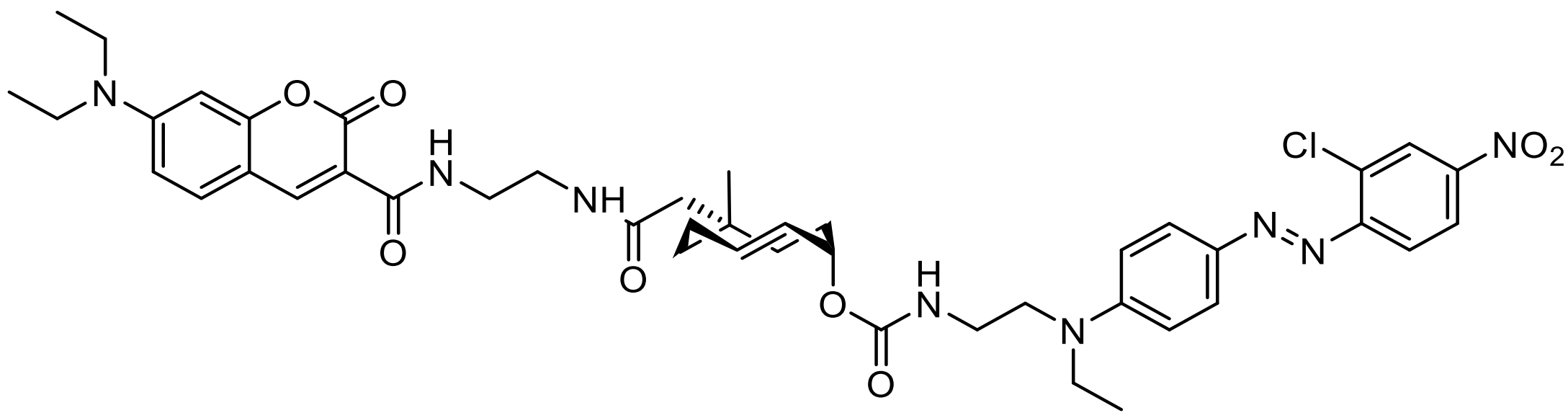

This compound was synthesized according to the published protocol.[19]

TAMRA-TCO (29)

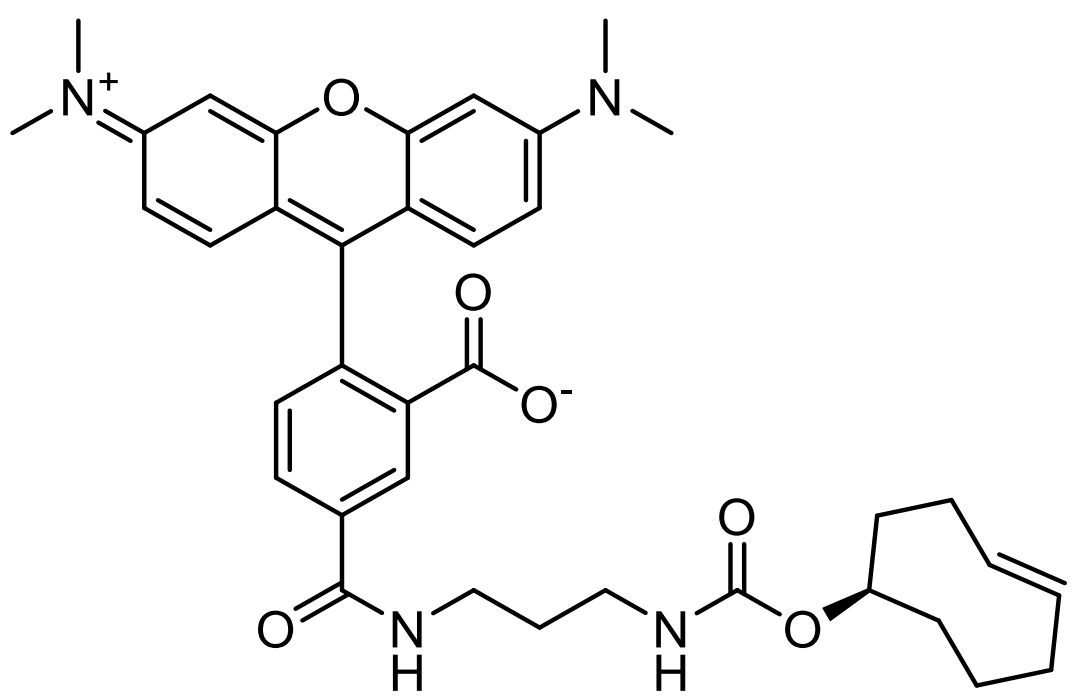

This compound was synthesized according to the published protocol.[20-21]

2.7 Synthesis of the doxorubicin caged compounds

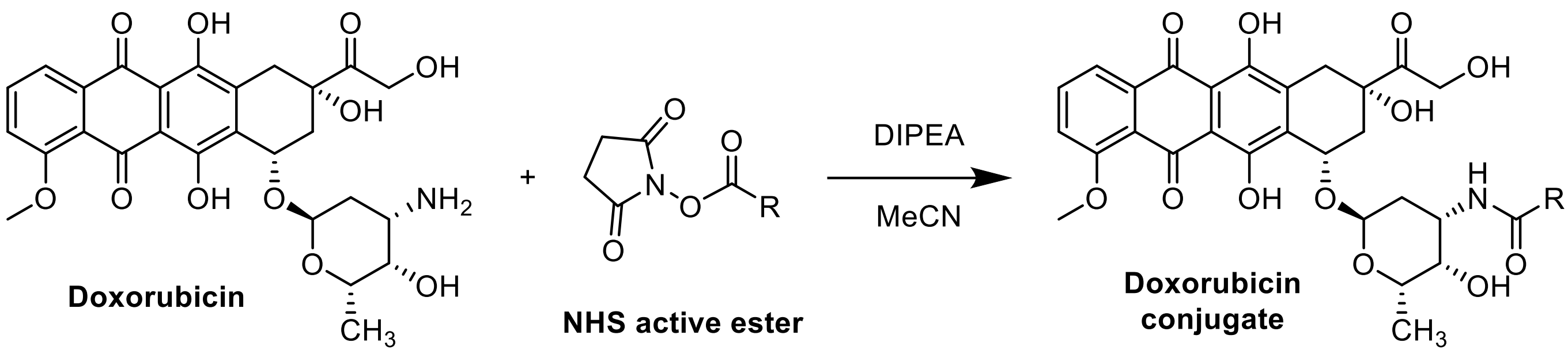

General procedure for the synthesis of the doxorubicin caged compounds:

Under an argon atmosphere, **doxorubicin hydrochloride (30)** and the corresponding **NHS active ester** were dissolved in dry MeCN, followed by the addition of **DIPEA**. The reaction mixture was stirred at RT until full conversion was observed by LC-MS analysis. The crude reaction mixture was diluted with water and purified by reversed-phase chromatography (C18 column, eluent: gradient of H_2O/MeCN) followed by lyophilization to obtain the desired doxorubicin derivative.

Doxo-Tz (31)

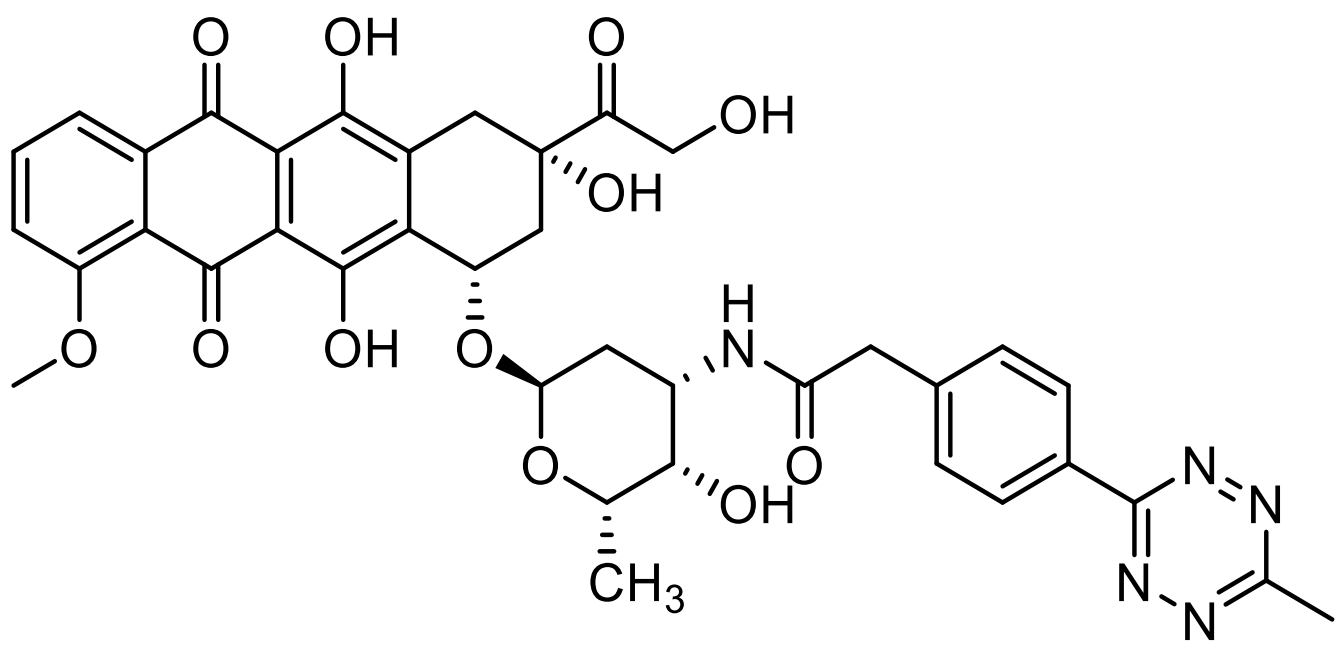

Doxorubicin hydrochloride (30) (22 mg, 38 µmol, 1.0 equiv.), **methyltetrazine-NHS ester** (14 mg, 42 µmol, 1.1 equiv.), **DIPEA** (27 µL, 0.15 mmol, 4 equiv.), **MeCN** (384 µL). **Doxo-Tz (31)** was obtained as a red solid (13 mg, 17 µmol, 44%).

^{1}H NMR (400 MHz, DMSO-d_6): δ = 14.01 (s, 1H, CO**H**), 13.25 (s, 1H, CO**H**), 8.33 (d, $^3J_{H-H}$ = 8.3 Hz, 2H, CC**H**CHC), 8.00–7.93 (m, 1H, N**H**), 7.92–7.83 (m, 2H, C**H**^{Ar}), 7.66–7.57 (m, 1H, C**H**^{Ar}), 7.49 (d, $^3J_{H-H}$ = 8.3 Hz, 2H, CC**H**CHC), 5.44 (s, 1H, OCHCH$_2$CO**H**), 5.27–5.21 (m, 1H, C**H**OO), 4.99–4.91 (m, 1H, OC**H**CH$_2$COH), 4.56 (s, 2H, CH$_2$O**H**), 4.17 (q, $^3J_{H-H}$ = 6.5 Hz, 1H, C**H**CH$_3$), 4.05–3.93 (m, 4H, NHC**H**CH$_2$, OC**H$_3$**), 3.57–3.52 (m, 1H, C**H$_2$**CONH), 3.46–3.41 (m, 1H, CH$_3$CHC**H**OH), 3.03–2.87 (m, 6H, C**H$_3$**CNN, C**H$_2$**), 2.42–2.06 (m, 2H,C**H$_2$**), 1.95–1.85 (m, 1H, C**H$_2$**), 1.50–1.41 (m, 1H, C**H$_2$**), 1.13 (d, $^3J_{H-H}$ = 6.5 Hz, 3H, C**H$_3$**), **^{13}C NMR** (101 MHz, DMSO-d_6): δ = 213.8 (C**C**OCH$_2$), 186.6 (C**C**OC), 186.5 (C**C**OC), 168.9 (**C**ONH), 167.0 (**C**$_q^{Ar}$), 163.2 (**C**$_q^{Ar}$), 160.8 (CH$_3$O**C**), 157.9 (**C**$_q^{Ar}$), 156.1 (**C**$_q^{Ar}$), 154.5 (OHC$_q^{Ar}$), 141.5 (OH**C**$_q^{Ar}$), 136.2 (**C**H^{Ar}),135.5 (**C**$_q^{Ar}$), 134.7 (**C**$_q^{Ar}$), 134.0 (**C**$_q^{Ar}$), 130.0 (**C**H^{Ar}), 127.2 (**C**H^{Ar}), 120.0 (**C**$_q^{Ar}$), 119.7 (**C**H^{Ar}), 119.0 (**C**H^{Ar}), 110.8 (**C**$_q^{Ar}$), 110.7

(C_q^{Ar}), 100.4 (**C**HOO), 75.0 (OH**C**CHOCH$_2$), 70.0 (O**C**HCH$_2$COH), 68.1 (CH$_3$CH**C**HOH), 66.7 (**C**HCH$_3$), 63.7 (**C**H$_2$OH), 56.6 (O**C**H$_3$), 45.2 (NH**C**H), 42.1 (**C**H$_2$CONH), 36.7 (**C**H$_2$), 32.1 (**C**H$_2$), 29.8 (**C**H$_2$), 20.8 (**C**H$_3$CNN), 17.0 (CH**C**H$_3$). ppm, **LRMS** (ESI+), *m/z* (%): 778.2 $[M+Na]^+$ (100), 1533.5 $[2M+Na]^+$ (5), **LRMS** (ESI−), *m/z* (%): 754.2 $[M-H]^-$ (100), **HRMS** (ESI+): calculated for $C_{38}H_{37}O_{12}N_5Na^+$ ($[M+H]^+$) 778.2331, found 778.2333, **HRMS** (ESI−): calculated for $C_{38}H_{36}O_{12}N_5^-$ ($[M-H]^-$) 754.2366, found 754.2370.

Doxo-TCO (32)

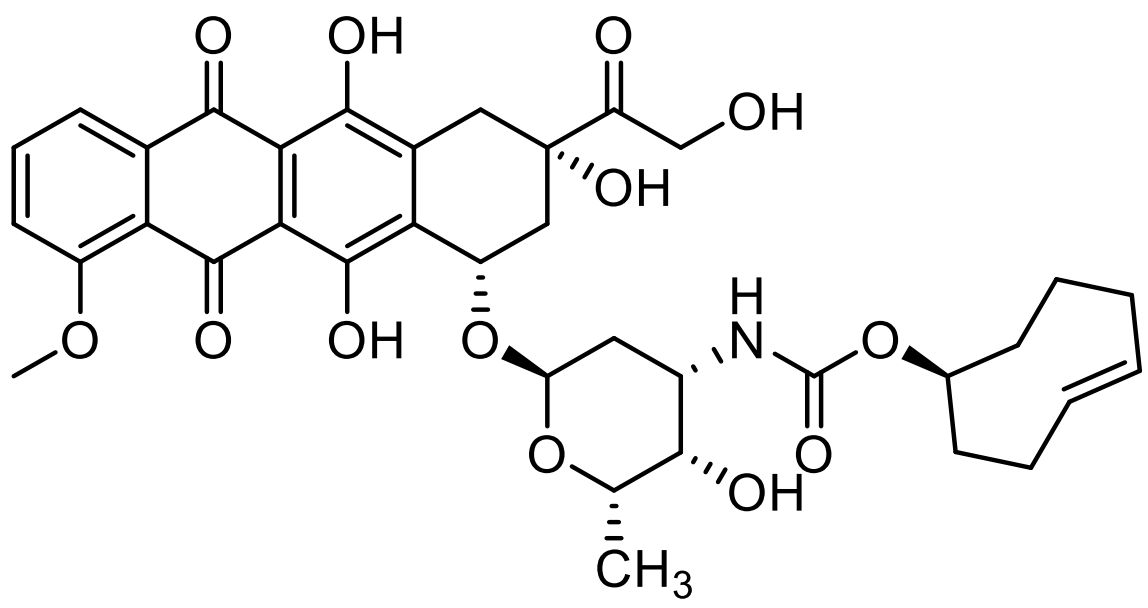

Doxorubicin hydrochloride (30) (19 mg, 33 µmol, 1.0 equiv.), **TCO-4-en-NHS ester** (10 mg, 37 µmol, 1.1 equiv.), **DIPEA** (23 µL, 0.13 mmol, 4 equiv.), **MeCN** (333 µL). **Doxo-TCO (32)** was obtained as a red solid (16 mg, 23 µmol, 69%).

^{1}H NMR (400 MHz, DMSO-d_6): δ = 13.98 (s, 1H, CO**H**), 13.22 (s, 1H, CO**H**), 7.90–7.80 (m, 2H, C**H**^{Ar}), 7.64–7.55 (m, 1H, C**H**^{Ar}), 6.78–6.66 (m, 1H, N**H**), 5.71–5.59 (m, 1H, C**H**=CH), 5.52–5.39 (m, 1H, C**H**=CH), 5.27–5.20 (m, 2H, OCHCH$_2$CO**H**, C**H**OO), 4.97–4.88 (m, 1H, OC**H**CH$_2$COH), 4.88–4.79 (m, 1H, CH$_2$O**H**), 4.77–4.69 (m, 1H, CH$_3$CHCHO**H**), 4.69–4.61 (m, 1H, OC**H**CH$_2$CH$_2$), 4.57 (d, $^3J_{H\text{-}H}$ = 5.9 Hz, 2H, C**H$_2$**OH), 4.16 (q, $^3J_{H\text{-}H}$ = 6.5 Hz, 1H, C**H**CH$_3$), 3.96 (s, 3H, OC**H$_3$**), 3.78–3.66 (m, 1H, NHC**H**), 3.47–3.41 (m, 1H, CH$_3$CHC**H**OH), 3.04–2.83 (m, 2H, C**H$_2$**), 2.33–1.04 (m, 14H, C**H$_2$**), 1.14 (d, $^3J_{H\text{-}H}$ = 6.5 Hz, 3H, CHC**H$_3$**) ppm, **^{13}C NMR** (101 MHz, DMSO-d_6): δ = 213.8 (C**C**OCH$_2$), 186.4 (C**C**OC), 186.3 (C**C**OC), 160.7 (CH$_3$O**C**), 156.1 (**C**OONH), 155.0 (OHC_q^{Ar}), 154.5 (OHC_q^{Ar}), 136.2 (**C**H^{Ar}), 135.5 (C_q^{Ar}), 135.5 (**C**H=CH), 135.2 (C_q^{Ar}), 135.1 (C_q^{Ar}), 134.6 (C_q^{Ar}), 134.0 (C_q^{Ar}), 131.5–131.3 (m, **C**H=CH), 119.9 (**C**H^{Ar}), 119.7 (C_q^{Ar}), 118.9 (**C**H^{Ar}), 110.7 (C_q^{Ar}), 110.6 (C_q^{Ar}), 100.4 (**C**HOO), 74.9 (OH**C**CHOCH$_2$), 69.9 (O**C**HCH$_2$COH), 68.5–68.2 (m, O**C**HCH$_2$CH$_2$, CH$_3$CH**C**HOH), 66.9–66.7 (m, **C**HCH$_3$), 63.7 (**C**H$_2$OH), 56.6 (O**C**H$_3$), 46.9 (NH**C**H), 40.5 (**C**H$_2$), 40.4 (**C**H$_2$), 36.5 (**C**H$_2$), 34.0–33.9 (m, **C**H$_2$), 32.2–32.1 (m, **C**H$_2$), 32.0 (**C**H$_2$), 30.0 (**C**H$_2$), 29.6–29.4 (m, **C**H$_2$), 27.5–27.3 (m, **C**H$_2$), 17.0 (CH**C**H$_3$) ppm, **LRMS** (ESI+), *m/z* (%): 718.2 $[M+Na]^+$ (100), 713.3 $[M+NH_4]^+$ (5), 734.2 $[M+K]^+$ (5), 1413.5 $[2M+Na]^+$ (5), **LRMS** (ESI−), *m/z* (%): 694.3 $[M-H]^+$ (100), **HRMS** (ESI−): calculated for $C_{36}H_{40}O_{13}N^-$ ($[M-H]^-$) 694.2505, found 694.2510.

Doxo-rTz (33)

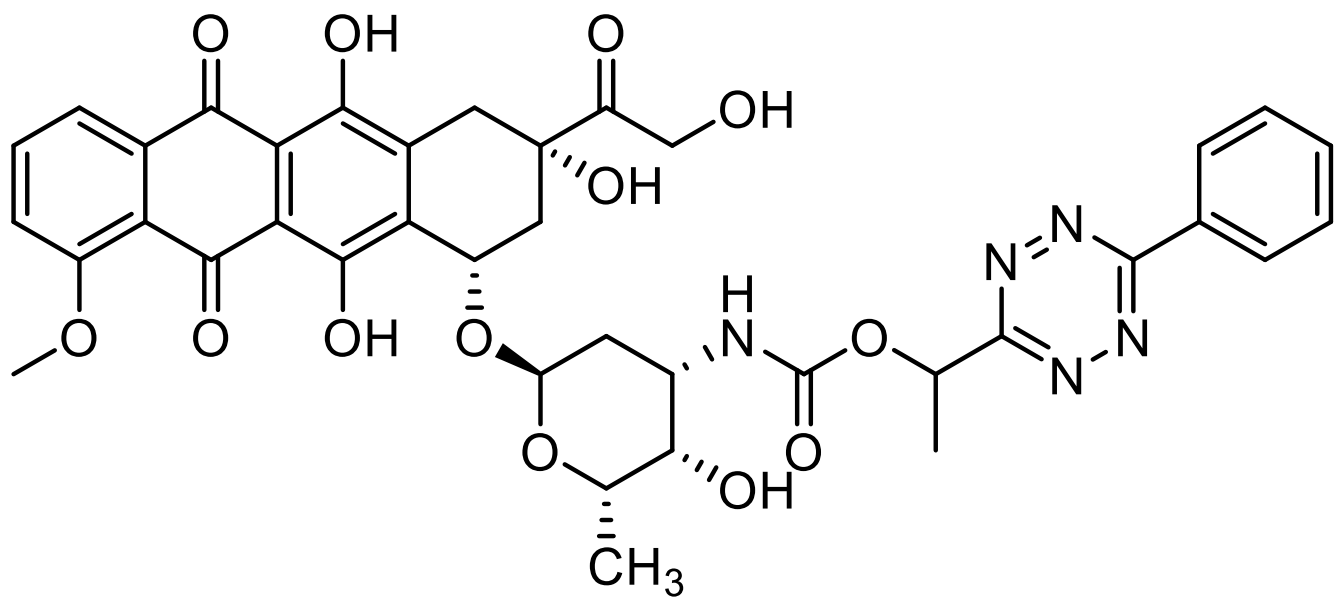

Doxorubicin hydrochloride (30) (22 mg, 38 µmol, 1.0 equiv.), **2,5-dioxopyrrolidin-1-yl (1-(6-phenyl-1,2,4,5-tetrazin-3-yl)ethyl) carbonate (46)** (14 mg, 41 µmol, 1.1 equiv.), **DIPEA** (26 µL, 0.15 mmol, 4 equiv.), **MeCN** (372 µL). **Doxo-rTz (33)** was obtained as a red solid (18.3 mg, 24 µmol, 64%).

^{1}H NMR (400 MHz, DMSO-d_6): δ = 13.98–13.88 (m, 1H, CO**H**), 13.21–13.14 (m, 1H, CO**H**), 8.49–8.30 (m, 2H, C**H**^{Ar}), 7.86–7.44 (m, 6H, C**H**^{Ar}), 7.31–7.12 (m, 1H, N**H**), 6.08–5.97 (m, 1H, CH_3C**H**CNN), 5.42–5.32 (m, 1H, $OCHCH_2CO$**H**), 5.23–5.17 (m, 1H, C**H**OO), 4.92–4.80 (m, 2H, CH_2O**H**, OC**H**CH_2COH), 4.78–4.65 (m, 1H, CH_3CHCHO**H**), 4.55 (d, J = 5.7 Hz, 2H, C**H₂**OH), 4.19–4.08 (m, 1H, CH_3C**H**CHOH), 3.97–3.87 (m, 3H, OC**H₃**), 3.75–3.61 (m, 1H, NHC**H**), 3.50–3.38 (m, 1H, CH_3CHC**H**OH), 2.99–2.79 (m, 2H, C**H₂**), 2.22–2.01 (m, 2H, C**H₂**), 1.96–1.82 (m, 1H, C**H₂**), 1.71–1.64 (m, 3H, C**H₃**CHCNN), 1.57–1.40 (m, 1H, C**H₂**), 1.17–1.04 (m, 3H, C**H₃**CHCHOH) ppm, **^{13}C NMR** (101 MHz, DMSO-d_6): δ = 213.9–213.7 (m, C**C**OCH_2), 186.5–186.1 (m, C**C**OC), 168.8–168.5 (m, **C**$_q^{Tz}$), 164.3–164.1 (m, **C**$_q^{Tz}$), 160.8–160.6 (m, CH_3O**C**), 156.1–155.9 (m, **C**OONH), 154.7–154.4 (m, OH**C**$_q^{Ar}$), 136.3–135.9 (m, **C**H^{Ar}), 135.5–135.3 (m, **C**$_q^{Ar}$), 134.6–134.4 (m, **C**$_q^{Ar}$), 134.1–133.8 (m, **C**$_q^{Ar}$), 132.9–132.6 (m, **C**$_q^{Ar}$), 131.6–131.4 (m, **C**H^{Ar}), 129.6–129.2 (m, **C**H^{Ar}), 127.8–127.5 (m, **C**H^{Ar}), 119.9–119.7 (m, **C**H^{Ar}), 119.7–119.5 (m, **C**$_q^{Ar}$), 119.0–118.8 (m, **C**H^{Ar}), 110.7–110.6 (m, **C**$_q^{Ar}$), 110.6–110.5 (m, **C**$_q^{Ar}$), 100.4–100.2 (m, **C**HOO), 75.0–74.8 (m, OH**C**CHOCH_2), 70.2–70.0 (m, CH_3**C**HCNN), 69.9–69.7 (m, O**C**HCH_2COH), 68.2–67.8 (m, CH_3CH**C**HOH), 66.8–66.6 (m, CH_3**C**HCHOH), 63.8–63.6 (m, **C**H_2OH), 56.6–56.3 (m, O**C**H_3), 47.6–47.3 (m, NH**C**H), 36.7–36.4 (m, **C**H_2), 32.2–31.8 (m, **C**H_2), 30.0–29.6 (m, **C**H_2), 19.6–19.4 (m, **C**H_3CHCNN), 17.2–16.9 (m, **C**H_3CHCHOH) ppm, **LRMS** (ESI+), *m/z* (%): 794.2 $[M+Na]^+$ (100), 1565.5 $[2M+Na]^+$ (10), **LRMS** (ESI−), *m/z* (%): 792.2 $[M-2H+Na]^+$ (100), 770.2 $[M-H]^-$ (45), **HRMS** (ESI−): calculated for $C_{38}H_{36}O_{13}N_5^-$ ($[M-H]^-$) 770.2315, found 770.2320, calculated for $C_{38}H_{35}O_{13}N_5Na^-$ ($[M-2H+Na]^-$) 792.2135, found 792.2139.

Doxo-rTCO (34)

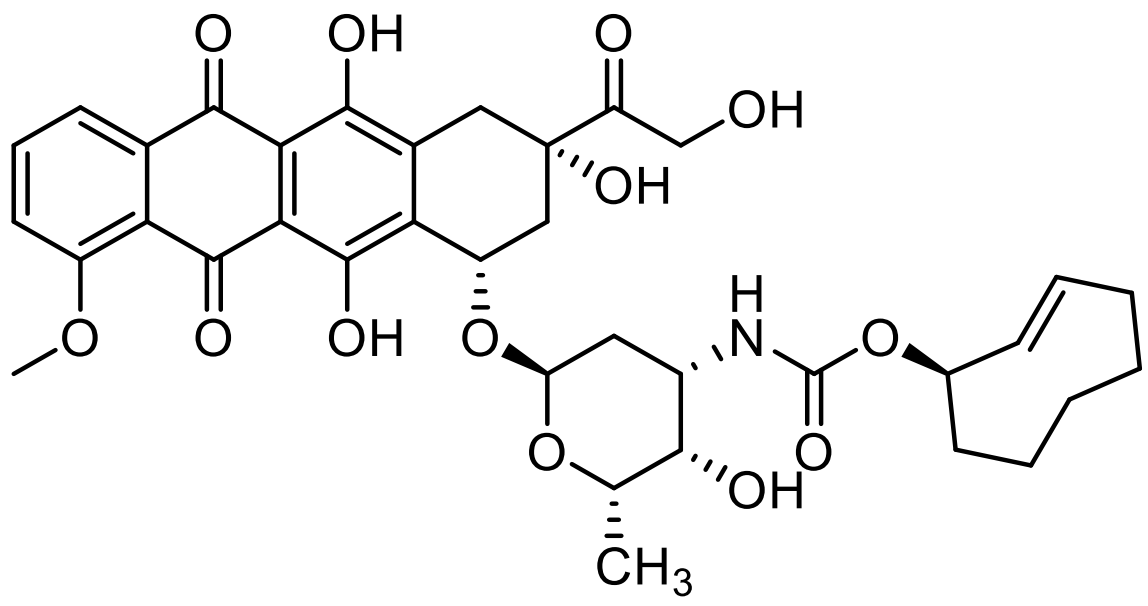

Doxorubicin hydrochloride (30) (19 mg, 33 µmol, 1.0 equiv.), **TCO*-NHS ester** (10 mg, 36 µmol, 1.1 equiv.), **DIPEA** (23 µL, 0.13 mmol, 4 equiv.), **MeCN** (327 µL). **Doxo-rTCO (34)** was obtained as a red solid (16 mg, 23 µmol, 70%).

^{1}H NMR (400 MHz, DMSO-d_6): δ = 13.98 (s, 1H, CO**H**), 13.21 (s, 1H, CO**H**), 7.90–7.80 (m, 2H, C**H**^{Ar}), 7.64–7.56 (m, 1H, C**H**^{Ar}), 6.75–6.65 (m, 1H, N**H**), 5.83–5.63 (m, 1H, C**H**=CH), 5.53–5.42 (m, 1H, C**H**=CH), 5.42–5.32 (m, 1H, OCHCH$_2$CO**H**), 5.29–5.15 (m, 1H, C**H**OO), 5.12–5.06 (m, 1H, OC**H**CH$_2$CH$_2$), 4.93–4.82 (m, 2H, OC**H**CH$_2$COH, CH$_2$O**H**), 4.73–4.67 (m, 1H, CH$_3$CHCHO**H**), 4.57 (d, $^{3}J_{H\text{-}H}$ = 5.7 Hz, 2H, C**H$_2$**OH), 4.15 (q, $^{3}J_{H\text{-}H}$ = 6.56 Hz, 1H, C**H**CH$_3$), 3.96 (s, 3H, OC**H$_3$**), 3.74–3.64 (m, 1H, NHC**H**), 3.47–3.39 (m, 1H, CH$_3$CHC**H**OH), 3.04–2.71 (m, 2H, C**H$_2$**), 2.43–0.65 (m, 14H, C**H$_2$**), 1.13 (d, $^{3}J_{H\text{-}H}$ = 6.5 Hz, 3H, CHC**H$_3$**) ppm, **^{13}C NMR** (101 MHz, DMSO-d_6): δ = 213.8 (C**C**OCH$_2$), 186.4 (C**C**OC), 186.3 (C**C**OC), 160.7 (CH$_3$O**C**), 156.1 (**C**OONH), 154.6 (OH**C**$_q^{Ar}$), 154.5 (OH**C**$_q^{Ar}$), 136.2 (**C**H^{Ar}), 135.5 (**C**$_q^{Ar}$), 134.6 (**C**$_q^{Ar}$), 134.0 (**C**$_q^{Ar}$), [132.3, 132.1] (CH=CH), [130.9, 130.6] (CH=CH), 119.9 (**C**H^{Ar}), 119.7 (**C**$_q^{Ar}$), 118.9 (**C**H^{Ar}), 110.7 (**C**H^{Ar}), 110.6 (**C**$_q^{Ar}$), 100.4 (**C**HOO), 74.9 (OH**C**CHOCH$_2$), 72.6 (O**C**HCH$_2$CH$_2$), 69.9 (O**C**HCH$_2$COH), 68.3–68.0 (m, CH$_3$CH**C**HOH), 66.8–66.6 (m, **C**HCH$_3$), 63.7 (**C**H$_2$OH), 56.5 (O**C**H$_3$), 46.9 (NH**C**H), 36.5 (**C**H$_2$), 35.5 (**C**H$_2$), 35.3 (**C**H$_2$), 35.2 (**C**H$_2$), 32.0 (**C**H$_2$), 29.9 (**C**H$_2$), 28.4 (**C**H$_2$), 23.7 (**C**H$_2$), 23.6 (**C**H$_2$), 17.0 (CH**C**H$_3$) ppm, **LRMS** (ESI+), *m/z* (%): 718.2 $[M+Na]^+$ (100), 713.3 $[M+NH_4]^+$ (5), 1413.5 $[2M+Na]^+$ (5), **LRMS** (ESI−), *m/z* (%): 694.3 $[M-H]^+$ (100), **HRMS** (ESI−): calculated for $C_{36}H_{40}O_{13}N^-$ ($[M-H]^-$) 694.2505, found 694.2508.

3. REFERENCES

[1] M. S. Kyllönen, S. Parkkila, H. Rajaniemi, A. Waheed, J. H. Grubb, G. N. Shah, W. S. Sly, K. Kaunisto "Localization of carbonic anhydrase XII to the basolateral membrane of H+-secreting cells of mouse and rat kidney", *J Histochem Cytochem* **2003**, *51*, 1217-1224. https://doi.org/10.1177/002215540305100912

[2] S. S. Lam, J. D. Martell, K. J. Kamer, T. J. Deerinck, M. H. Ellisman, V. K. Mootha, A. Y. Ting "Directed evolution of APEX2 for electron microscopy and proximity labeling", *Nat Methods* **2015**, *12*, 51-54. https://doi.org/10.1038/nmeth.3179

[3] N. Mosammaparast, K. R. Jackson, Y. R. Guo, C. J. Brame, J. Shabanowitz, D. F. Hunt, L. F. Pemberton "Nuclear import of histone H2A and H2B is mediated by a network of karyopherins", *J Cell Biol* **2001**, *153*, 251-262. https://doi.org/10.1083/jcb.153.2.251

[4] W. R. Ma, C. Mayr "A Membraneless Organelle Associated with the Endoplasmic Reticulum Enables 3′UTR-Mediated Protein-Protein Interactions", *Cell* **2018**, *175*, 1492-+. https://doi.org/10.1016/j.cell.2018.10.007

[5] N. Yamaguchi, M. N. Fukuda "Golgi Retention Mechanism of Beta-1,4-Galactosyltransferase - Membrane-Spanning Domain-Dependent Homodimerization and Association with Alpha-Tubulins and Beta-Tubulins", *J Biol Chem* **1995**, *270*, 12170-12176. https://doi.org/10.1074/jbc.270.20.12170

[6] M. Abu-Remaileh, G. A. Wyant, C. Kim, N. N. Laqtom, M. Abbasi, S. H. Chan, E. Freinkman, D. M. Sabatini "Lysosomal metabolomics reveals V-ATPase- and mTOR-dependent regulation of amino acid efflux from lysosomes", *Science* **2017**, *358*, 807-+. https://doi.org/10.1126/science.aan6298

[7] G. A. Wyant, M. Abu-Remaileh, R. L. Wolfson, W. W. Chen, E. Freinkman, L. V. Danai, M. G. V. Heiden, D. M. Sabatini "mTORC1 Activator SLC38A9 Is Required to Efflux Essential Amino Acids from Lysosomes and Use Protein as a Nutrient", *Cell* **2017**, *171*, 642-+. https://doi.org/10.1016/j.cell.2017.09.046

[8] S. Bolte, F. P. Cordelières "A guided tour into subcellular colocalization analysis in light microscopy", *J Microsc-Oxford* **2006**, *224*, 213-232. https://doi.org/10.1111/j.1365-2818.2006.01706.x

[9] M. Sadoine, Y. Ishikawa, T. J. Kleist, M. M. Wudick, M. Nakamura, G. Grossmann, W. B. Frommer, C. H. Ho "Designs, applications, and limitations of genetically encoded fluorescent sensors to explore plant biology", *Plant Physiol* **2021**, *187*, 485-503. https://doi.org/10.1093/plphys/kiab353

[10] M. Q. Zhu, W. Z. Li, L. Sun, Z. N. Lv, X. F. Yang, Y. Wang "Advances in fluorescent probes for targeting organelles: Design strategies, applications and perspectives", *Coordin Chem Rev* **2024**, *512*. https://doi.org/10.1016/j.ccr.2024.215893

[11] M. J. Wang, Y. Da, Y. Tian "Fluorescent proteins and genetically encoded biosensors", *Chem Soc Rev* **2023**, *52*, 1189-1214. https://doi.org/10.1039/d2cs00419d

[12] K. Sakai, M. Kaji, Y. Takamatsu, K. Tsuchiya, K. Torigoe, K. Tsubone, T. Yoshimura, K. Esumi, H. Sakai, M. Abe "Fluorocarbon–hydrocarbon gemini surfactant mixtures in aqueous solution", *Colloids and Surfaces A: Physicochemical and Engineering Aspects* **2009**, *333*, 26-31. https://doi.org/10.1016/j.colsurfa.2008.09.022

[13] D. I. Danylchuk, S. Moon, K. Xu, A. S. Klymchenko "Switchable Solvatochromic Probes for Live-Cell Super-resolution Imaging of Plasma Membrane Organization", *Angewandte Chemie* **2019**, *131*, 15062-15066. https://doi.org/10.1002/ange.201907690

[14] X. Zhang, A. Xu, Y. Ran, C. Wei, F. Xie, J. Wu "Design, synthesis and biological evaluation of phenyl vinyl sulfone based NLRP3 inflammasome inhibitors", *Bioorg Chem* **2022**, *128*, 106010. https://doi.org/10.1016/j.bioorg.2022.106010

[15] K. Saito, K. Nakajima, T. Taniguchi, O. Iwamoto, S. Shibuya, Y. Ogawa, K. Aoki, N. Kurikawa, S. Tanaka, M. Ogitani, E. Kioi, K. Ito, N. Nishihama, T. Mikkaichi, W. Saitoh, **2019**. Patent Number: A1 EP3438103.

[16] K. Saito, K. Nakajima, Y. Ogawa, M. Makino, K. Ito, S. Nagata, H. Makoto, **2020**. Patent Number: A1 US2020/216433.

[17] A. H. A. M. van Onzen, R. M. Versteegen, F. J. M. Hoeben, I. A. W. Filot, R. Rossin, T. Zhu, J. Wu, P. J. Hudson, H. M. Janssen, W. ten Hoeve, M. S. Robillard "Bioorthogonal Tetrazine Carbamate Cleavage by Highly Reactive trans-Cyclooctene", *J Am Chem Soc* **2020**, *142*, 10955-10963. https://doi.org/10.1021/jacs.0c00531

[18] J. Galeta, R. Dzijak, J. Oboril, M. Dracínsky, M. Vrabel "A Systematic Study of Coumarin-Tetrazine Light-Up Probes for Bioorthogonal Fluorescence Imaging", *Chem-Eur J* **2020**, *26*, 9945-9953. https://doi.org/10.1002/chem.202001290

[19] M. Rahm, P. Keppel, V. Slachtová, R. Dzijak, M. Dracínsky, S. Bellová, P. E. Reyes-Gutiérrez, S. Stepánová, J. Raffler, E. Tloustová, H. Mertlíková-Kaiserová, H. Mikula, M. Vrabel "Sulfonated Hydroxyaryl-Tetrazines with Increased pKa for Accelerated Bioorthogonal Click-to-Release Reactions in Cells", *Angew Chem Int Edit* **2025**, *64*. https://doi.org/10.1002/anie.202411713

[20] H. E. Murrey, J. C. Judkins, C. W. Am Ende, T. E. Ballard, Y. Fang, K. Riccardi, L. Di, E. R. Guilmette, J. W. Schwartz, J. M. Fox, D. S. Johnson "Systematic Evaluation of Bioorthogonal Reactions in Live Cells with Clickable HaloTag Ligands: Implications for Intracellular Imaging", *J Am Chem Soc* **2015**, *137*, 11461-11475. https://doi.org/10.1021/jacs.5b06847

[21] C. J. Paresi, Q. Liu, Y.-M. Li "Benzimidazole covalent probes and the gastric H+/K+-ATPase as a model system for protein labeling in a copper-free setting", *Molecular BioSystems(MBS)* **2016**, *12*, 1772-1780. https://doi.org/10.1039/c6mb00024j

4. NMR SPECTRA

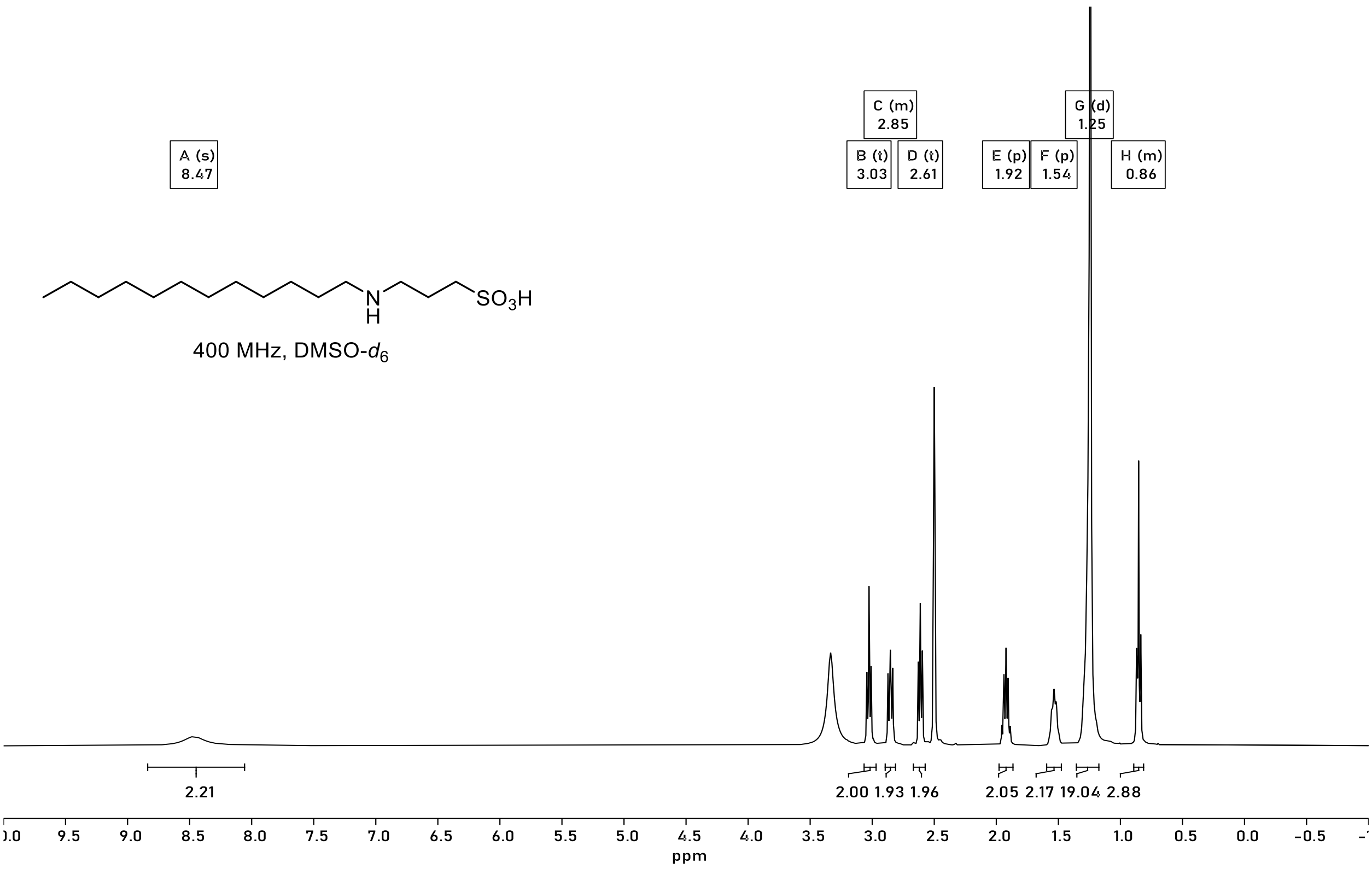

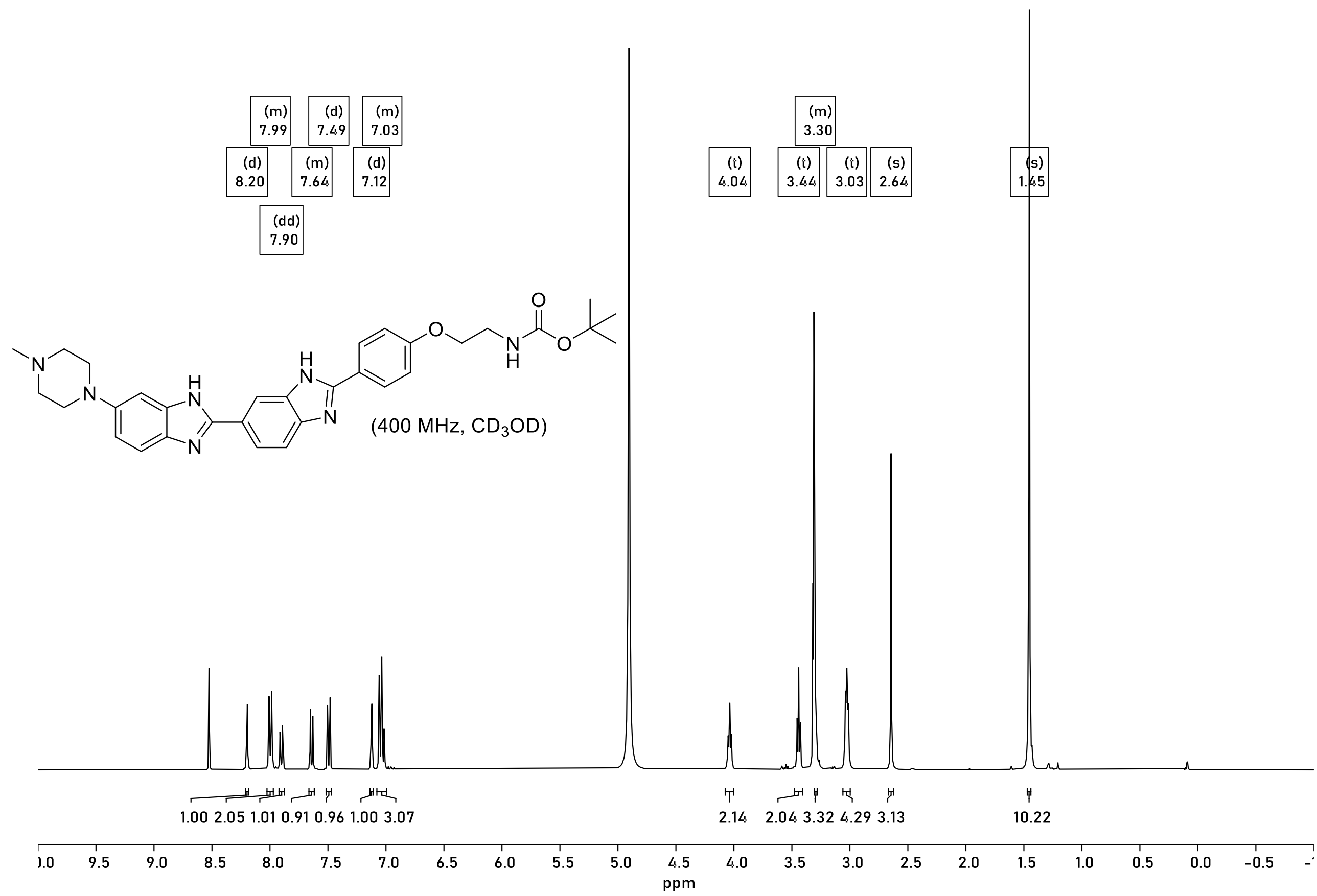

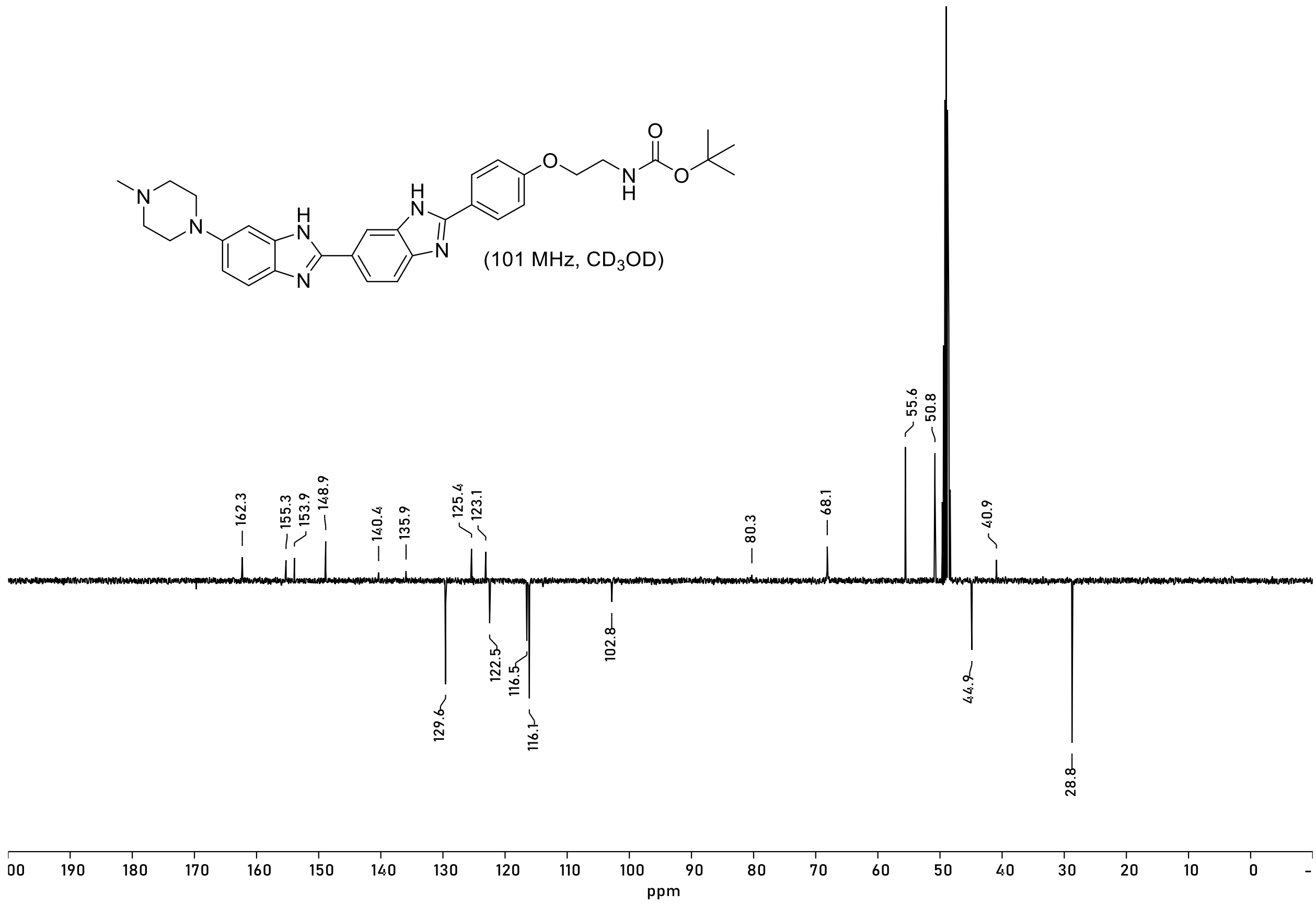
(101 MHz, CD3OD)
162.3
155.3
153.9
148.9
140.4
135.9
125.4
123.1
80.3
68.1
55.6
50.8
40.9
129.6
122.5
116.5
116.1
102.8
44.9
28.8
ppm

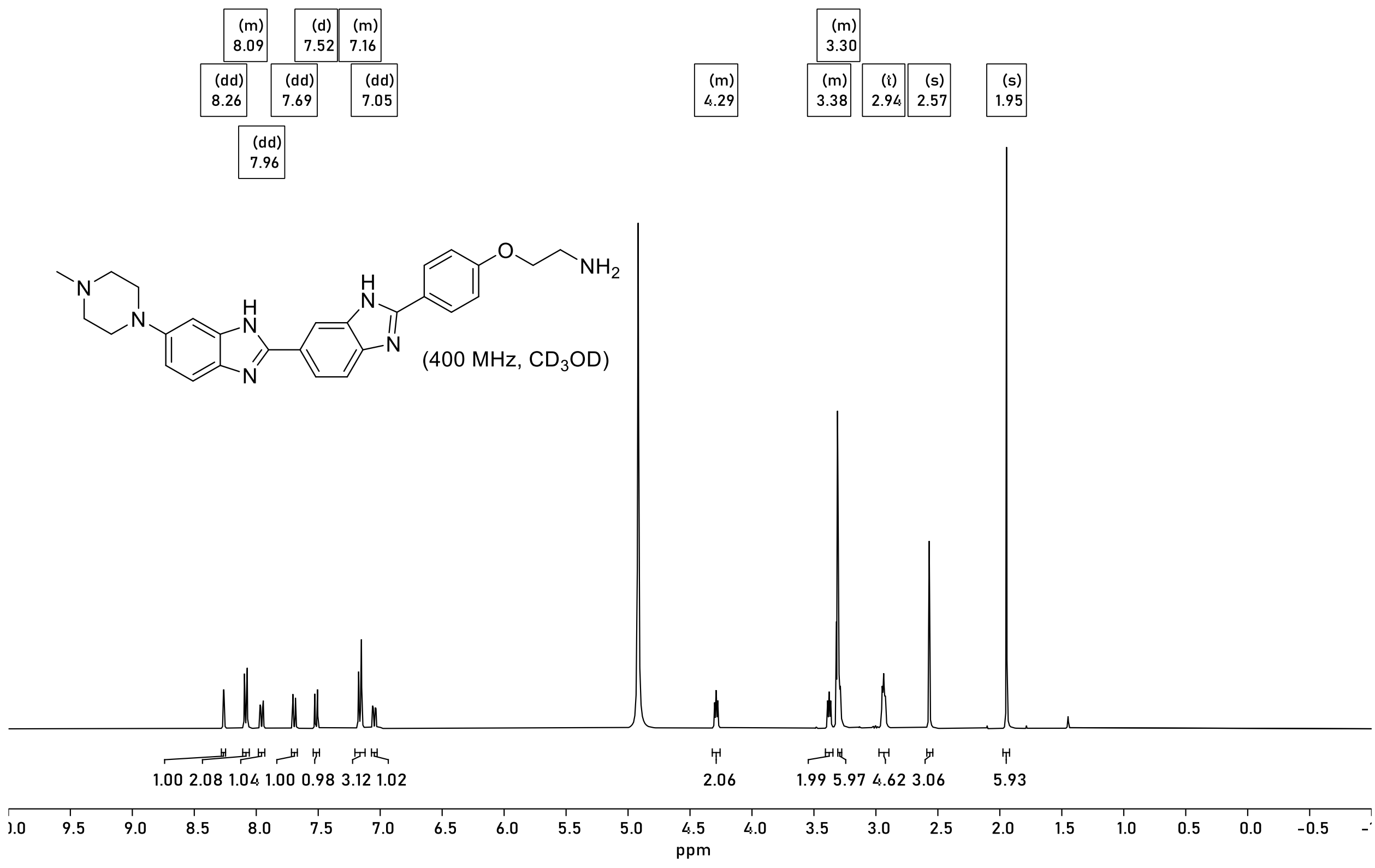
(m) 8.09
(d) 7.52
(m) 7.16
(dd) 8.26
(dd) 7.69
(dd) 7.05
(dd) 7.96
(m) 4.29
(m) 3.30
(m) 3.38
(t) 2.94
(s) 2.57
(s) 1.95
(400 MHz, CD3OD)
1.00 2.08 1.04 1.00 0.98 3.12 1.02
2.06
1.99 5.97 4.62 3.06
5.93
ppm

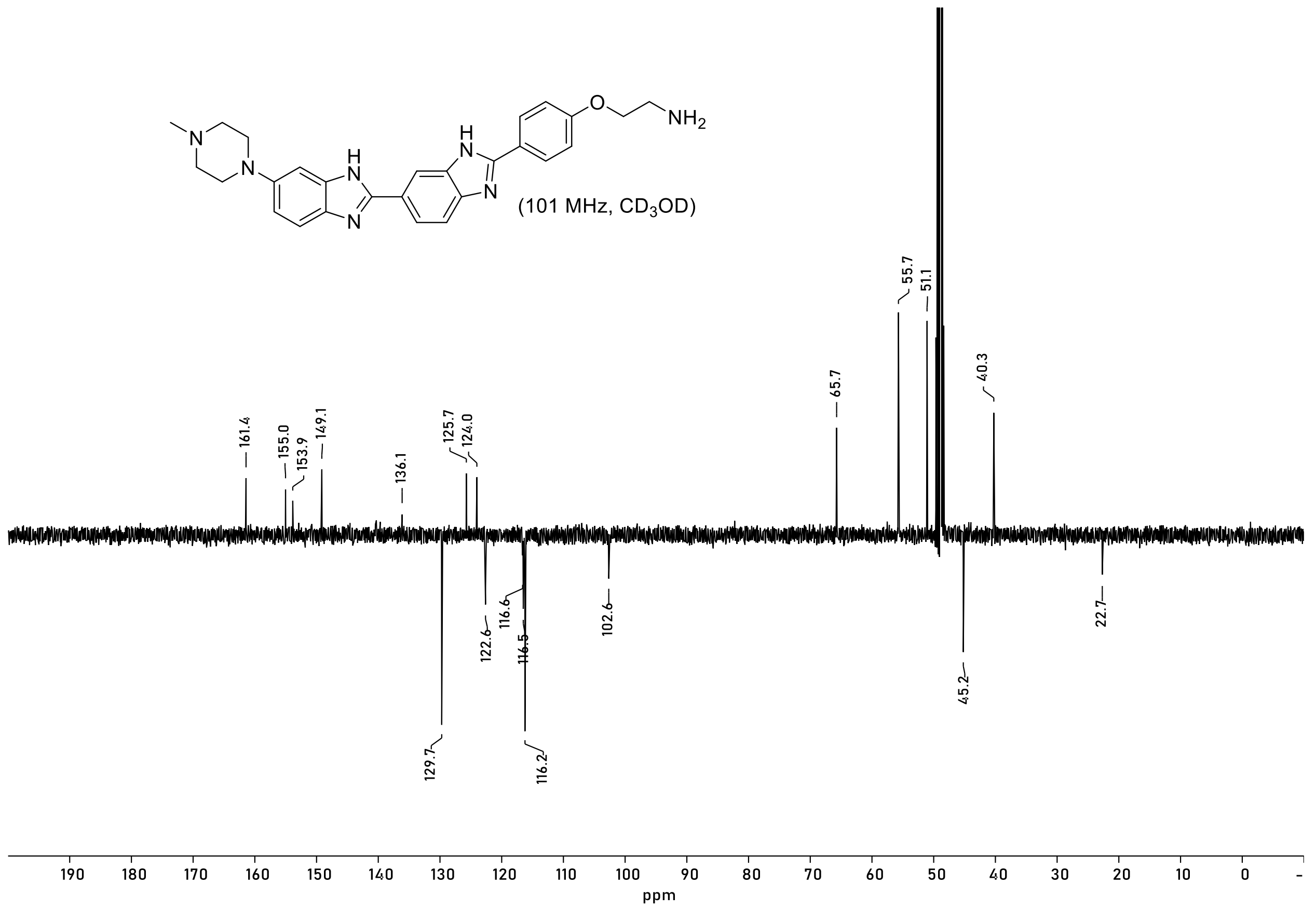

NH2
(101 MHz, CD3OD)
161.4
155.0
153.9
149.1
136.1
125.7
124.0
65.7
55.7
51.1
40.3
129.7
122.6
116.6
116.5
116.2
102.6
45.2
22.7
190 180 170 160 150 140 130 120 110 100 90 80 70 60 50 40 30 20 10 0
ppm

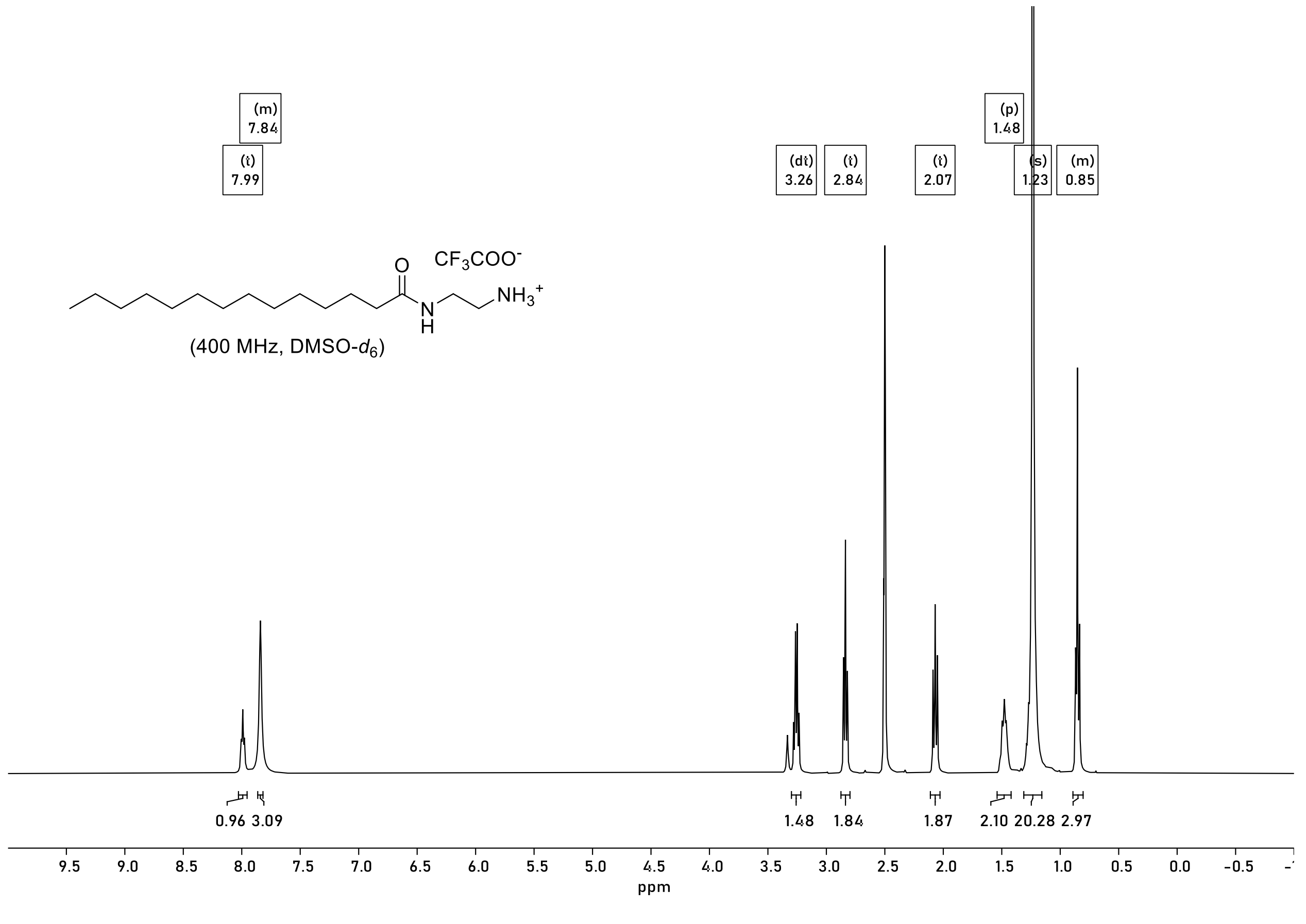

(m) 7.84
(t) 7.99
(dt) 3.26
(t) 2.84
(t) 2.07
(p) 1.48
(s) 1.23
(m) 0.85
O
CF3COO-
N
H
NH3+
(400 MHz, DMSO-d6)
0.96 3.09
1.48
1.84
1.87
2.10 20.28 2.97
9.5 9.0 8.5 8.0 7.5 7.0 6.5 6.0 5.5 5.0 4.5 4.0 3.5 3.0 2.5 2.0 1.5 1.0 0.5 0.0 -0.5
ppm

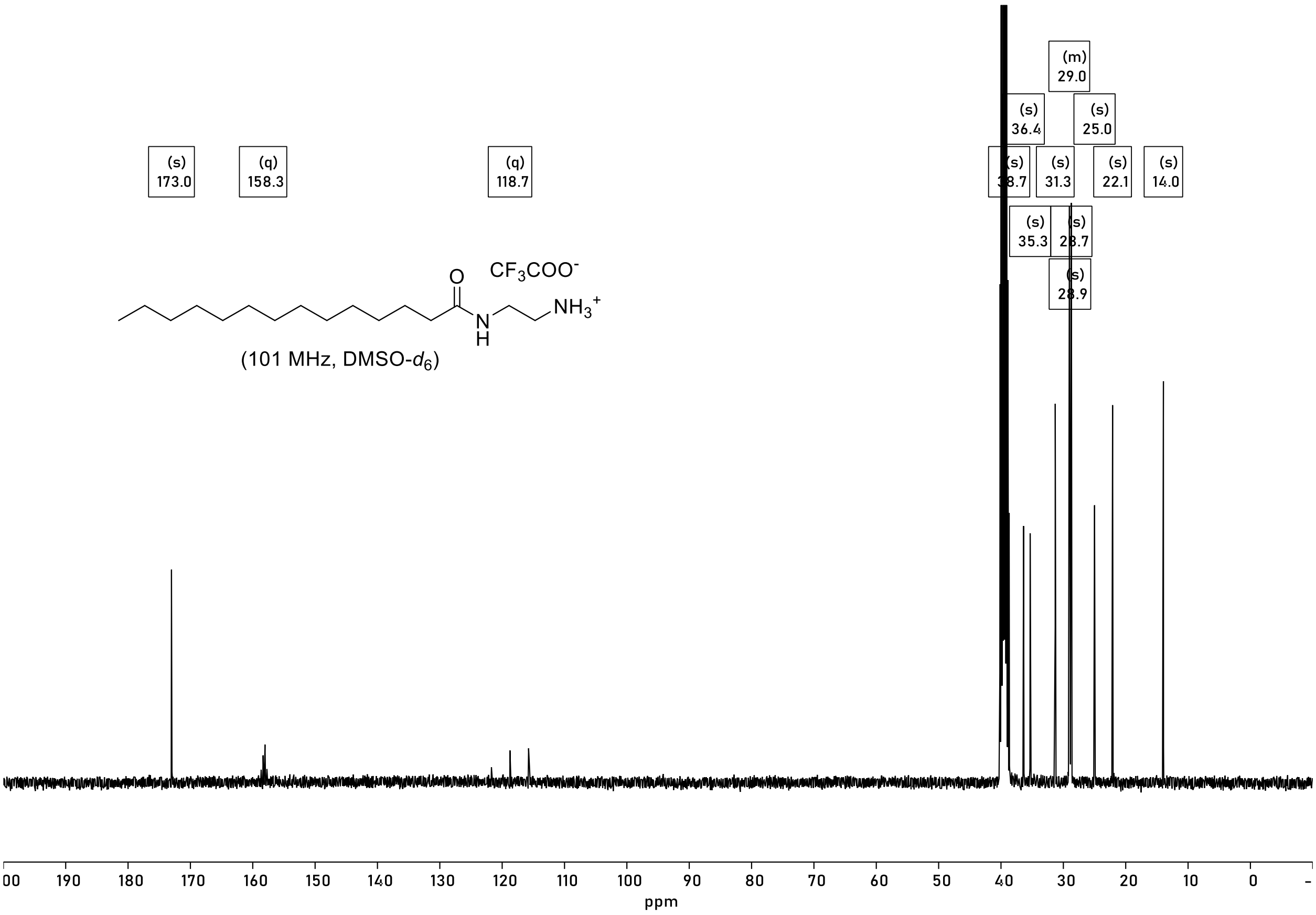
(s) 173.0
(q) 158.3
(q) 118.7
(m) 29.0
(s) 36.4
(s) 25.0
(s) 31.3
(s) 22.1
(s) 14.0
(s) 35.3
O
CF_3COO^-
N
H
NH_3^+
(101 MHz, DMSO-d_6)
ppm

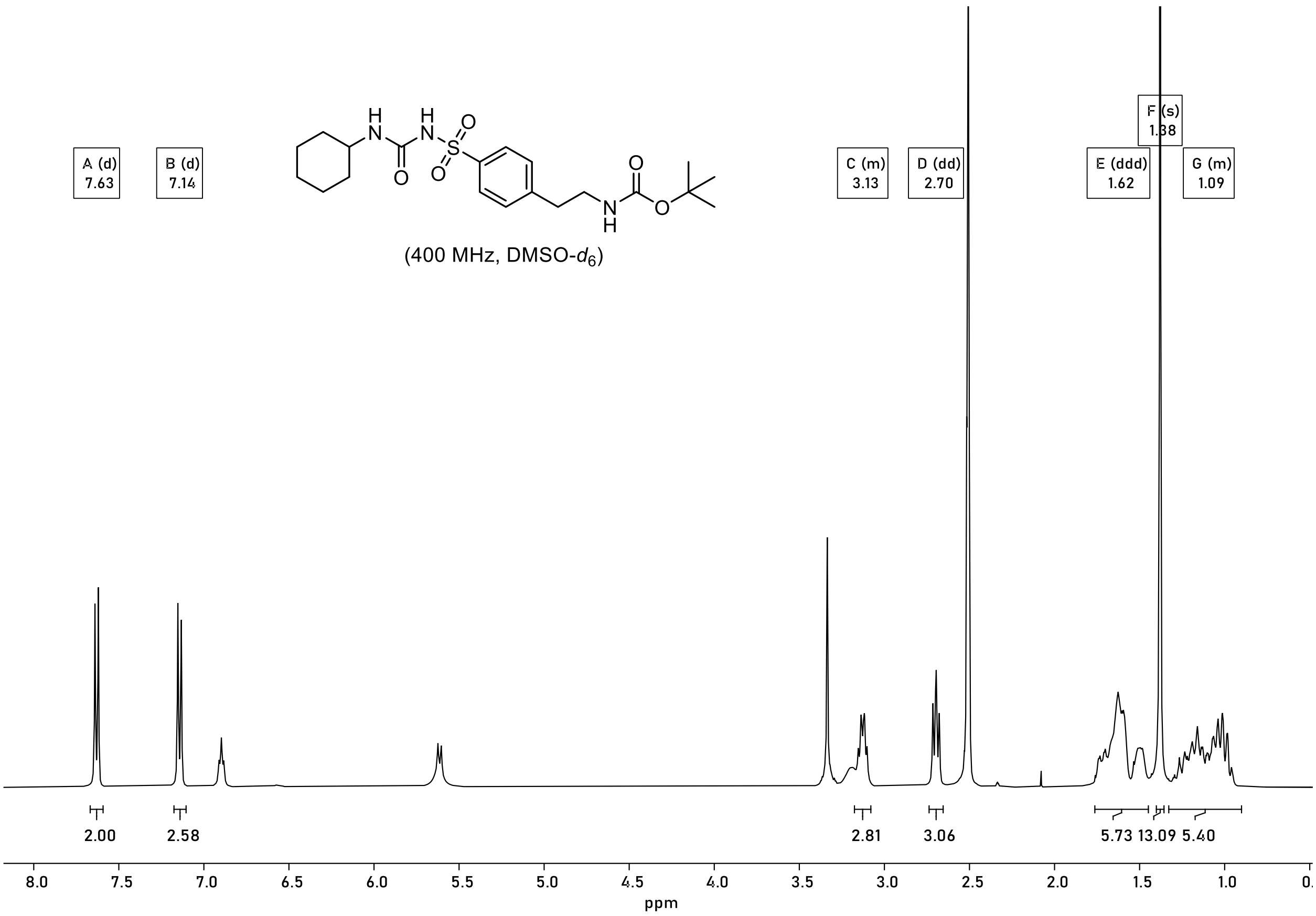
A (d) 7.63
B (d) 7.14
C (m) 3.13
D (dd) 2.70
E (ddd) 1.62
F (s) 1.38
G (m) 1.09
H
N
O
S
(400 MHz, DMSO-d_6)
2.00
2.58
2.81
3.06
5.73 13.09 5.40
ppm

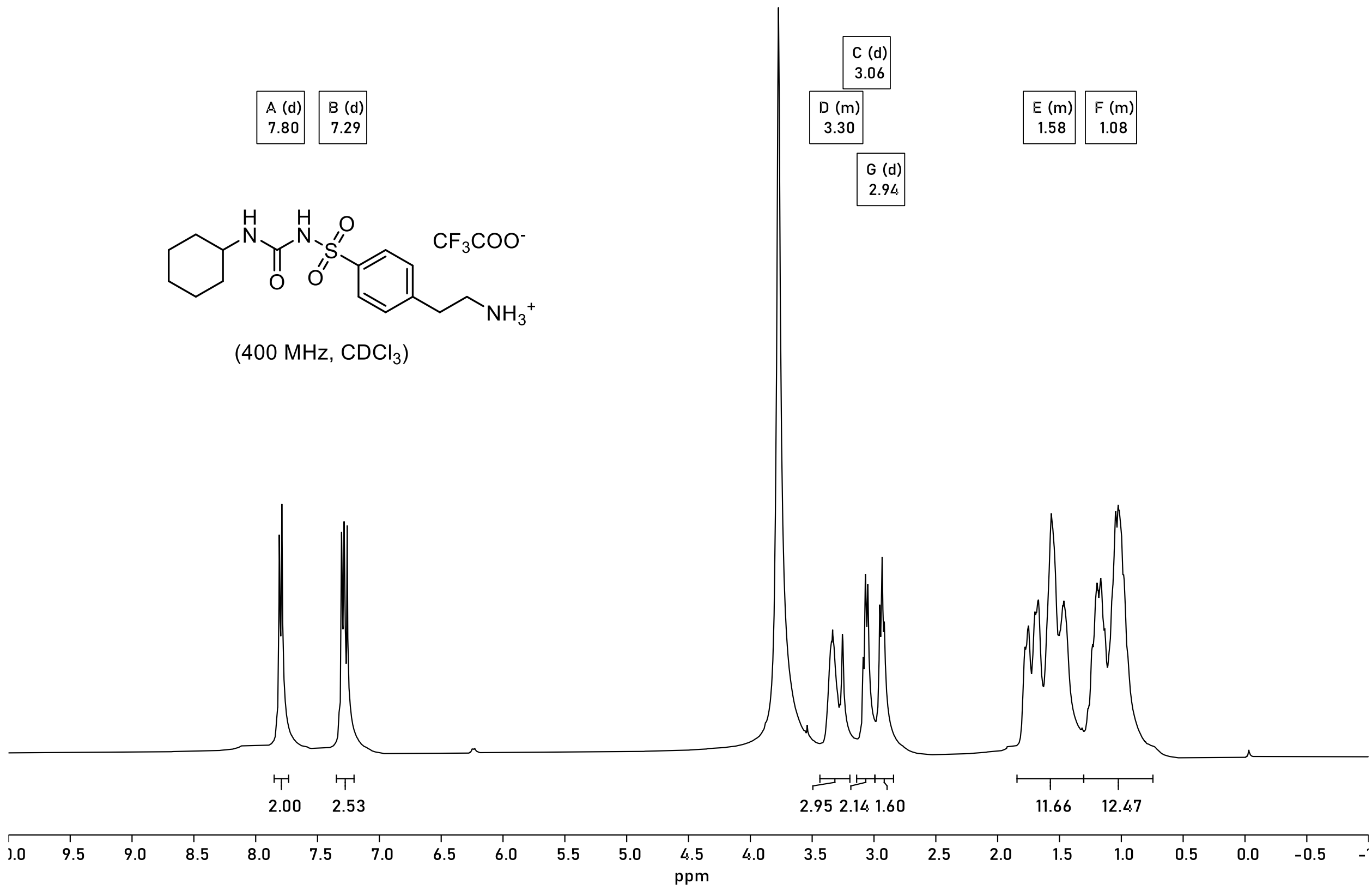
A (d)
7.80
B (d)
7.29
C (d)
3.06
D (m)
3.30
G (d)
2.94
E (m)
1.58
F (m)
1.08
CF3COO-
NH3+
(400 MHz, CDCl3)
2.00
2.53
2.95 2.14 1.60
11.66
12.47
ppm

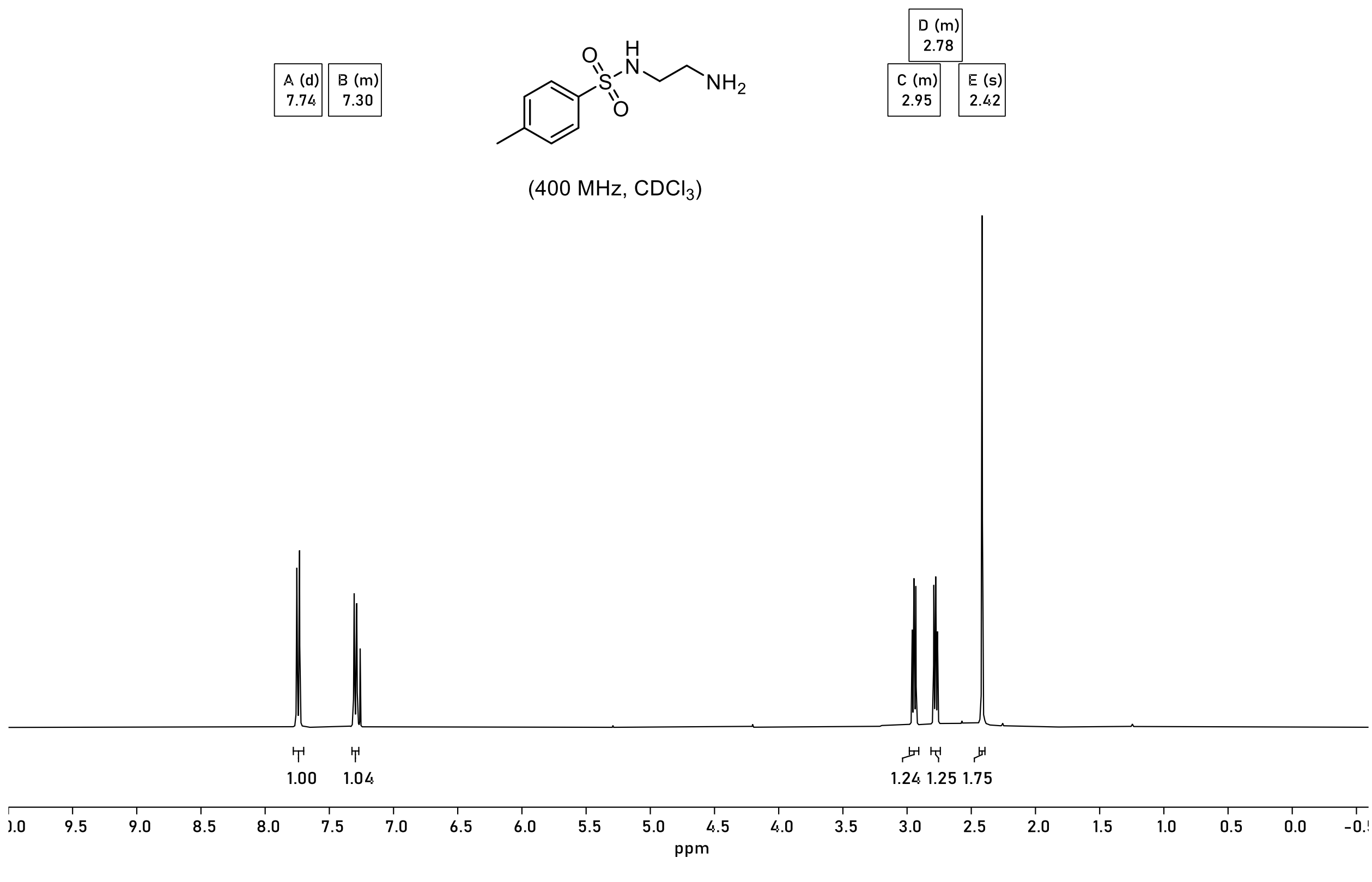
A (d)
7.74
B (m)
7.30
D (m)
2.78
C (m)
2.95
E (s)
2.42
NH2
(400 MHz, CDCl3)
1.00
1.04
1.24 1.25 1.75
ppm

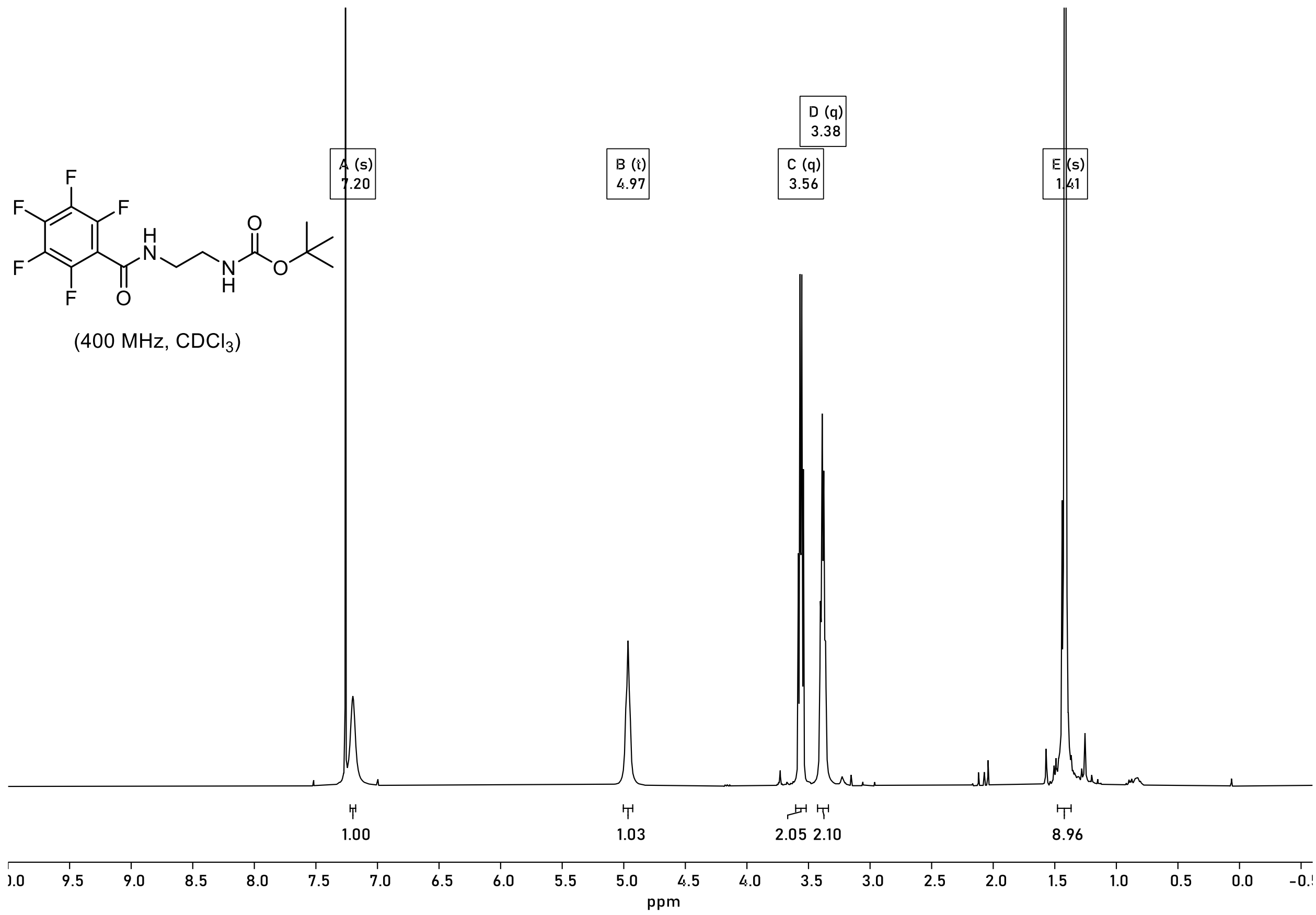
(400 MHz, CDCl3)
A (s)
7.20
B (t)
4.97
C (q)
3.56
D (q)
3.38
E (s)
1.41
1.00
1.03
2.05
2.10
8.96
ppm

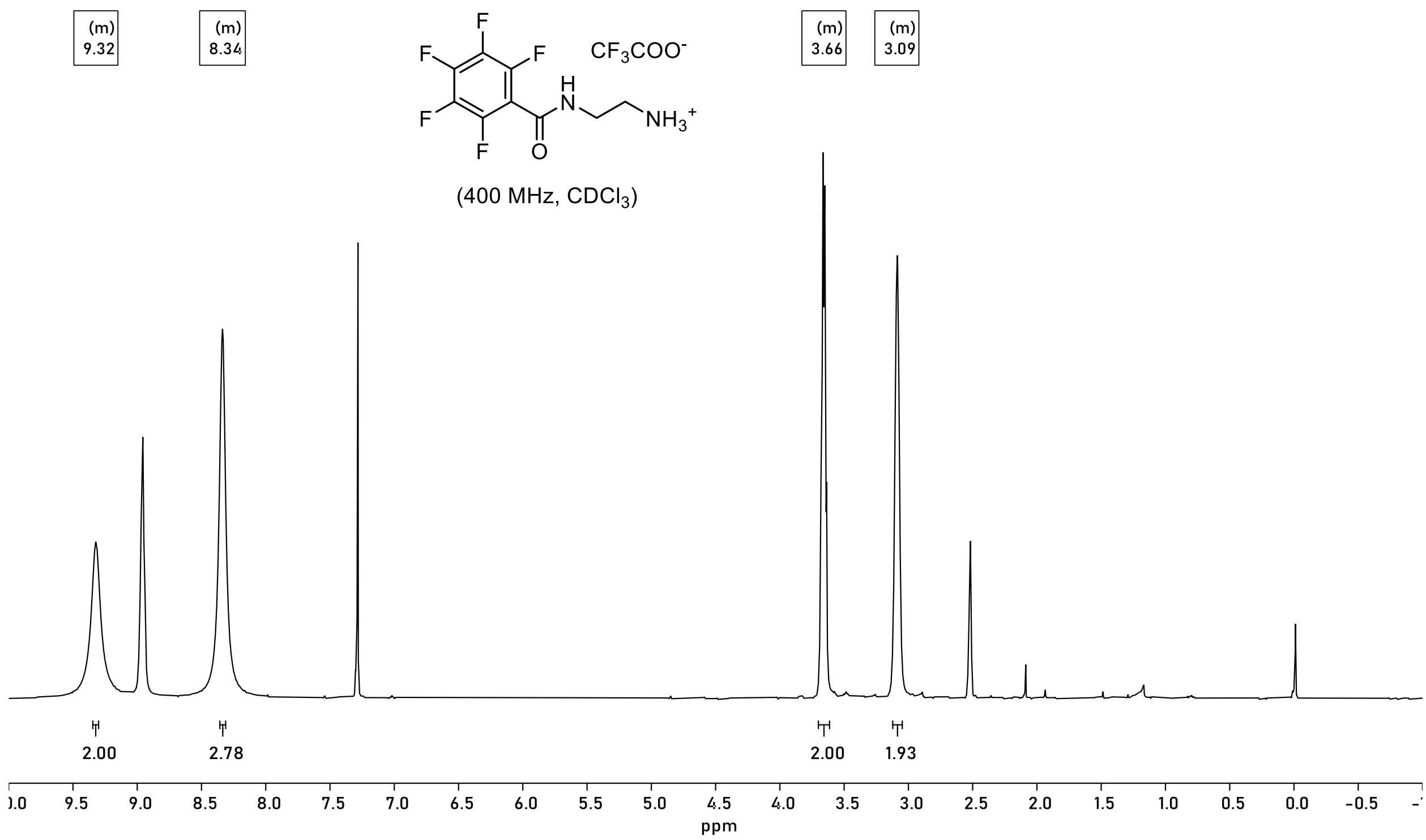
CF3COO-
NH3+
(400 MHz, CDCl3)
(m)
9.32
(m)
8.34
(m)
3.66
(m)
3.09
2.00
2.78
2.00
1.93
ppm

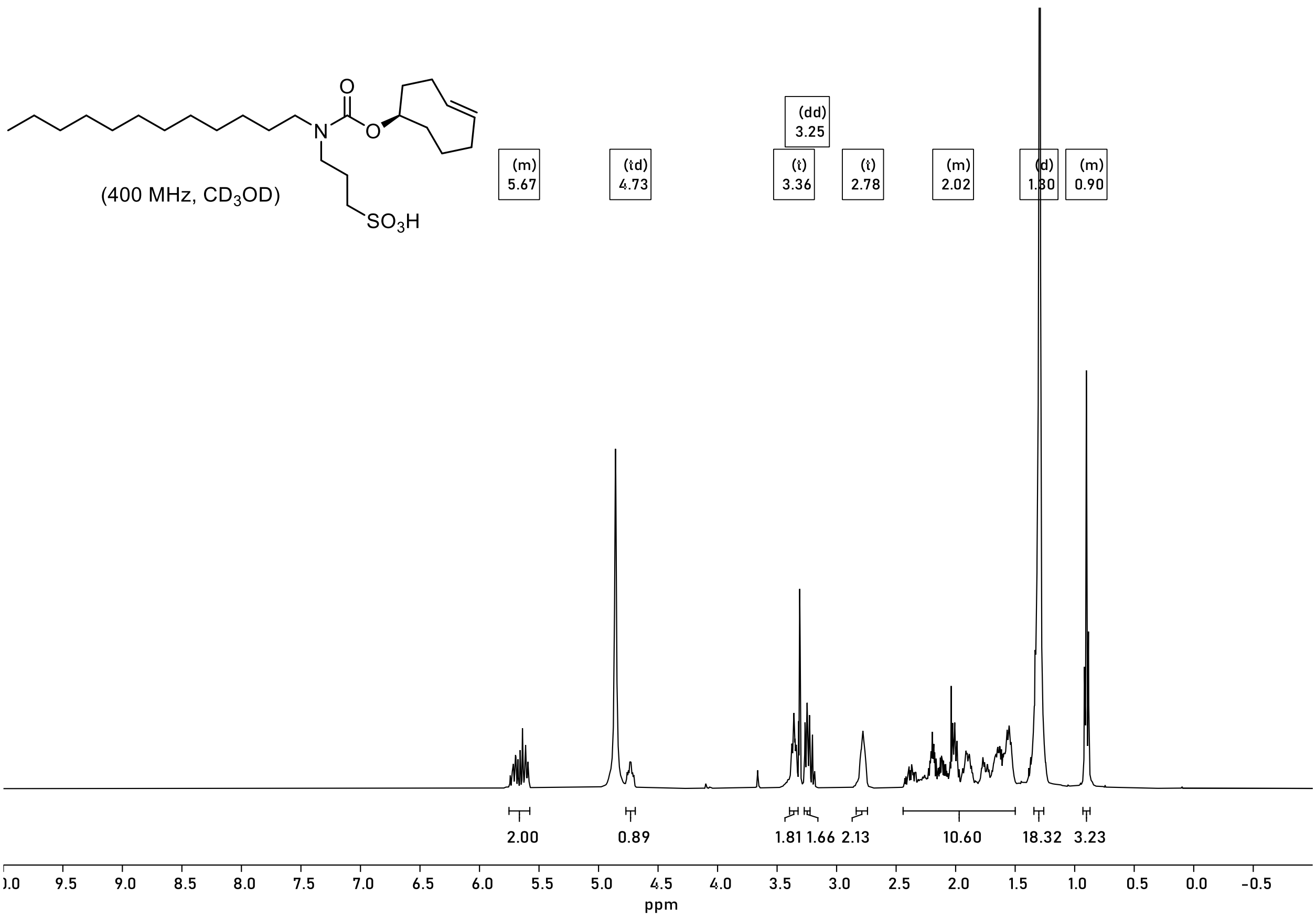
(400 MHz, CD_3OD)
SO_3H
(m) 5.67
(td) 4.73
(dd) 3.25
(t) 3.36
(t) 2.78
(m) 2.02
(d) 1.30
(m) 0.90
2.00
0.89
1.81
1.66
2.13
10.60
18.32
3.23
ppm

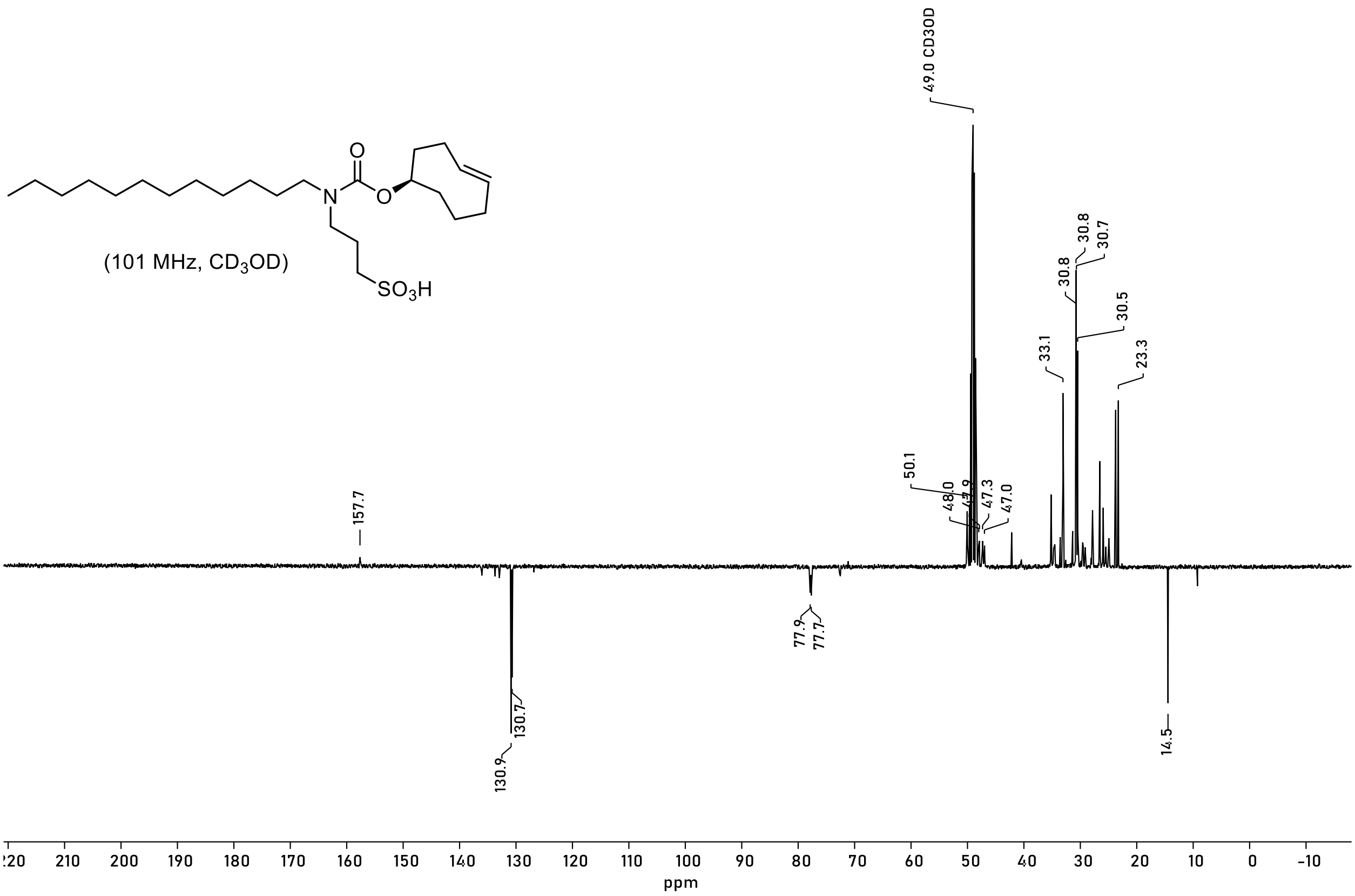
(101 MHz, CD_3OD)
SO_3H
49.0 CD3OD
157.7
130.9
130.7
77.9
77.7
50.1
48.0
47.3
47.0
33.1
30.8
30.8
30.7
30.5
23.3
14.5
ppm

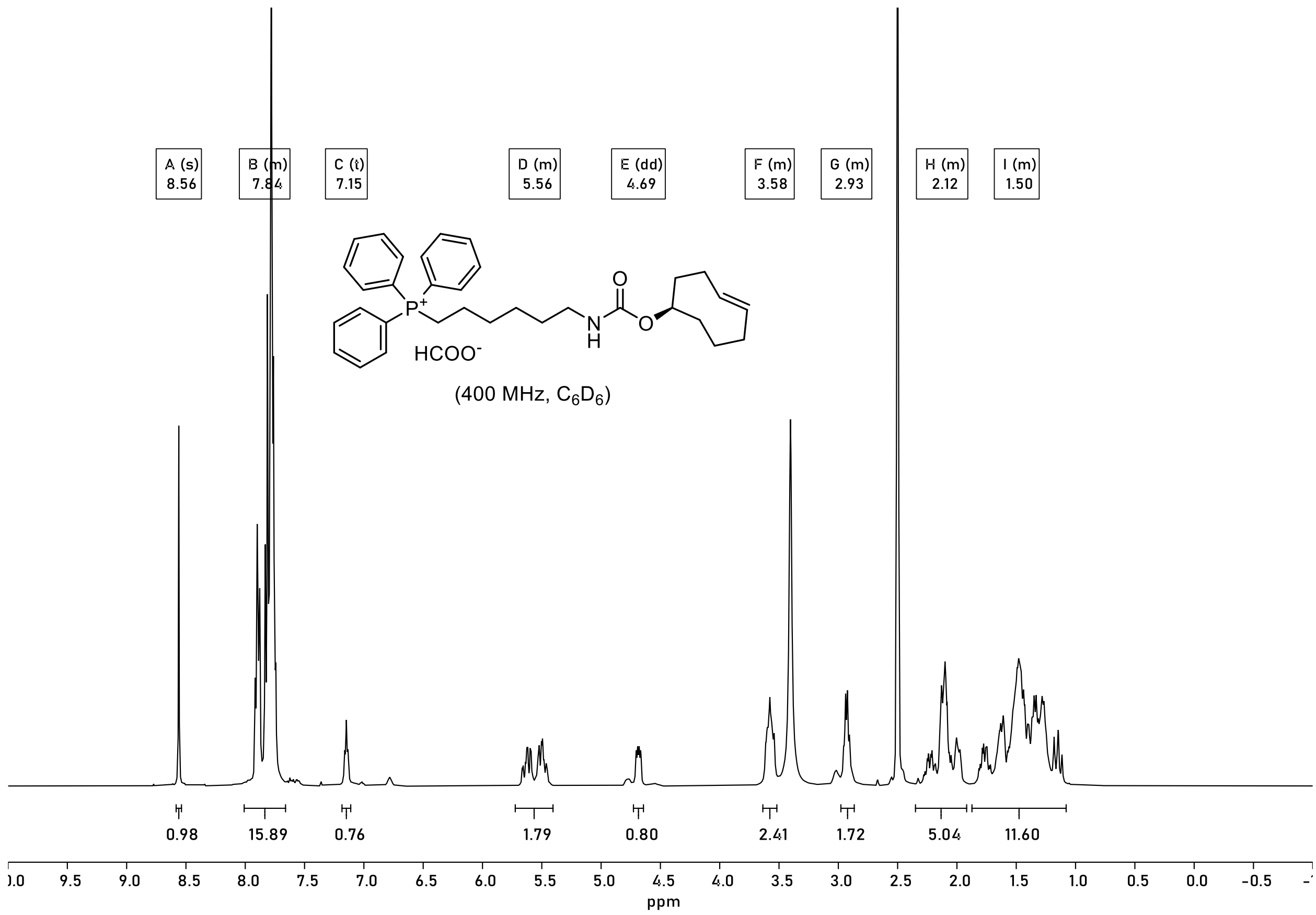
A (s)
8.56
B (m)
7.84
C (t)
7.15
D (m)
5.56
E (dd)
4.69
F (m)
3.58
G (m)
2.93
H (m)
2.12
I (m)
1.50
HCOO-
(400 MHz, C6D6)
0.98
15.89
0.76
1.79
0.80
2.41
1.72
5.04
11.60
ppm

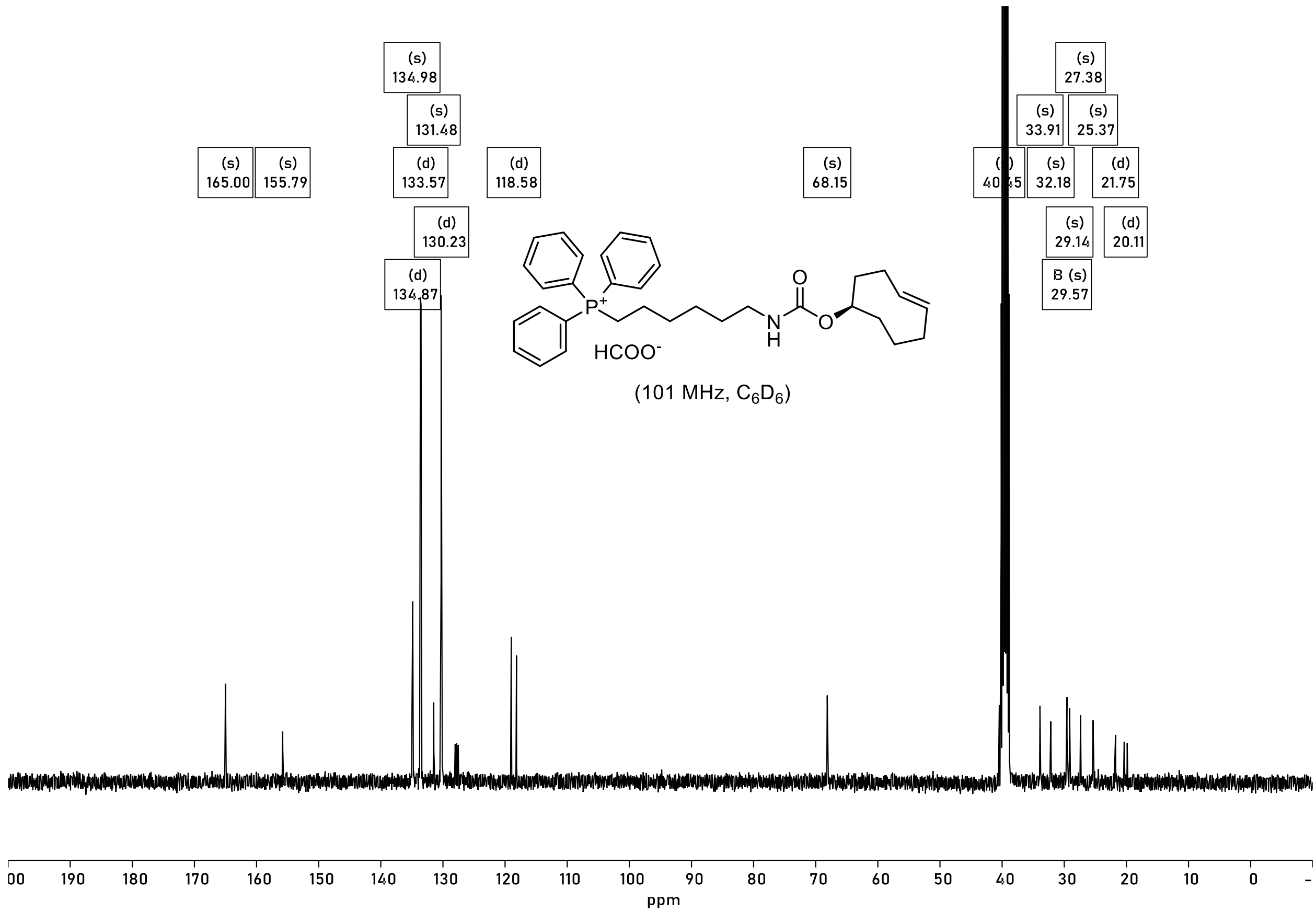
(s)
134.98
(s)
131.48
(s)
165.00
(s)
155.79
(d)
133.57
(d)
118.58
(d)
130.23
(d)
134.87
(s)
68.15
(s)
27.38
(s)
33.91
(s)
25.37
(s)
32.18
(d)
21.75
(s)
29.14
(d)
20.11
B (s)
29.57
HCOO-
(101 MHz, C6D6)
ppm

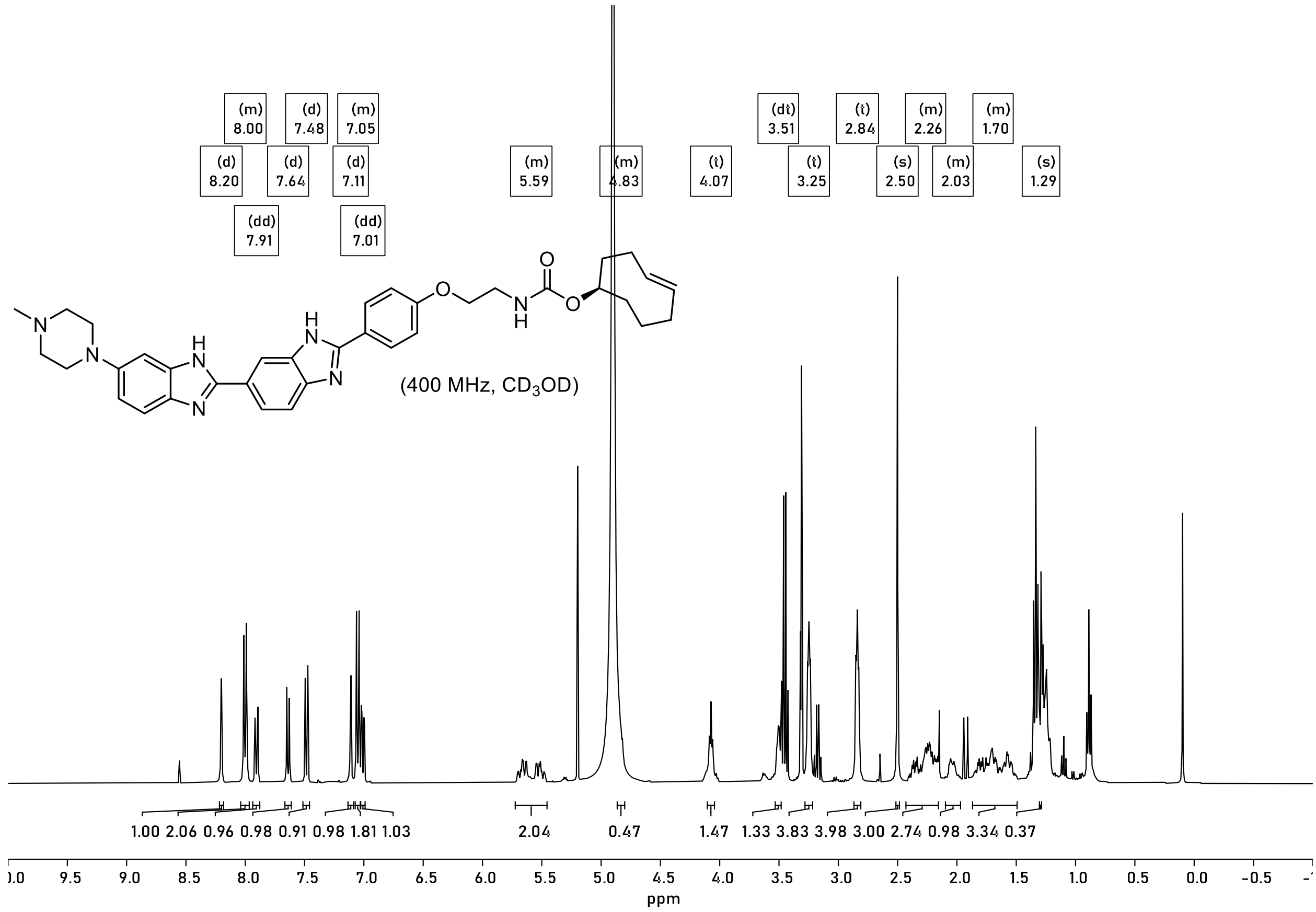
(400 MHz, CD3OD)
ppm

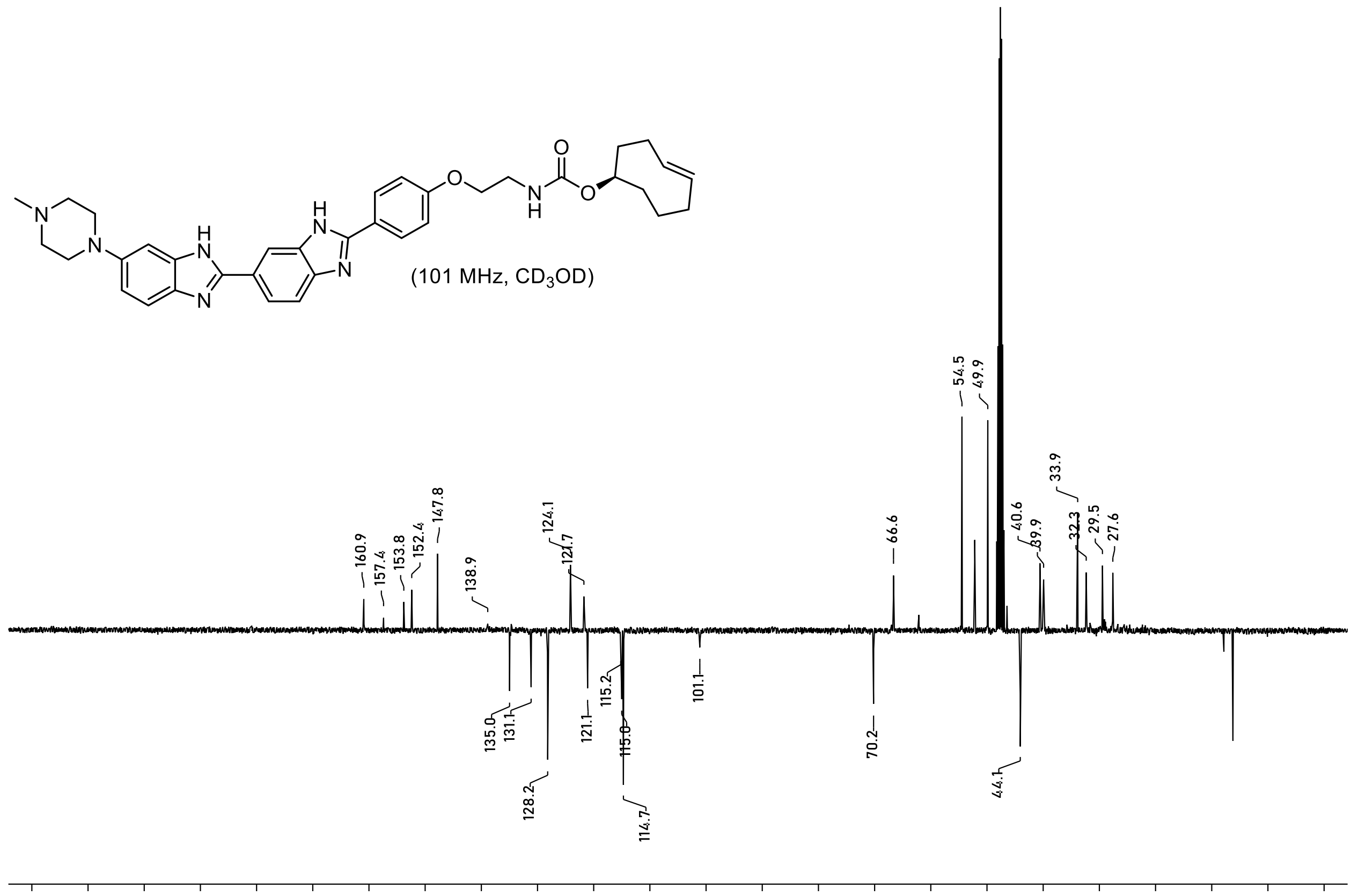
(101 MHz, CD3OD)
ppm

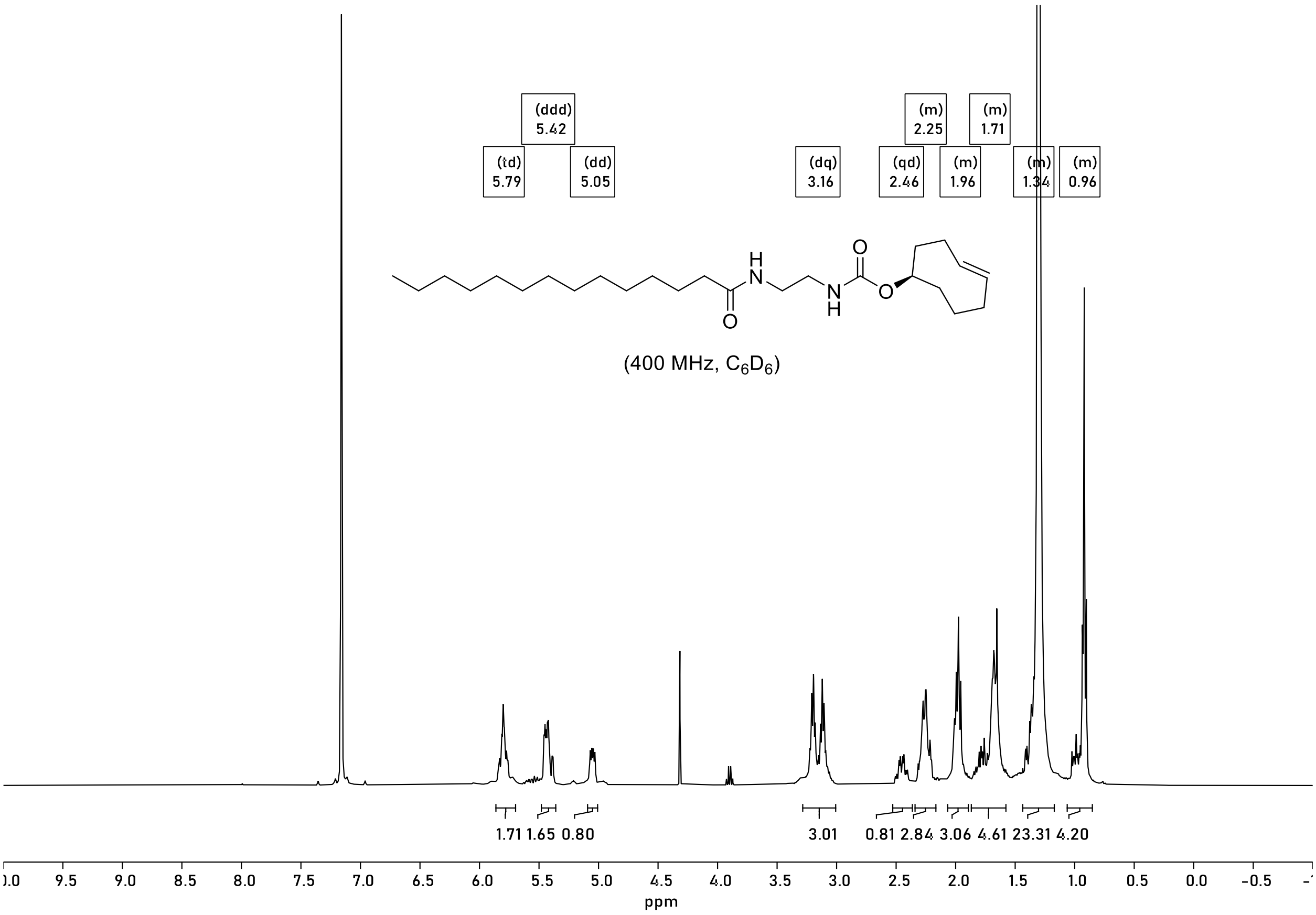
(ddd)
5.42
(td)
5.79
(dd)
5.05
(dq)
3.16
(m)
2.25
(m)
1.71
(qd)
2.46
(m)
1.96
(m)
1.34
(m)
0.96
(400 MHz, C_6D_6)
1.71 1.65 0.80
3.01
0.81 2.84 3.06 4.61 23.31 4.20
ppm

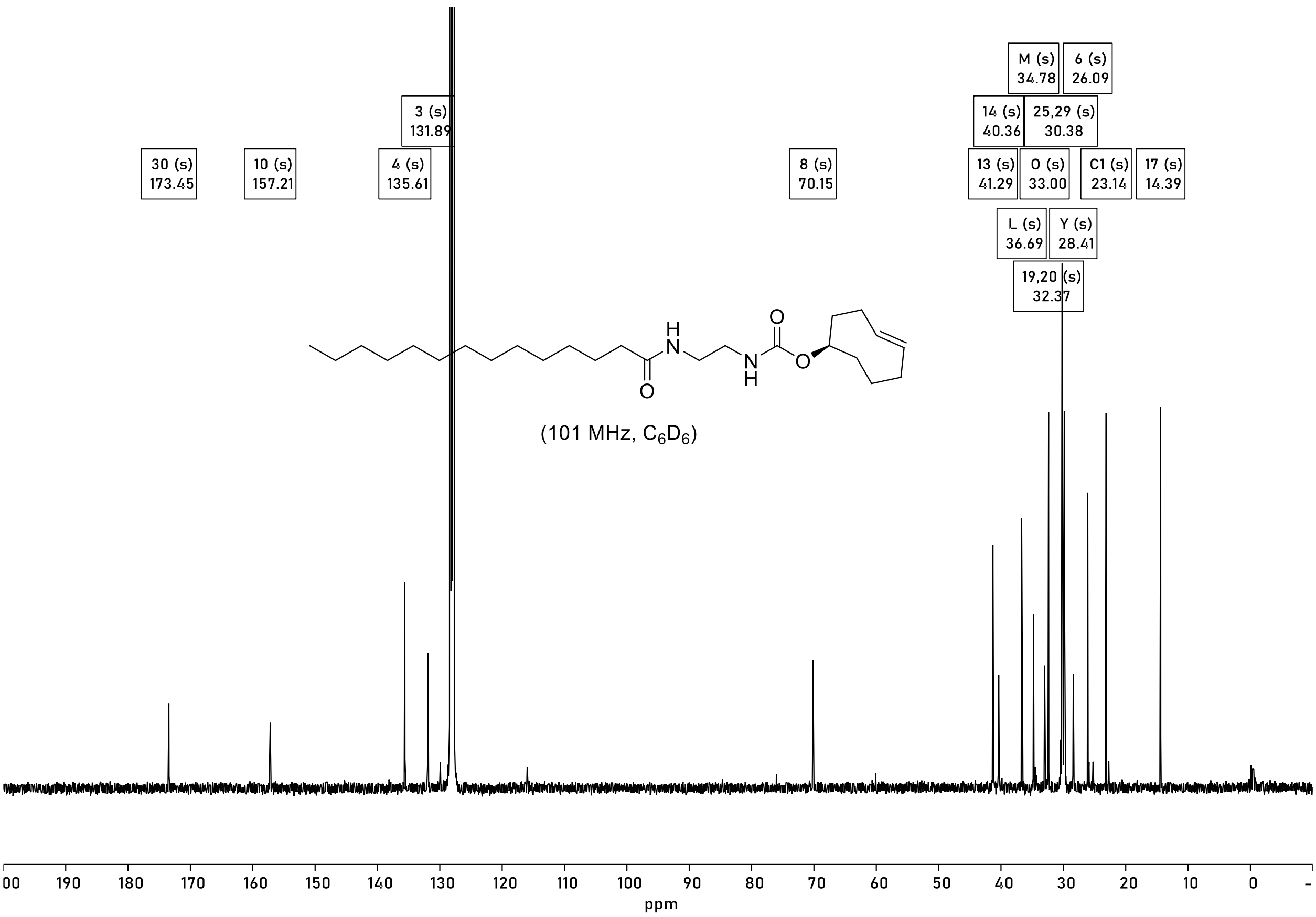
M (s)
34.78
6 (s)
26.09
3 (s)
131.89
14 (s)
40.36
25,29 (s)
30.38
30 (s)
173.45
10 (s)
157.21
4 (s)
135.61
8 (s)
70.15
13 (s)
41.29
O (s)
33.00
C1 (s)
23.14
17 (s)
14.39
L (s)
36.69
Y (s)
28.41
19,20 (s)
32.37
(101 MHz, C_6D_6)
ppm

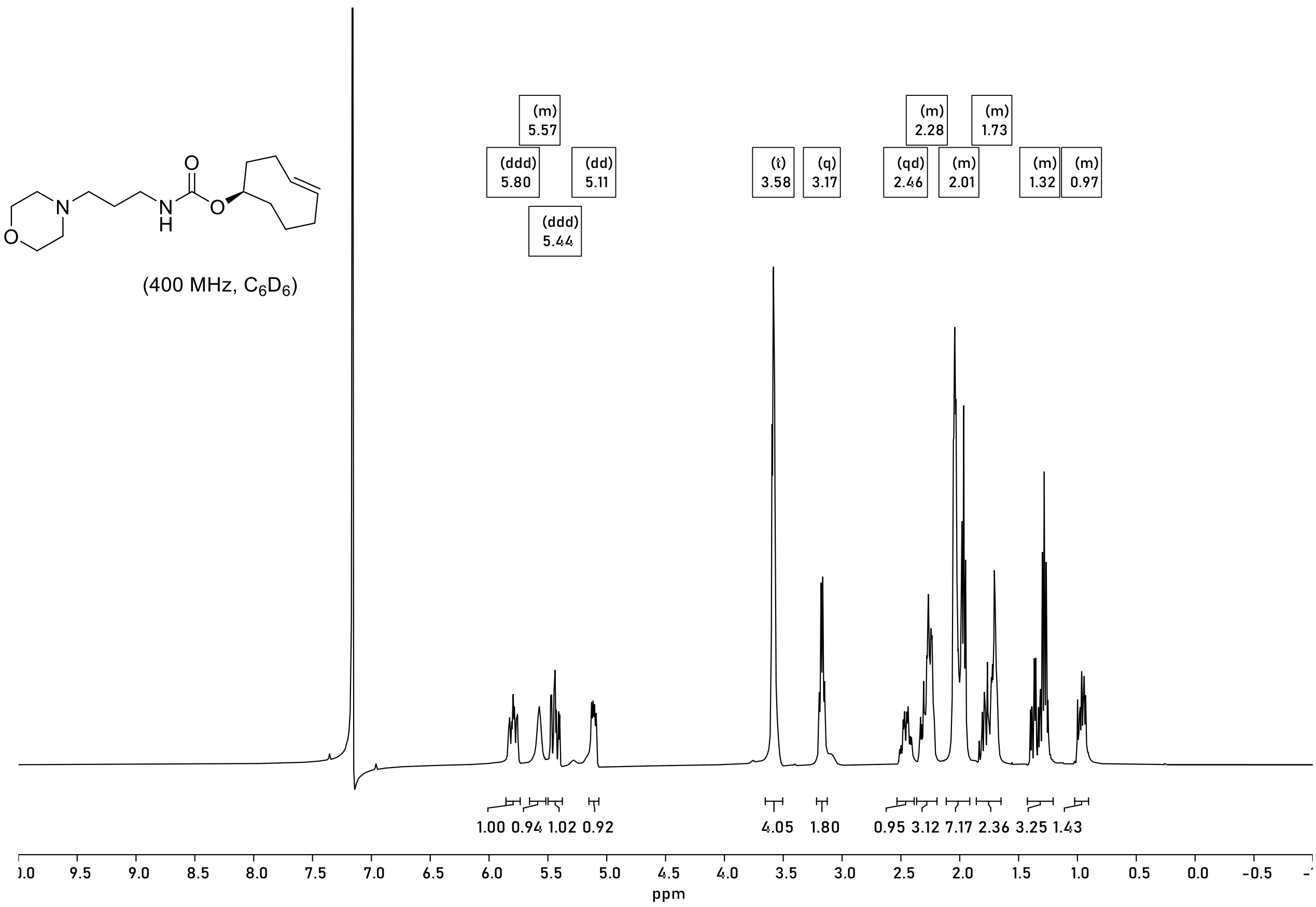
(400 MHz, C6D6)
(m) 5.57
(ddd) 5.80
(dd) 5.11
(ddd) 5.44
(t) 3.58
(q) 3.17
(m) 2.28
(m) 1.73
(qd) 2.46
(m) 2.01
(m) 1.32
(m) 0.97
1.00 0.94 1.02 0.92
4.05 1.80
0.95 3.12 7.17 2.36 3.25 1.43
ppm

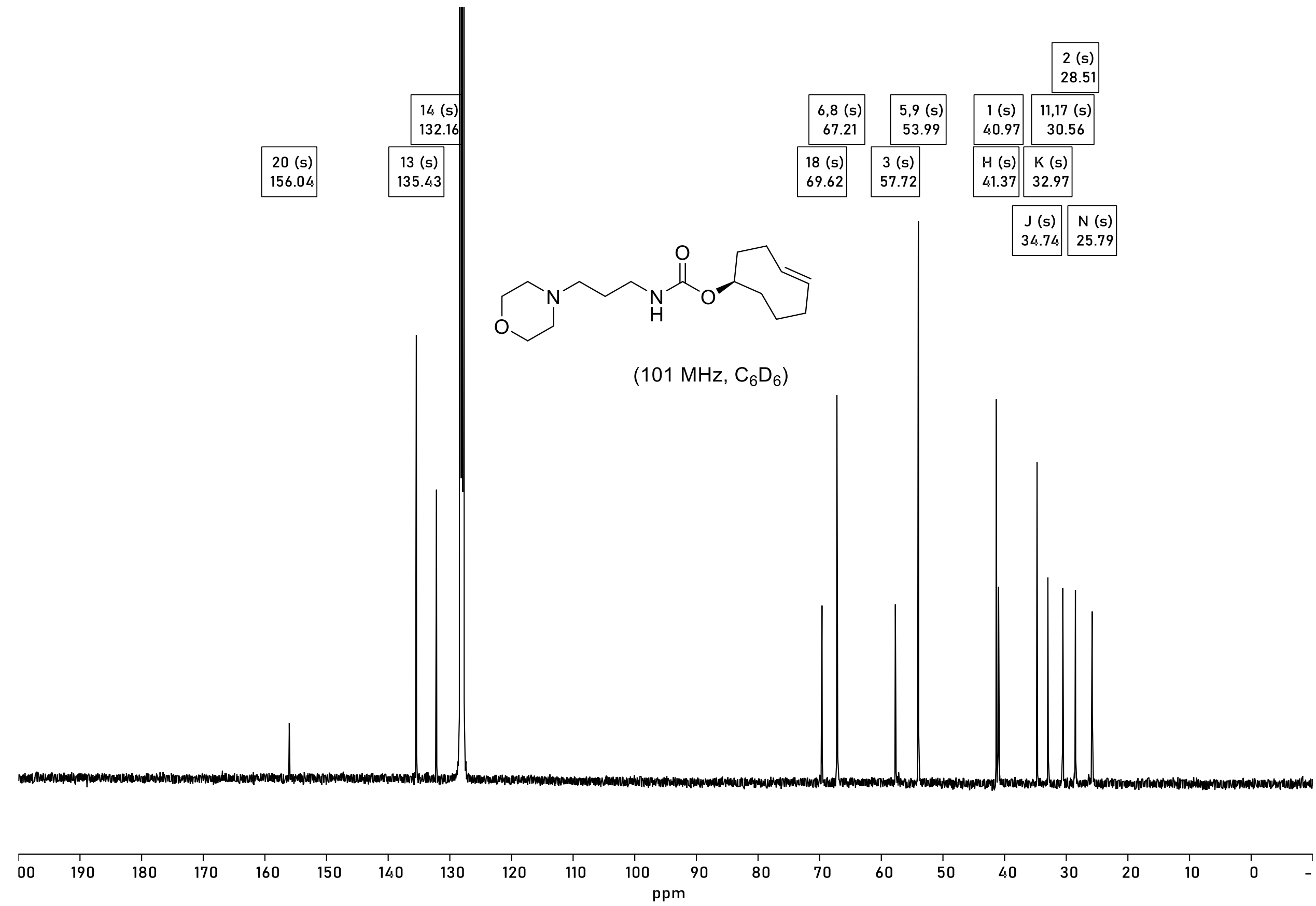
2 (s) 28.51
14 (s) 132.16
6,8 (s) 67.21
5,9 (s) 53.99
1 (s) 40.97
11,17 (s) 30.56
20 (s) 156.04
13 (s) 135.43
18 (s) 69.62
3 (s) 57.72
H (s) 41.37
K (s) 32.97
J (s) 34.74
N (s) 25.79
(101 MHz, C6D6)
ppm

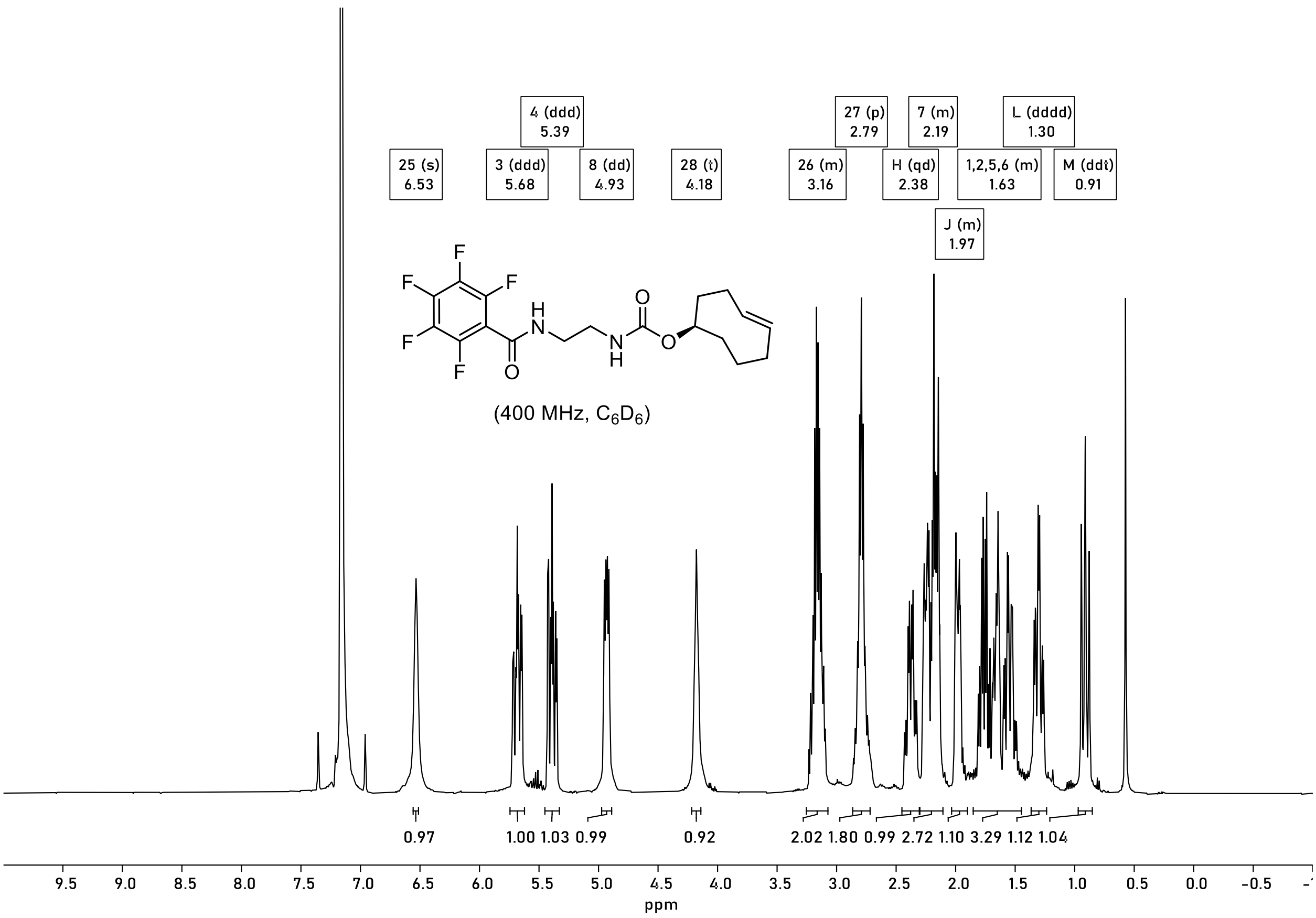
4 (ddd) 5.39
27 (p) 2.79
7 (m) 2.19
L (dddd) 1.30
25 (s) 6.53
3 (ddd) 5.68
8 (dd) 4.93
28 (t) 4.18
26 (m) 3.16
H (qd) 2.38
1,2,5,6 (m) 1.63
M (ddt) 0.91
J (m) 1.97
(400 MHz, C6D6)
0.97
1.00 1.03 0.99
0.92
2.02 1.80 0.99 2.72 1.10 3.29 1.12 1.04
ppm

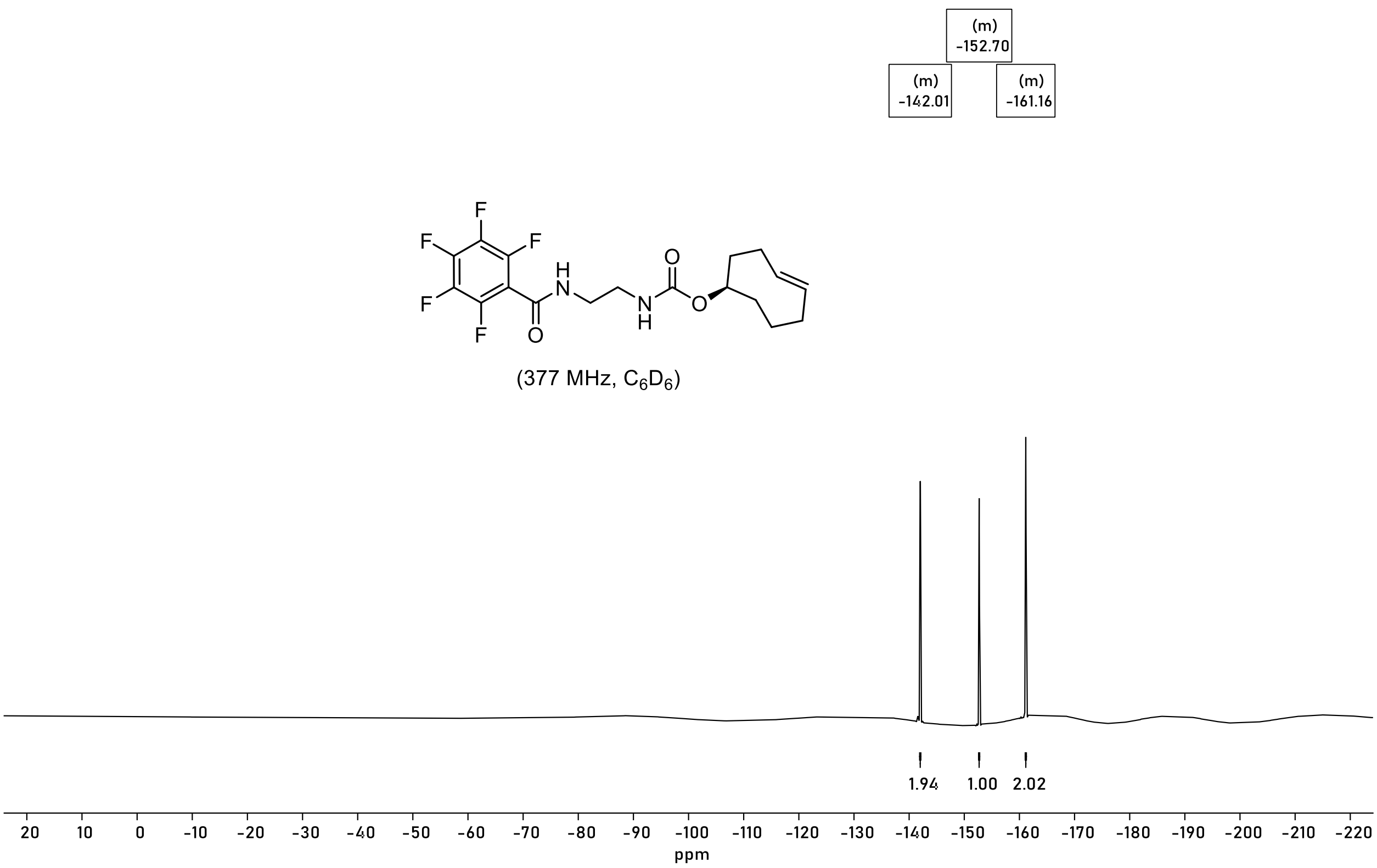
(m) -152.70
(m) -142.01
(m) -161.16
(377 MHz, C6D6)
1.94 1.00 2.02
ppm

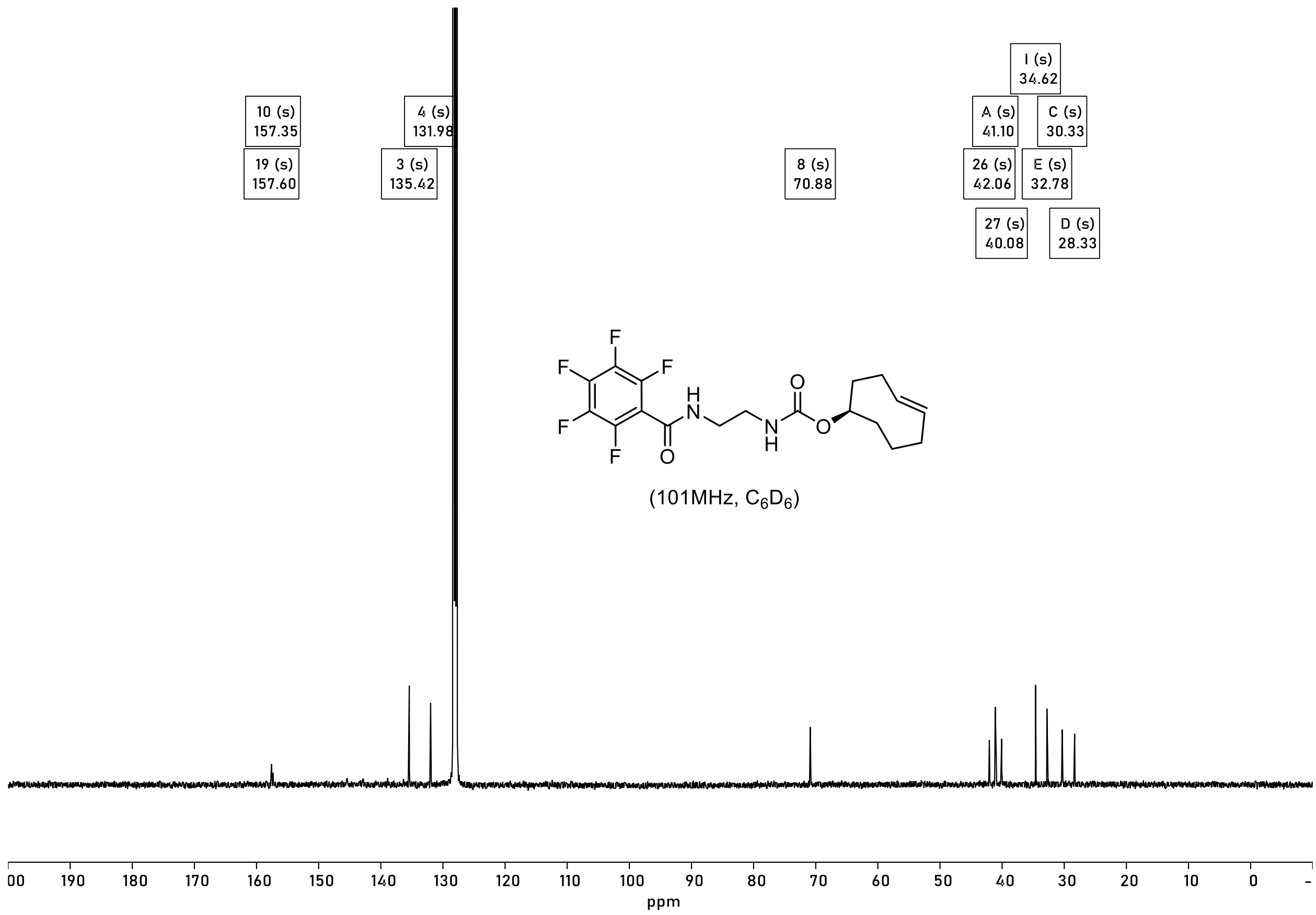

10 (s)
157.35
19 (s)
157.60
4 (s)
131.98
3 (s)
135.42
8 (s)
70.88
I (s)
34.62
A (s)
41.10
C (s)
30.33
26 (s)
42.06
E (s)
32.78
27 (s)
40.08
D (s)
28.33
(101MHz, C_6D_6)
ppm

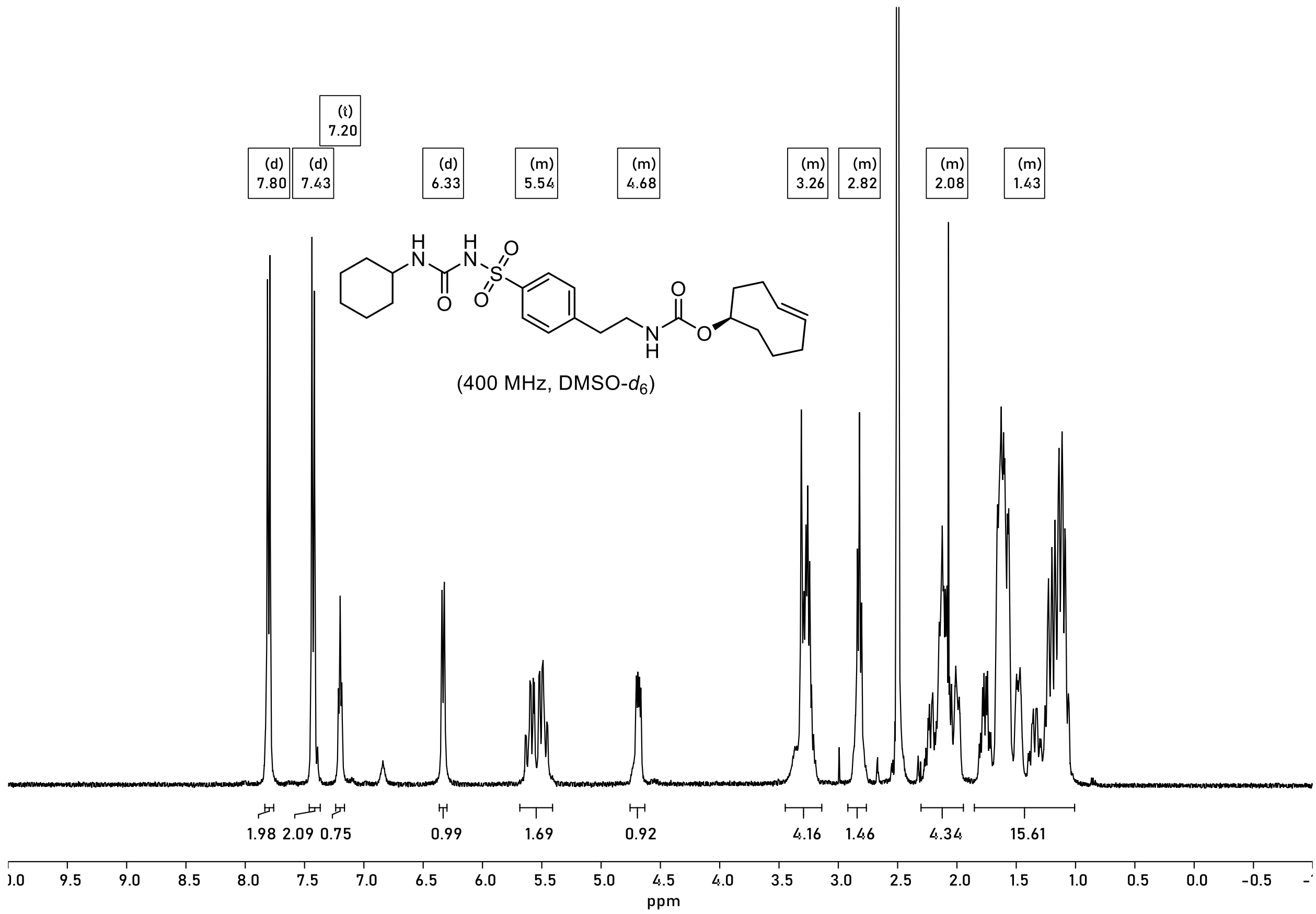

(t)
7.20
(d)
7.80
(d)
7.43
(d)
6.33
(m)
5.54
(m)
4.68
(m)
3.26
(m)
2.82
(m)
2.08
(m)
1.43
(400 MHz, DMSO-d_6)
1.98 2.09 0.75
0.99
1.69
0.92
4.16
1.46
4.34
15.61
ppm

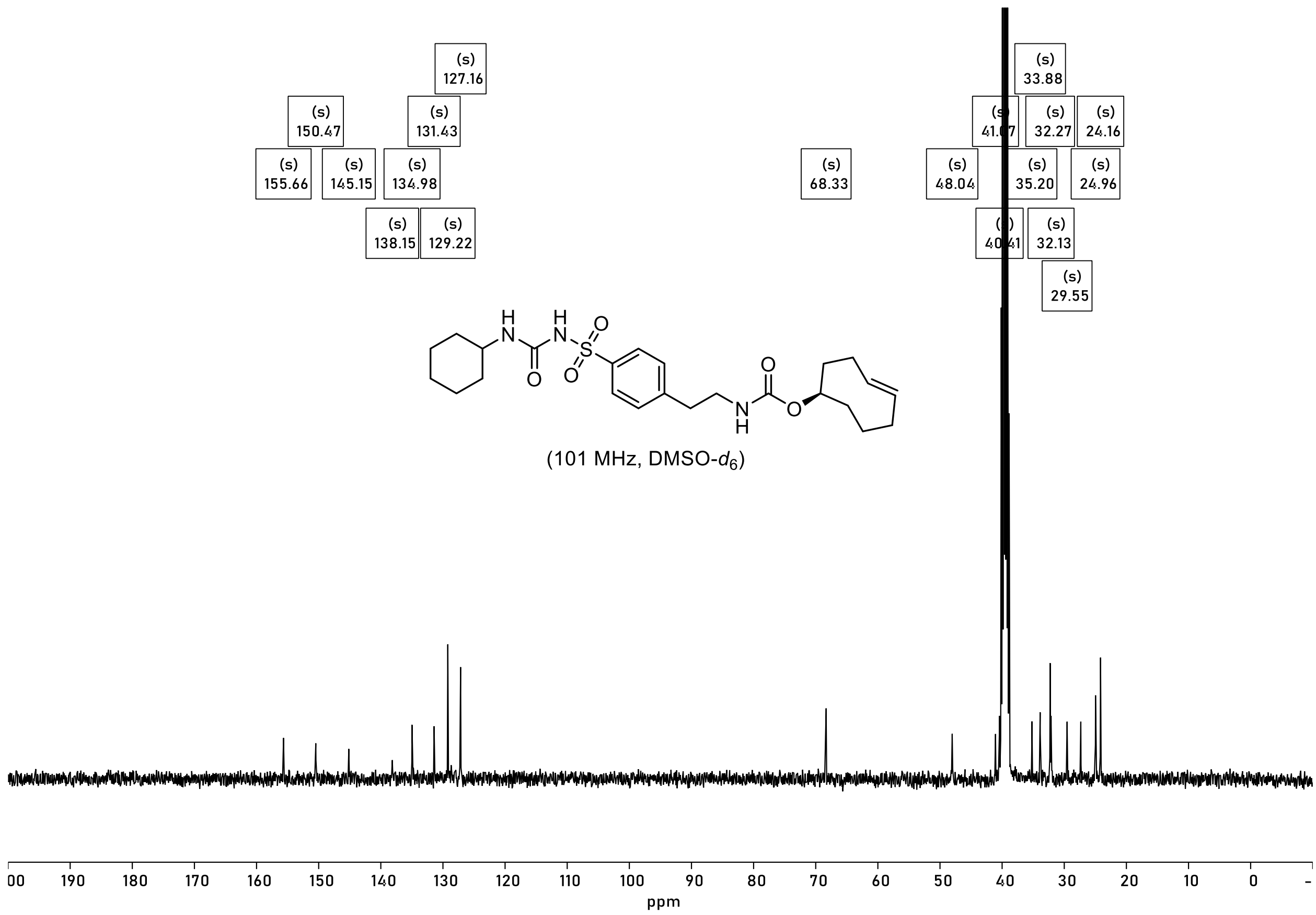

155.66
150.47
145.15
138.15
134.98
131.43
129.22
127.16
68.33
48.04
35.20
33.88
32.27
32.13
29.55
24.96
24.16
(101 MHz, DMSO-d_6)
ppm

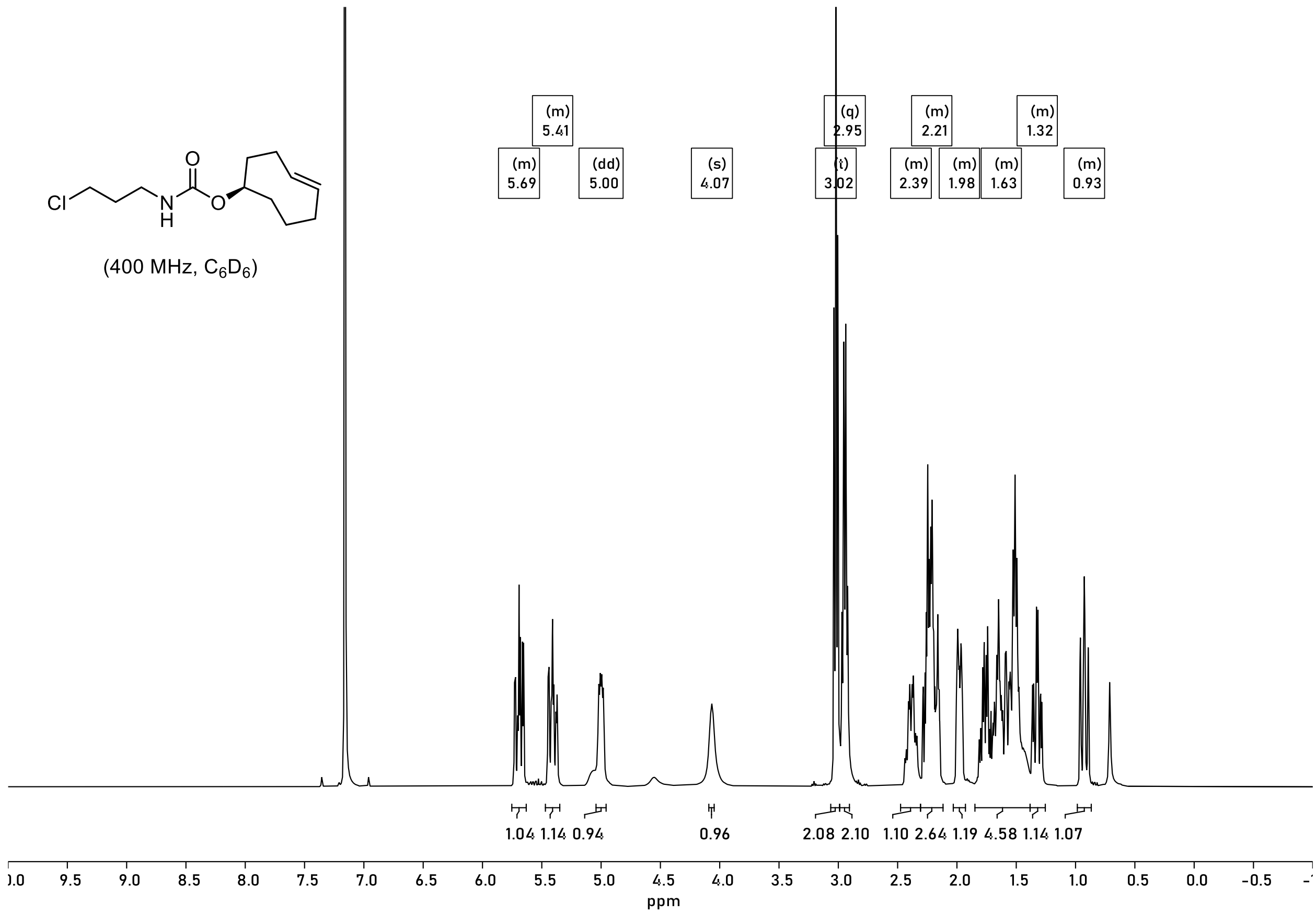

Cl
N
H
O
O
(400 MHz, C_6D_6)
(m) 5.69
(m) 5.41
(dd) 5.00
(s) 4.07
(t) 3.02
(q) 2.95
(m) 2.39
(m) 2.21
(m) 1.98
(m) 1.63
(m) 1.32
(m) 0.93
1.04 1.14 0.94 0.96 2.08 2.10 1.10 2.64 1.19 4.58 1.14 1.07
ppm

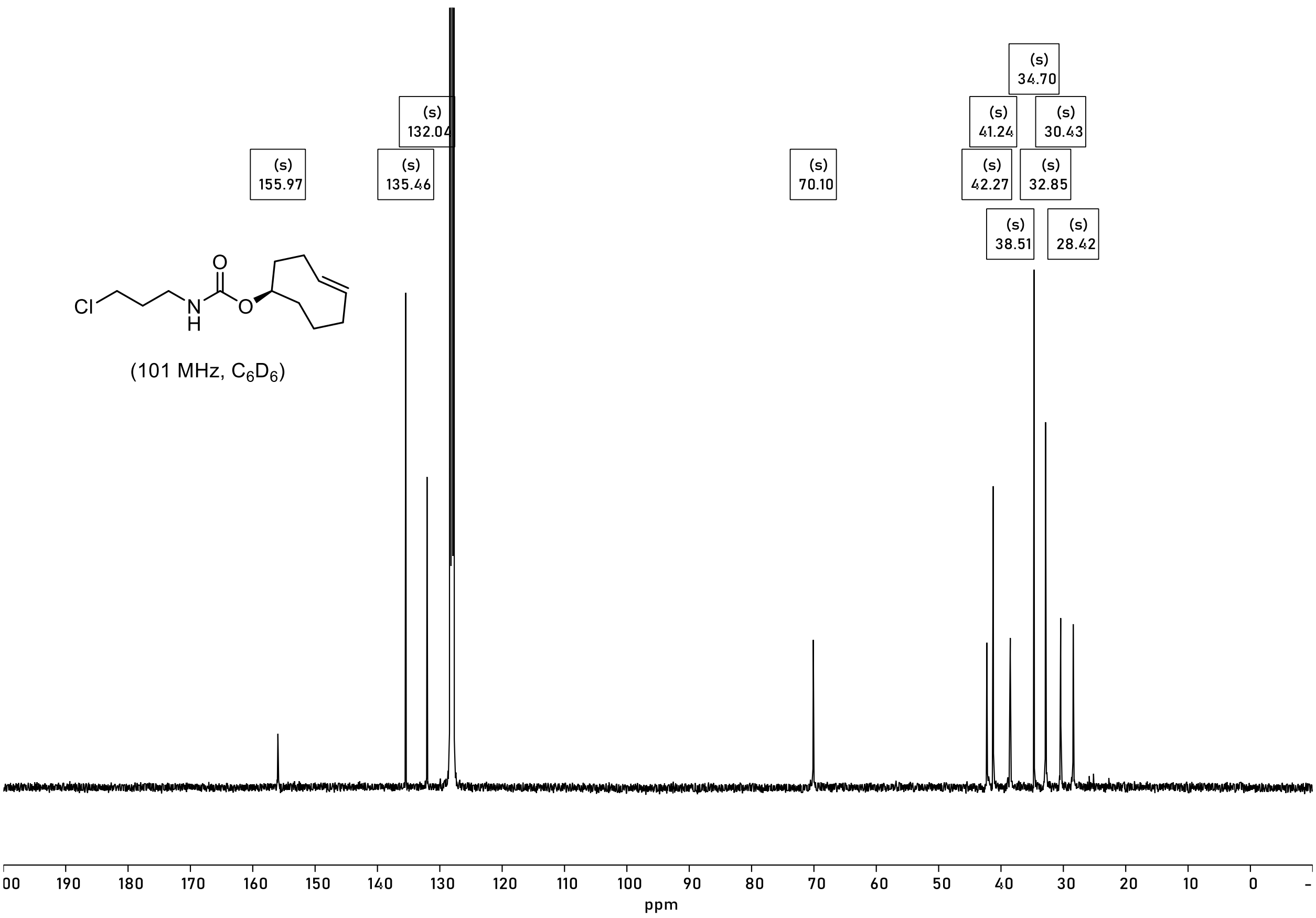
(s) 155.97
(s) 135.46
(s) 132.04
(s) 70.10
(s) 42.27
(s) 41.24
(s) 38.51
(s) 34.70
(s) 32.85
(s) 30.43
(s) 28.42
Cl
N
H
O
O
(101 MHz, C_6D_6)
00 190 180 170 160 150 140 130 120 110 100 90 80 70 60 50 40 30 20 10 0
ppm

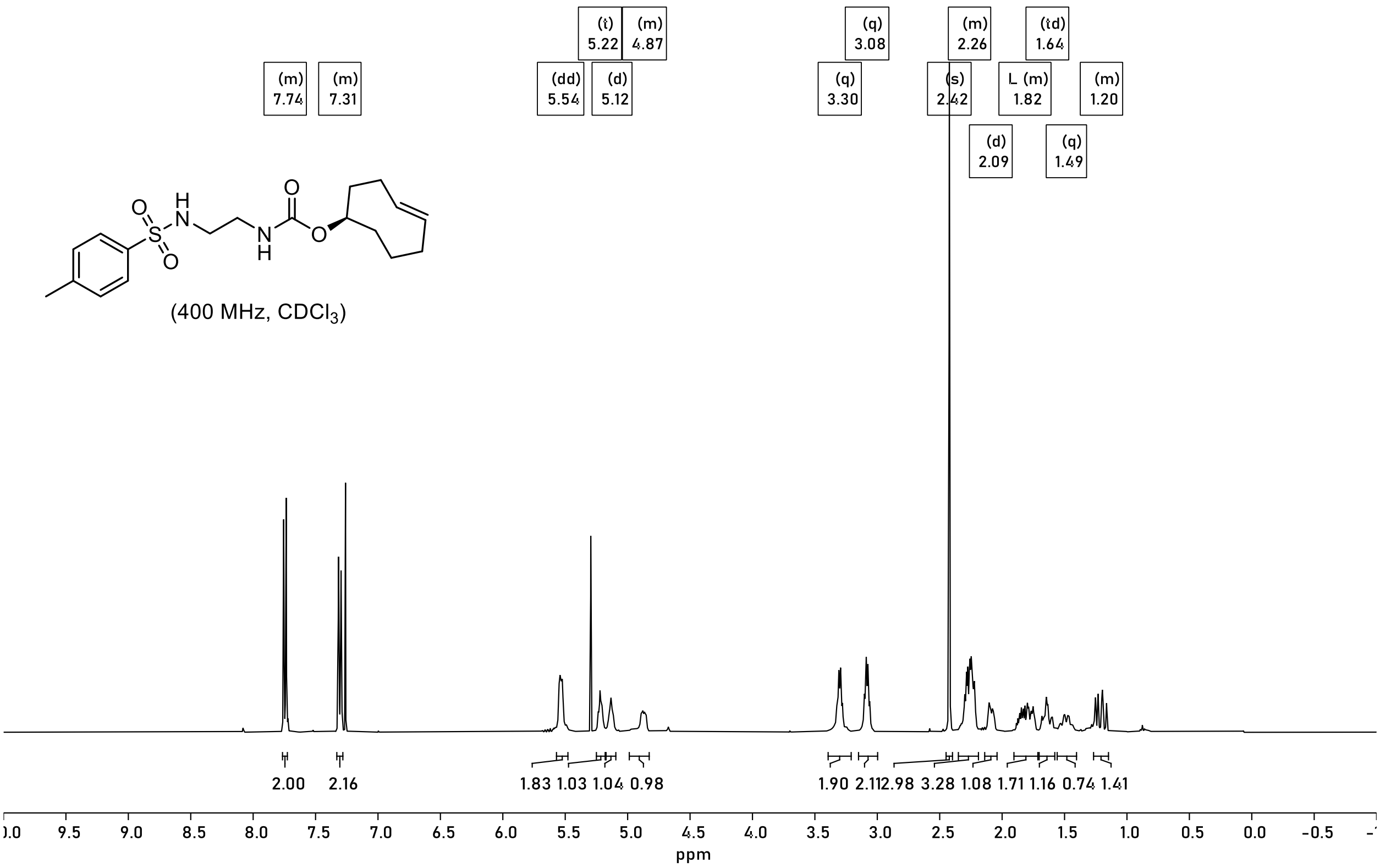
(m) 7.74
(m) 7.31
(dd) 5.54
(t) 5.22
(d) 5.12
(m) 4.87
(q) 3.30
(q) 3.08
(s) 2.42
(m) 2.26
(d) 2.09
(m) 1.82
(td) 1.64
(q) 1.49
(m) 1.20
O
S
O
H
N
N
H
O
O
(400 MHz, $CDCl_3$)
2.00 2.16
1.83 1.03 1.04 0.98
1.90 2.11 2.98 3.28 1.08 1.71 1.16 0.74 1.41
9.5 9.0 8.5 8.0 7.5 7.0 6.5 6.0 5.5 5.0 4.5 4.0 3.5 3.0 2.5 2.0 1.5 1.0 0.5 0.0 -0.5
ppm

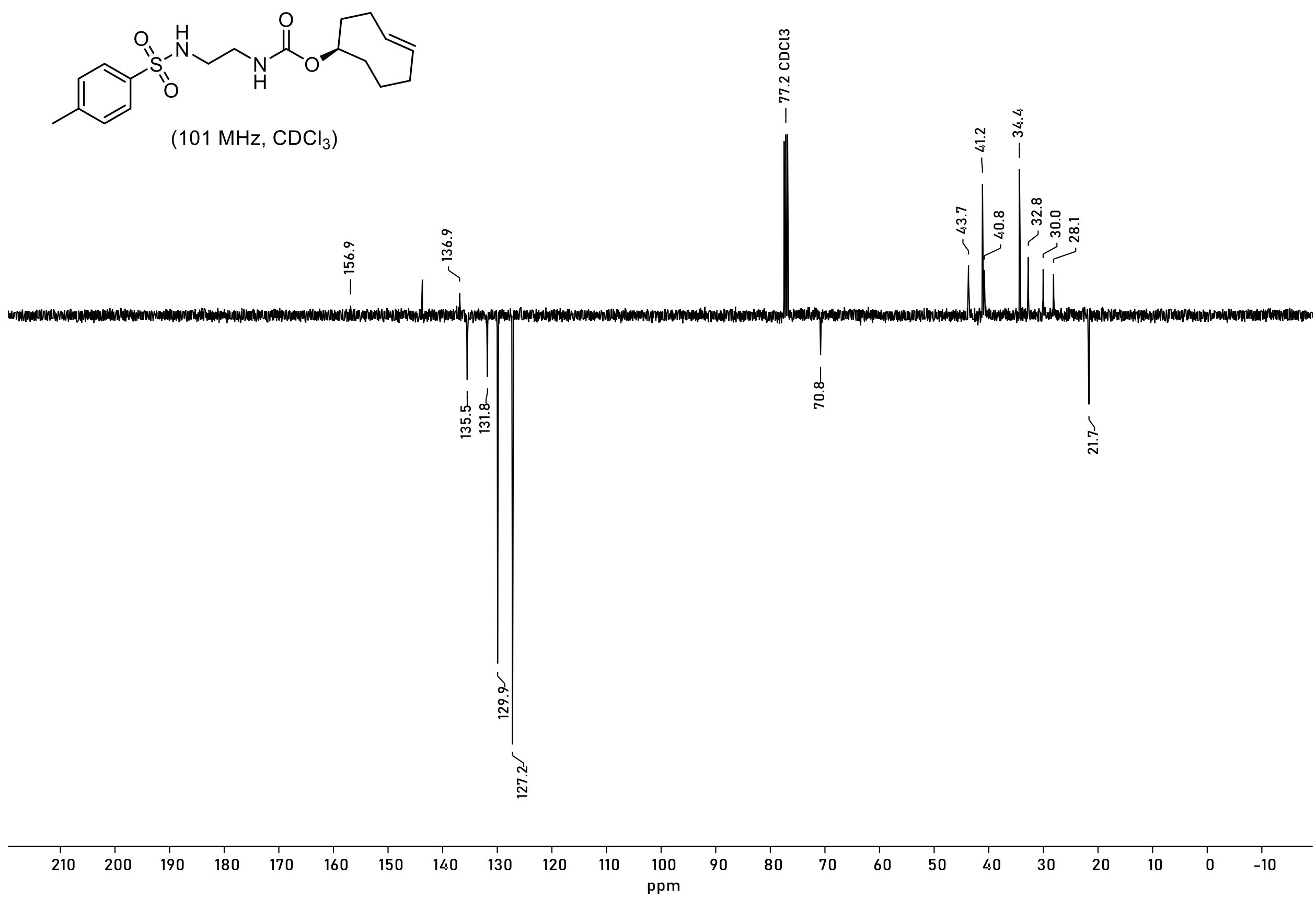
(101 MHz, CDCl3)
77.2 CDCl3
156.9
136.9
135.5
131.8
129.9
127.2
70.8
43.7
41.2
40.8
34.4
32.8
30.0
28.1
21.7
210 200 190 180 170 160 150 140 130 120 110 100 90 80 70 60 50 40 30 20 10 0 -10
ppm

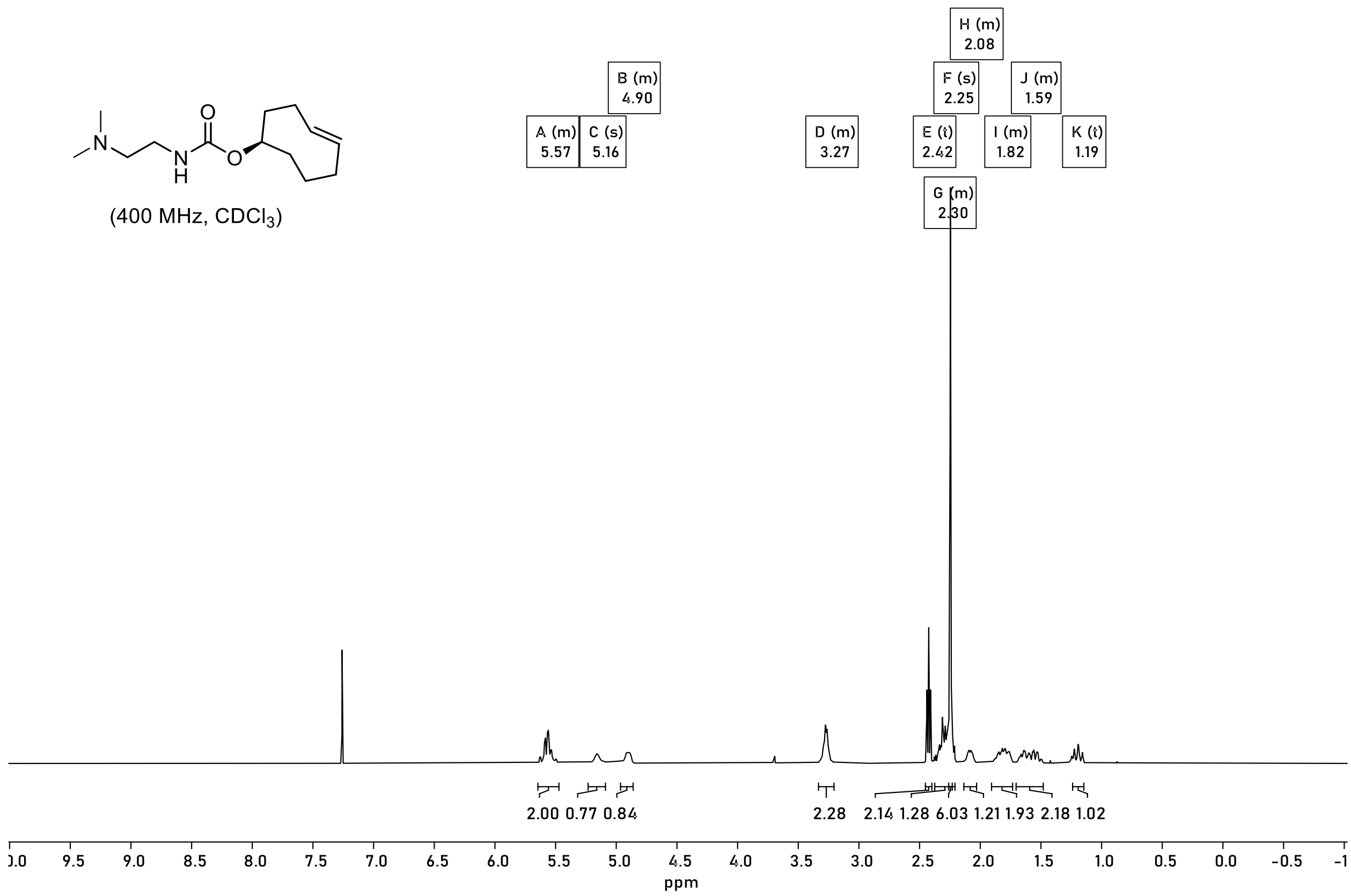
(400 MHz, CDCl3)
A (m) 5.57
B (m) 4.90
C (s) 5.16
D (m) 3.27
E (t) 2.42
F (s) 2.25
G (m) 2.30
H (m) 2.08
I (m) 1.82
J (m) 1.59
K (t) 1.19
2.00 0.77 0.84 2.28 2.14 1.28 6.03 1.21 1.93 2.18 1.02
9.5 9.0 8.5 8.0 7.5 7.0 6.5 6.0 5.5 5.0 4.5 4.0 3.5 3.0 2.5 2.0 1.5 1.0 0.5 0.0 -0.5
ppm

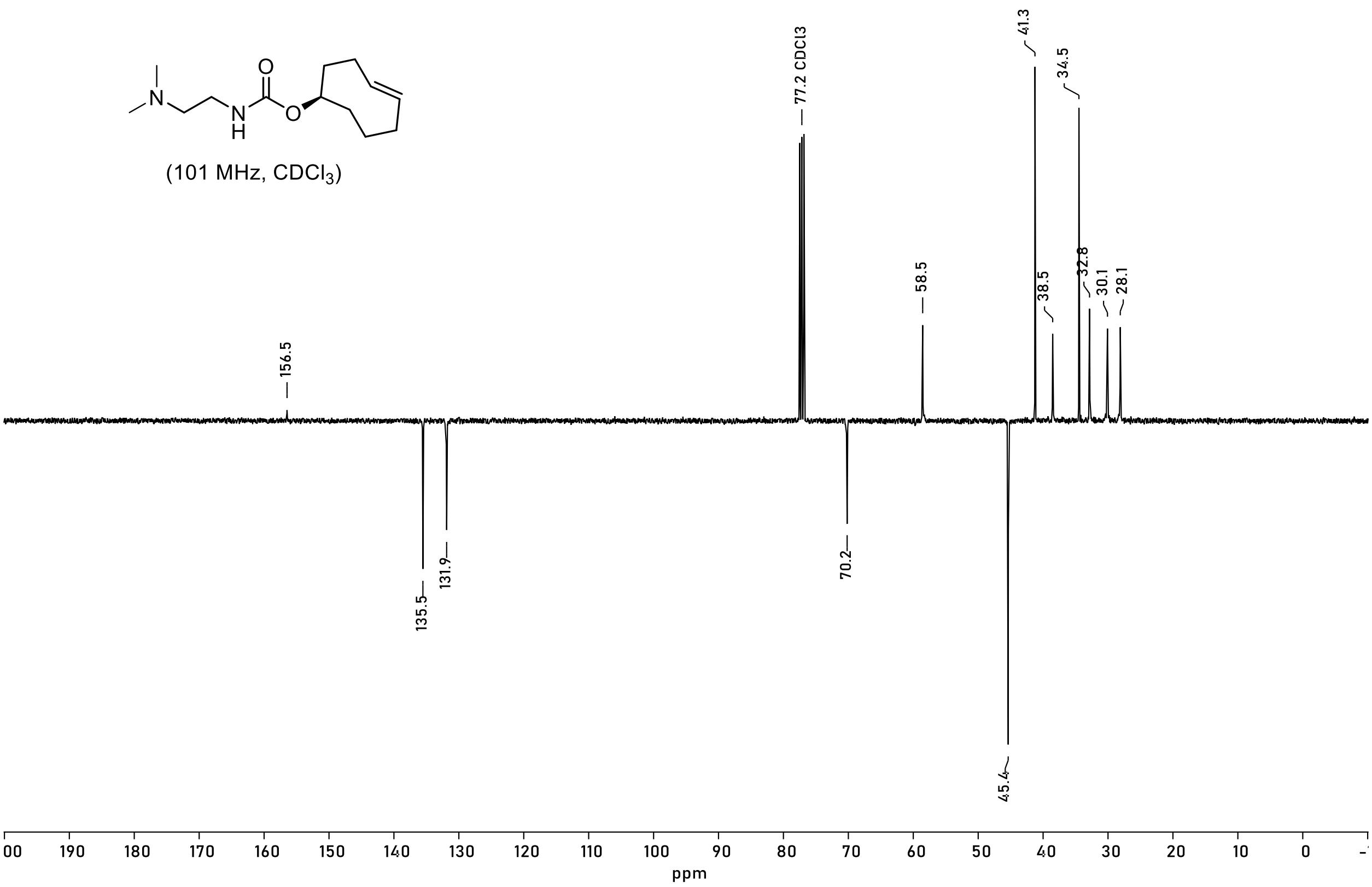
(101 MHz, CDCl3)
77.2 CDCl3
41.3
34.5
58.5
38.5
32.8
30.1
28.1
156.5
135.5
131.9
70.2
45.4
ppm

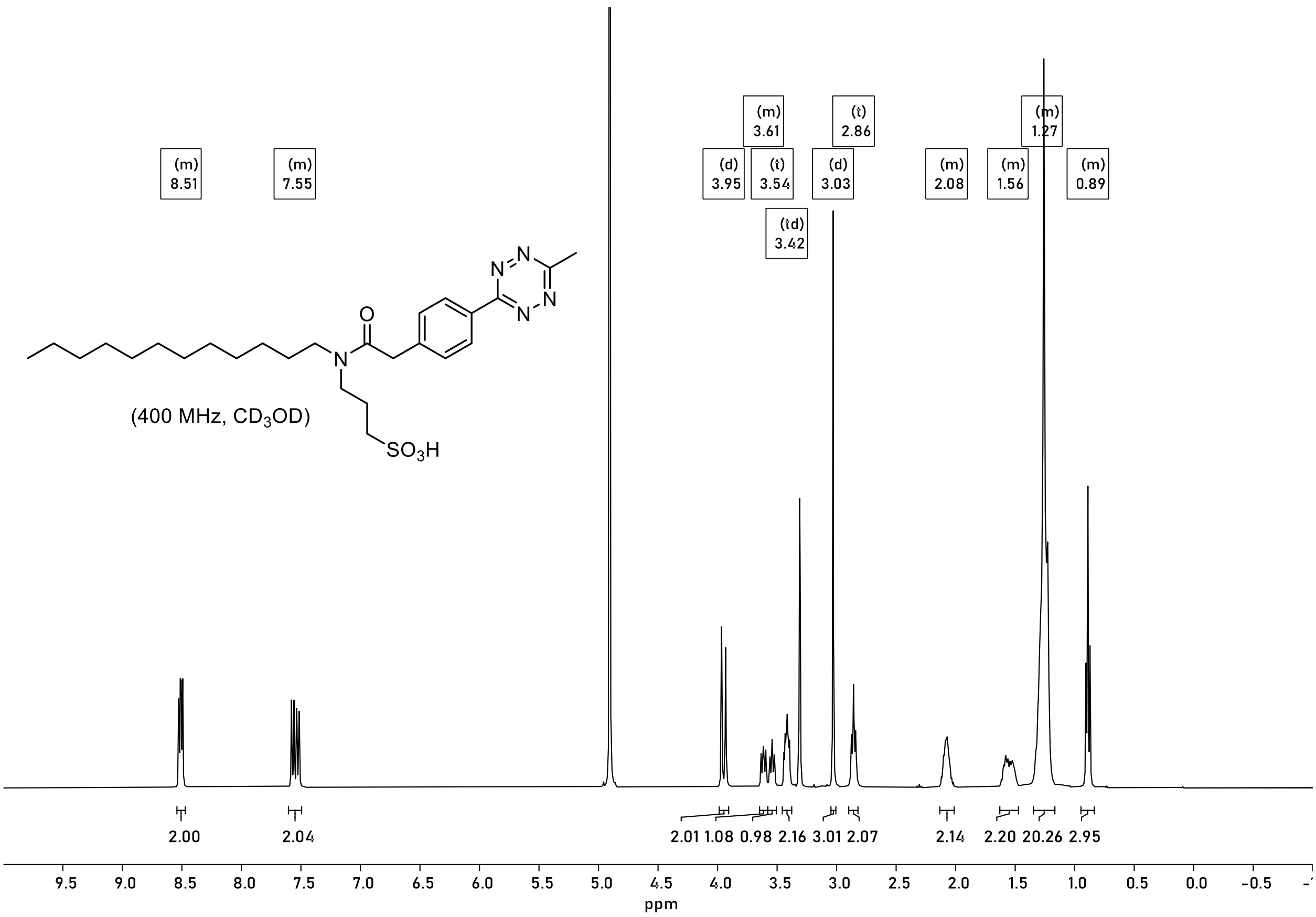
(400 MHz, CD3OD)
SO3H
(m) 8.51
(m) 7.55
(d) 3.95
(m) 3.61
(t) 3.54
(td) 3.42
(d) 3.03
(t) 2.86
(m) 2.08
(m) 1.56
(m) 1.27
(m) 0.89
2.00
2.04
2.01 1.08 0.98 2.16 3.01 2.07
2.14
2.20 20.26 2.95
ppm

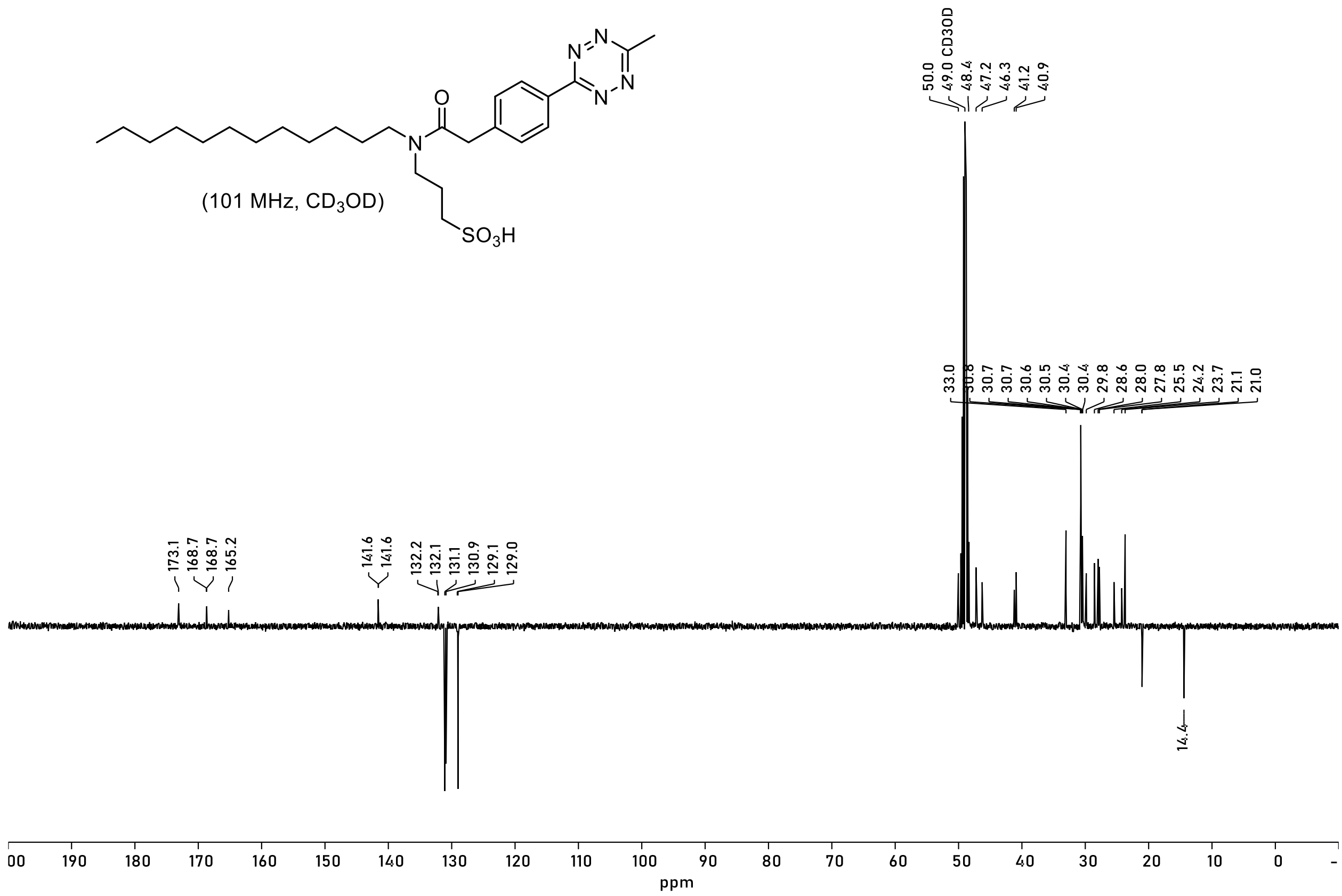
(101 MHz, CD3OD)
SO3H
ppm

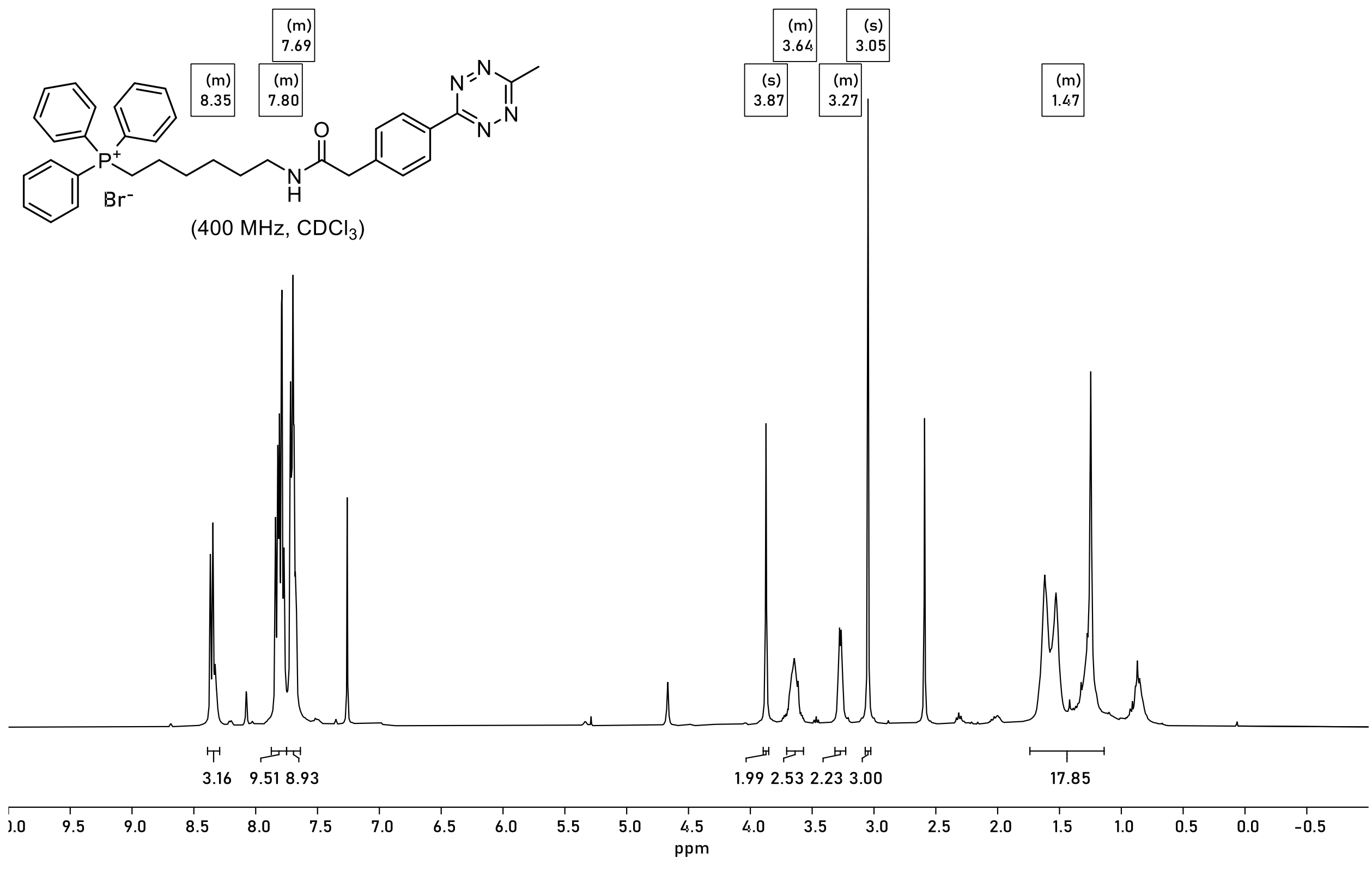
Br⁻
(400 MHz, CDCl3)
ppm

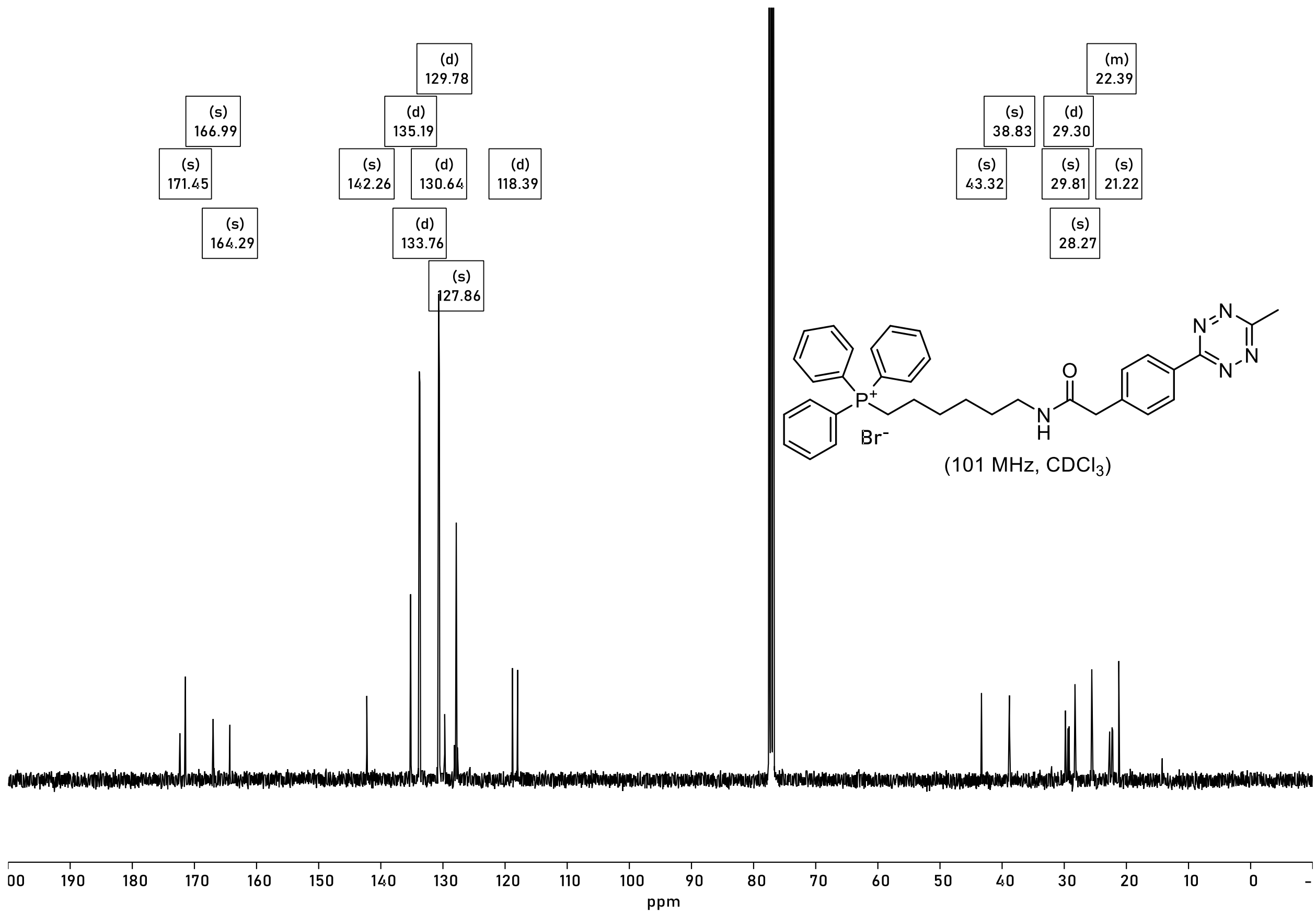
(d) 129.78
(s) 166.99
(d) 135.19
(s) 171.45
(s) 142.26
(d) 130.64
(d) 118.39
(s) 164.29
(d) 133.76
(s) 127.86
(m) 22.39
(s) 38.83
(d) 29.30
(s) 43.32
(s) 29.81
(s) 21.22
(s) 28.27
Br⁻
(101 MHz, CDCl3)
ppm

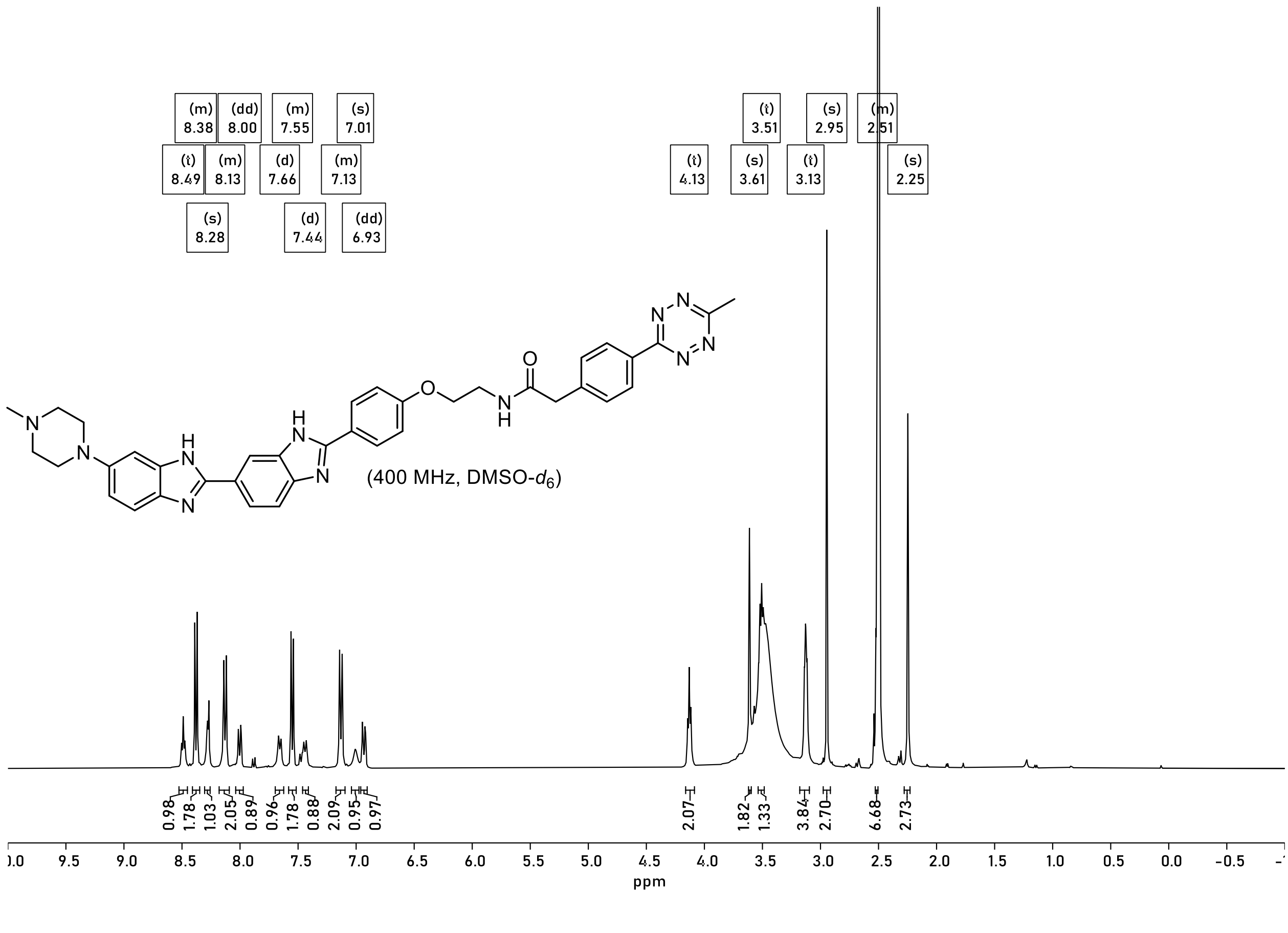
(m) 8.38
(dd) 8.00
(m) 7.55
(s) 7.01
(t) 8.49
(m) 8.13
(d) 7.66
(m) 7.13
(s) 8.28
(d) 7.44
(dd) 6.93
(t) 3.51
(s) 2.95
(m) 2.51
(t) 4.13
(s) 3.61
(t) 3.13
(s) 2.25
(400 MHz, DMSO-d6)
ppm

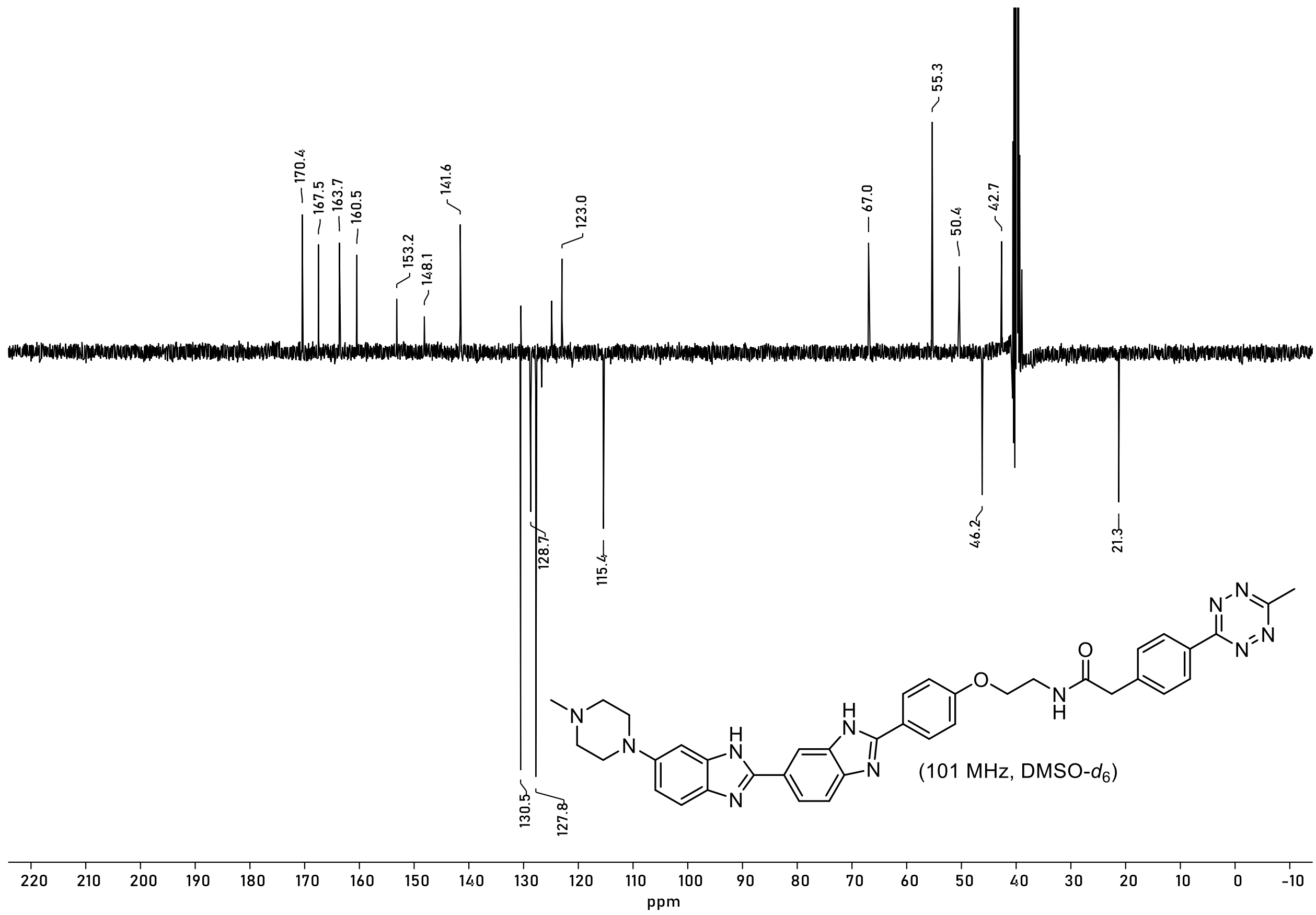
170.4
167.5
163.7
160.5
153.2
148.1
141.6
123.0
67.0
55.3
50.4
42.7
128.7
115.4
46.2
21.3
130.5
127.8
(101 MHz, DMSO-d_6)
220 210 200 190 180 170 160 150 140 130 120 110 100 90 80 70 60 50 40 30 20 10 0 -10
ppm

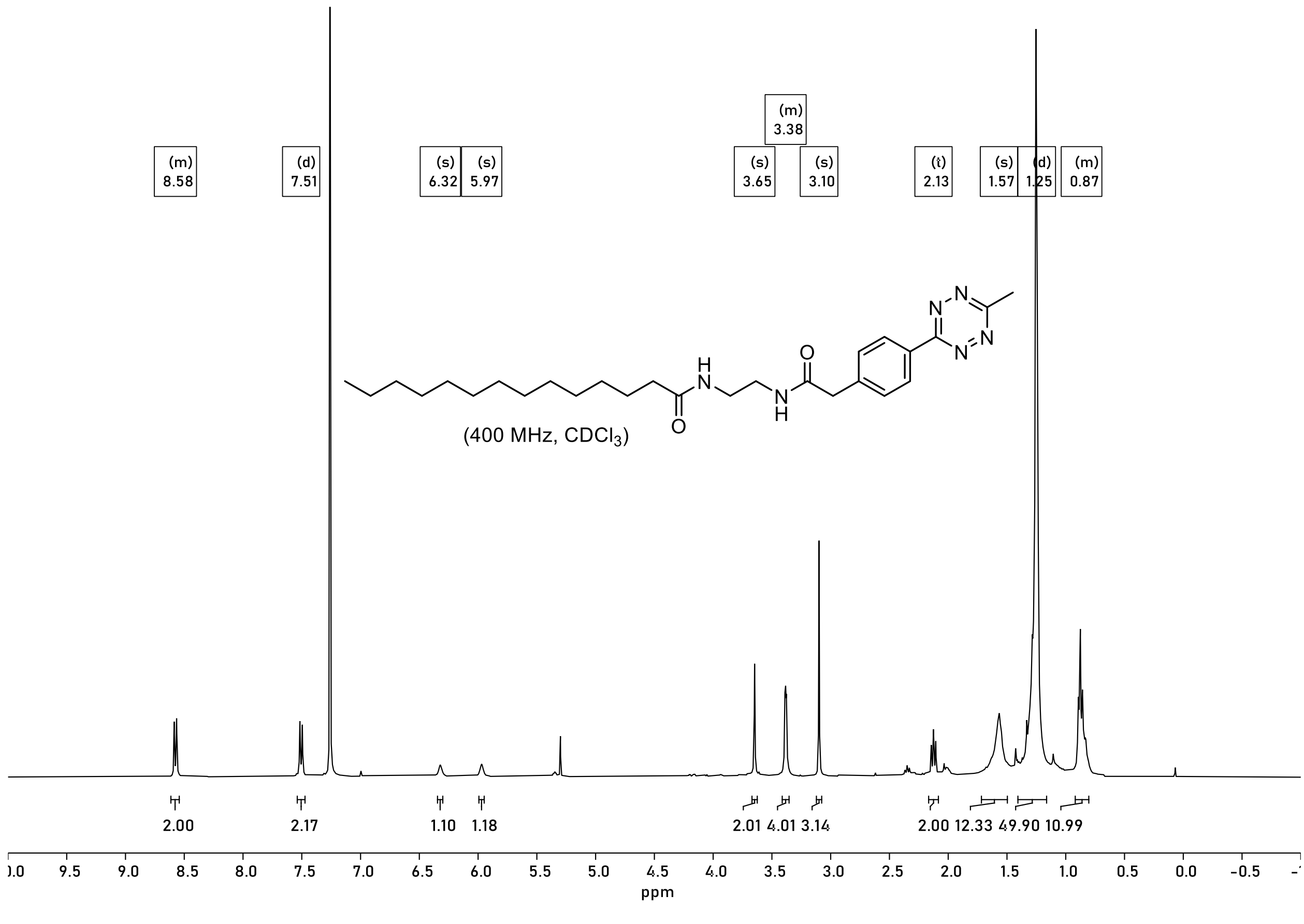
(m) 8.58
(d) 7.51
(s) 6.32
(s) 5.97
(m) 3.38
(s) 3.65
(s) 3.10
(t) 2.13
(s) 1.57
(d) 1.25
(m) 0.87
(400 MHz, $CDCl_3$)
2.00 2.17 1.10 1.18 2.01 4.01 3.14 2.00 12.33 49.90 10.99
9.5 9.0 8.5 8.0 7.5 7.0 6.5 6.0 5.5 5.0 4.5 4.0 3.5 3.0 2.5 2.0 1.5 1.0 0.5 0.0 -0.5
ppm

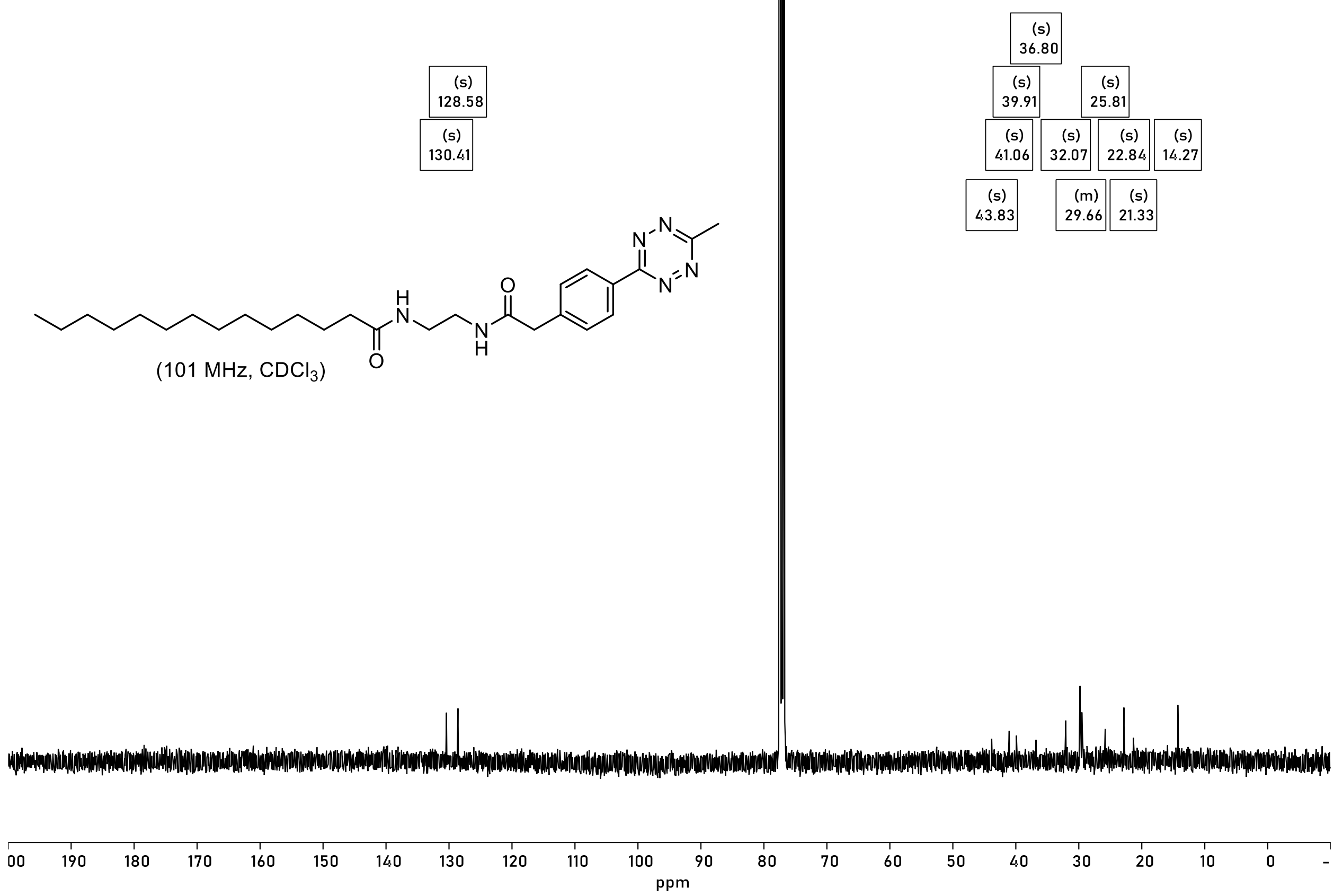
(s) 36.80
(s) 128.58
(s) 130.41
(s) 39.91
(s) 25.81
(s) 41.06
(s) 32.07
(s) 22.84
(s) 14.27
(s) 43.83
(m) 29.66
(s) 21.33
(101 MHz, CDCl3)
ppm

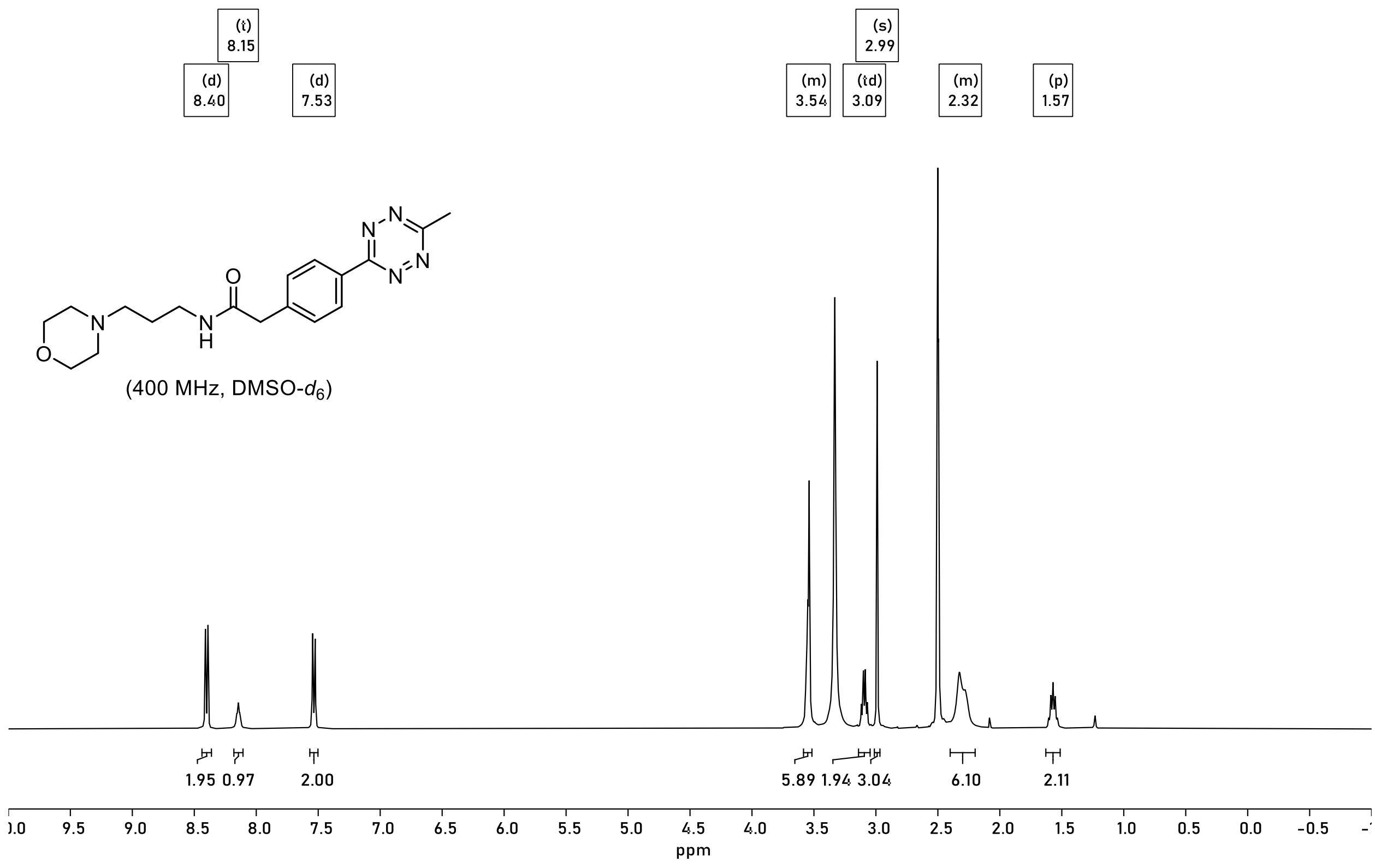
(t) 8.15
(d) 8.40
(d) 7.53
(s) 2.99
(m) 3.54
(td) 3.09
(m) 2.32
(p) 1.57
(400 MHz, DMSO-d6)
1.95
0.97
2.00
5.89
1.94
3.04
6.10
2.11
ppm

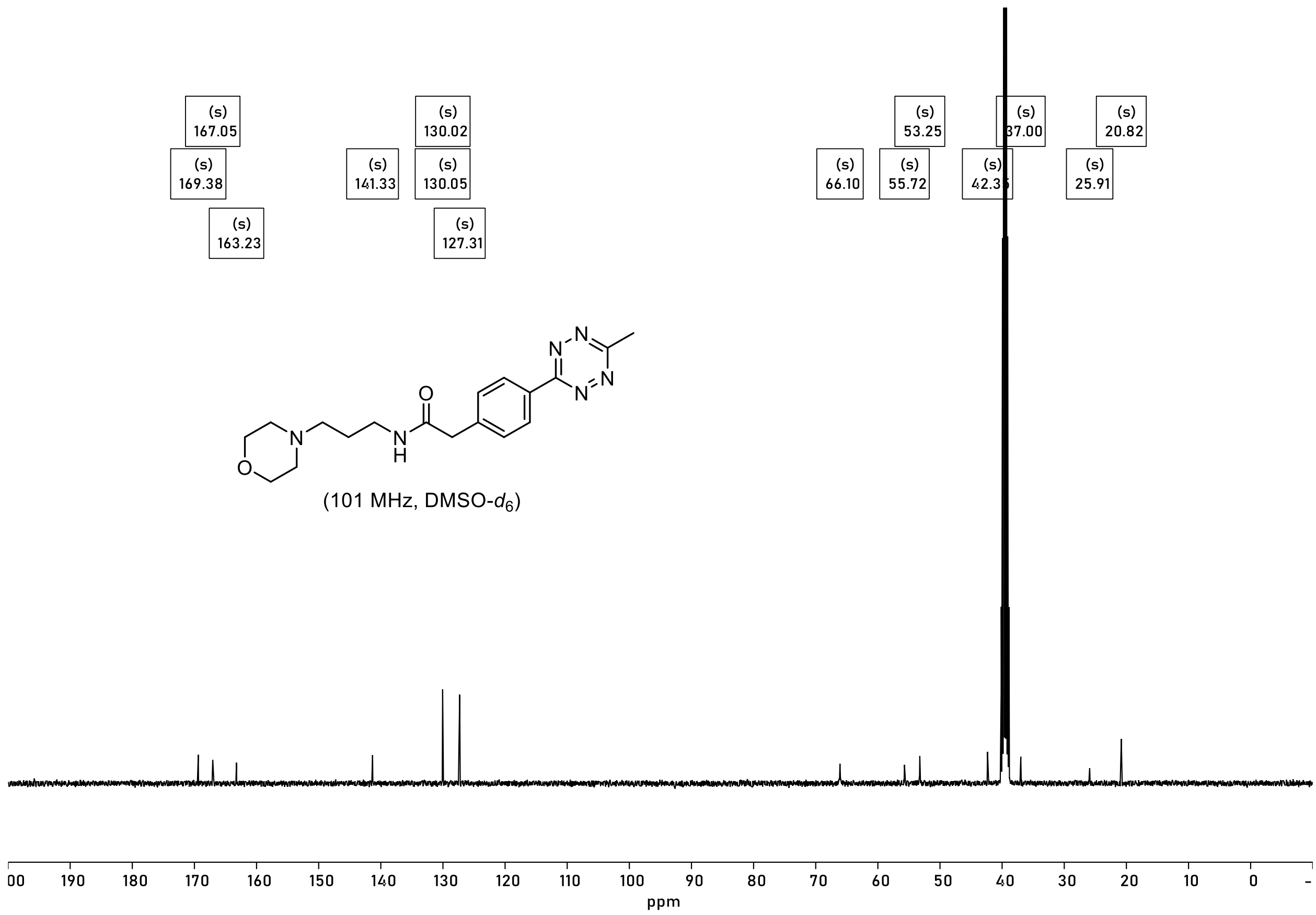
(s) 167.05
(s) 169.38
(s) 163.23
(s) 141.33
(s) 130.02
(s) 130.05
(s) 127.31
(s) 66.10
(s) 55.72
(s) 53.25
(s) 42.35
(s) 37.00
(s) 25.91
(s) 20.82
(101 MHz, DMSO-d_6)
ppm

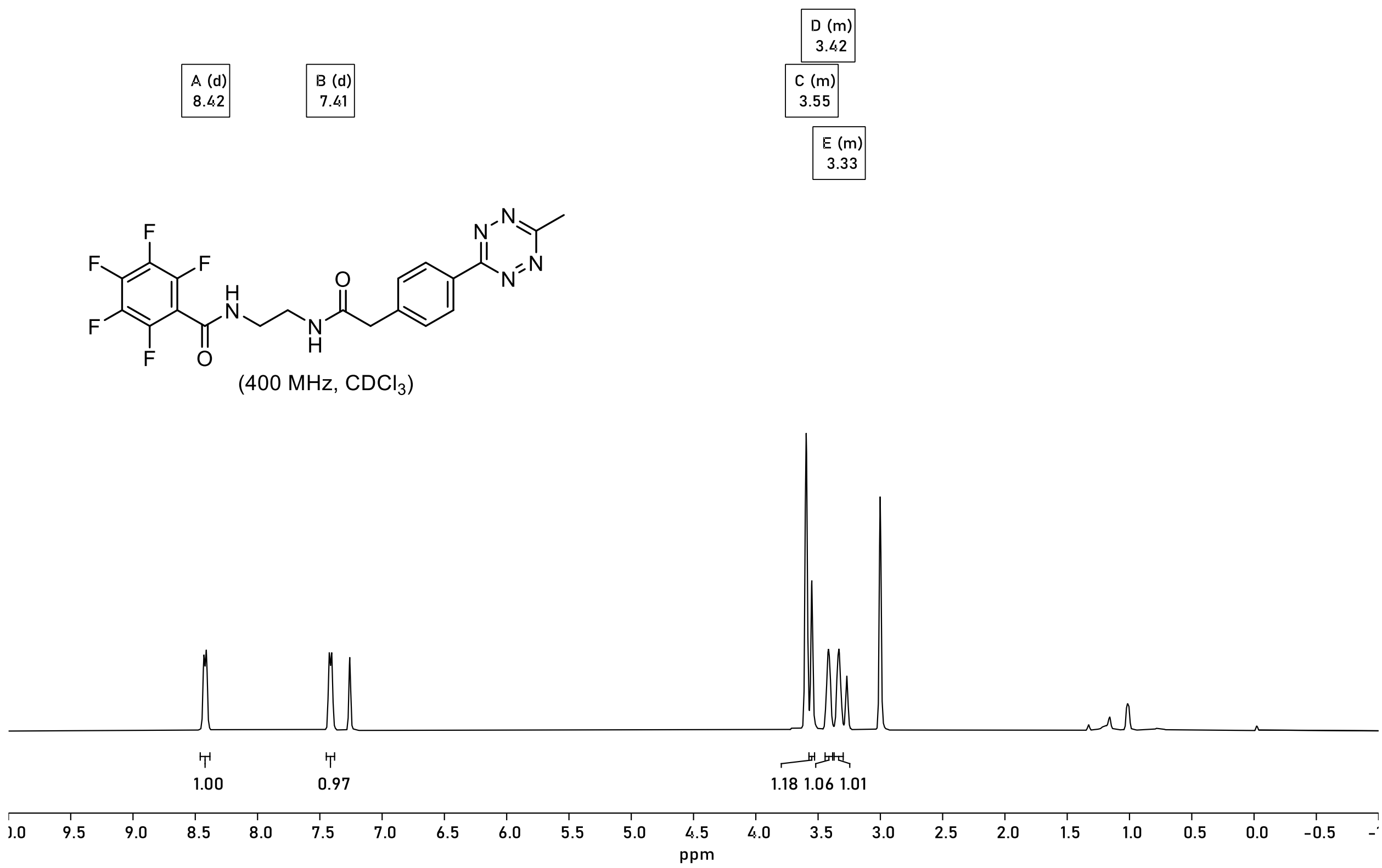
A (d) 8.42
B (d) 7.41
C (m) 3.55
D (m) 3.42
E (m) 3.33
(400 MHz, $CDCl_3$)
1.00
0.97
1.18 1.06 1.01
ppm

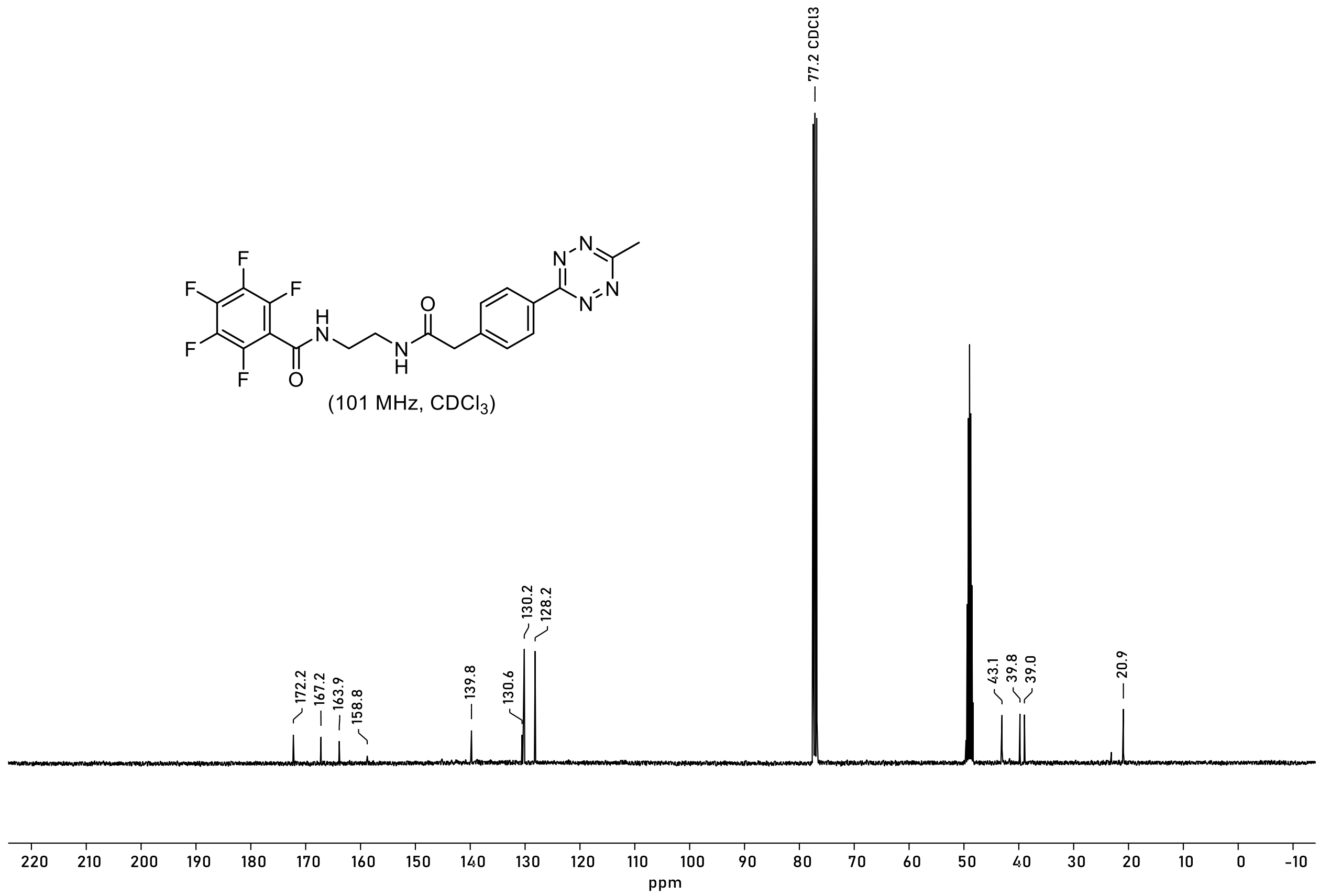

F
F
F
F
F
H
N
N
H
O
O
N
N
N
N
(101 MHz, CDCl3)
77.2 CDCl3
172.2
167.2
163.9
158.8
139.8
130.6
130.2
128.2
43.1
39.8
39.0
20.9
220 210 200 190 180 170 160 150 140 130 120 110 100 90 80 70 60 50 40 30 20 10 0 -10
ppm

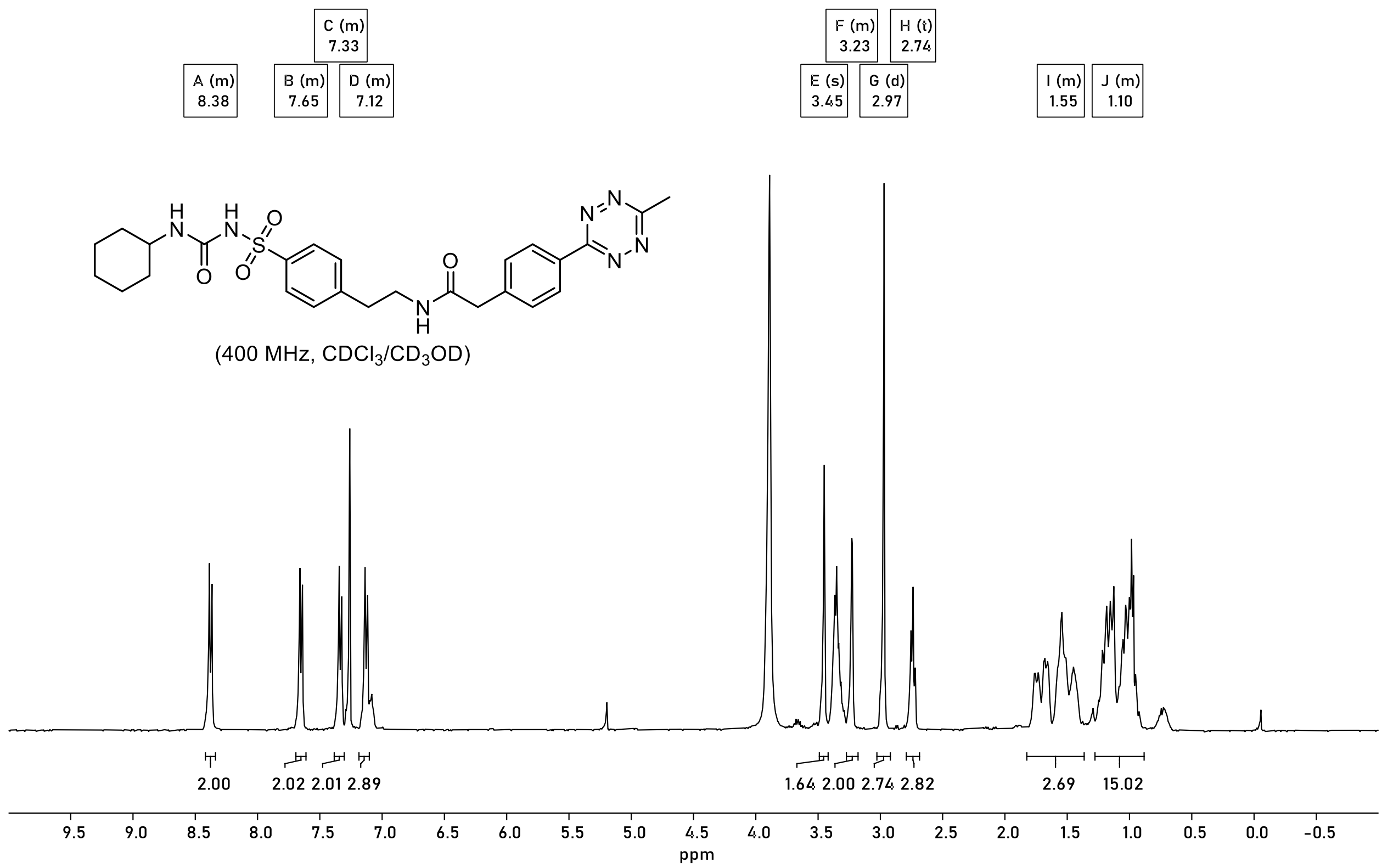

A (m) 8.38
B (m) 7.65
C (m) 7.33
D (m) 7.12
E (s) 3.45
F (m) 3.23
G (d) 2.97
H (t) 2.74
I (m) 1.55
J (m) 1.10
H
N
H
N
O
S
O
O
N
H
O
N
N
N
N
(400 MHz, CDCl3/CD3OD)
2.00
2.02 2.01 2.89
1.64 2.00 2.74 2.82
2.69
15.02
9.5 9.0 8.5 8.0 7.5 7.0 6.5 6.0 5.5 5.0 4.5 4.0 3.5 3.0 2.5 2.0 1.5 1.0 0.5 0.0 -0.5
ppm

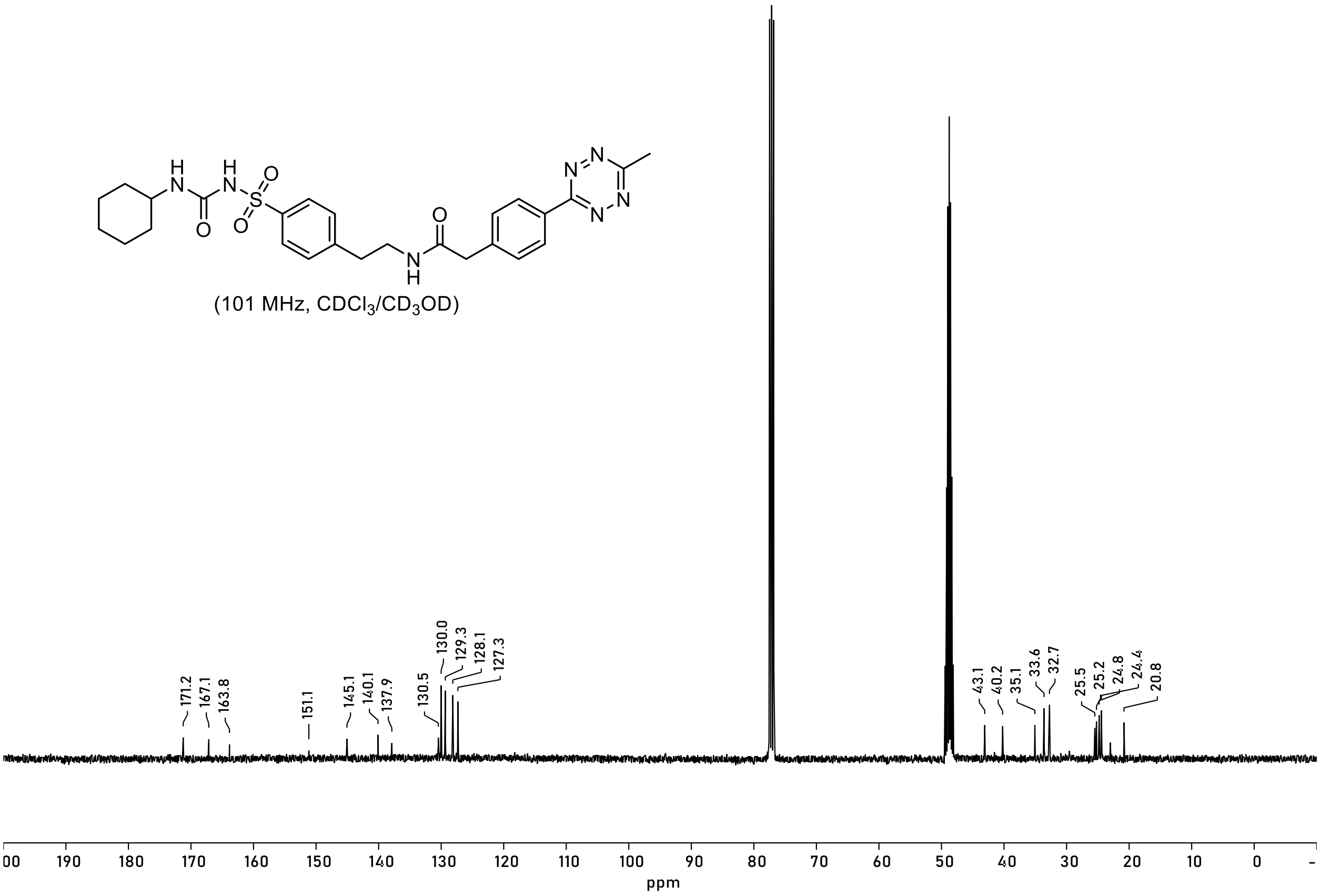
(101 MHz, CDCl3/CD3OD)
171.2
167.1
163.8
151.1
145.1
140.1
137.9
130.5
130.0
129.3
128.1
127.3
43.1
40.2
35.1
33.6
32.7
25.5
25.2
24.8
24.4
20.8
ppm

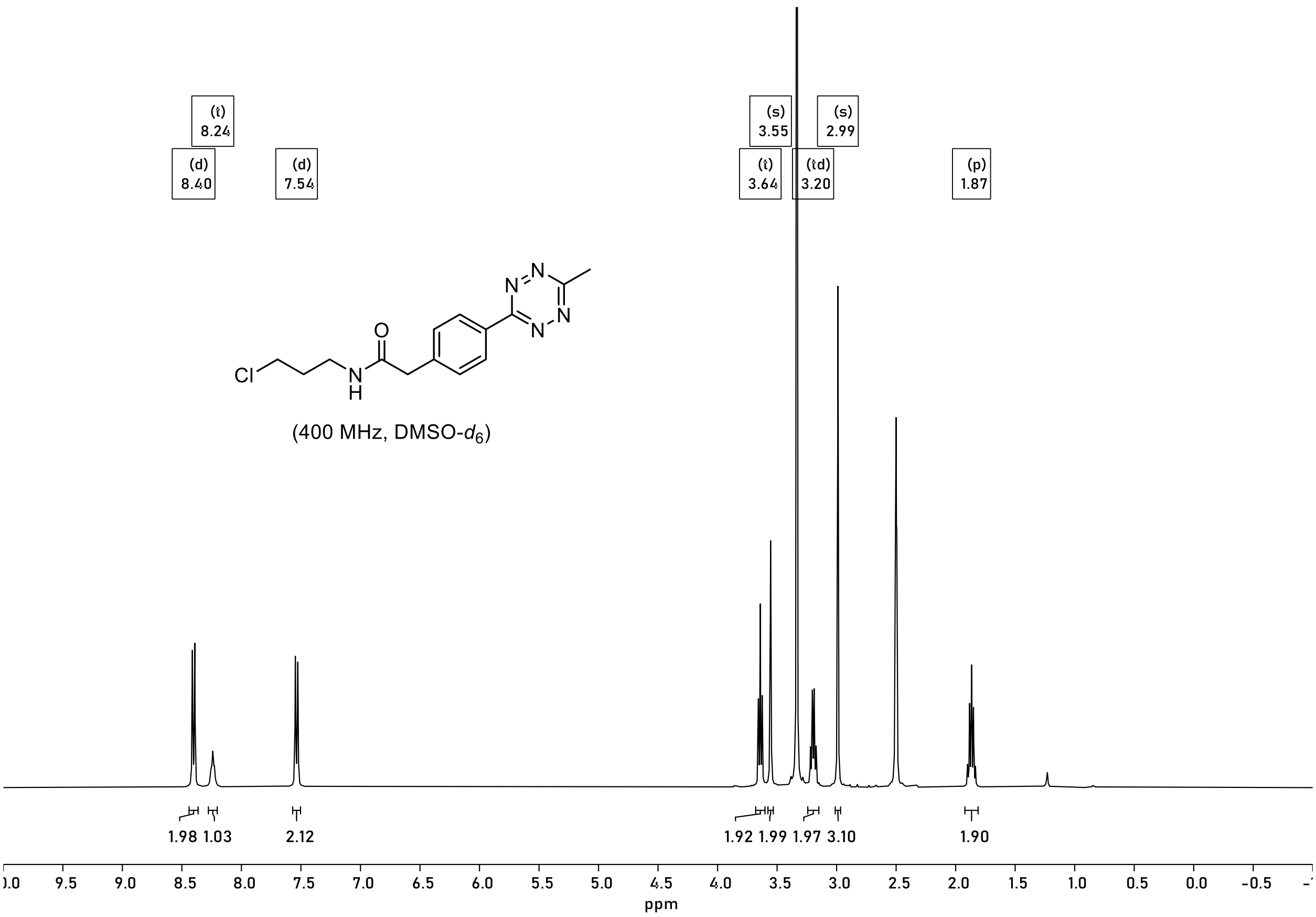
(t) 8.24
(d) 8.40
(d) 7.54
(s) 3.55
(s) 2.99
(t) 3.64
(td) 3.20
(p) 1.87
Cl
(400 MHz, DMSO-d6)
1.98 1.03
2.12
1.92 1.99 1.97 3.10
1.90
ppm

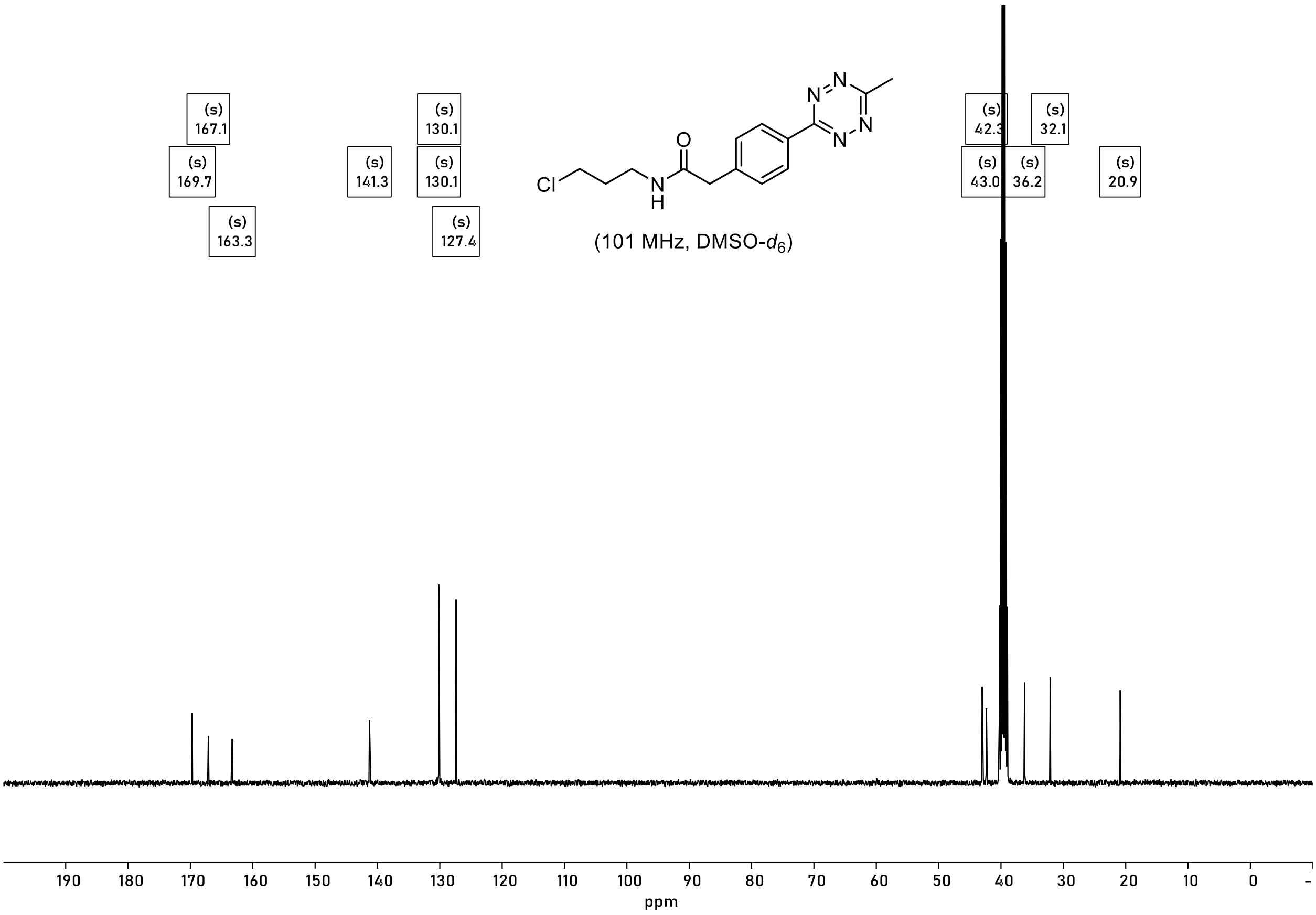
(101 MHz, DMSO-d_6)
Cl
N
H
O
N
N
N
N
(s) 167.1
(s) 169.7
(s) 163.3
(s) 141.3
(s) 130.1
(s) 130.1
(s) 127.4
(s) 42.3
(s) 43.0
(s) 36.2
(s) 32.1
(s) 20.9
190 180 170 160 150 140 130 120 110 100 90 80 70 60 50 40 30 20 10 0
ppm

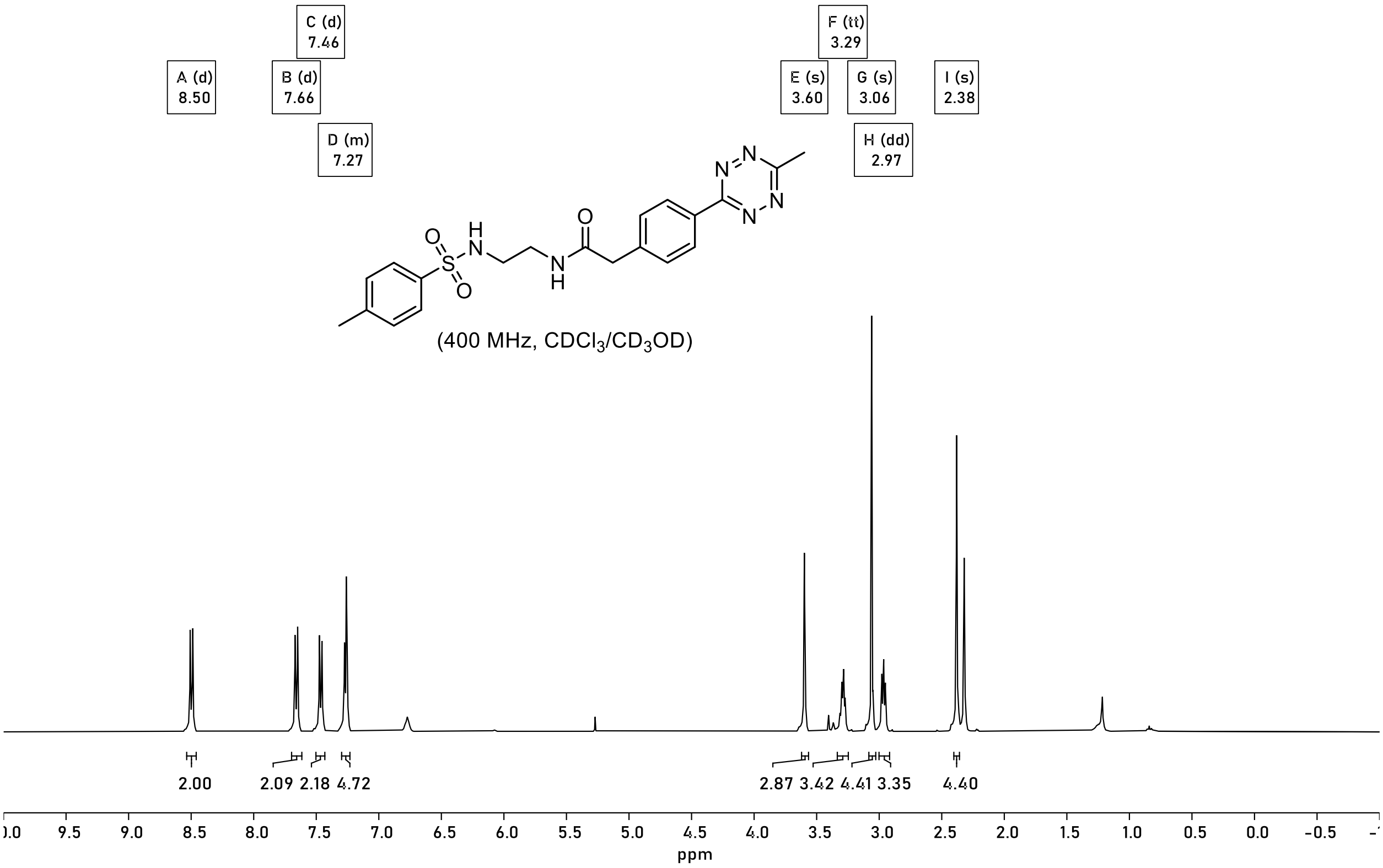
(400 MHz, $CDCl_3/CD_3OD$)
A (d) 8.50
B (d) 7.66
C (d) 7.46
D (m) 7.27
E (s) 3.60
F (tt) 3.29
G (s) 3.06
H (dd) 2.97
I (s) 2.38
2.00
2.09 2.18 4.72
2.87 3.42 4.41 3.35
4.40
9.5 9.0 8.5 8.0 7.5 7.0 6.5 6.0 5.5 5.0 4.5 4.0 3.5 3.0 2.5 2.0 1.5 1.0 0.5 0.0 -0.5
ppm

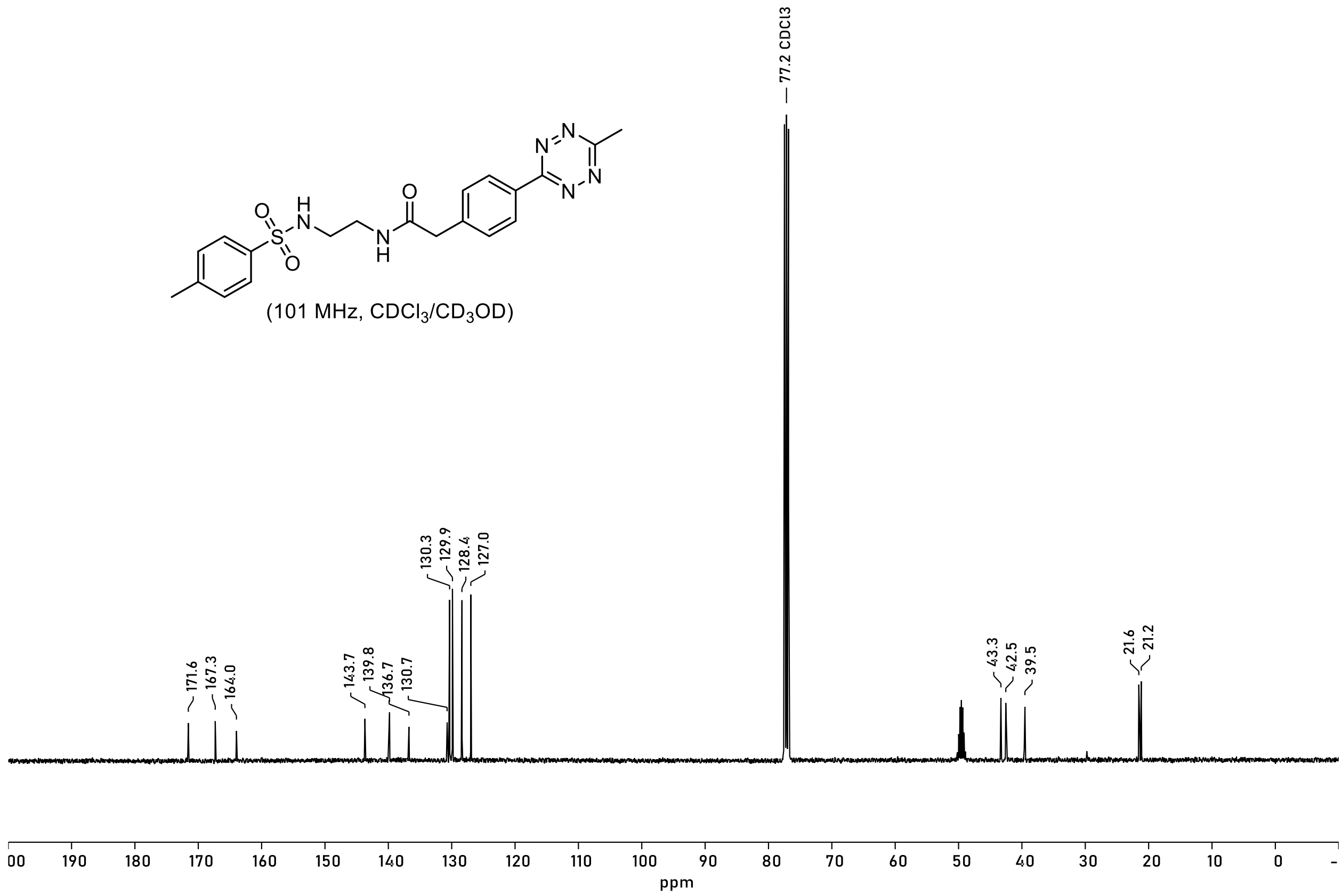
(101 MHz, CDCl3/CD3OD)
77.2 CDCl3
171.6
167.3
164.0
143.7
139.8
136.7
130.7
130.3
129.9
128.4
127.0
43.3
42.5
39.5
21.6
21.2
ppm

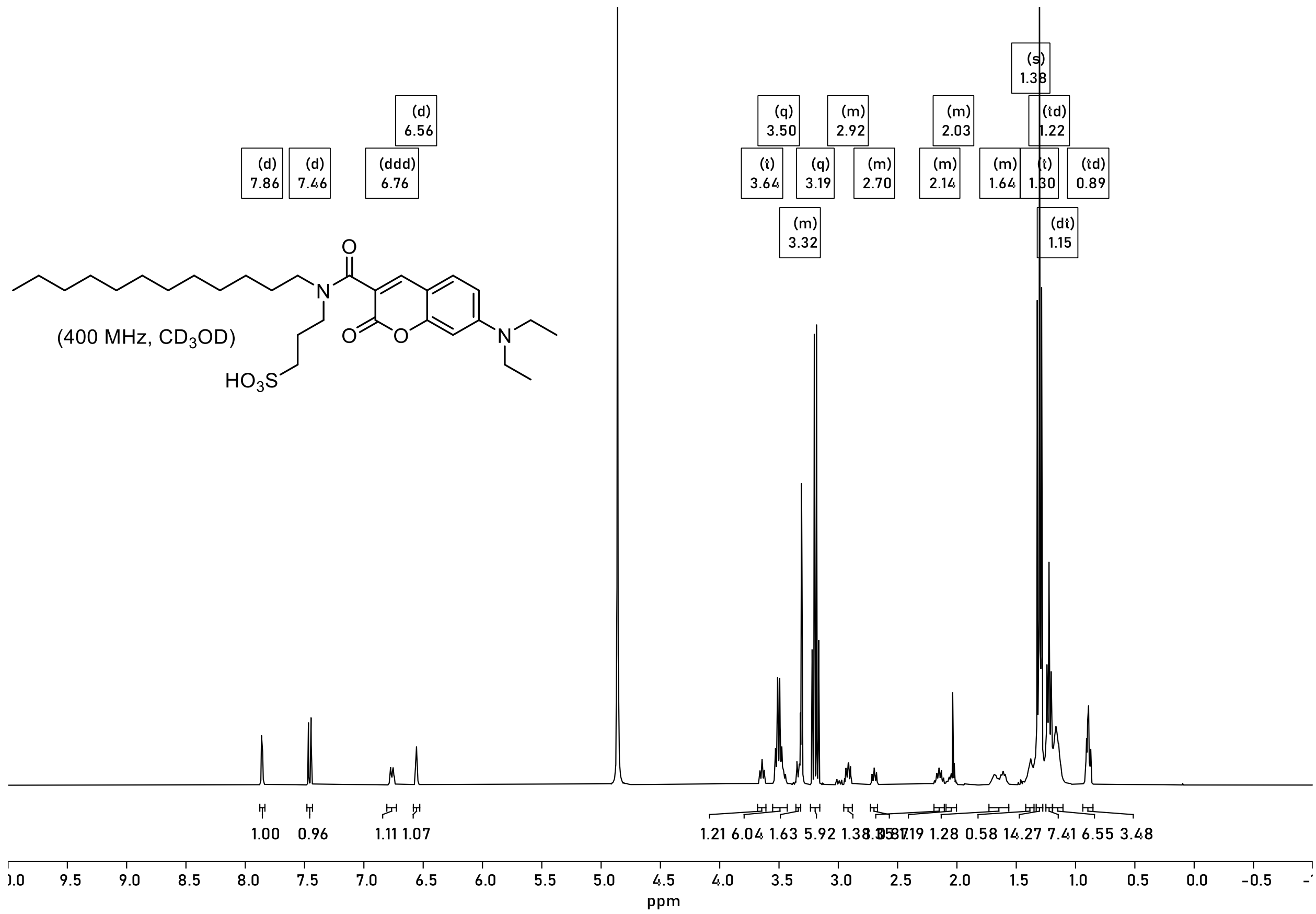
(400 MHz, CD3OD)
(d) 7.86
(d) 7.46
(ddd) 6.76
(d) 6.56
(t) 3.64
(q) 3.50
(m) 3.32
(q) 3.19
(m) 2.92
(m) 2.70
(m) 2.14
(m) 2.03
(m) 1.64
(s) 1.38
(t) 1.30
(td) 1.22
(dt) 1.15
(td) 0.89
1.00
0.96
1.11
1.07
1.21
6.04
1.63
5.92
1.28
0.58
14.27
7.41
6.55
3.48
ppm

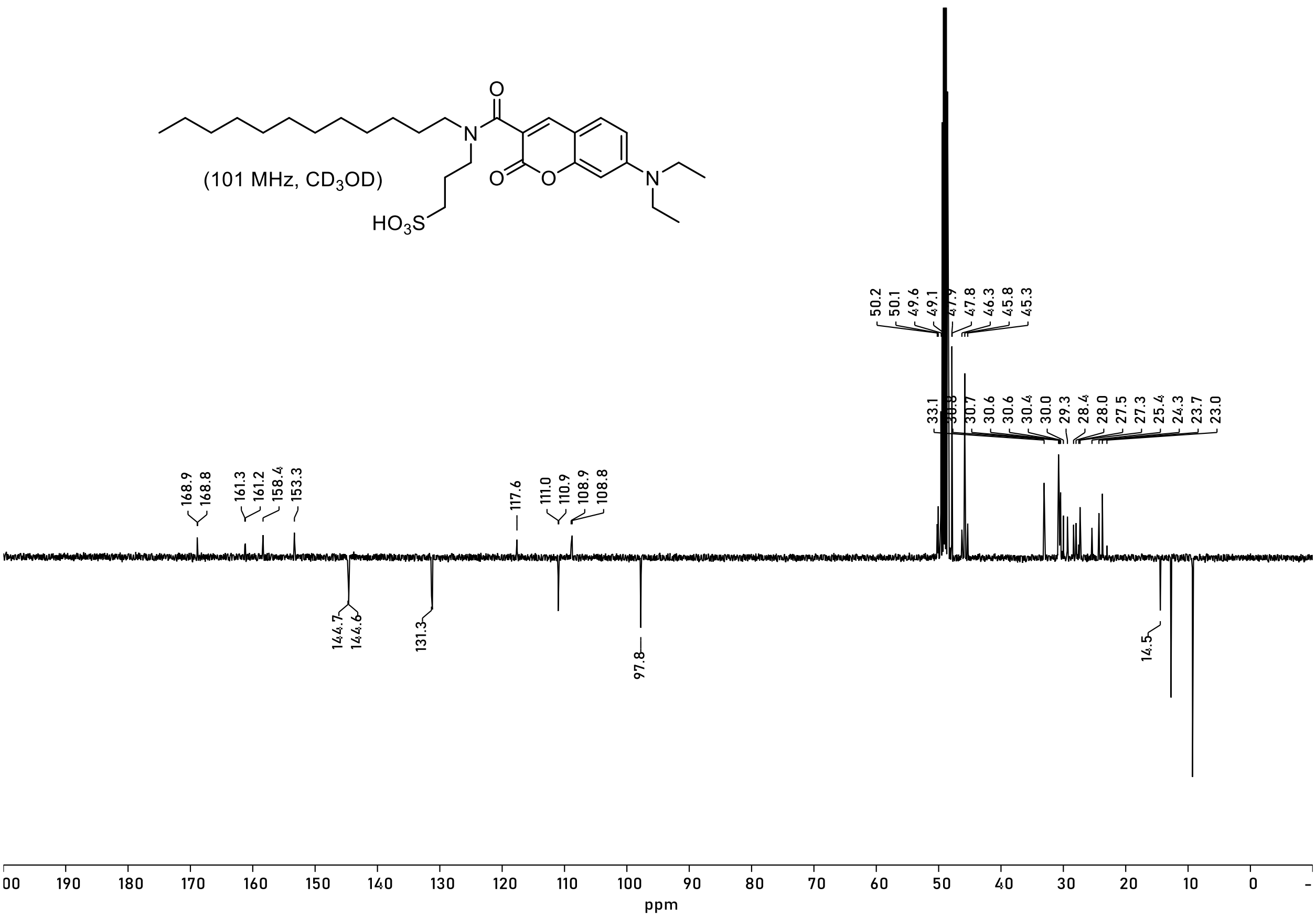
(101 MHz, CD_3OD)
HO_3S
168.9
168.8
161.3
161.2
158.4
153.3
117.6
111.0
110.9
108.9
108.8
144.7
144.6
131.3
97.8
50.2
50.1
49.6
49.1
47.9
47.8
46.3
45.8
45.3
33.1
30.7
30.6
30.6
30.4
30.0
29.3
28.4
28.0
27.5
27.3
25.4
24.3
23.7
23.0
14.5
ppm

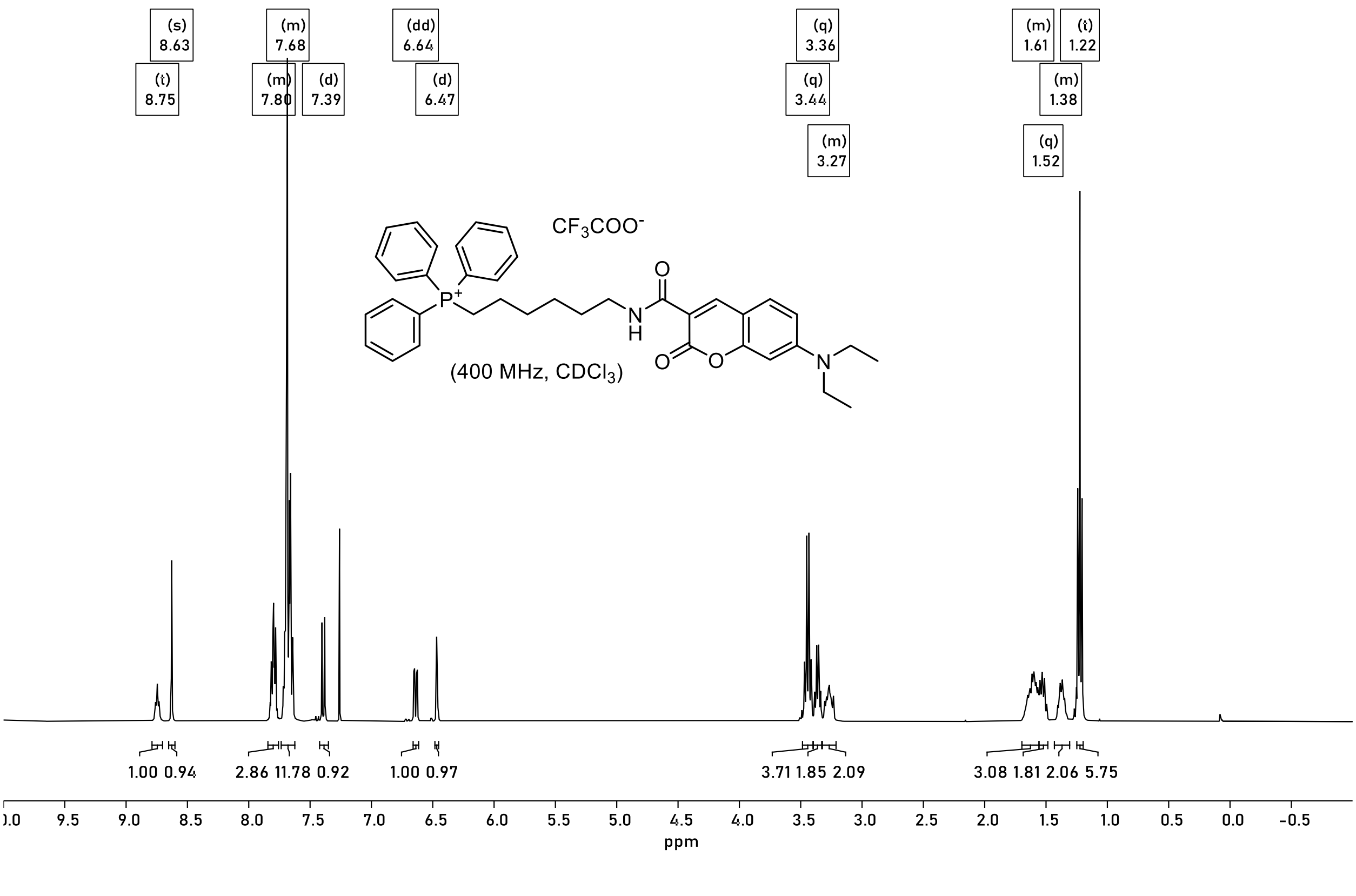
(s) 8.63
(t) 8.75
(m) 7.68
(m) 7.80
(d) 7.39
(dd) 6.64
(d) 6.47
(q) 3.36
(q) 3.44
(m) 3.27
(m) 1.61
(t) 1.22
(m) 1.38
(q) 1.52
CF_3COO^-
(400 MHz, $CDCl_3$)
1.00 0.94
2.86 11.78 0.92
1.00 0.97
3.71 1.85 2.09
3.08 1.81 2.06 5.75
ppm

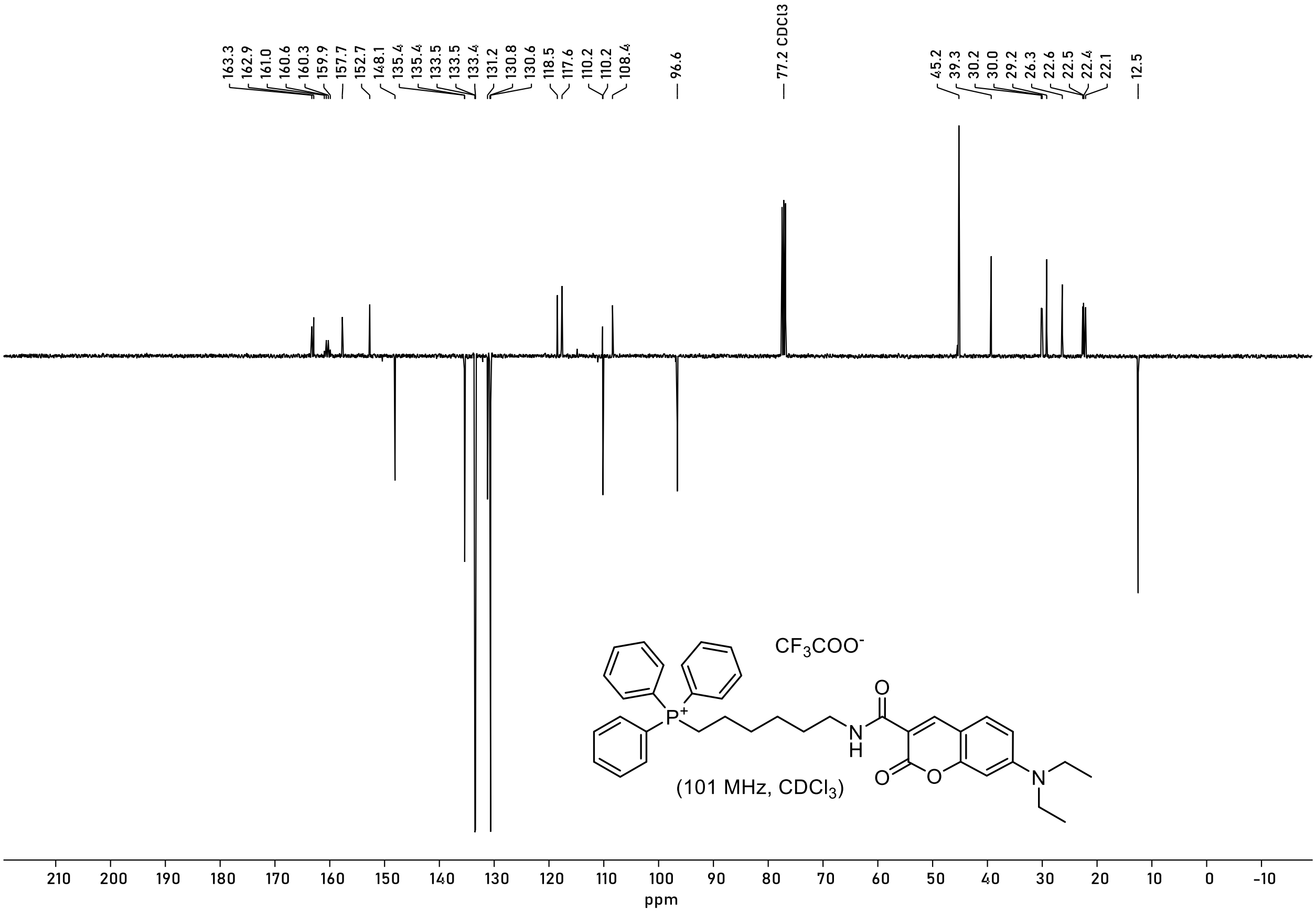

163.3
162.9
161.0
160.6
160.3
159.9
157.7
152.7
148.1
135.4
135.4
133.5
133.5
133.4
131.2
130.8
130.6
118.5
117.6
110.2
110.2
108.4
96.6
77.2 CDCl3
45.2
39.3
30.2
30.0
29.2
26.3
22.6
22.5
22.4
22.1
12.5
CF3COO-
(101 MHz, CDCl3)
ppm

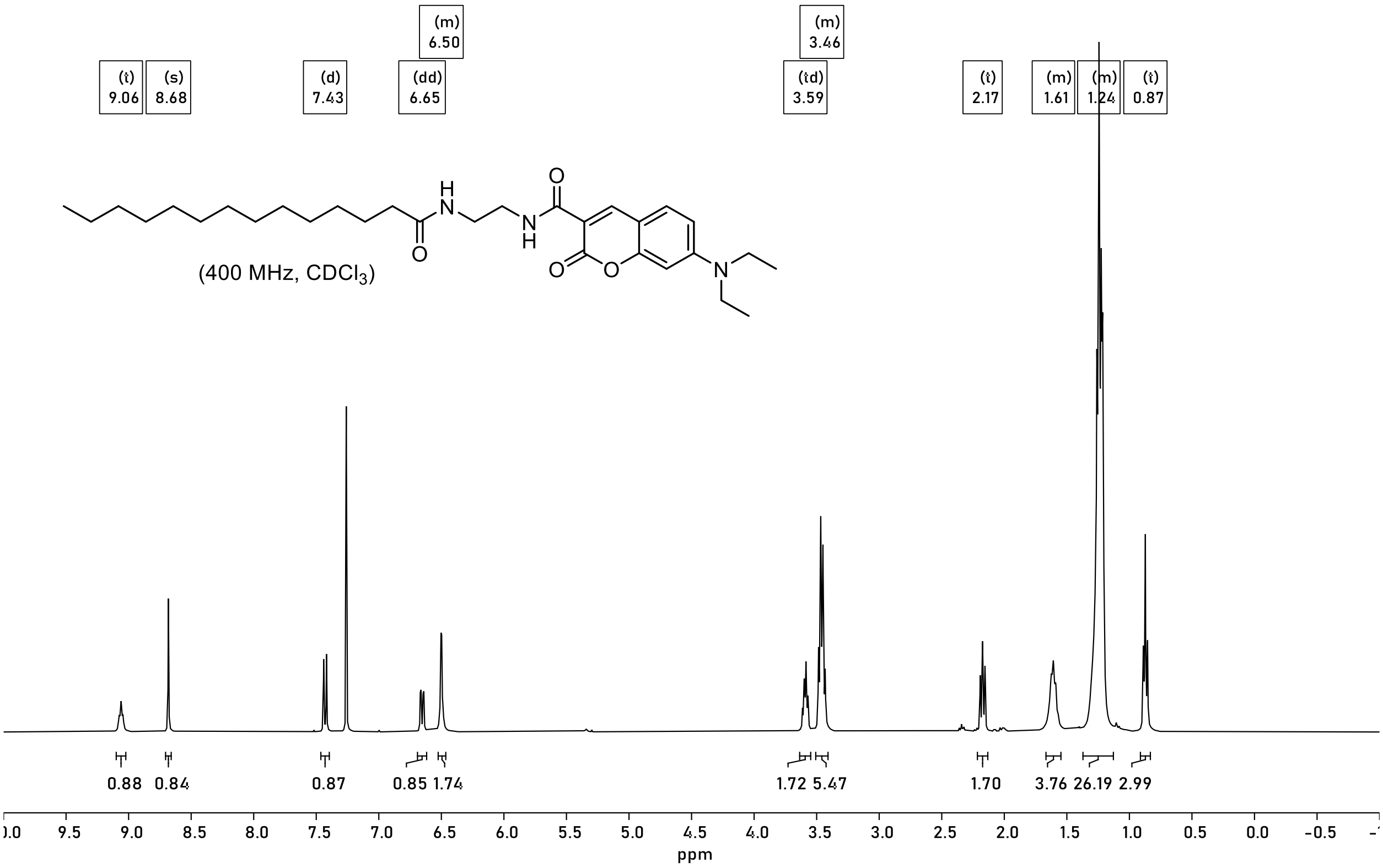

(t) 9.06
(s) 8.68
(d) 7.43
(m) 6.50
(dd) 6.65
(m) 3.46
(td) 3.59
(t) 2.17
(m) 1.61
(m) 1.24
(t) 0.87
(400 MHz, CDCl3)
0.88
0.84
0.87
0.85
1.74
1.72
5.47
1.70
3.76
26.19
2.99
ppm

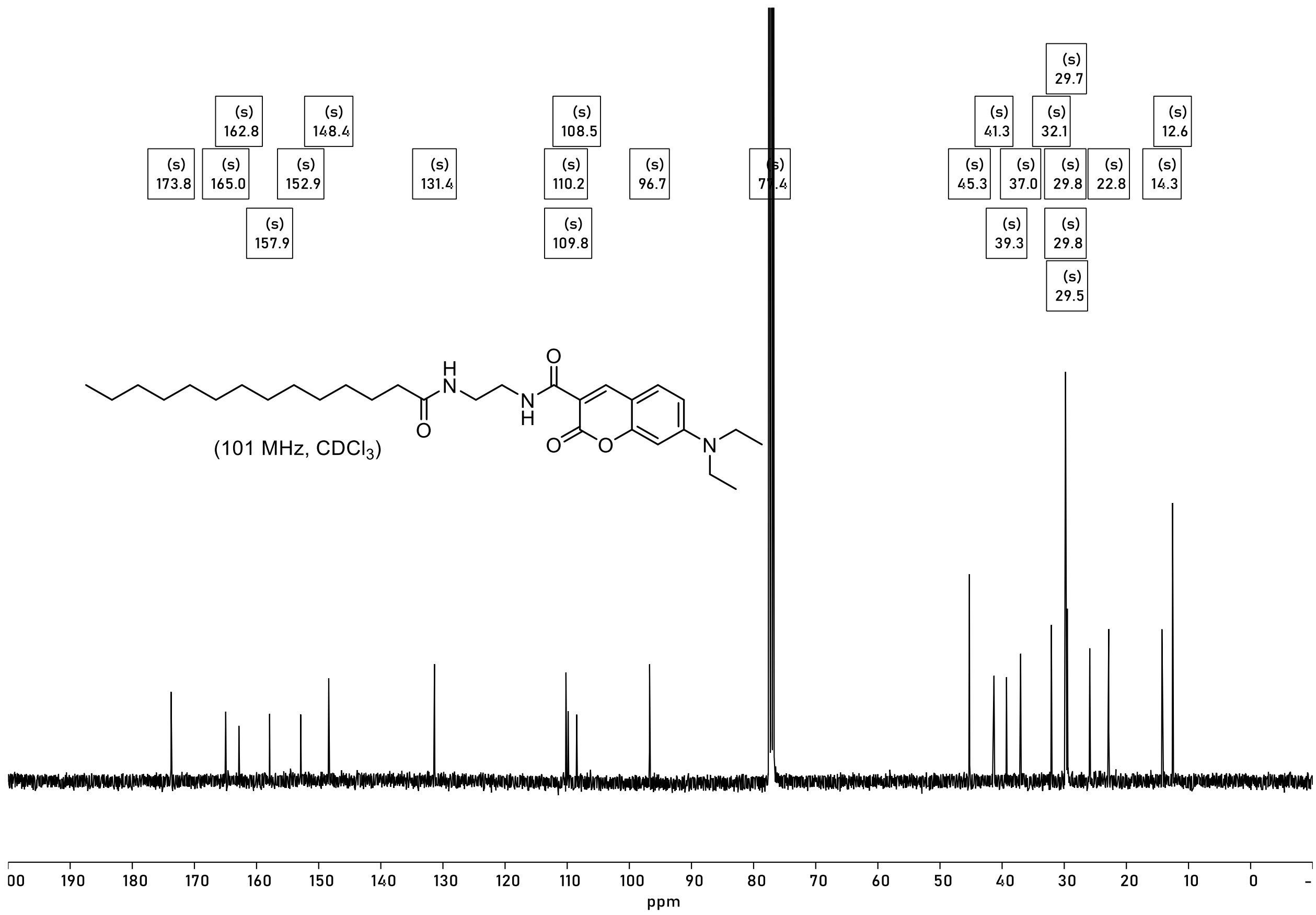
(s) 173.8
(s) 165.0
(s) 162.8
(s) 157.9
(s) 152.9
(s) 148.4
(s) 131.4
(s) 110.2
(s) 109.8
(s) 108.5
(s) 96.7
(s) 77.4
(s) 45.3
(s) 41.3
(s) 39.3
(s) 37.0
(s) 32.1
(s) 29.8
(s) 29.8
(s) 29.7
(s) 29.5
(s) 22.8
(s) 14.3
(s) 12.6
(101 MHz, CDCl3)
ppm

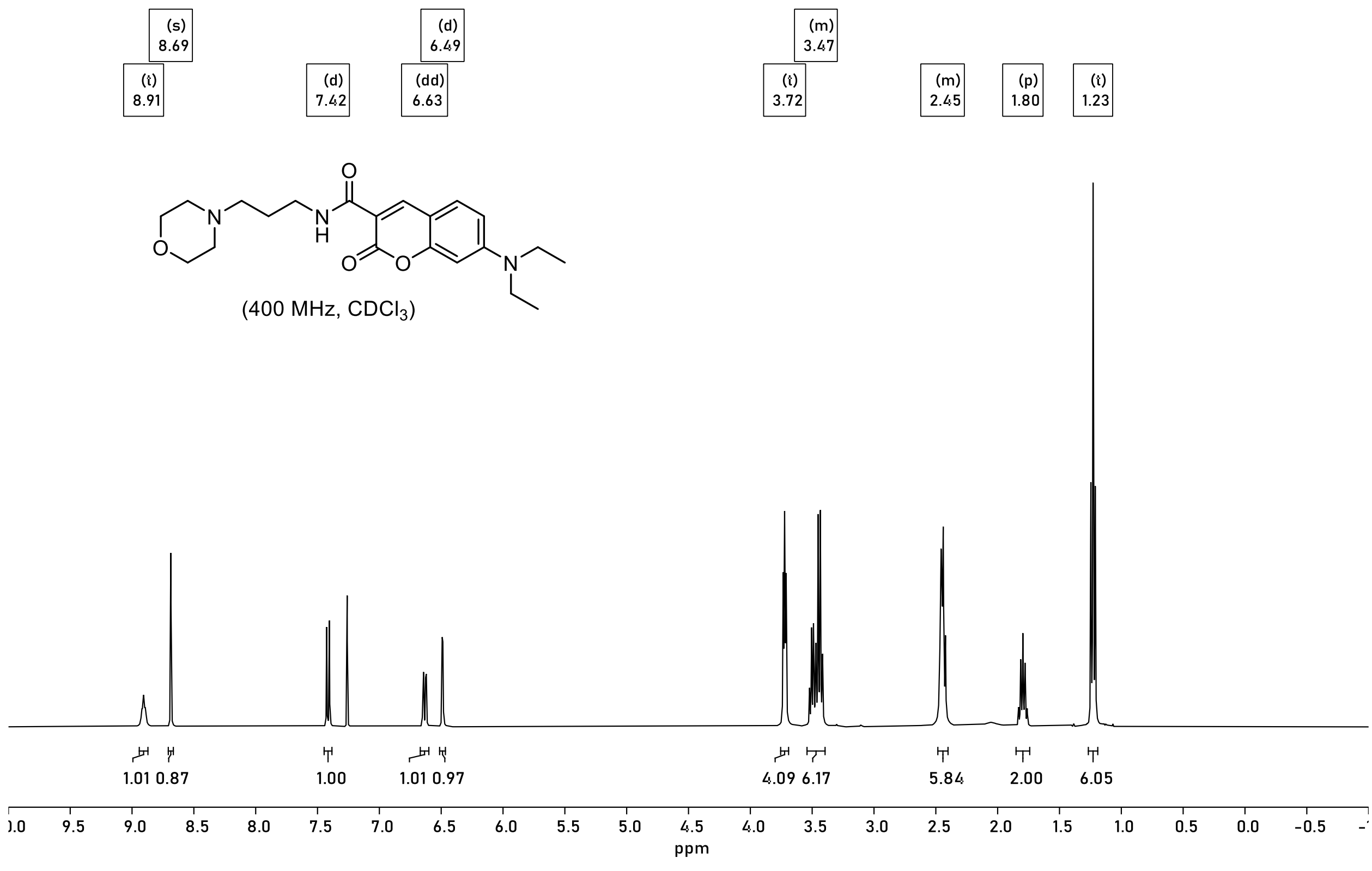
(t) 8.91
(s) 8.69
(d) 7.42
(dd) 6.63
(d) 6.49
(t) 3.72
(m) 3.47
(m) 2.45
(p) 1.80
(t) 1.23
(400 MHz, CDCl3)
1.01 0.87
1.00
1.01 0.97
4.09 6.17
5.84
2.00
6.05
ppm

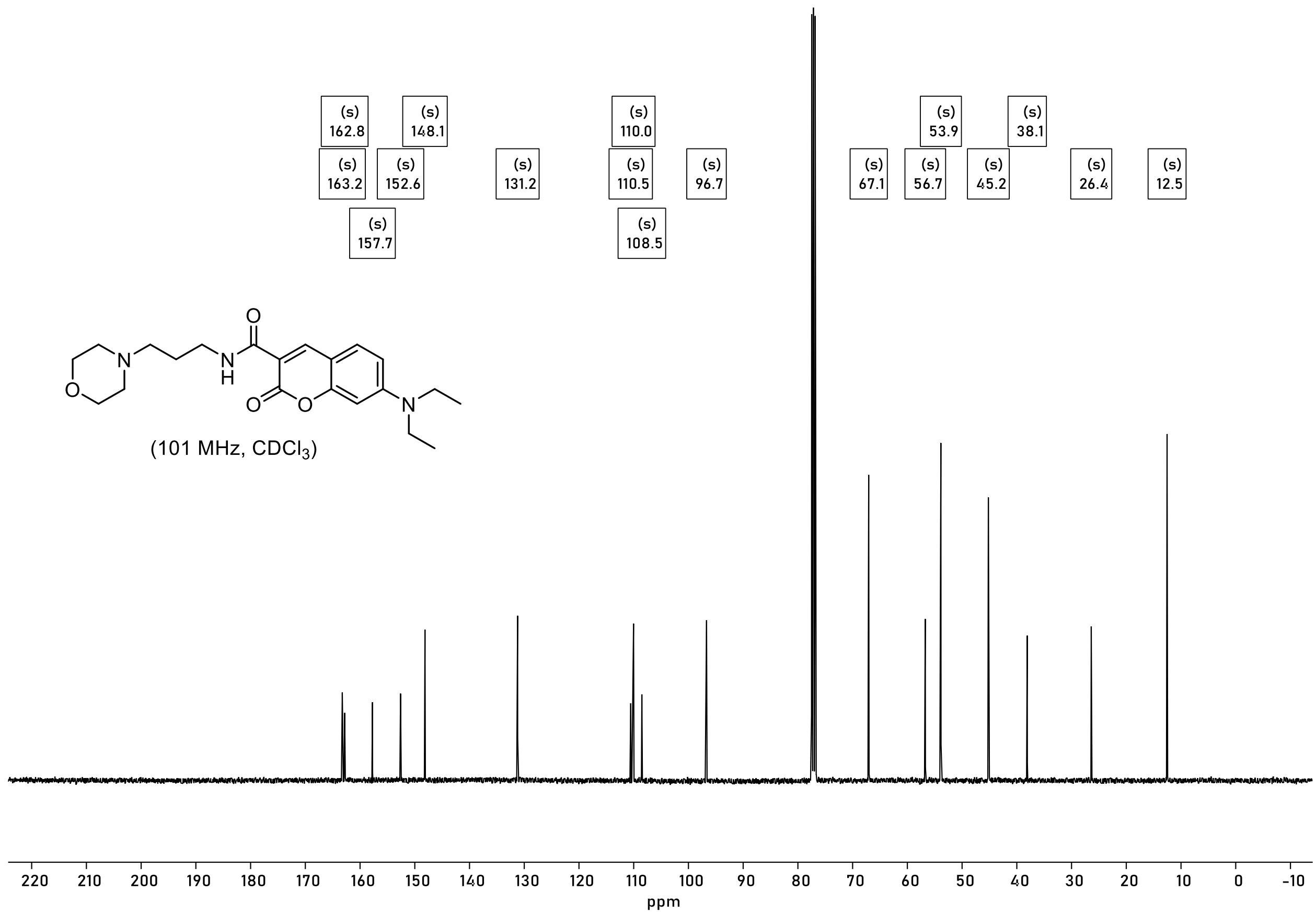
(s)
162.8
(s)
148.1
(s)
110.0
(s)
53.9
(s)
38.1
(s)
163.2
(s)
152.6
(s)
131.2
(s)
110.5
(s)
96.7
(s)
67.1
(s)
56.7
(s)
45.2
(s)
26.4
(s)
12.5
(s)
157.7
(s)
108.5
(101 MHz, CDCl3)
ppm

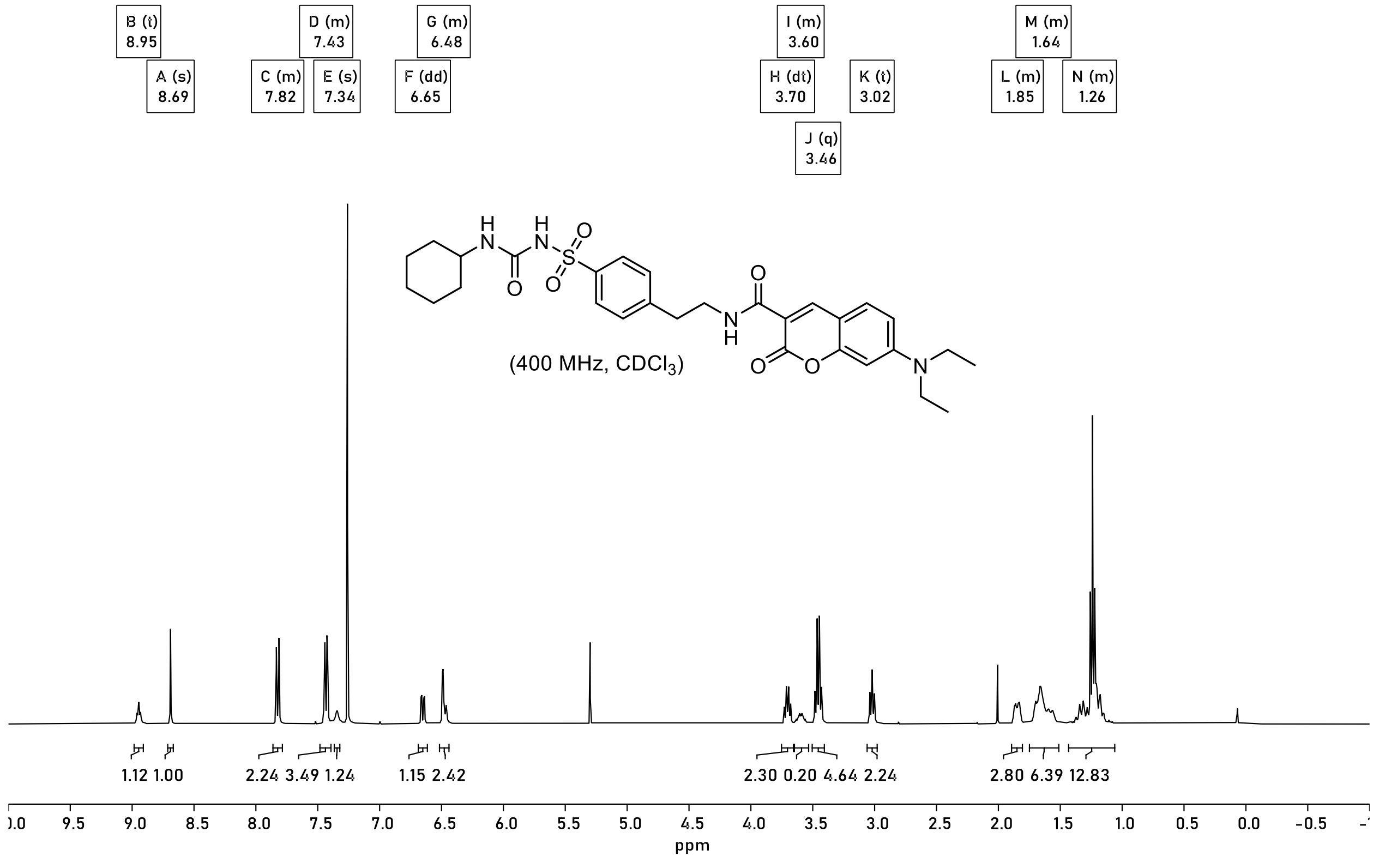
B (t)
8.95
D (m)
7.43
G (m)
6.48
I (m)
3.60
M (m)
1.64
A (s)
8.69
C (m)
7.82
E (s)
7.34
F (dd)
6.65
H (dt)
3.70
K (t)
3.02
L (m)
1.85
N (m)
1.26
J (q)
3.46
(400 MHz, CDCl3)
1.12 1.00 2.24 3.49 1.24 1.15 2.42 2.30 0.20 4.64 2.24 2.80 6.39 12.83
ppm

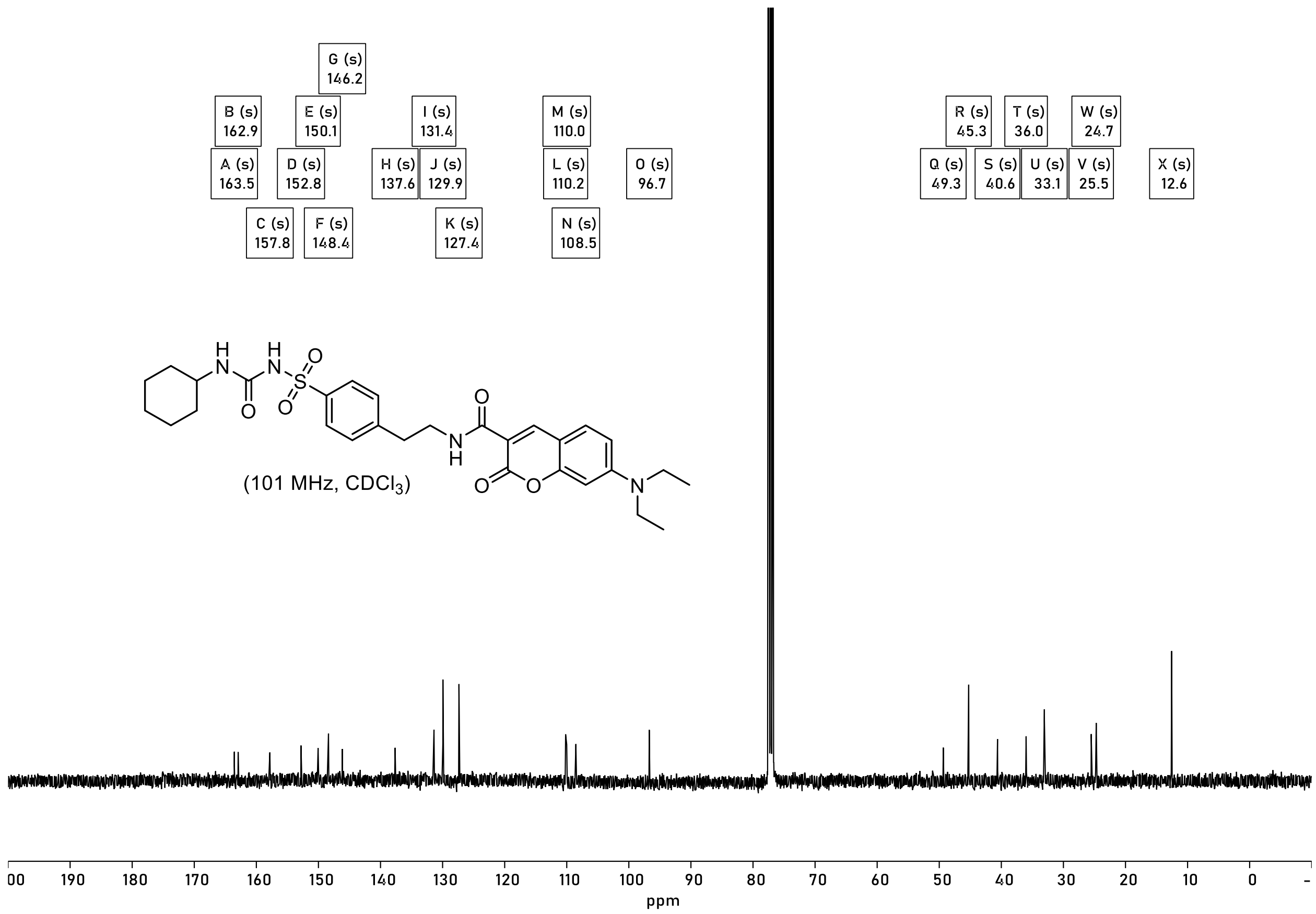
G (s)
146.2
B (s)
162.9
E (s)
150.1
I (s)
131.4
M (s)
110.0
R (s)
45.3
T (s)
36.0
W (s)
24.7
A (s)
163.5
D (s)
152.8
H (s)
137.6
J (s)
129.9
L (s)
110.2
O (s)
96.7
Q (s)
49.3
S (s)
40.6
U (s)
33.1
V (s)
25.5
X (s)
12.6
C (s)
157.8
F (s)
148.4
K (s)
127.4
N (s)
108.5
(101 MHz, CDCl3)
ppm

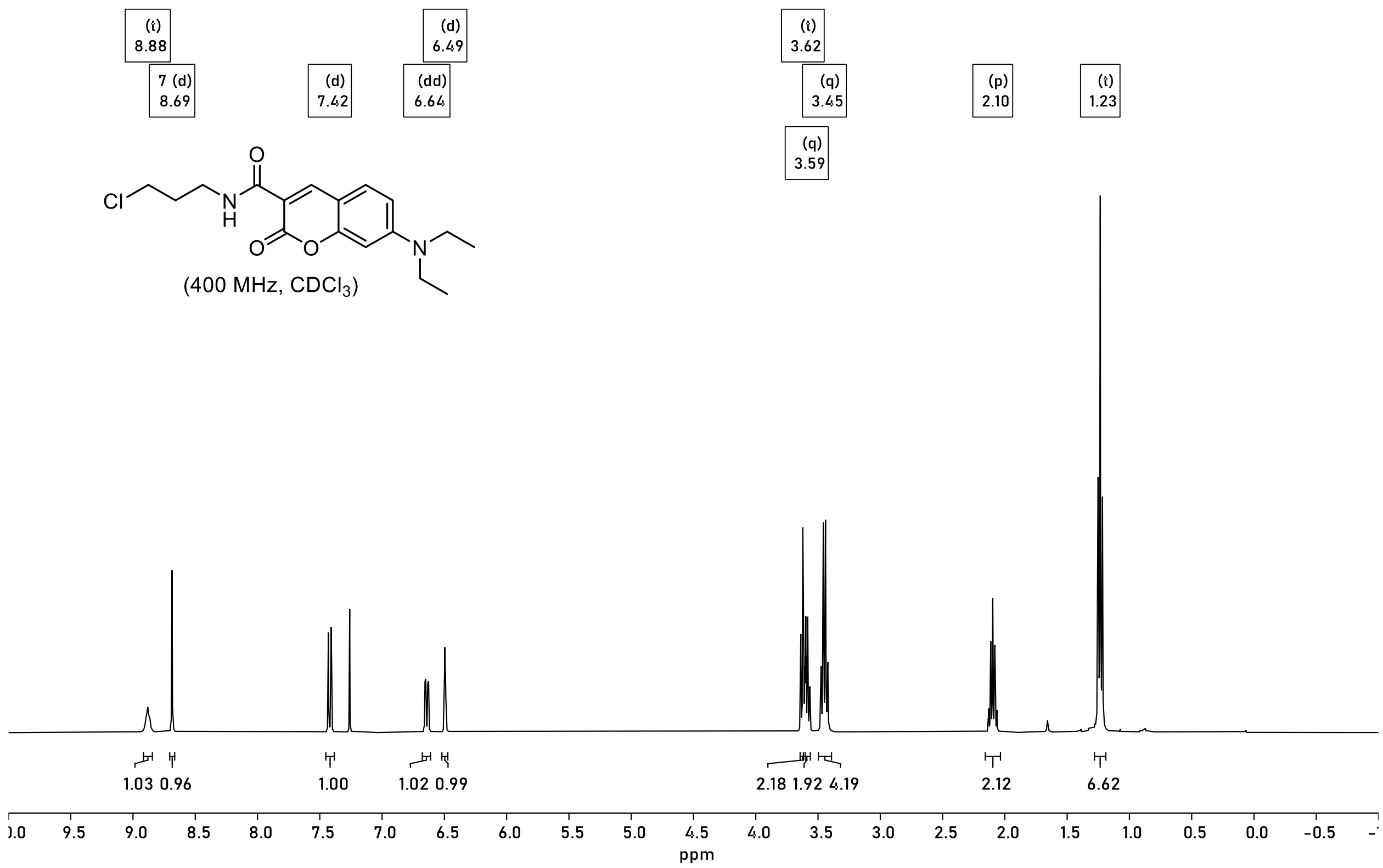
(t)
8.88
(d)
6.49
(t)
3.62
7 (d)
8.69
(d)
7.42
(dd)
6.64
(q)
3.45
(p)
2.10
(t)
1.23
(q)
3.59
(400 MHz, CDCl3)
1.03 0.96
1.00
1.02 0.99
2.18 1.92 4.19
2.12
6.62
ppm

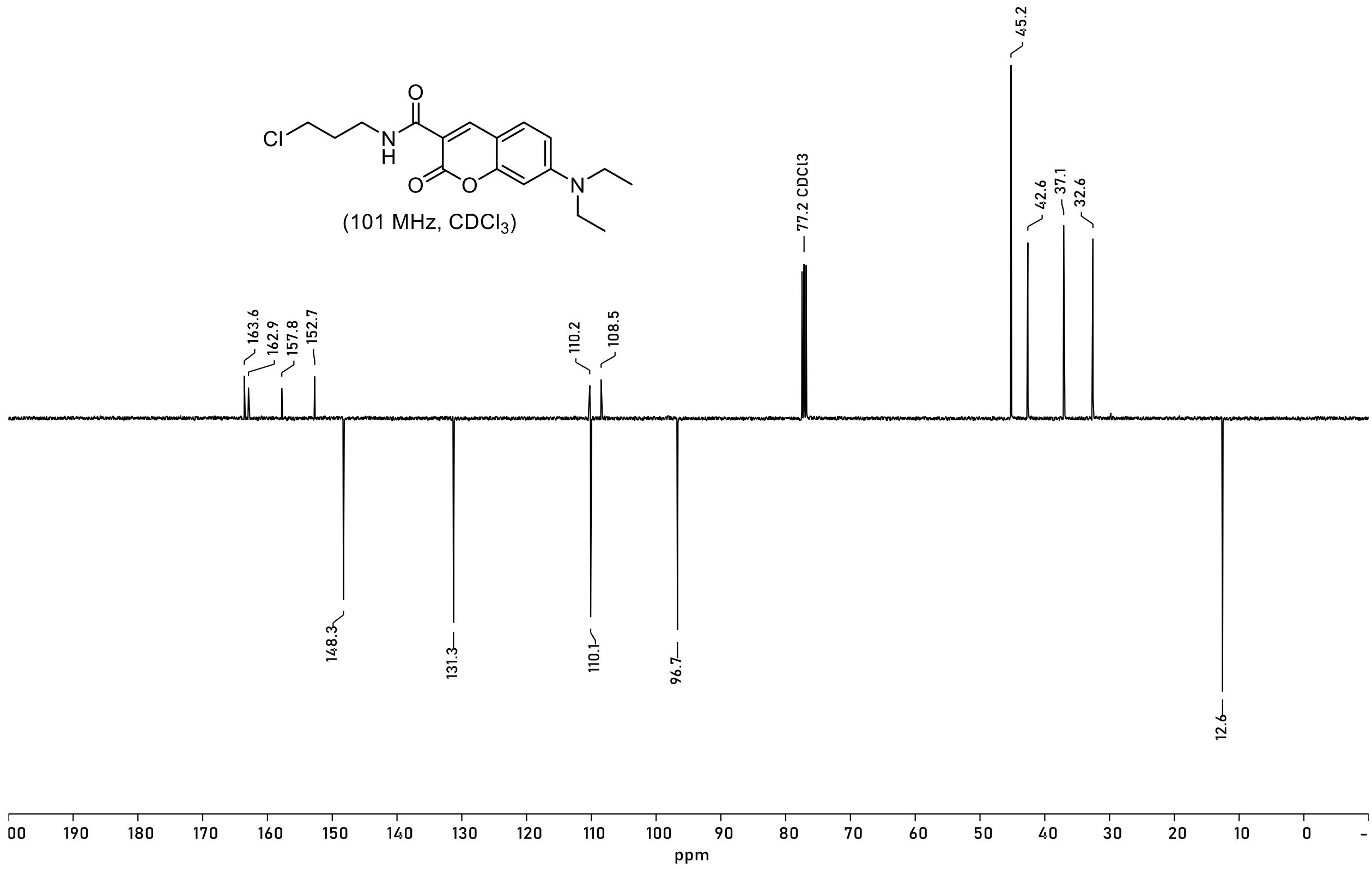
Cl
N
H
O
O
O
N
(101 MHz, CDCl3)
163.6
162.9
157.8
152.7
110.2
108.5
77.2 CDCl3
45.2
42.6
37.1
32.6
148.3
131.3
110.1
96.7
12.6
ppm

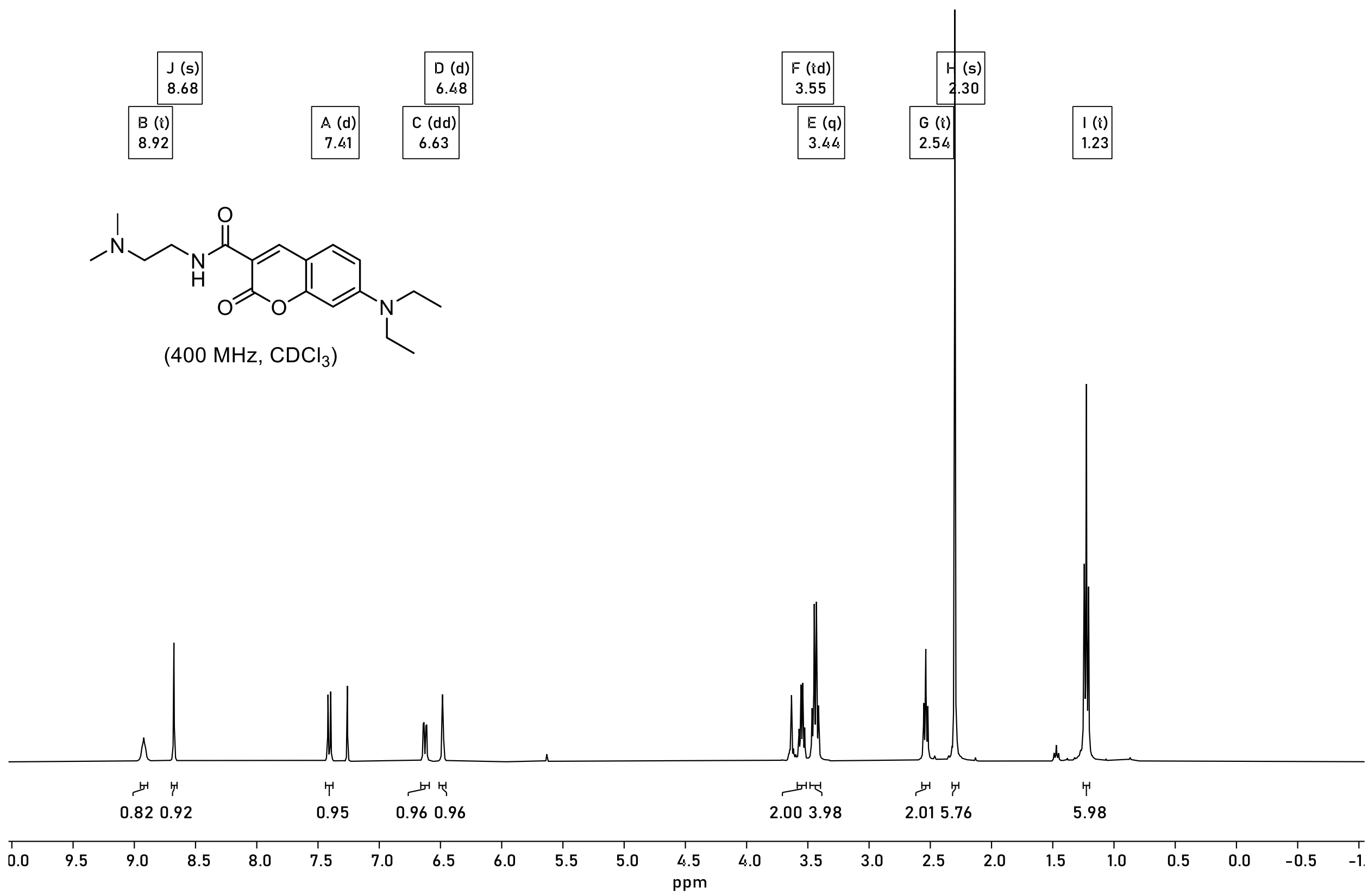
J (s)
8.68
B (t)
8.92
A (d)
7.41
D (d)
6.48
C (dd)
6.63
F (td)
3.55
E (q)
3.44
H (s)
2.30
G (t)
2.54
I (t)
1.23
N
N
H
O
O
O
N
(400 MHz, CDCl3)
0.82 0.92
0.95
0.96 0.96
2.00 3.98
2.01 5.76
5.98
ppm

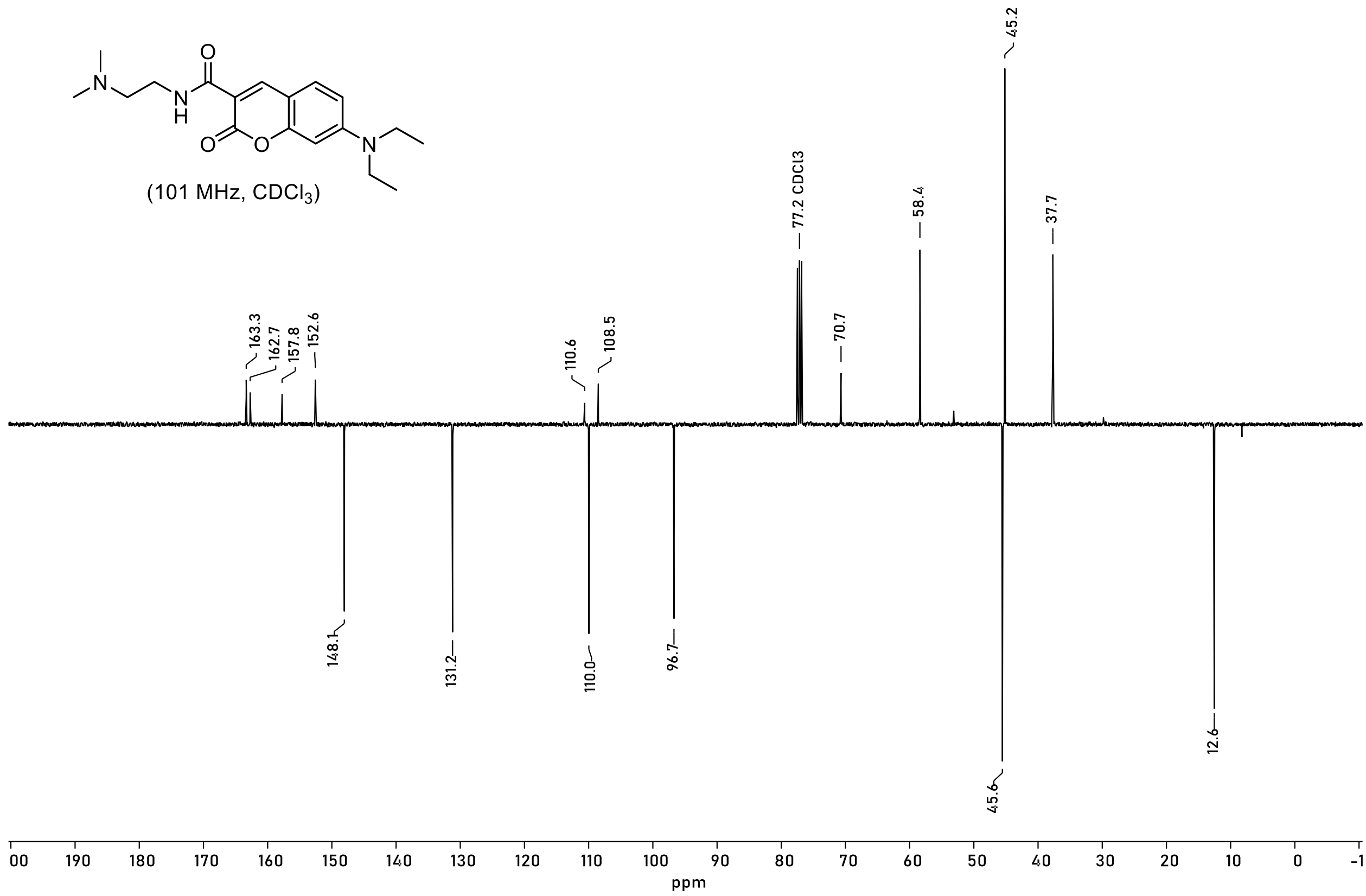
(101 MHz, CDCl3)
163.3
162.7
157.8
152.6
110.6
108.5
77.2 CDCl3
70.7
58.4
45.2
37.7
148.1
131.2
110.0
96.7
12.6
45.6
ppm

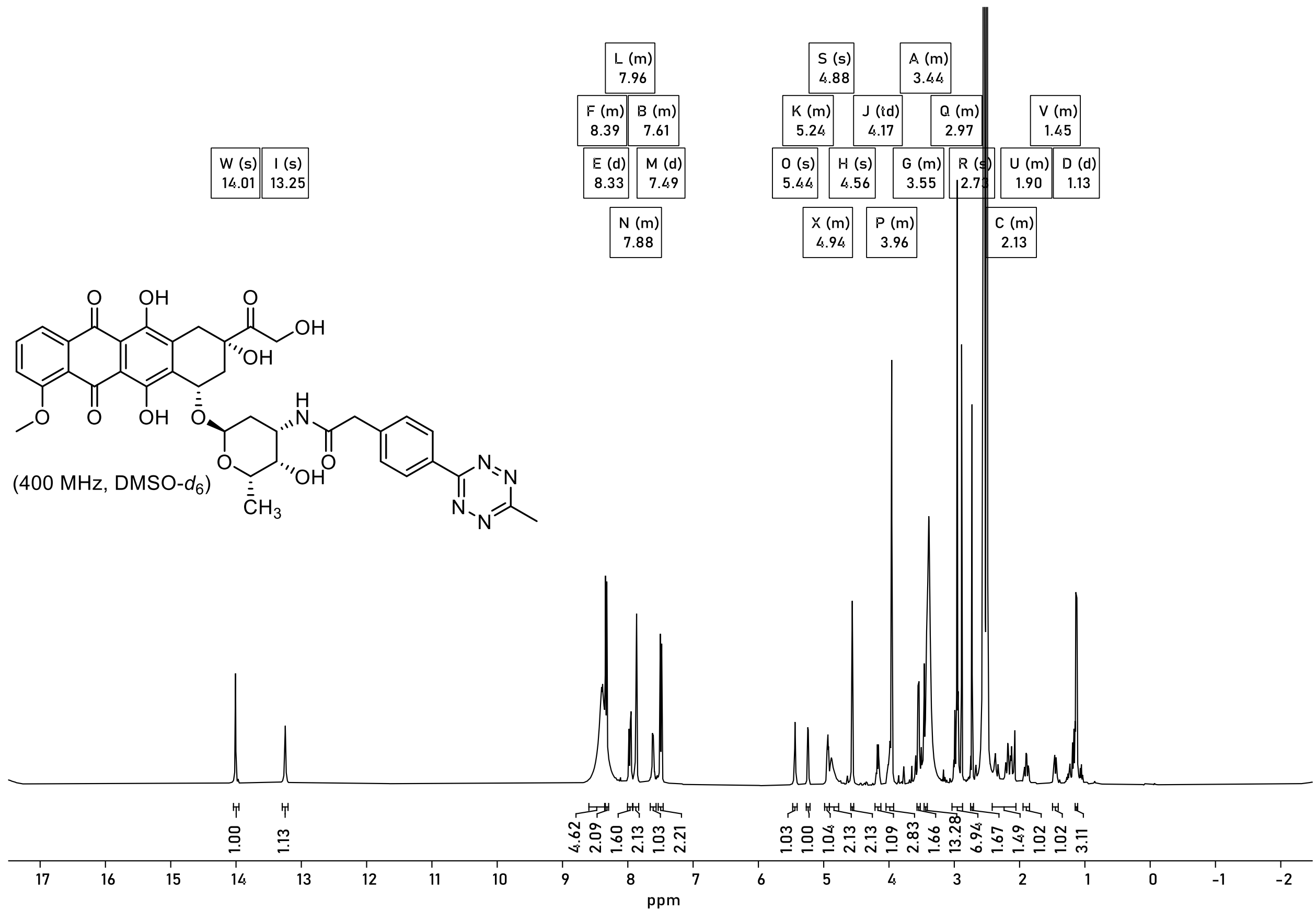
(400 MHz, DMSO-d6)
L (m) 7.96
F (m) 8.39
B (m) 7.61
E (d) 8.33
M (d) 7.49
N (m) 7.88
W (s) 14.01
I (s) 13.25
S (s) 4.88
A (m) 3.44
K (m) 5.24
J (td) 4.17
Q (m) 2.97
V (m) 1.45
O (s) 5.44
H (s) 4.56
G (m) 3.55
R (s) 2.73
U (m) 1.90
D (d) 1.13
X (m) 4.94
P (m) 3.96
C (m) 2.13
1.00
1.13
4.62
2.09
1.60
2.13
1.03
2.21
1.03
1.00
1.04
2.13
2.13
1.09
2.83
1.66
13.28
6.94
1.67
1.49
1.02
1.02
3.11
ppm

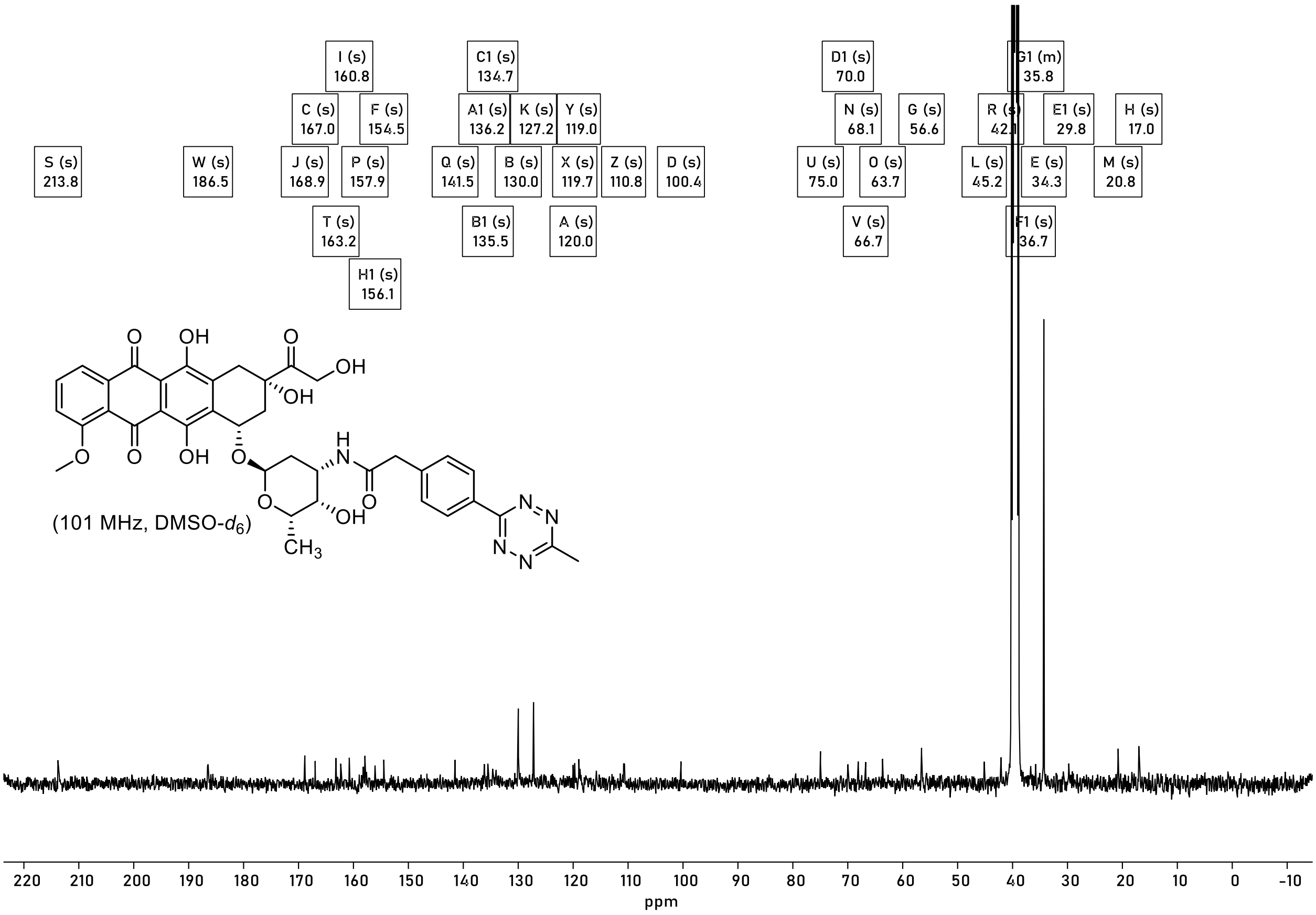

I (s) 160.8
C1 (s) 134.7
D1 (s) 70.0
G1 (m) 35.8
C (s) 167.0
F (s) 154.5
A1 (s) 136.2
K (s) 127.2
Y (s) 119.0
N (s) 68.1
G (s) 56.6
E1 (s) 29.8
H (s) 17.0
S (s) 213.8
W (s) 186.5
J (s) 168.9
P (s) 157.9
Q (s) 141.5
B (s) 130.0
X (s) 119.7
Z (s) 110.8
D (s) 100.4
U (s) 75.0
O (s) 63.7
L (s) 45.2
E (s) 34.3
M (s) 20.8
T (s) 163.2
B1 (s) 135.5
A (s) 120.0
V (s) 66.7
F1 (s) 36.7
H1 (s) 156.1
(101 MHz, DMSO-d_6)
ppm

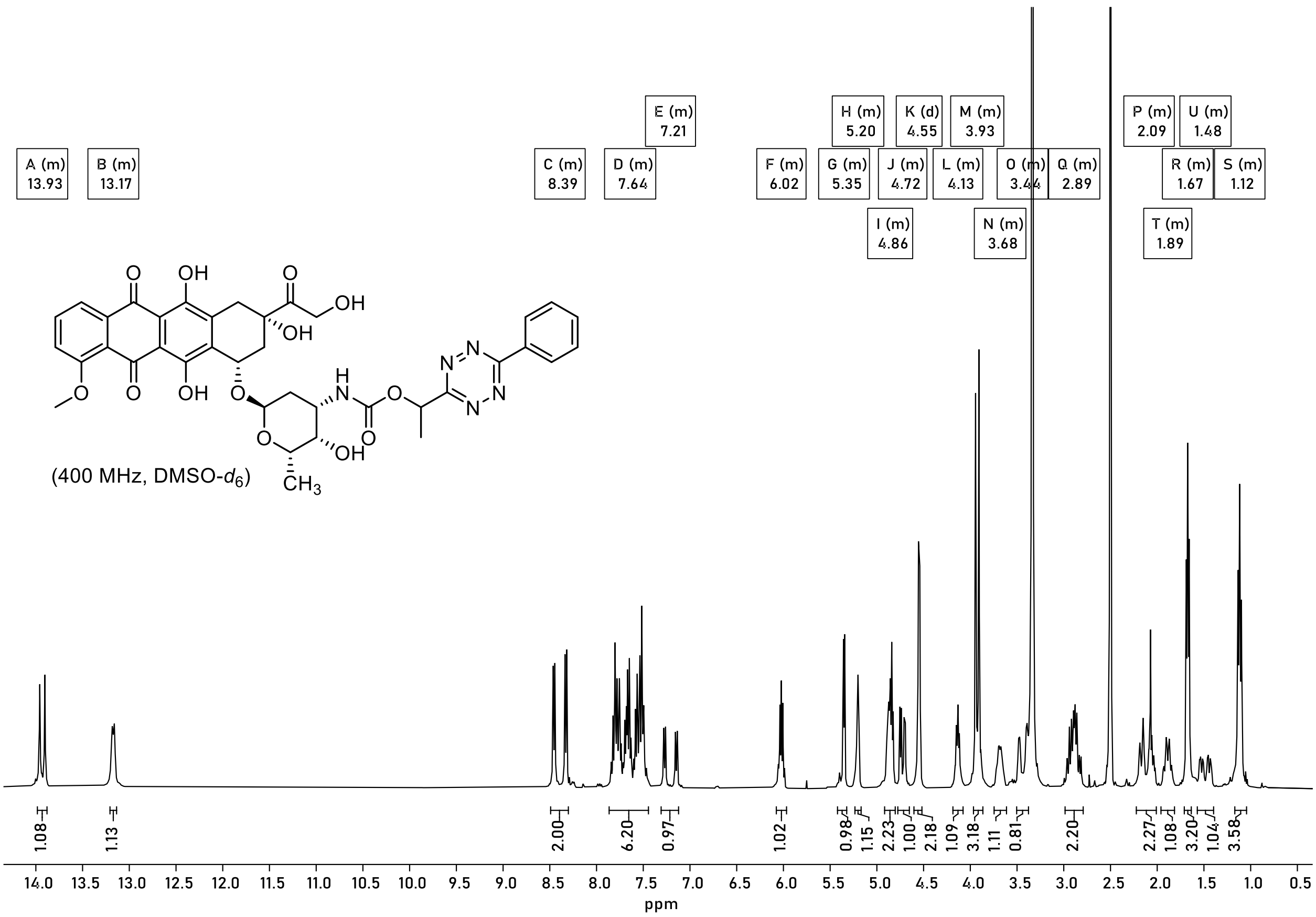

E (m) 7.21
H (m) 5.20
K (d) 4.55
M (m) 3.93
P (m) 2.09
U (m) 1.48
A (m) 13.93
B (m) 13.17
C (m) 8.39
D (m) 7.64
F (m) 6.02
G (m) 5.35
J (m) 4.72
L (m) 4.13
O (m) 3.44
Q (m) 2.89
R (m) 1.67
S (m) 1.12
I (m) 4.86
N (m) 3.68
T (m) 1.89
(400 MHz, DMSO-d_6)
ppm

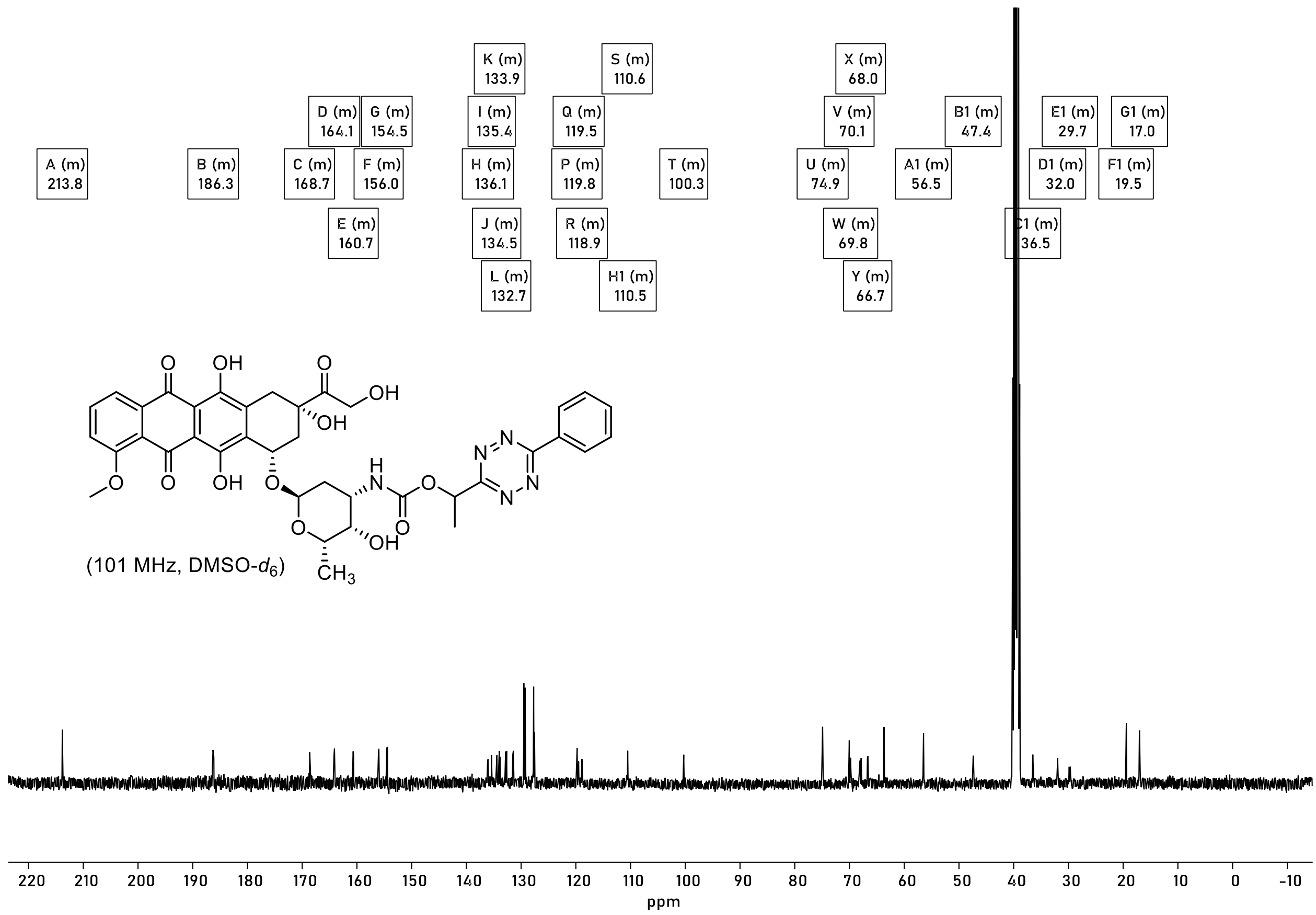
A (m) 213.8
B (m) 186.3
C (m) 168.7
D (m) 164.1
E (m) 160.7
F (m) 156.0
G (m) 154.5
H (m) 136.1
I (m) 135.4
J (m) 134.5
K (m) 133.9
L (m) 132.7
P (m) 119.8
Q (m) 119.5
R (m) 118.9
S (m) 110.6
H1 (m) 110.5
T (m) 100.3
U (m) 74.9
V (m) 70.1
W (m) 69.8
X (m) 68.0
Y (m) 66.7
A1 (m) 56.5
B1 (m) 47.4
C1 (m) 36.5
D1 (m) 32.0
E1 (m) 29.7
F1 (m) 19.5
G1 (m) 17.0
(101 MHz, DMSO-d_6)
ppm

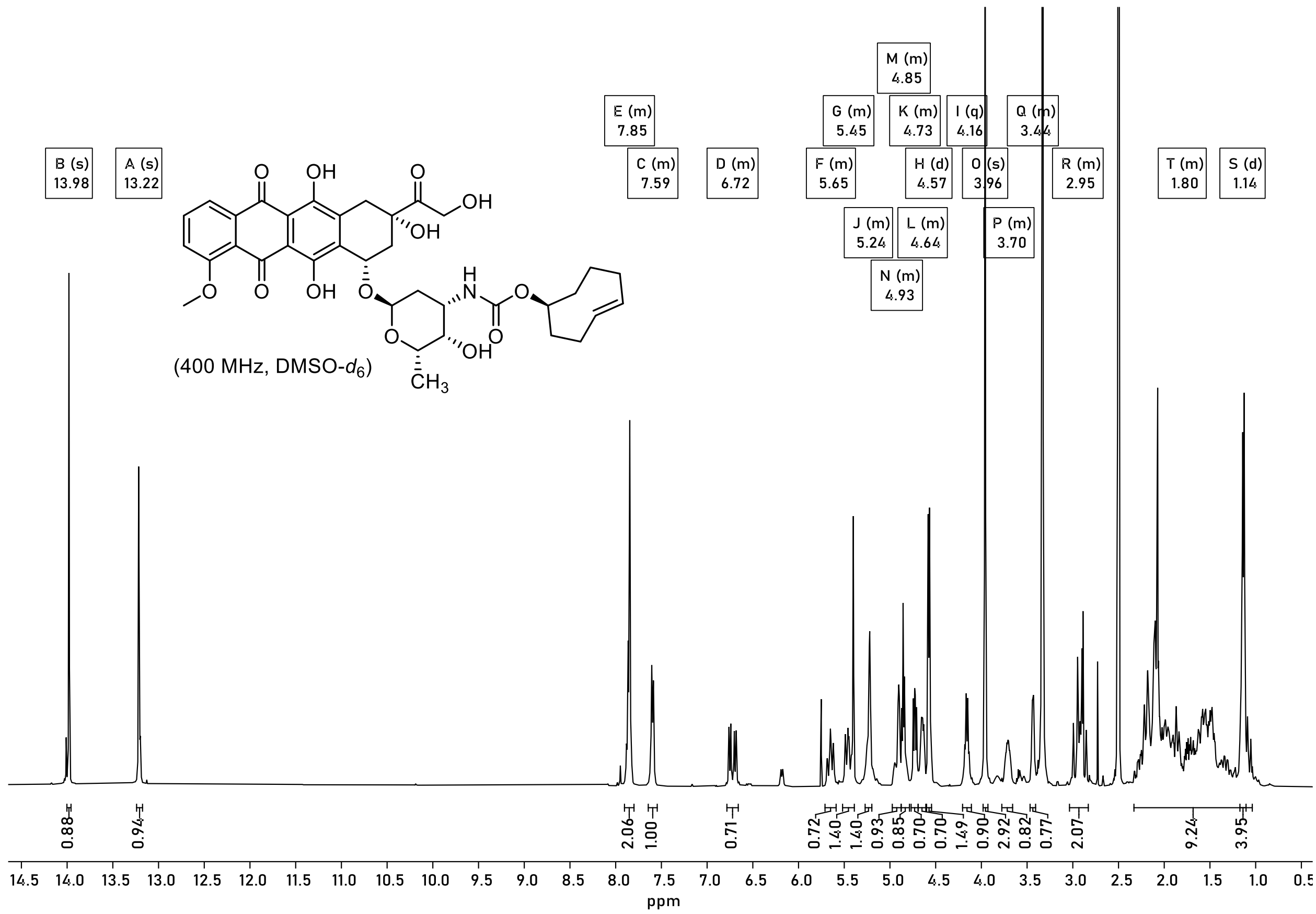
B (s) 13.98
A (s) 13.22
E (m) 7.85
C (m) 7.59
D (m) 6.72
F (m) 5.65
G (m) 5.45
J (m) 5.24
N (m) 4.93
M (m) 4.85
K (m) 4.73
L (m) 4.64
H (d) 4.57
I (q) 4.16
O (s) 3.96
P (m) 3.70
Q (m) 3.44
R (m) 2.95
T (m) 1.80
S (d) 1.14
(400 MHz, DMSO-d_6)
ppm

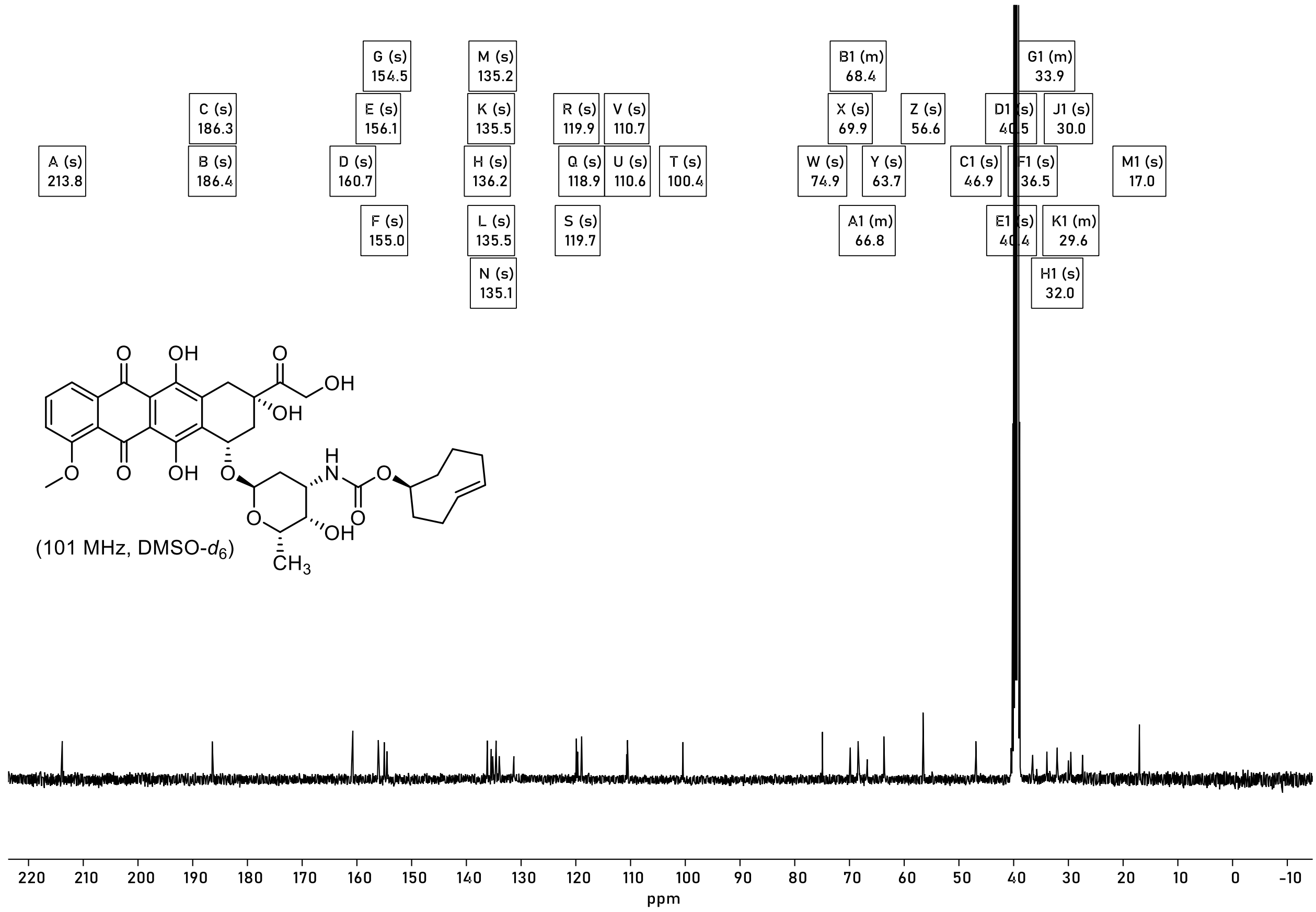
A (s) 213.8
B (s) 186.4
C (s) 186.3
D (s) 160.7
E (s) 156.1
F (s) 155.0
G (s) 154.5
H (s) 136.2
K (s) 135.5
L (s) 135.5
M (s) 135.2
N (s) 135.1
Q (s) 118.9
R (s) 119.9
S (s) 119.7
U (s) 110.6
V (s) 110.7
T (s) 100.4
W (s) 74.9
X (s) 69.9
B1 (m) 68.4
A1 (m) 66.8
Y (s) 63.7
Z (s) 56.6
C1 (s) 46.9
D1 (s) 40.5
E1 (s) 40.4
F1 (s) 36.5
G1 (m) 33.9
H1 (s) 32.0
J1 (s) 30.0
K1 (m) 29.6
M1 (s) 17.0
(101 MHz, DMSO-d6)
220 210 200 190 180 170 160 150 140 130 120 110 100 90 80 70 60 50 40 30 20 10 0 -10
ppm

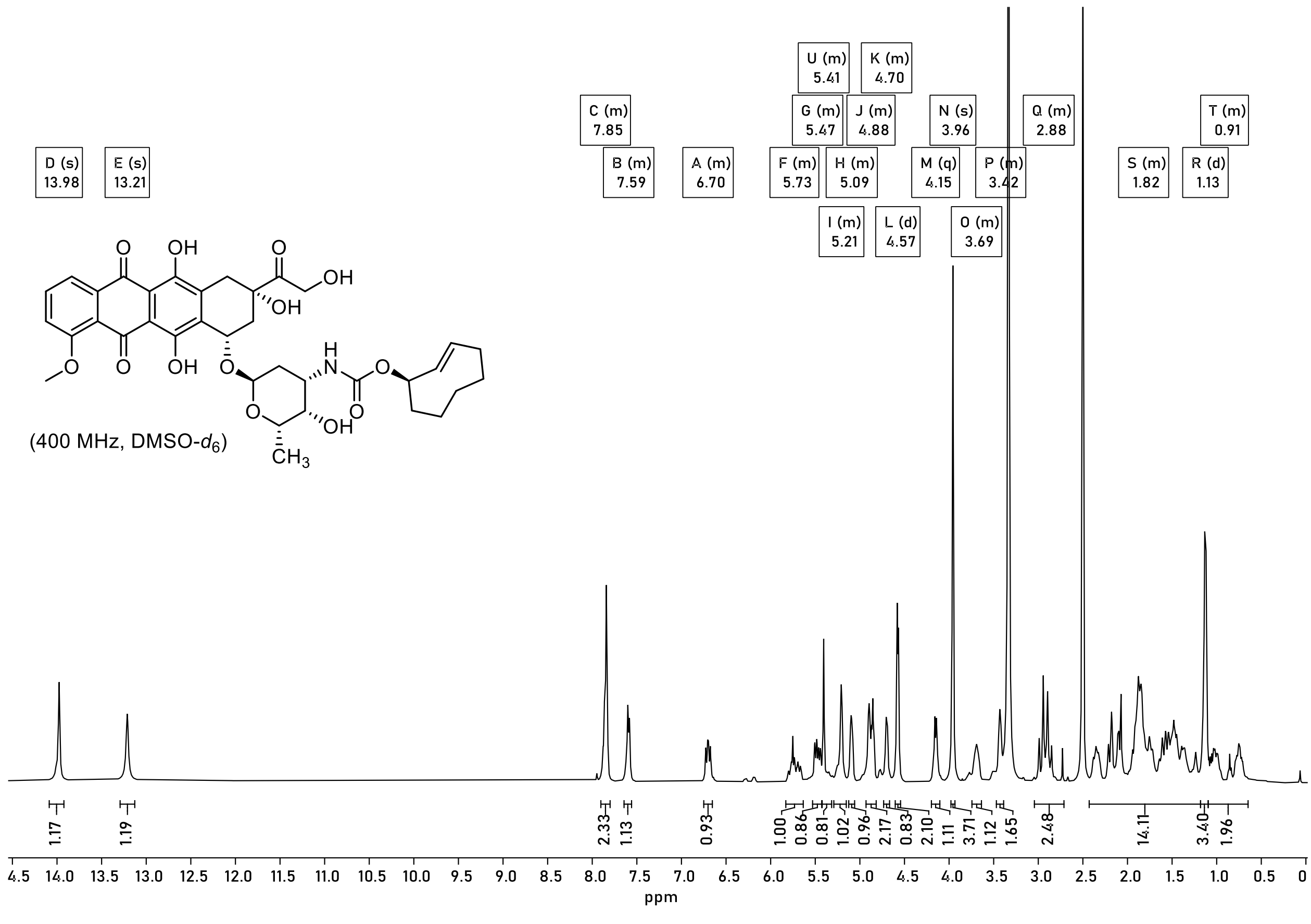
D (s) 13.98
E (s) 13.21
C (m) 7.85
B (m) 7.59
A (m) 6.70
F (m) 5.73
G (m) 5.47
U (m) 5.41
I (m) 5.21
H (m) 5.09
J (m) 4.88
K (m) 4.70
L (d) 4.57
M (q) 4.15
N (s) 3.96
O (m) 3.69
P (m) 3.42
Q (m) 2.88
S (m) 1.82
R (d) 1.13
T (m) 0.91
(400 MHz, DMSO-d6)
1.17 1.19 2.33 1.13 0.93 1.00 0.86 0.81 1.02 0.96 2.17 0.83 2.10 1.11 3.71 1.12 1.65 2.48 14.11 3.40 1.96
14.5 14.0 13.5 13.0 12.5 12.0 11.5 11.0 10.5 10.0 9.5 9.0 8.5 8.0 7.5 7.0 6.5 6.0 5.5 5.0 4.5 4.0 3.5 3.0 2.5 2.0 1.5 1.0 0.5 0
ppm

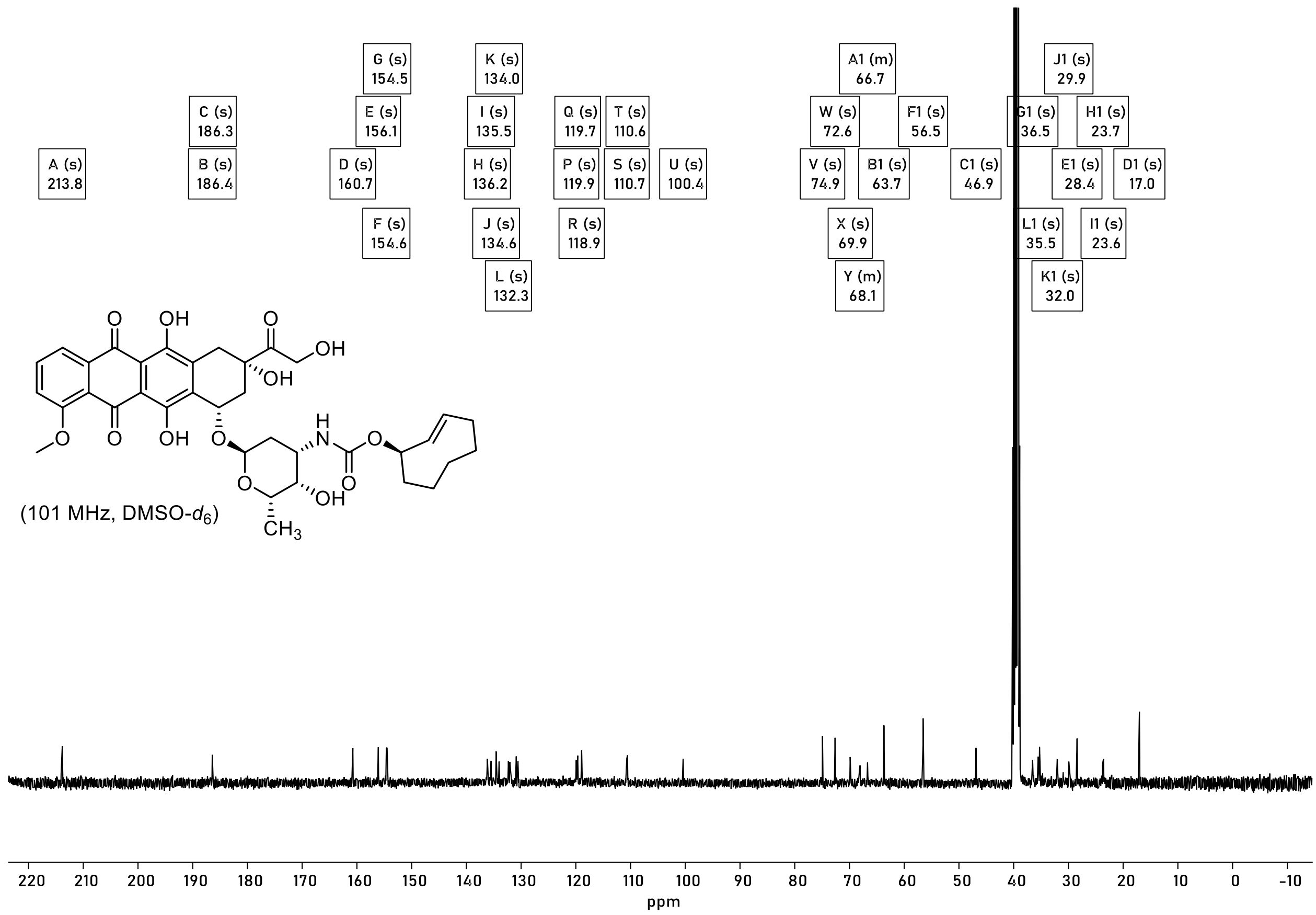
A (s) 213.8
C (s) 186.3
B (s) 186.4
G (s) 154.5
E (s) 156.1
D (s) 160.7
F (s) 154.6
K (s) 134.0
I (s) 135.5
H (s) 136.2
J (s) 134.6
L (s) 132.3
Q (s) 119.7
P (s) 119.9
R (s) 118.9
T (s) 110.6
S (s) 110.7
U (s) 100.4
A1 (m) 66.7
W (s) 72.6
V (s) 74.9
B1 (s) 63.7
X (s) 69.9
Y (m) 68.1
F1 (s) 56.5
C1 (s) 46.9
G1 (s) 36.5
L1 (s) 35.5
K1 (s) 32.0
J1 (s) 29.9
H1 (s) 23.7
E1 (s) 28.4
I1 (s) 23.6
D1 (s) 17.0
(101 MHz, DMSO-d_6)
ppm

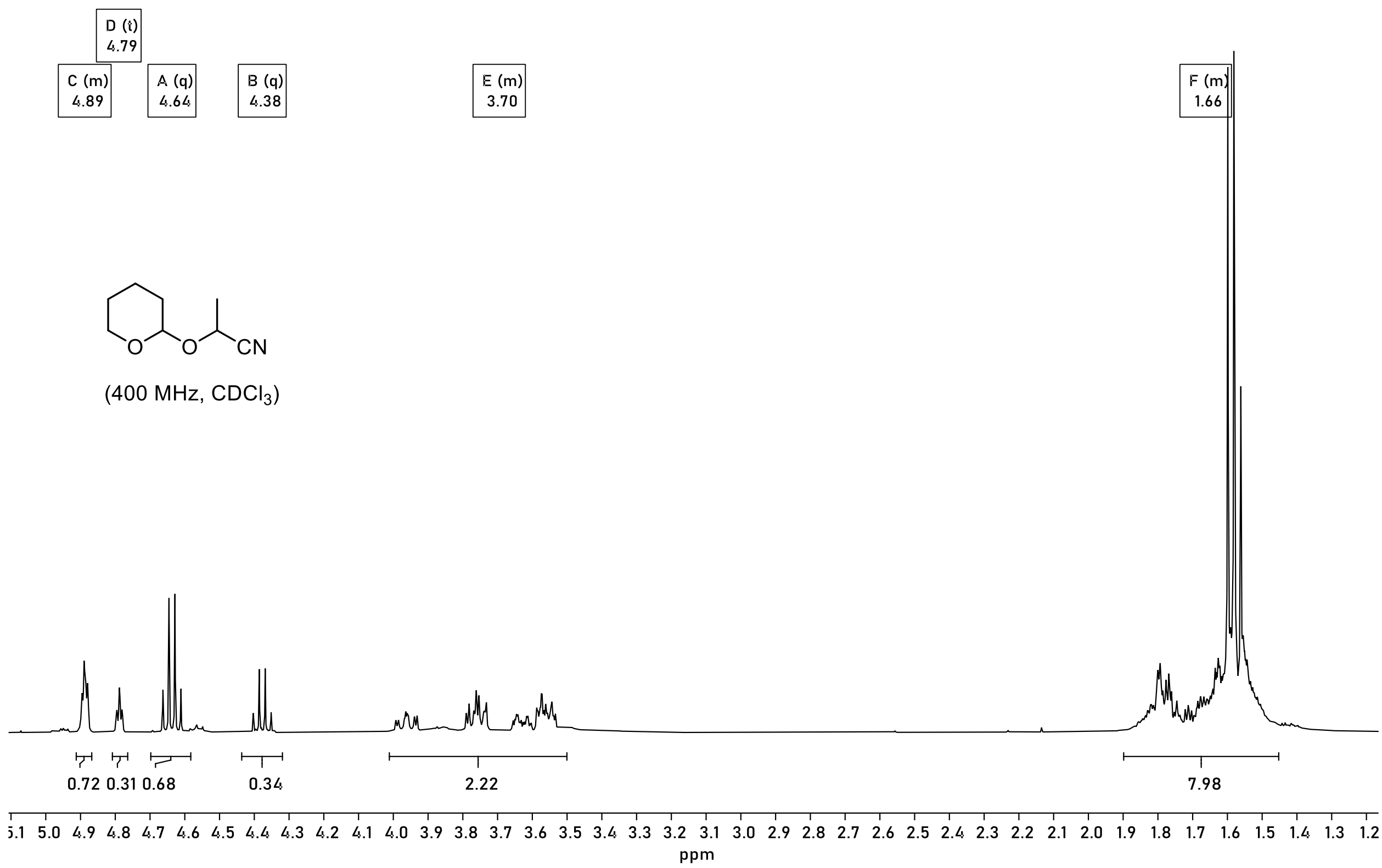
D (t) 4.79
C (m) 4.89
A (q) 4.64
B (q) 4.38
E (m) 3.70
F (m) 1.66
(400 MHz, $CDCl_3$)
0.72 0.31 0.68
0.34
2.22
7.98
ppm

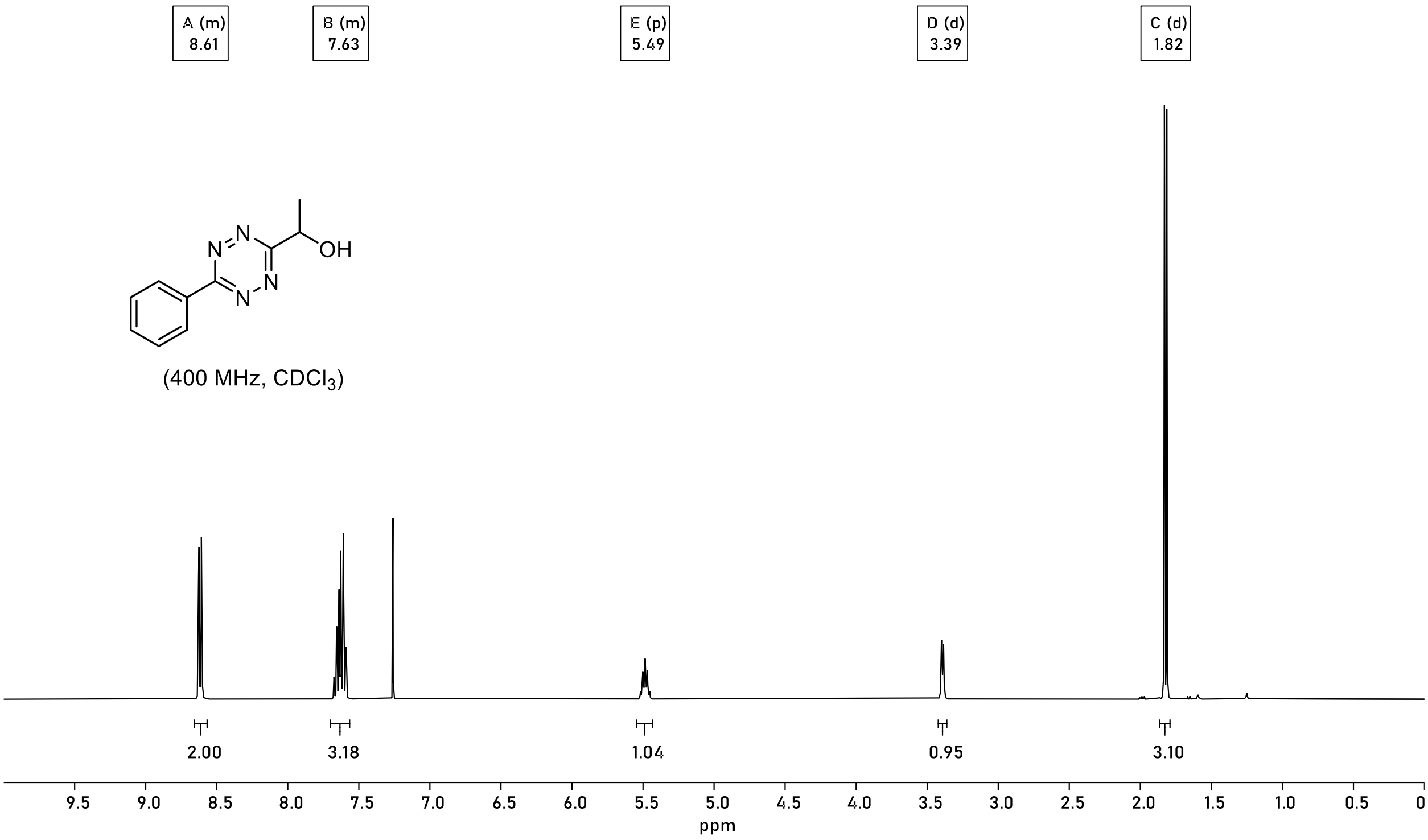

A (m)
8.61
B (m)
7.63
E (p)
5.49
D (d)
3.39
C (d)
1.82
OH
(400 MHz, CDCl3)
2.00
3.18
1.04
0.95
3.10
ppm

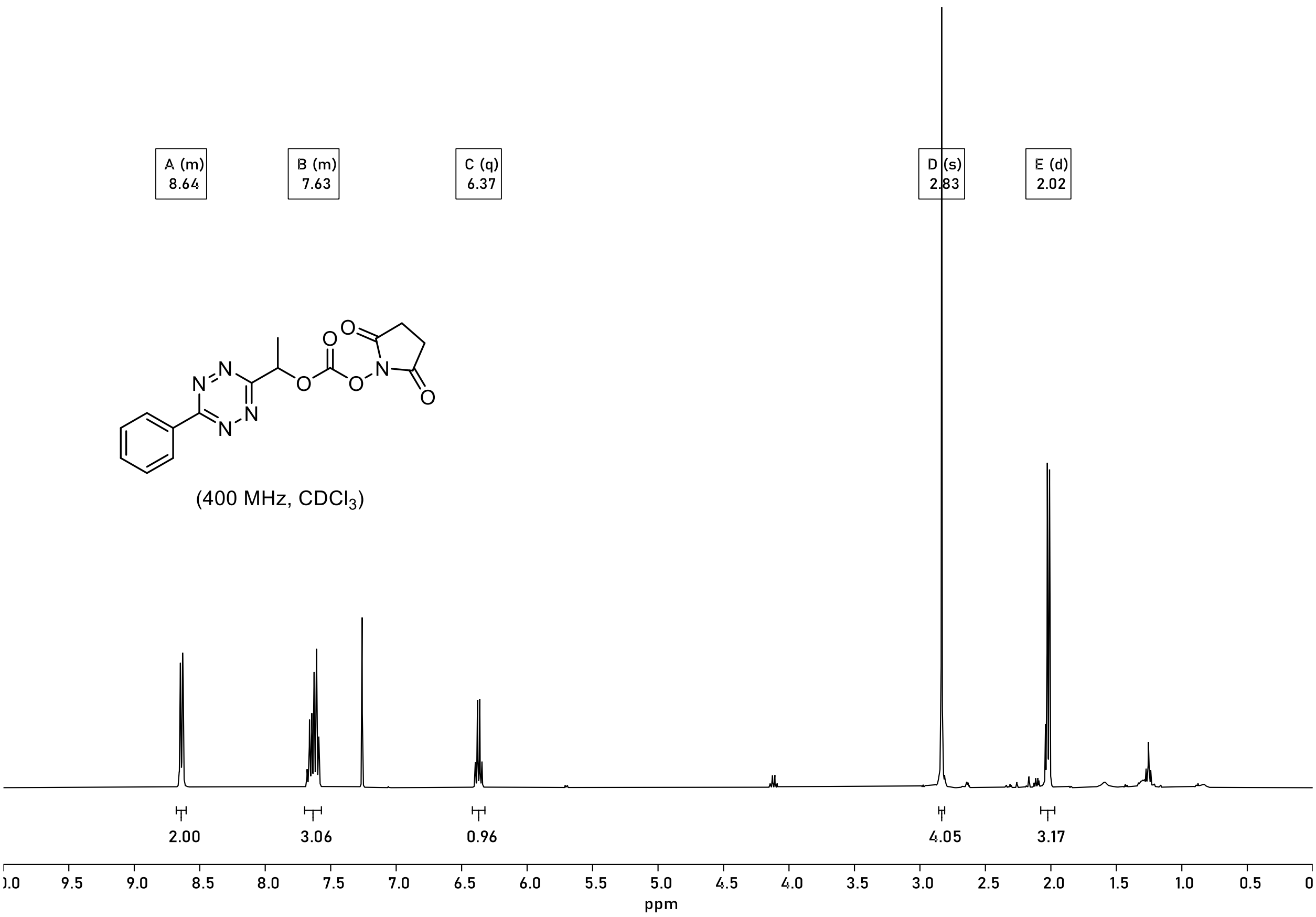

A (m)
8.64
B (m)
7.63
C (q)
6.37
D (s)
2.83
E (d)
2.02
(400 MHz, CDCl3)
2.00
3.06
0.96
4.05
3.17
ppm

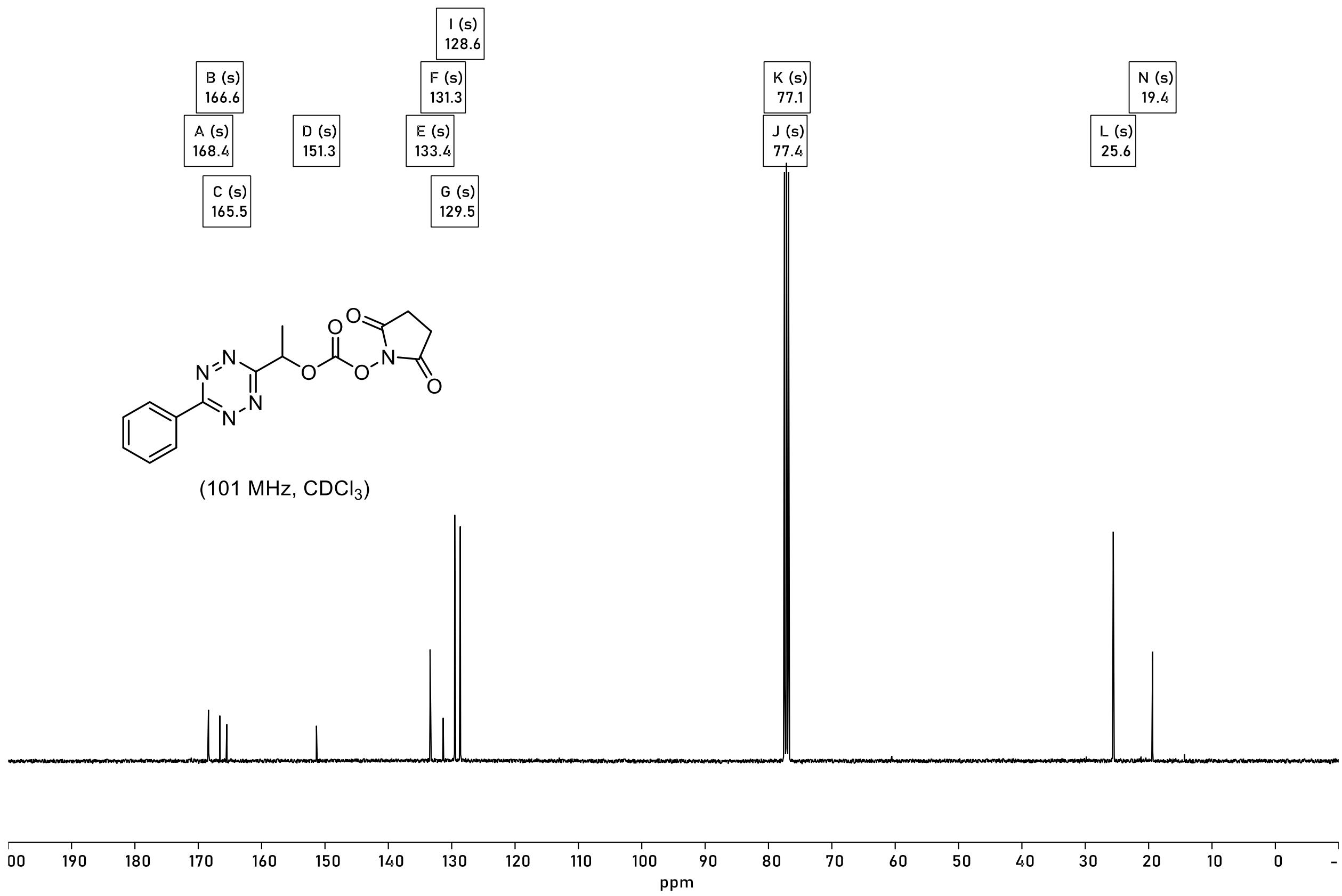

I (s)
128.6
B (s)
166.6
F (s)
131.3
K (s)
77.1
N (s)
19.4
A (s)
168.4
D (s)
151.3
E (s)
133.4
J (s)
77.4
L (s)
25.6
C (s)
165.5
G (s)
129.5
(101 MHz, CDCl3)
ppm
