# Supplementary material for: Organelle‐Resolved Tetrazine‐trans‐Cyclooctene Click Chemistry for Cargo Delivery and Release
Source: Chemistry. 2026 Jun 1;32(35):e71198. doi: 10.1002/chem.71198 (PMC13587637; doi:10.1002/chem.71198)
Supplement: Supplementary file 2 [file CHEM-32-e71198-s002.pdf]

Organelle-Resolved Tetrazine-*trans*-Cyclooctene Click Chemistry for Cargo Delivery and Release

Oleh Durydivka, Marek Chovanec, Rastislav Dzijak, Michal Rahm, Jachym Hrusak, Paul E. Reyes-Gutierrez, and Milan Vrabel*

Institute of Organic Chemistry and Biochemistry of the Czech Academy of Sciences, Flemingovo nám. 2, 160 00, Prague, Czech Republic

*Correspondence: vrabel@uochb.cas.cz

Tables S1-3

Table S1. The overview of targeting groups, commercial trackers, and genetic markers used in the study.

Cell compartment	Targeting group	Chemical tracker	Genetic marker
Plasma membrane	Alkyl chain (C = 11) + Sulphonyl	CellMask Deep Red Plasma Membrane Stain	CA12-Spark
Mitochondria	Triphenylphosphonium	MitoTracker Deep Red FM	COX4A-Spark
Nucleus	Hoechst 33258	DRAQ5 Fluorescent Probe	H2B-Spark
Endoplasmic reticulum	Pentafluorophenol<br>Propyl chloride<br>Sulphonylureas	BODIPY FL glibenclamide<br>BODIPY TR glibenclamide	SEC61B-Spark
Golgi apparatus	Myristate	BODIPY FL C_5-ceramide<br>BODIPY TR C_5-ceramide	B4GALT1-Spark
Lysosome	Morpholine<br>Dimethylamine	LysoTracker Green DND-26<br>LysoTracker Red DND-99	TMEM192-Spark

CA12, carbonic anhydrase 12; COX4A, N-terminal mitochondrial localization sequence from cytochrome c oxidase 4A; H2B, histone H2B; SEC61B, protein transport protein Sec61 subunit beta; B4GALT1, first sixty amino acids of beta-1,4-galactosyltransferase; TMEM192, transmembrane protein 192

Table S2. LogP, logS, and logD values calculated for the organelle-targeting probes using StarDrop.

	**logP**	**logD**	**logS**	**logS at pH 7.4**
PM-TCO (**1**)	5.955	1.87	2.59	0.9612
PM-Tz (**2**)	5.765	1.756	2.153	0.7354
PM-Coum (**3**)	5.771	2.433	2.397	1.753
Mito-TCO (**4**)	8.516	3.635	1.082	-0.081
Mito-Tz (**5**)	7.867	3.777	0.3109	0.0869
Mito-Coum (**6**)	8.459	4.86	0.1957	0.8954
Nucl-TCO (**7**)	4.745	4.407	1.591	1.86
Nucl-Tz (**8**)	4.52	4.536	0.9601	1.664
ER-F-TCO (**9**)	2.911	2.911	0.0037	0.0037
ER-F-Tz (**10**)	2.841	2.841	-0.526	-0.526
ER-Cl-TCO (**11**)	3.078	3.078	2.805	2.805
ER-Cl-Tz (**12**)	2.331	2.331	2.35	2.35
ER-Cl-Coum (**13**)	3.09	2.656	2.273	2.76
ER-SU1-TCO (**14**)	4.227	1.999	0.8608	1.868
ER-SU1-Tz (**15**)	3.219	2.039	-0.022	2.349
ER-SU1-Coum (**16**)	4.167	2.961	0.2176	3.139
ER-SU2-TCO (**17**)	3.391	3.391	2.214	2.214
ER-SU2-Tz (**18**)	2.622	2.622	1.353	1.353
Golgi-TCO (**19**)	5.799	5.799	1.293	1.293
Golgi-Tz (**20**)	5.889	5.889	0.5445	0.5445
Golgi-Coum (**21**)	6.116	3.571	0.5563	0.99
Lyso-M-TCO (**22**)	2.237	0.7355	4.57	2.6
Lyso-M-Tz (**23**)	1.691	0.5493	3.338	1.97
Lyso-M-Coum (**24**)	2.118	2.353	3.415	2.722
Lyso-N-TCO (**25**)	2.35	0.3065	4.779	2.583
Lyso-N-Coum (**26**)	2.477	1.649	3.577	2.818

Table S3. The potency of the products of the click-to-release reaction and the controls in the cell viability assay after 72 h incubation.

	**logIC_{50} ± SEM, M**	**IC_{50}, μM**	**% of Doxo (95% CI)**
10 μM Nucl-TCO (**7**) + Doxo-rTz (**33**)	-6.67 ± 0.06	0.22 ± 0.03	115 (76 to 174)
1 μM Mito-TCO (**4**) + Doxo-rTz (**33**)	-6.34 ± 0.09	0.46 ± 0.09	244* (146 to 407)
10 μM Mito-TCO (**4**) + Doxo-rTz (**33**)	-6.50 ± 0.08	0.32 ± 0.06	170* (104 to 277)
10 μM PM-TCO (**1**) + Doxo-rTz (**33**)	-6.20 ± 0.08	0.63 ± 0.12	338* (204 to 557)
10 μM ER-F-TCO (**9**) + Doxo-rTz (**33**)	-6.78 ± 0.07	0.17 ± 0.03	89 (57 to 137)
Veh + Doxo-rTz (**33**)	-6.15 ± 0.05	0.71 ± 0.07	379* (262 to 549)
10 μM Lyso-M-Tz (**23**) + Doxo-rTCO (**34**)	-6.54 ± 0.05	0.29 ± 0.03	154* (105 to 225)
Veh + Doxo-rTCO (**34**)	-5.44 ± 0.04	3.64 ± 0.32	1944* (1396 to 2724)
Doxo (**30**)	-6.73 ± 0.07	0.19 ± 0.03	100 (65 to 155)

* $p < 0.05$ vs. Doxo (**30**) by 95% CI
