# Supplementary material for: Organelle‐Resolved Tetrazine‐trans‐Cyclooctene Click Chemistry for Cargo Delivery and Release
Source: Chemistry. 2026 Jun 1;32(35):e71198. doi: 10.1002/chem.71198 (PMC13587637; doi:10.1002/chem.71198)
Supplement: Supplementary file 3 [file CHEM-32-e71198-s003.pdf]

Organelle-Resolved Tetrazine-*trans*-Cyclooctene Click Chemistry for Cargo Delivery and Release

Oleh Durydivka, Marek Chovanec, Rastislav Dzijak, Michal Rahm, Jachym Hrusak, Paul E. Reyes-Gutierrez, and Milan Vrabel*

Institute of Organic Chemistry and Biochemistry of the Czech Academy of Sciences, Flemingovo nám. 2, 160 00, Prague, Czech Republic

*Correspondence: vrabel@uochb.cas.cz

Figures S1-14

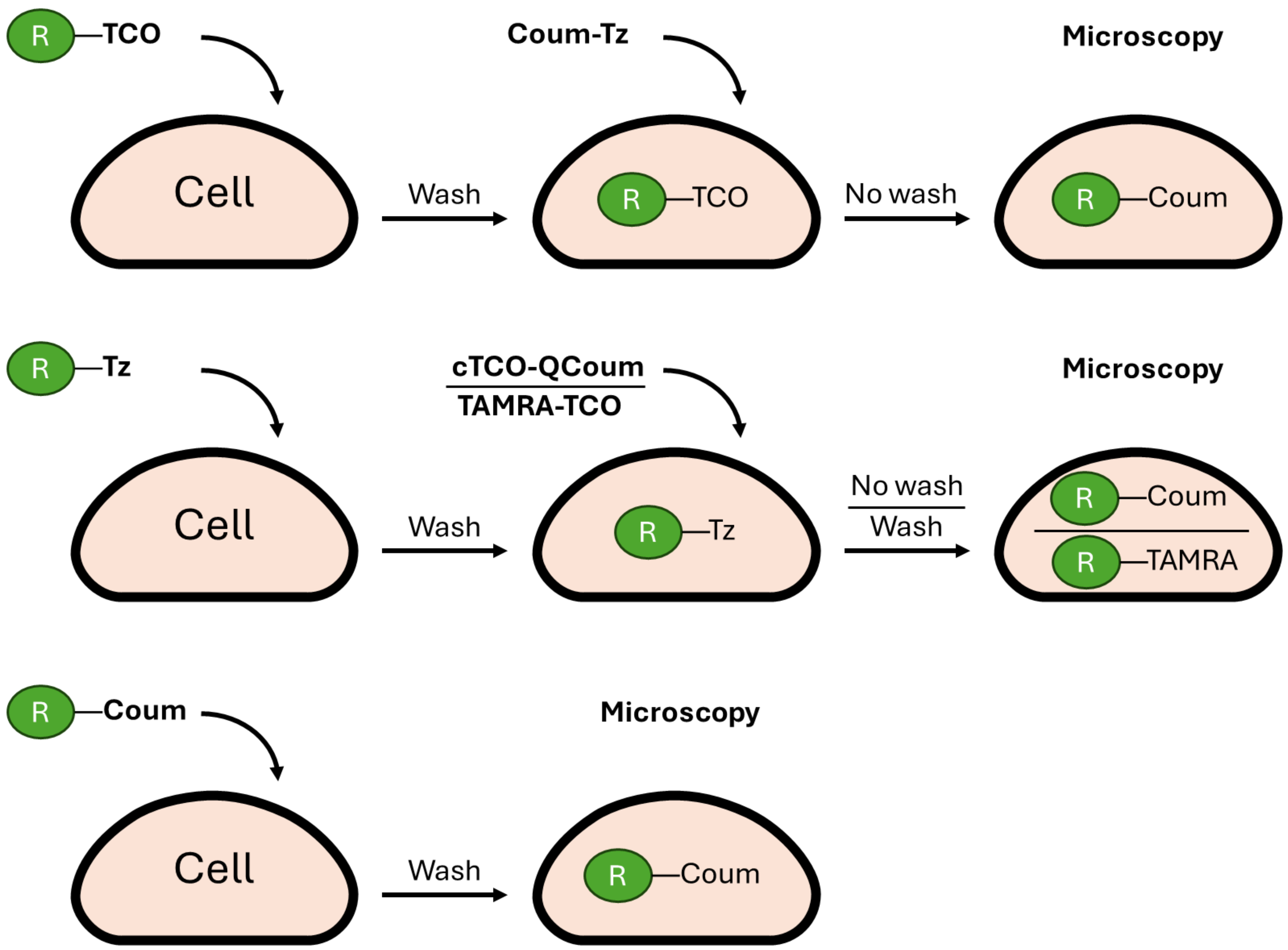

Suppl. Figure 1. Scheme of the click reaction-mediated labeling protocol. After the initial wash (not shown), the cells are incubated with TCO-, Tz-, or Coum-containing organelle-targeting probes, which is followed by another wash. The second incubation with Coum-Tz or cTCO-QCoum does not require a washing step because these fluorogenic compounds become fluorescent only after the click reaction takes place; therefore, after Incubation with these compounds, the cells are directly visualized under the microscope, unlike TAMRA-TCO, which requires washing.

Microscopy image figure captions:

Figures S2-7. Click-mediated labeling of organelle-targeting probes incubated with U2OS cells. The probe distribution in the cells (red color in composite images) was compared with the distribution of organelle markers (fused with Spark fluorescent protein) or chemical trackers (both in green color in composite images). Background intensities of the labeling fluorophores are shown, with image intensities adjusted in the same way as for the click labeling images. In addition, the distributions of organelle-targeting probes containing fluorophores are shown, as well as colocalization of organelle markers and chemical trackers. Scale bar 10 µm.

Figure S8. Subcellular distribution of doxorubicin and its derivatives in U2OS cells compared with the chemical organelle trackers. Scale bar 10 µm.

Figures S9-11. Click-mediated targeting of doxorubicin derivatives. The fluorescence of doxorubicin labeling products is compared with that of the chemical organelle trackers. Background labeling of the doxorubicin derivatives and the organelle-targeting probes alone is shown with image intensities adjusted in the same way as for the click labeling images. The distribution of the released doxorubicin is compared with the DRAQ5 nuclear labeling. Scale bar 10 µm.

Figure S2

Plasma membrane-targeting probes

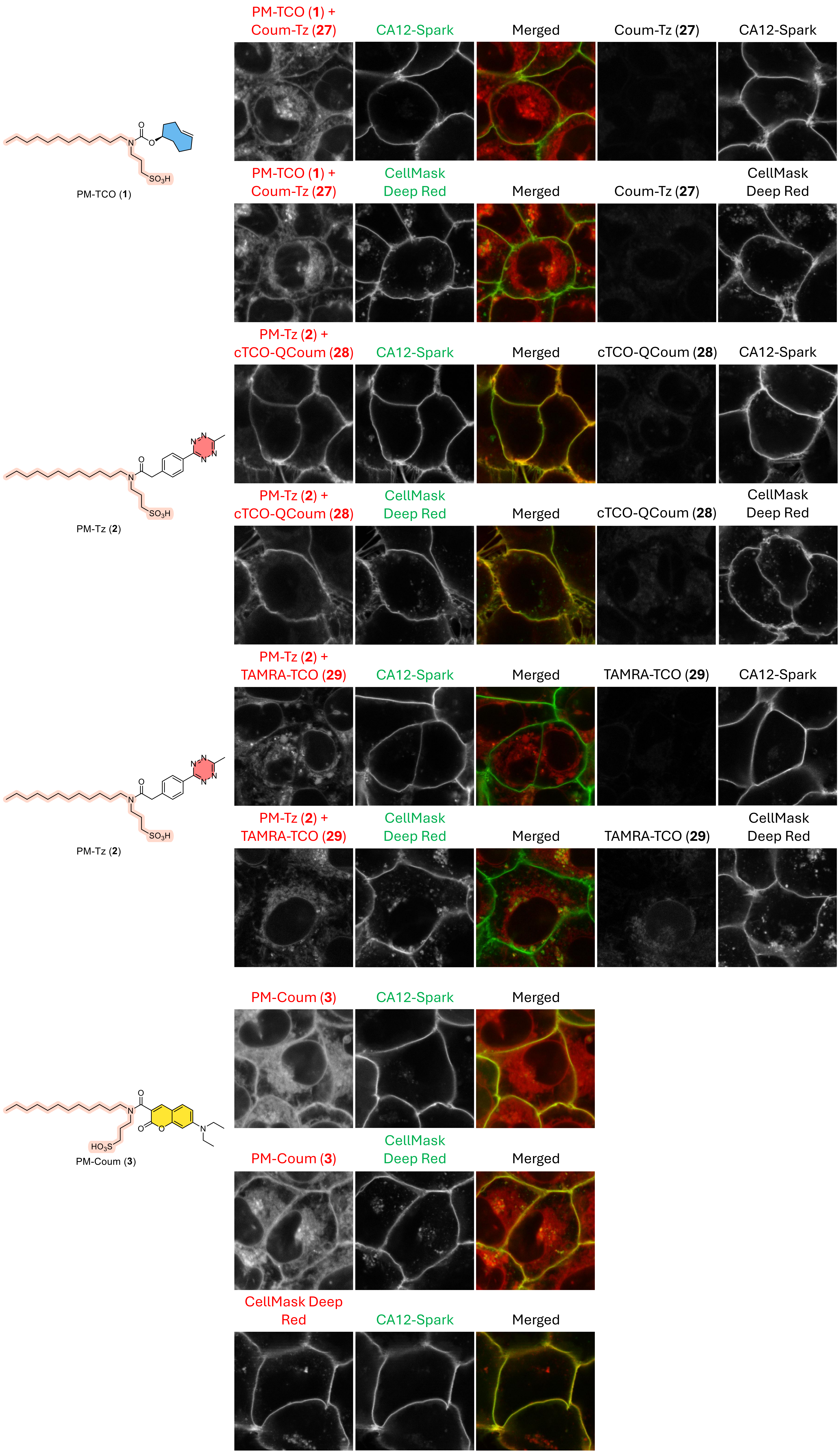

Figure S3

Mitochondrial-targeting probes

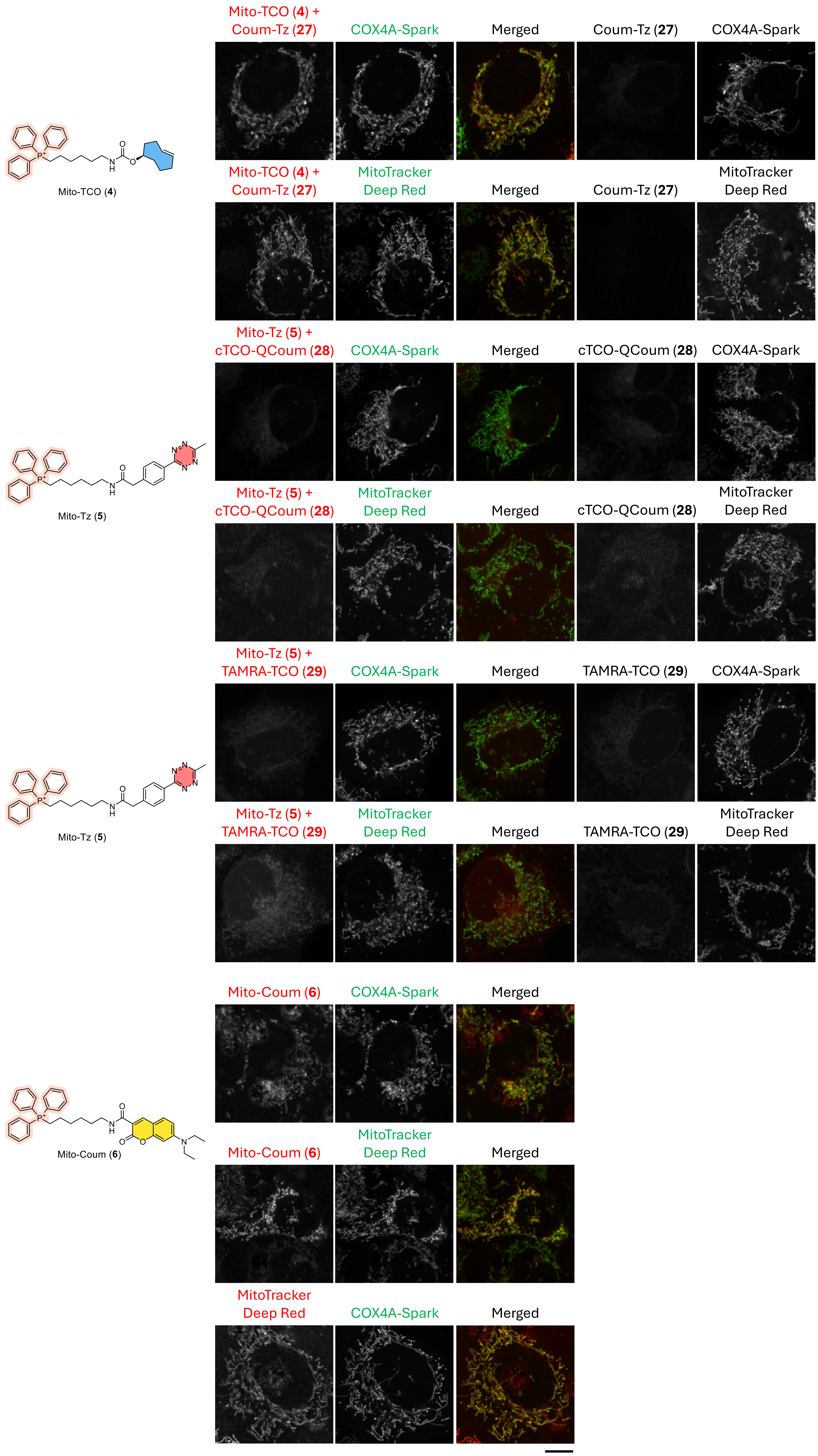

Figure S4

Nuclear-targeting probes

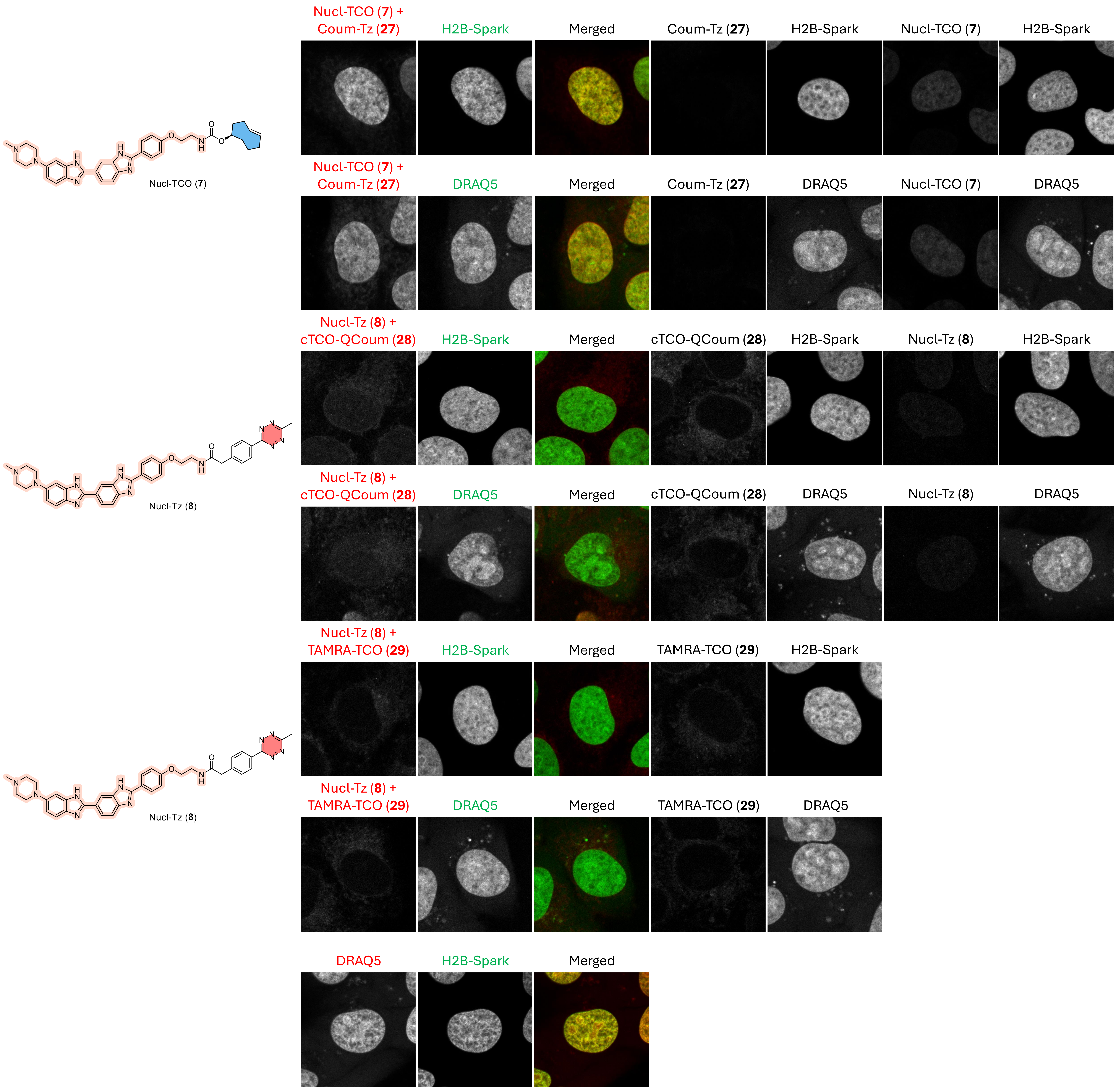

Figure S5

Endoplasmic reticulum-targeting probes

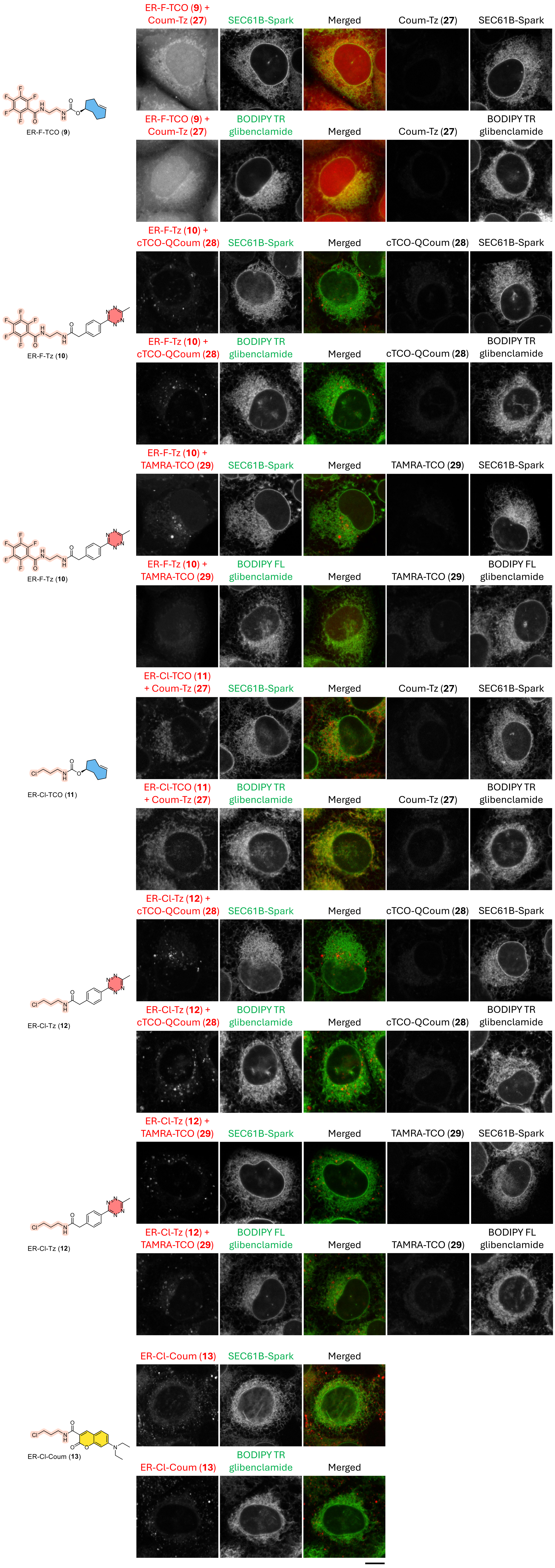

Figure S5

Endoplasmic reticulum-targeting probes (continued)

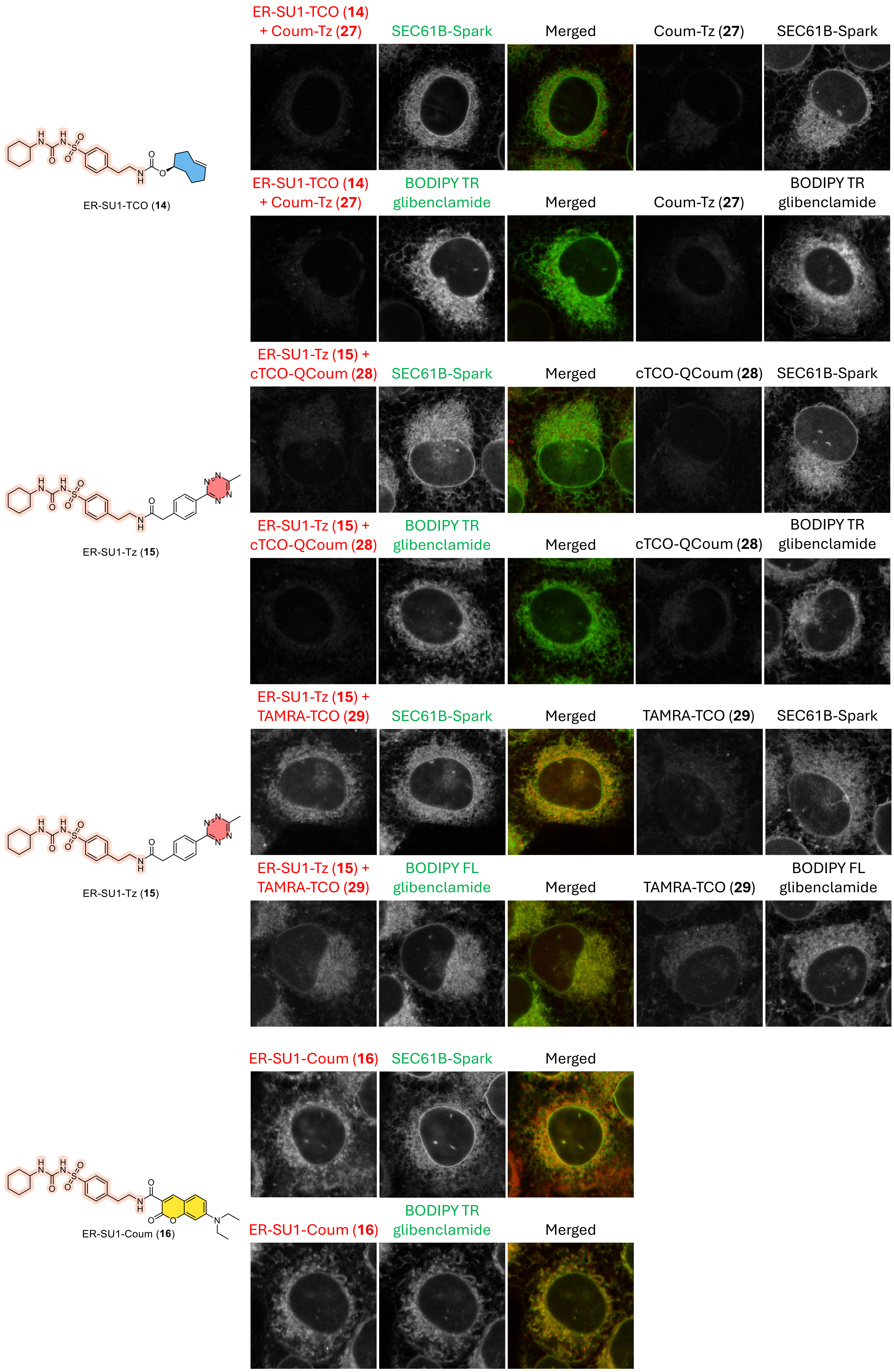

Figure S5

Endoplasmic reticulum-targeting probes (continued)

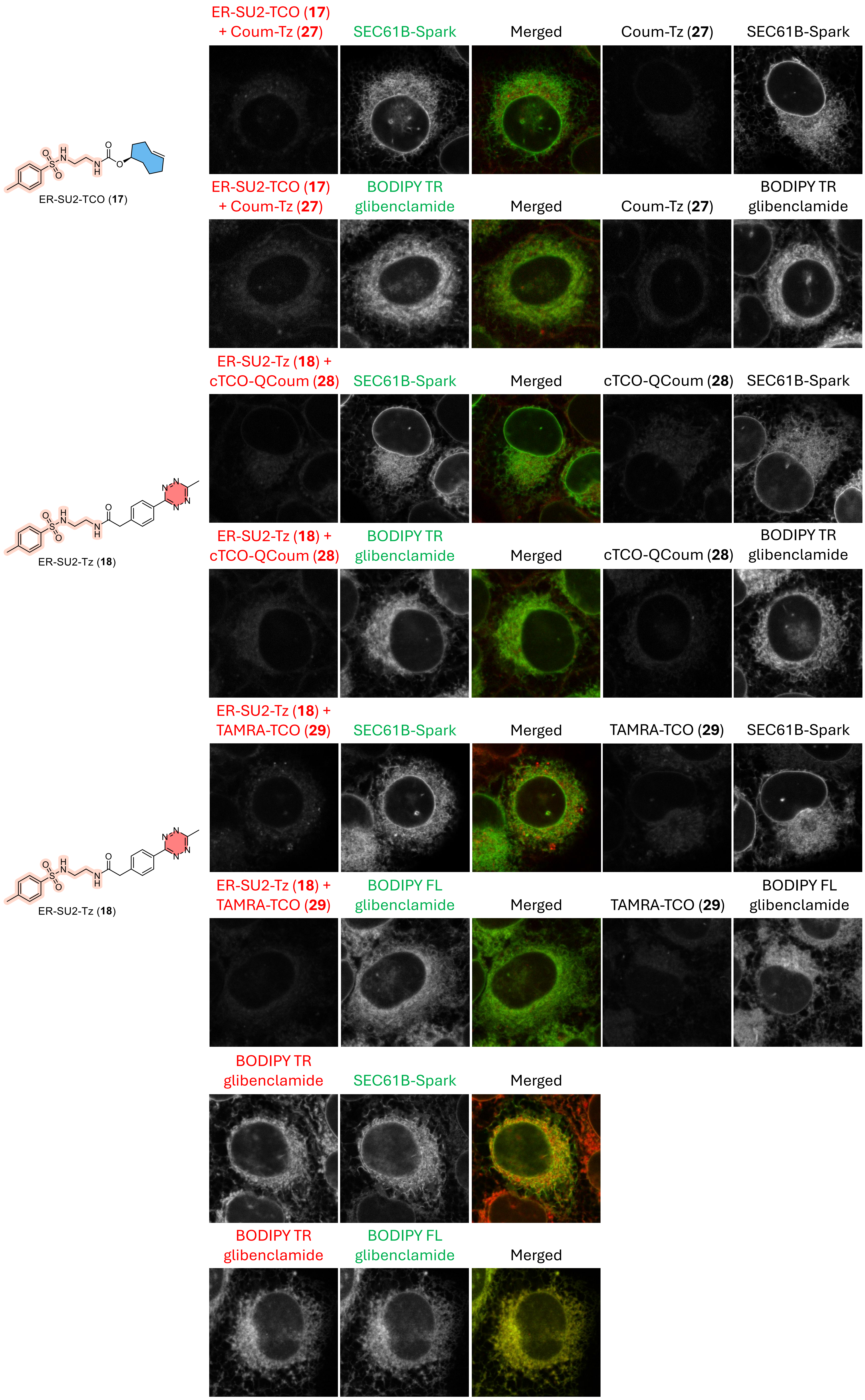

Figure S6

Golgi apparatus-targeting probes

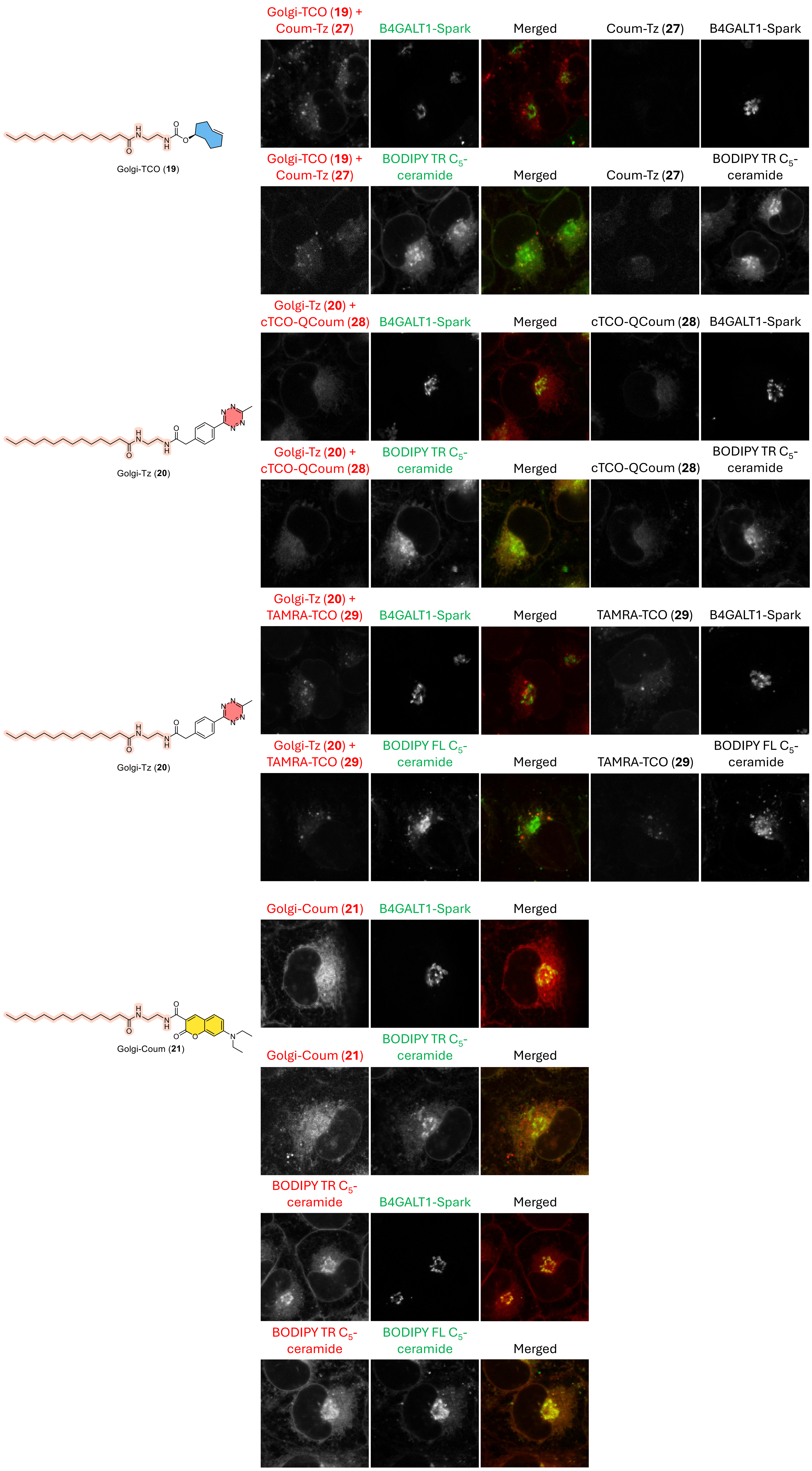

Figure S7

Lysosomal-targeting probes

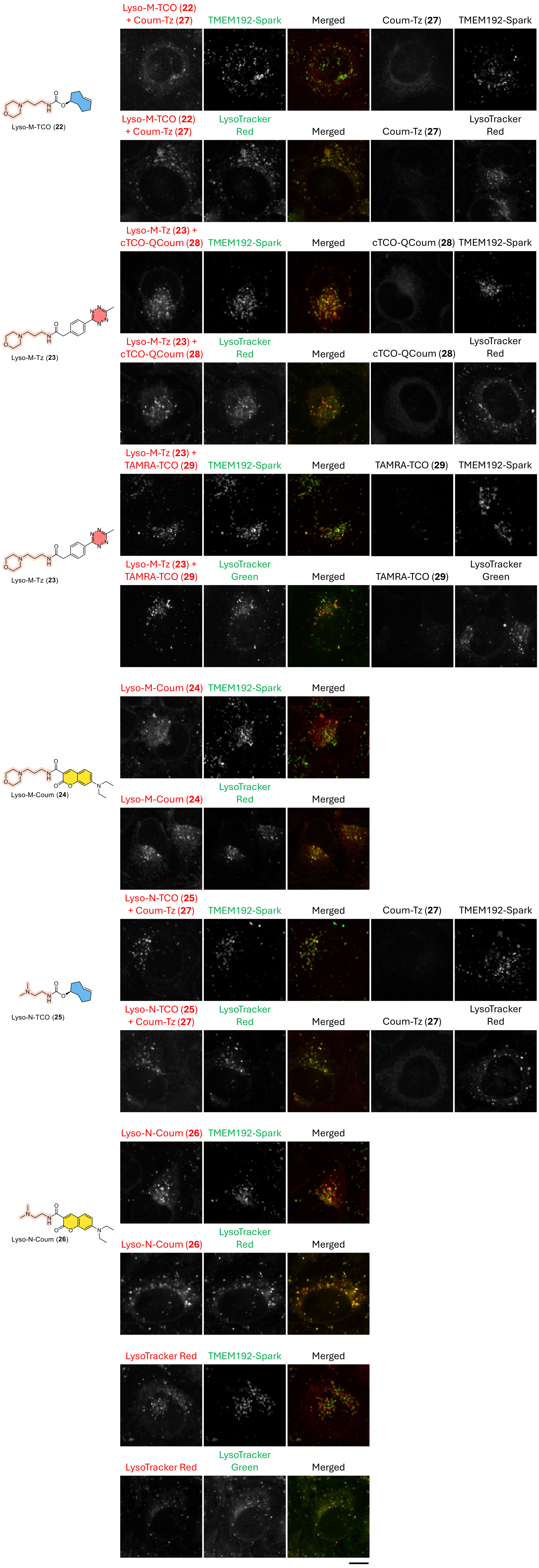

Figure S8

Doxorubicins – subcellular distribution

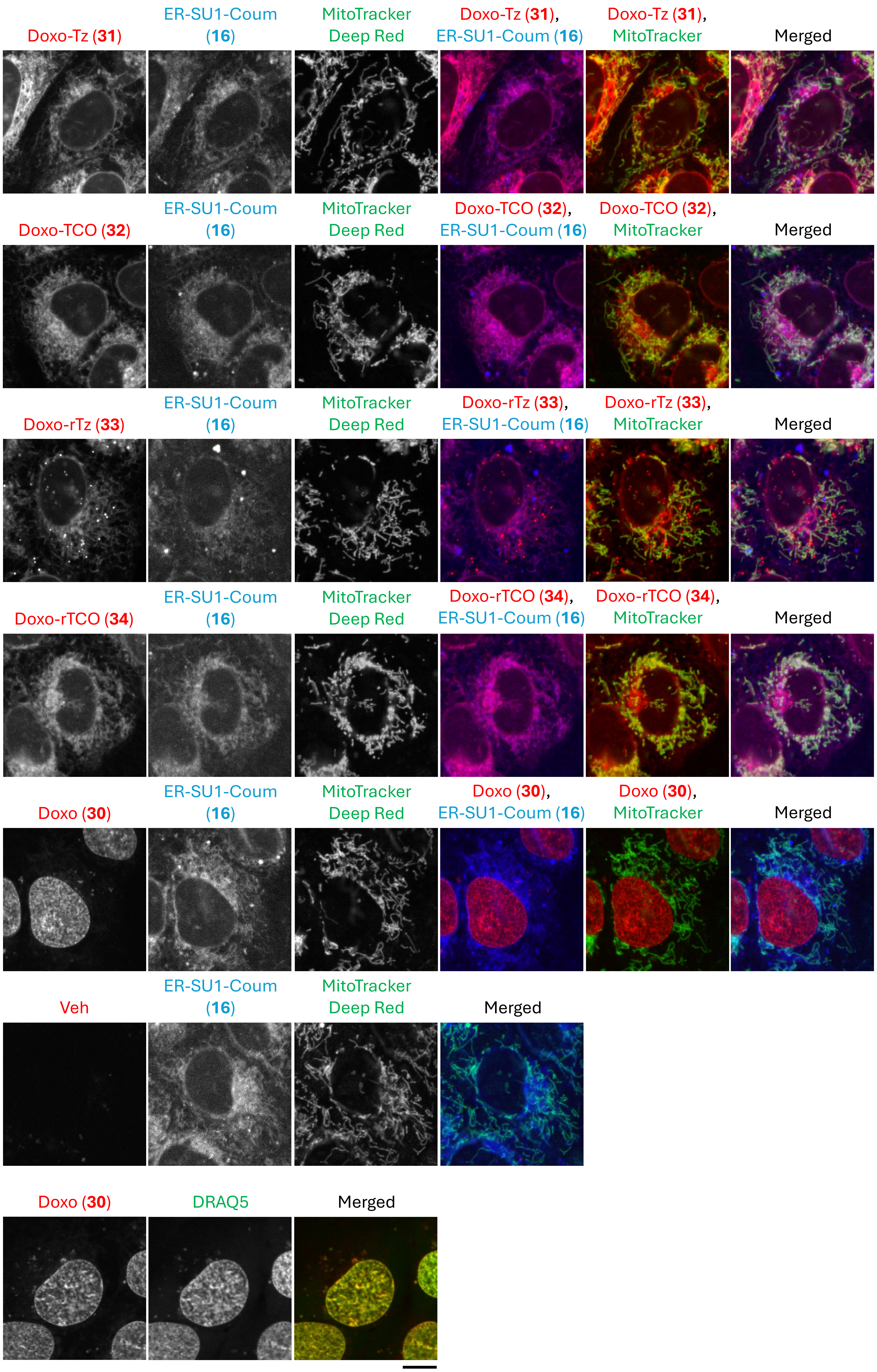

Figure S9

Doxorubicins – click-mediated localization

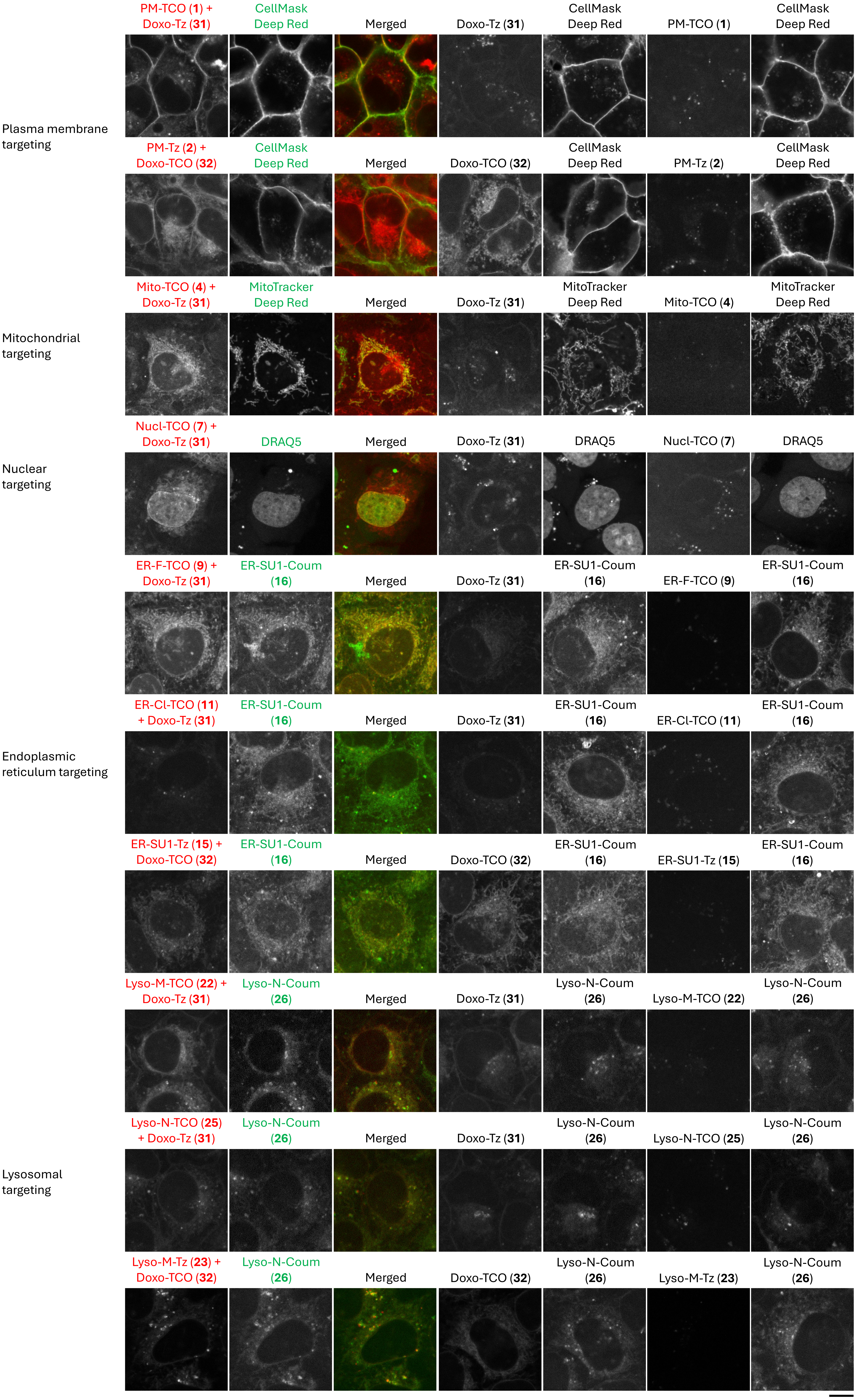

Figure S10

Doxorubicins – click-to-release

1 h post click

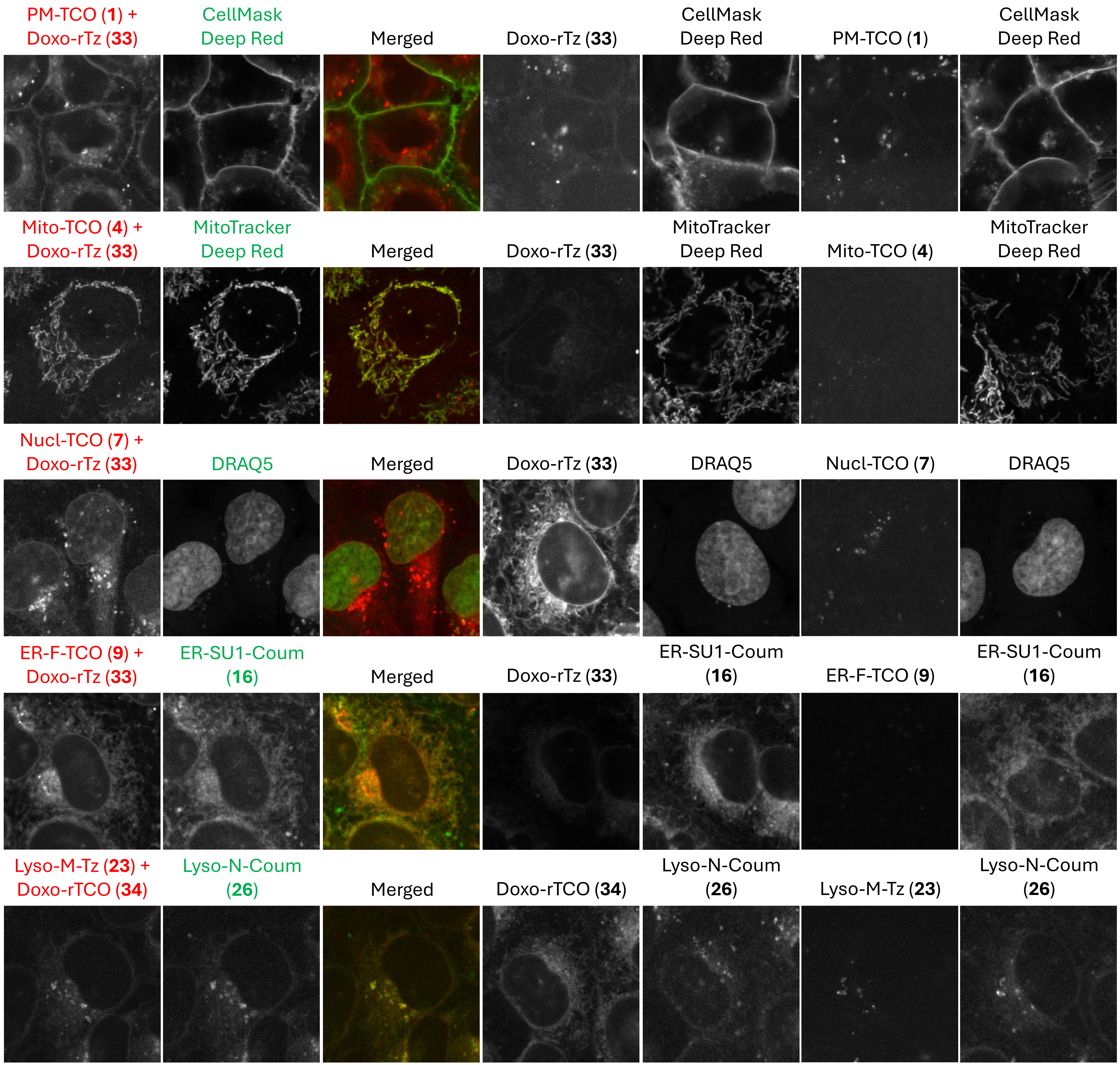

Figure S11

Doxorubicins – click-to-release

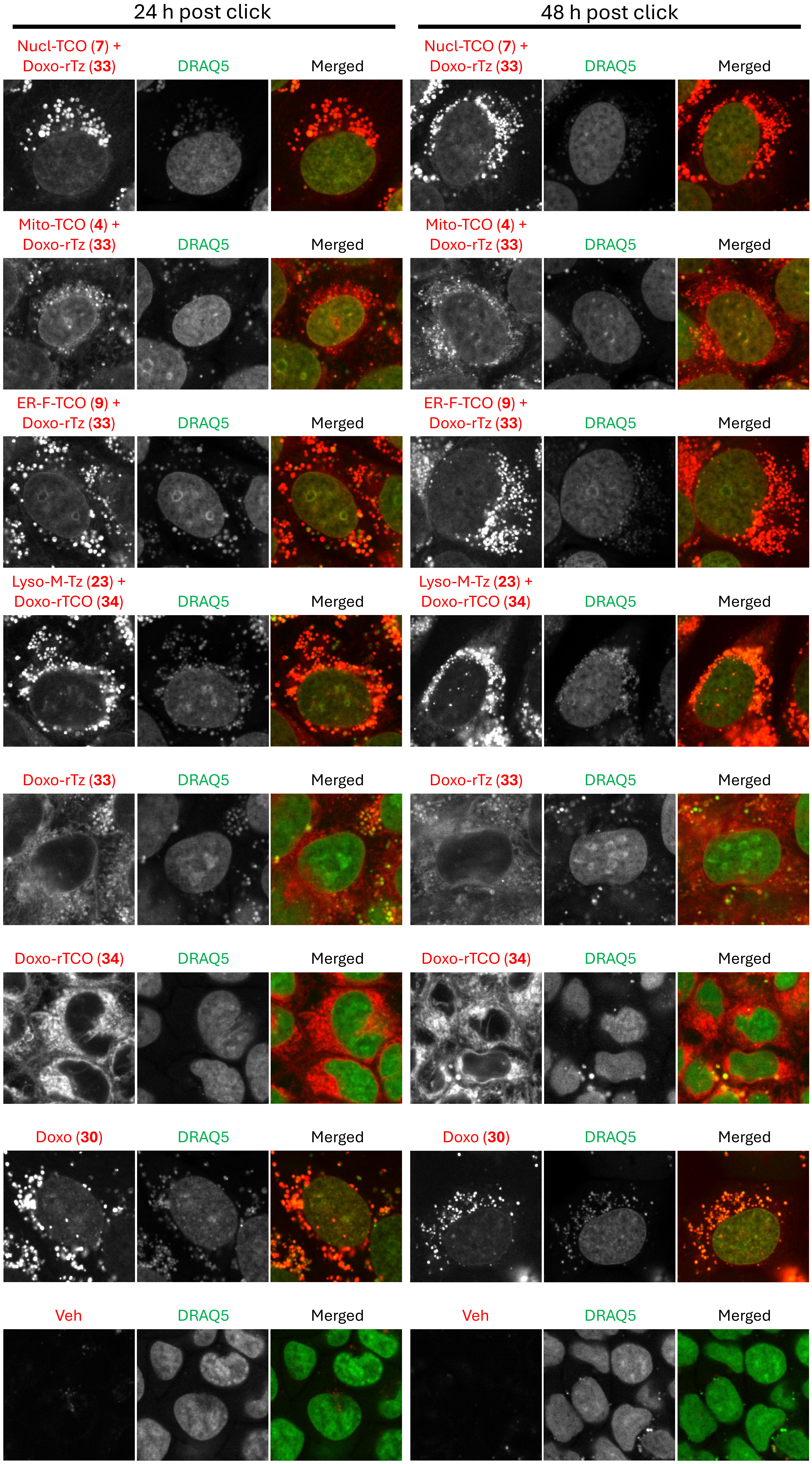

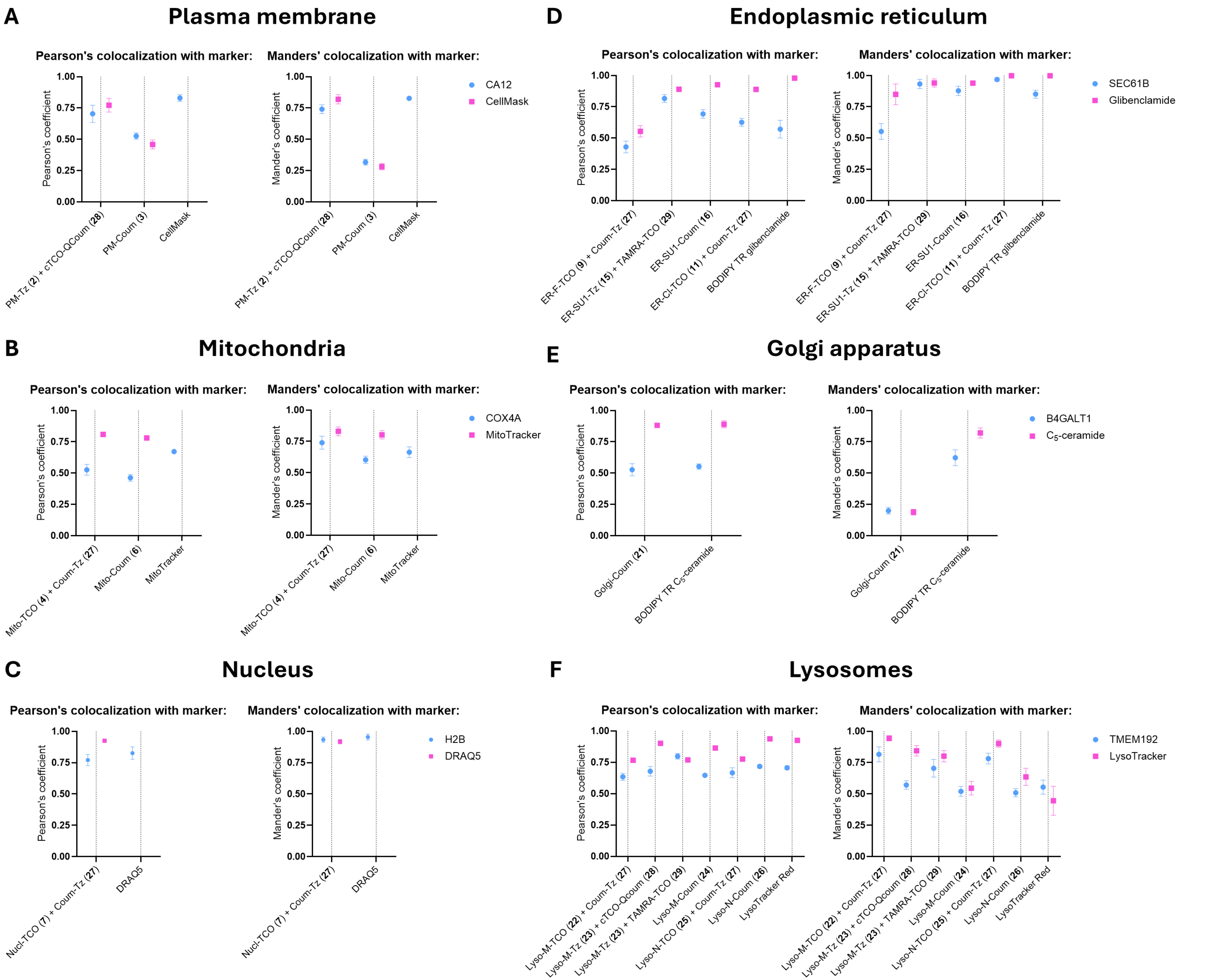

Figure S12. Colocalization quantification of the subcellular localization of the organelle-targeting probes targeting the plasma membrane (A), mitochondria (B), nucleus (C), endoplasmic reticulum (D), Golgi apparatus (E), and lysosomes (F). Pearson's and Manders' correlation coefficients are shown for the colocalization with the respective chemical trackers or genetic markers. Data are means ± SEM.

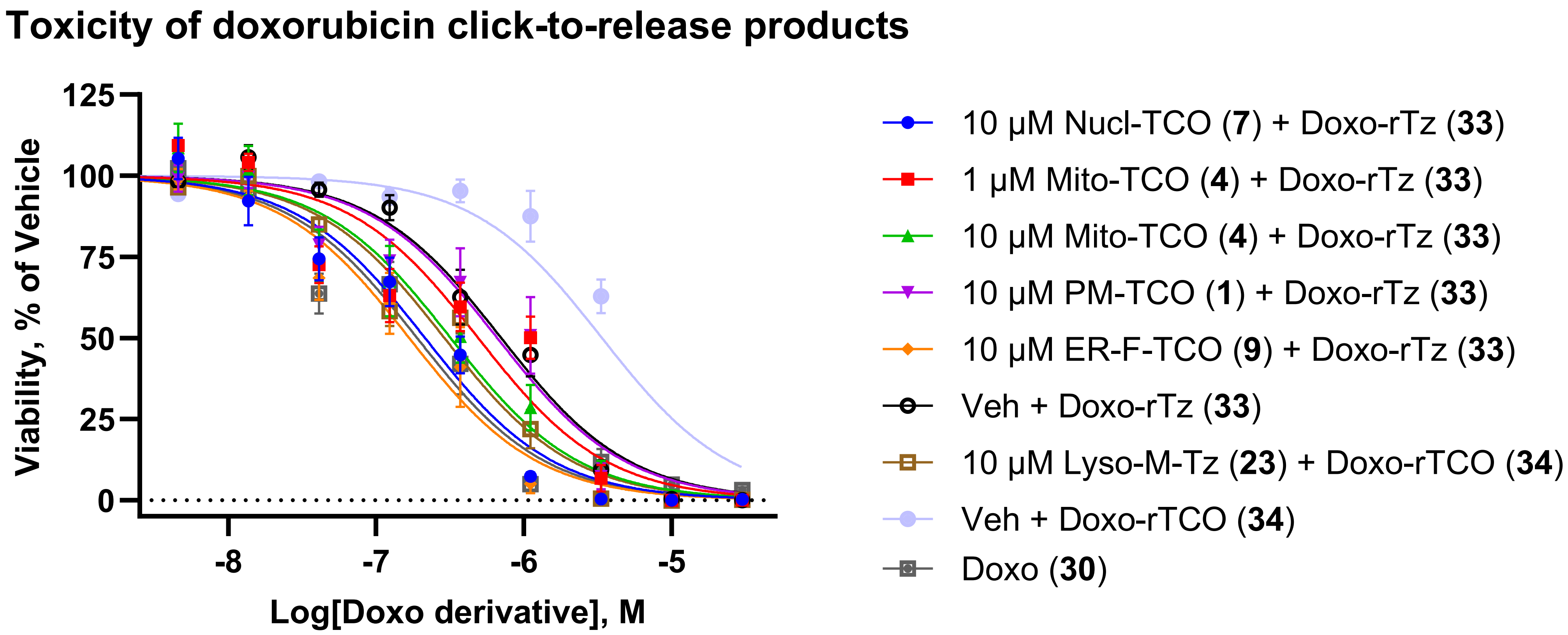

Figure S13. The effect of doxorubicin click-to-release products on cell viability. U2OS cells were incubated with the organelle-targeting probes for 1 h (15 min for the PM probe) or vehicle and then with the corresponding doxorubicin derivative or native doxorubicin for 72 h under standard cultivation conditions. Cell viability was measured at the end of the incubation using PrestoBlue reagent. Data are means ± SEM from four independent experiments.

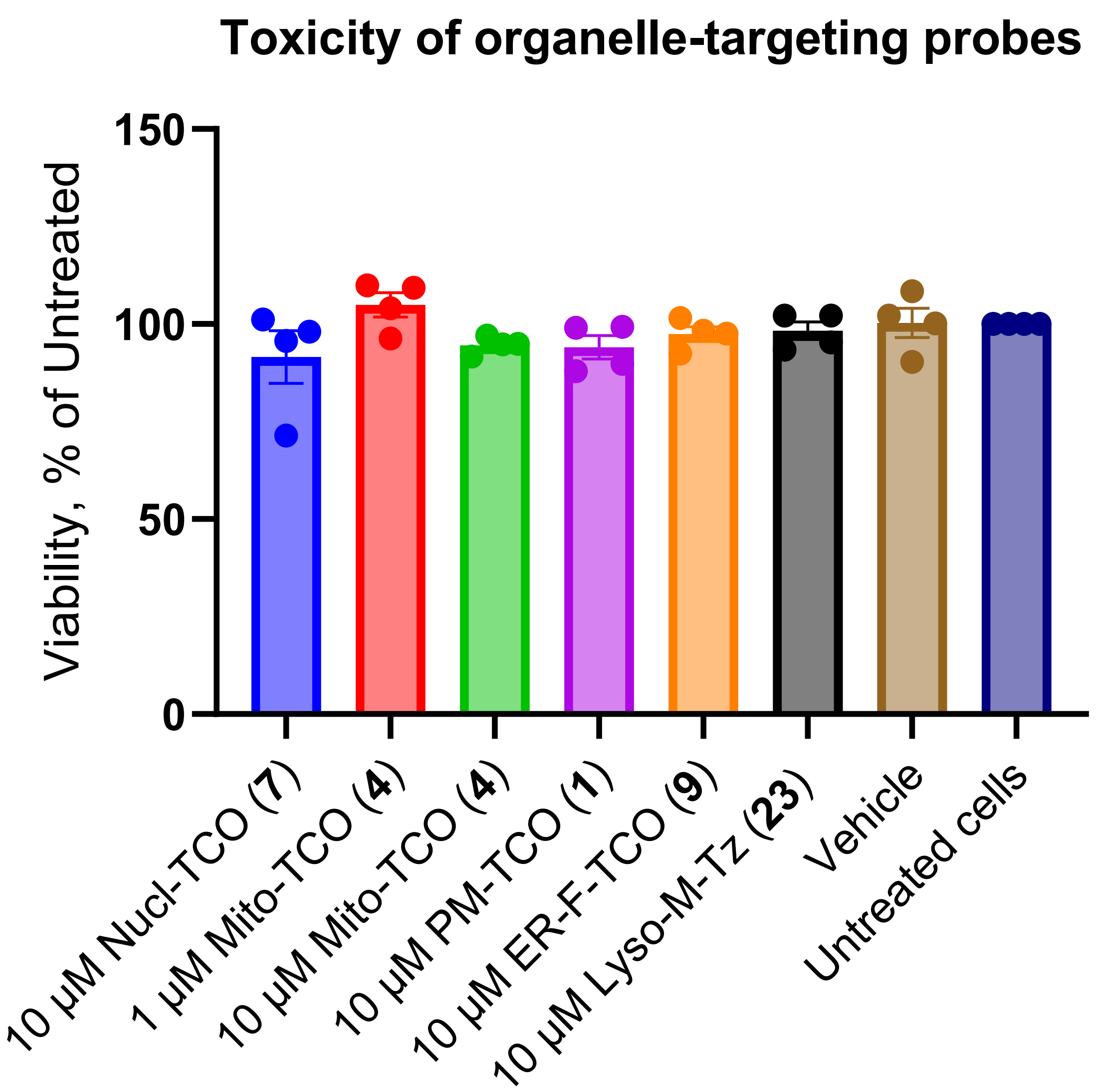

Figure S14. The effect of organelle-targeting probes on cell viability. U2OS cells were incubated with the indicated concentrations of the probes for 1 h (15 min for the PM probe) or vehicle. Data are means ± SEM from four independent experiments. p = 0.1882 by ANOVA.
